# Evidence for ligand- and solvent-induced disproportionation of uranium(IV)

**DOI:** 10.1038/s41467-021-25151-z

**Published:** 2021-08-10

**Authors:** Jingzhen Du, Iskander Douair, Erli Lu, John A. Seed, Floriana Tuna, Ashley J. Wooles, Laurent Maron, Stephen T. Liddle

**Affiliations:** 1 Department of Chemistry, The University of Manchester, Manchester, UK; 2 LPCNO, CNRS & INSA, Université Paul Sabatier, Toulouse, France; 3 Department of Chemistry and Photon Science Institute, The University of Manchester, Manchester, UK

**Keywords:** Coordination chemistry, Inorganic chemistry, Theoretical chemistry

## Abstract

Disproportionation, where a chemical element converts its oxidation state to two different ones, one higher and one lower, underpins the fundamental chemistry of metal ions. The overwhelming majority of uranium disproportionations involve uranium(III) and (V), with a singular example of uranium(IV) to uranium(V/III) disproportionation known, involving a nitride to imido/triflate transformation. Here, we report a conceptually opposite disproportionation of uranium(IV)-imido complexes to uranium(V)-nitride/uranium(III)-amide mixtures. This is facilitated by benzene, but not toluene, since benzene engages in a redox reaction with the uranium(III)-amide product to give uranium(IV)-amide and reduced arene. These disproportionations occur with potassium, rubidium, and cesium counter cations, but not lithium or sodium, reflecting the stability of the corresponding alkali metal-arene by-products. This reveals an exceptional level of ligand- and solvent-control over a key thermodynamic property of uranium, and is complementary to isolobal uranium(V)-oxo disproportionations, suggesting a potentially wider prevalence possibly with broad implications for the chemistry of uranium.

## Abstract (web summary)

Disproportion of uranium(IV) is rare, as it is usually the stable product of uranium(III) or (V) disproportionation. Here, the authors report uranium(IV) disproportionation to uranium(III) and (V) revealing ligand and solvent control over a key thermodynamic property of uranium

## Introduction

Disproportionation, where an ion in an oxidation state converts to ions with two different oxidation states, one higher and one lower, has fundamentally underpinned and defined the chemistry of metal ions for over two centuries^1,2^. Disproportionation of uranium was established 96 years ago^3,4^, and the status quo remained unchanged over seven decades^5,6^, where uranium(V) disproportionates to uranium(IV) and (VI), Eq. (1), and uranium(III) disproportionates to uranium(0) and (IV), Eq. (2). These intrinsic disproportionations, unless kinetically blocked, occur over a wide range of solvents, supporting ligands, and synthetic^7–9^ and environmental scenarios^10,11^, reflecting the inherent thermodynamic instability of these two uranium oxidation states.

1 $$2\,{{{{{\rm{U}}}}}}^{{{{{\rm{V}}}}}}{\,}(5{{{{{\rm{f}}}}}}^1)\,{{{{{\rm{\to }}}}}}\,{{{{{\rm{U}}}}}}^{{{{{\rm{IV}}}}}}(5{{{{{\rm{f}}}}}}^2)+{{{{{\rm{U}}}}}}^{{{{{\rm{VI}}}}}}\,(5{{{{{\rm{f}}}}}}^0),({{{{{\rm{est}}}}}}.{\,}1925)$$

2 $$4{\,}{{{{{\rm{U}}}}}}^{{{{{\rm{III}}}}}}{\,}(5{{{{{\rm{f}}}}}}^3){\,}{{{{{\rm{\to }}}}}}1{\,}{{{{{\rm{U}}}}}}^0{\,}(7{{{{{\rm{s}}}}}}^2{\,}6{{{{{\rm{d}}}}}}^1{\,}5{{{{{\rm{f}}}}}}^3)+3{\,}{{{{{\rm{U}}}}}}^{{{{{\rm{IV}}}}}}(5{{{{{\rm{f}}}}}}^2),({{{{{\rm{est}}}}}}.{\,}1953)$$

3 $$2{\,}{{{{{\rm{U}}}}}}^{{{{{\rm{IV}}}}}}{\,}(5{{{{{\rm{f}}}}}}^2){\,}{{{{{\rm{\to }}}}}}{\,}{{{{{\rm{U}}}}}}^{{{{{\rm{III}}}}}}{\,}(5{{{{{\rm{f}}}}}}^3)+{{{{{\rm{U}}}}}}^{{{{{\rm{V}}}}}}(5{{{{{\rm{f}}}}}}^1),{\,}(est.{\,\,}2016\,and\,this\,work)$$

In contrast to uranium(III) and (V), uranium(IV) and (VI) are conversely regarded as the most stable oxidation states of uranium in virtually all solvent media and ligand environments^7–13^. Indeed, the inherent thermodynamic properties that underpin the redox processes of each oxidation state of uranium are arguably often little changed by the chemical environment defined by ligands and solvent, and they are usually at best kinetically suppressed or contained rather than being decisively materially altered. Thus, whilst uranium(III) and (V) intrinsically engage in disproportionation reactions unless kinetically blocked, disproportionation of uranium(VI) has never been demonstrated. Only as recently as 2016 was uranium(IV) discovered to be capable of disproportionating to uranium(III/V), specifically where a bridging diuranium(IV)-nitride complex reacted with methyl-triflate to give a uranium(III)-triflate and a uranium(V)-imido complex, Equation (3)^14^. However, the factors that govern that remarkable example of uranium(IV) disproportionation remain unknown.

Here, we report an example of uranium(IV) disproportionation to uranium(III) and (V) (Eq. 3). Deprotonation of a uranium(IV)-amide to give -imido derivatives promotes, with mild heating, disproportionation to the corresponding uranium(III)-amide and uranium(V)-nitride complexes for three alkali metal salts in benzene, but not toluene, solvent. This reaction is conceptually the reverse reaction to the above example, namely imido to nitride/amide as opposed to nitride to imido/triflate. The disproportionation reaction reported here suggests an unusual level of external control over the thermodynamic properties of uranium, further diminishing the general imperviousness of uranium(IV) with respect to (III) and (V)^15^, and our results provide insight into the factors that are dictating the reactivity. The reactivity outlined here has parallels to that of uranyl(V)^10,11^, possibly suggesting a potentially wider prevalence in highly radiolytic and environmental scenarios^16^.

## Results

### Synthetic considerations

**Fig. 1:**
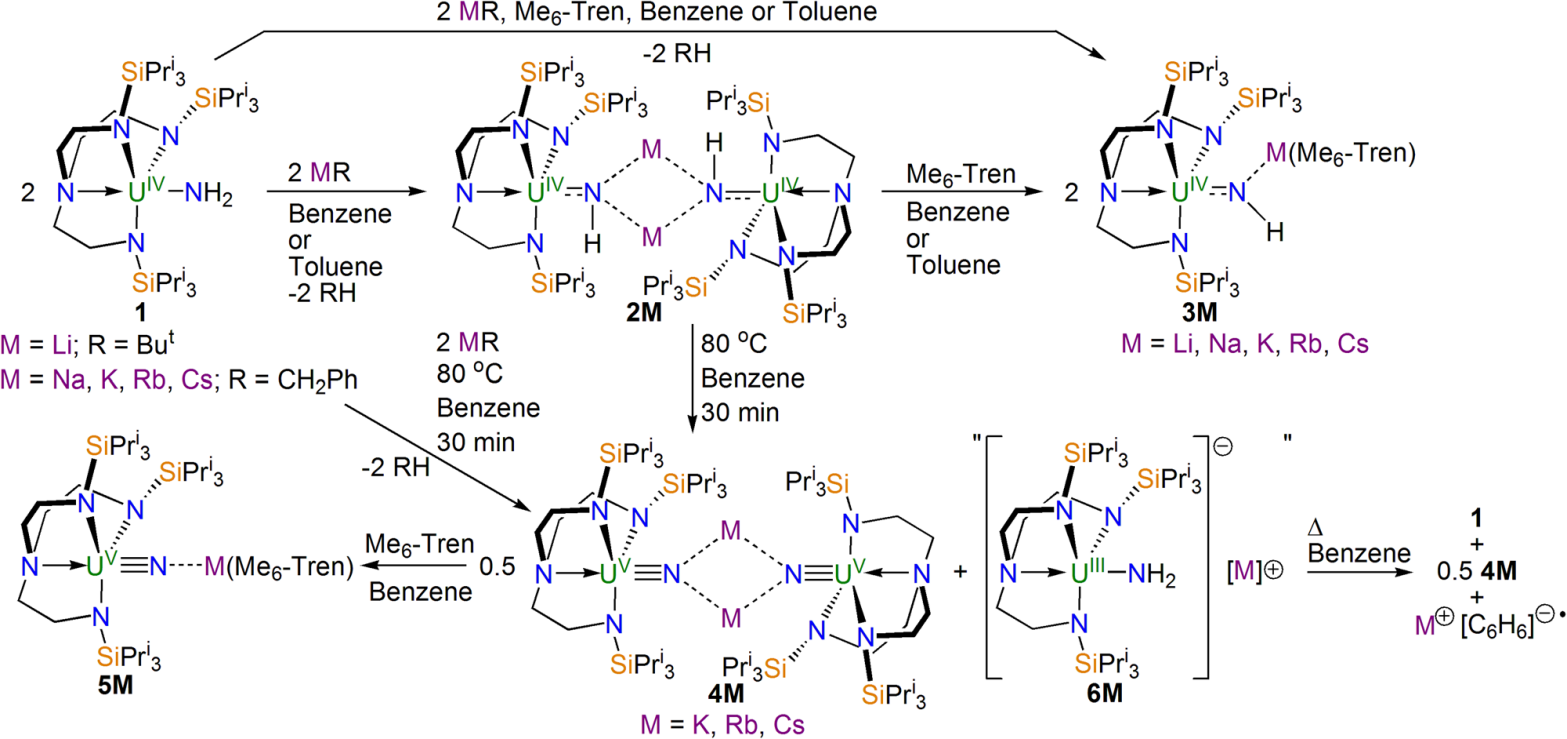
Synthesis of 2M–6M. Complex **1** is deprotonated by organo-alkali metal reagents to afford the imido dimers **2M**. The imido dimers **2M** can be converted to the imido monomers **3M** by addition of Me_6_-Tren. Either isolated or in situ prepared **2M** when heated in benzene afford the nitride dimers **4M**, which can be converted to the nitride monomers **5M** by addition of Me_6_-Tren. Complexes **6M** were not isolated, but their oxidation product, **1**, was isolated.

We reported previously^17^ that the uranium(IV)-amide complex [U^IV^(Tren^TIPS^)(NH_2_)] (**1**, Tren^TIPS^ = {N(CH_2_CH_2_NSiPr^i^_3_)_3_}^3−^) can be deprotonated by alkali metal alkyl reagents to produce the pink uranium(IV)-imido dimers [{U^IV^(Tren^TIPS^)(μ-NH)(μ-M)}_2_] (**2M**, M = Li, Na, K, Rb, Cs), Fig. 1, which are insoluble in benzene or toluene at ambient conditions. However, **2M** can be cleaved by addition of the capping agent N(CH_2_CH_2_NMe_2_)_3_ (Me_6_-Tren), producing the uranium(IV)-imido monomers [U^IV^(Tren^TIPS^)(μ-NH)(μ-M)(Me_6_-Tren)] (**3M**) in crystalline yields ranging from 35 to 76%, Fig. 1. Complexes **3M** are relatively more soluble in benzene or toluene than **2M**, rendering solution-state characterisation spectroscopically possible. Alternatively, **3M** can be prepared from **1** in a one-pot reaction instead of separate metallation then capping steps. Throughout both these reaction sequences the oxidation state of uranium remains unchanged at +4, and these reactions all work in benzene or toluene solvents. During the course of our study, we investigated the effect of thermolysis, and found that moderate heating (80 °C) of reaction mixtures of **1** and potassium, rubidium, or caesium alkyls in benzene did not afford **2K**, **2Rb**, and **2Cs**, respectively. Instead, a different outcome was observed, and the dark red uranium(V)-nitride dimers [{U^V^(Tren^TIPS^)(μ-N)(μ-M)}_2_] (**4M**, M = K, Rb, Cs)^18,19^ were isolated in crystalline yields of 18–30% (50% maximum yield for disproportionation reactions suggested by a uranium(V) product void of a N–H linkage, see below), Fig. 1. Given their structural similarities to **2K**/**Rb**/**Cs**, in order to enable meaningful comparisons to be made to monomeric **3K**/**Rb**/**Cs** and further confirm the identities of **4K**/**Rb**/**Cs**, we converted the latter dimeric trio to the dark red uranium(V)-nitride monomers [U^V^(Tren^TIPS^)(μ-N)(μ-M)(Me_6_-Tren)] (**5M**, M = K, Rb, Cs), and isolated them in crystalline yields of 46–76%. Fig. 1. In contrast, treatment of **1** with lithium and sodium alkyls does not furnish **4Li** and **4Na**, and instead **2Li** and **2Na** are consistently isolated. To complete and provide a calibrated **5M** series, we treated dimeric **4Li** and **4Na**, prepared by an established procedure^18^, with two equivalents of Me_6_-Tren to produce monomeric **5Li** and **5Na**. The NMR and IR spectra of **3M** and **5M** are consistent with their formulations (Supplementary Figs. 1–25).

### Crystallographic characterisation

**Fig. 2:**
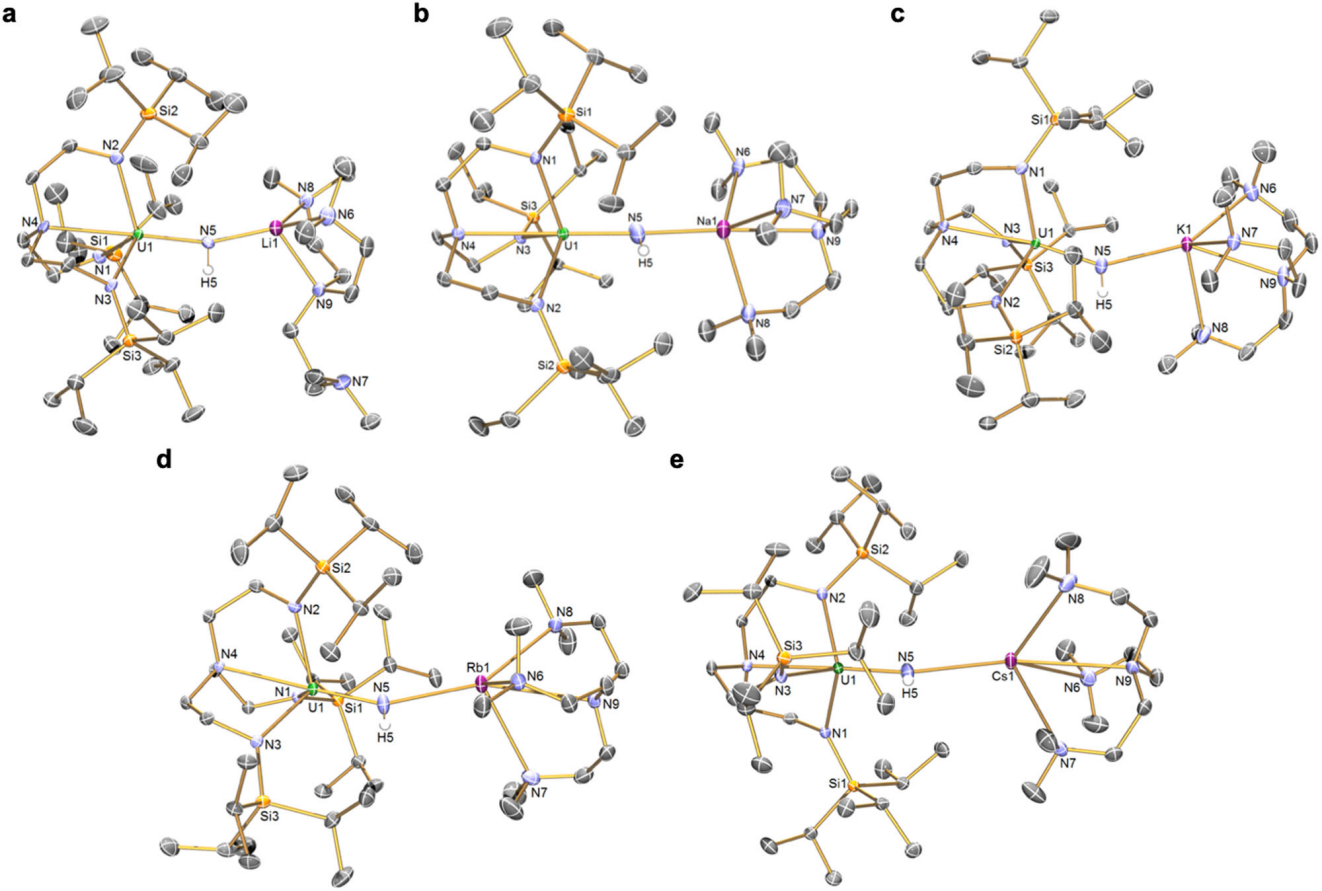
Solid-state single crystal structures of uranium(IV)-imido (3M) complexes at 150 K with 40% probability displacement ellipsoids. **a** complex **3Li**. **b** complex **3Na**. **c** complex **3K**. **d** complex **3Rb**. **e** complex **3Cs**. In each case all non-imido hydrogen atoms, lattice solvent molecules, and disorder components are omitted for clarity.

**Fig. 3:**
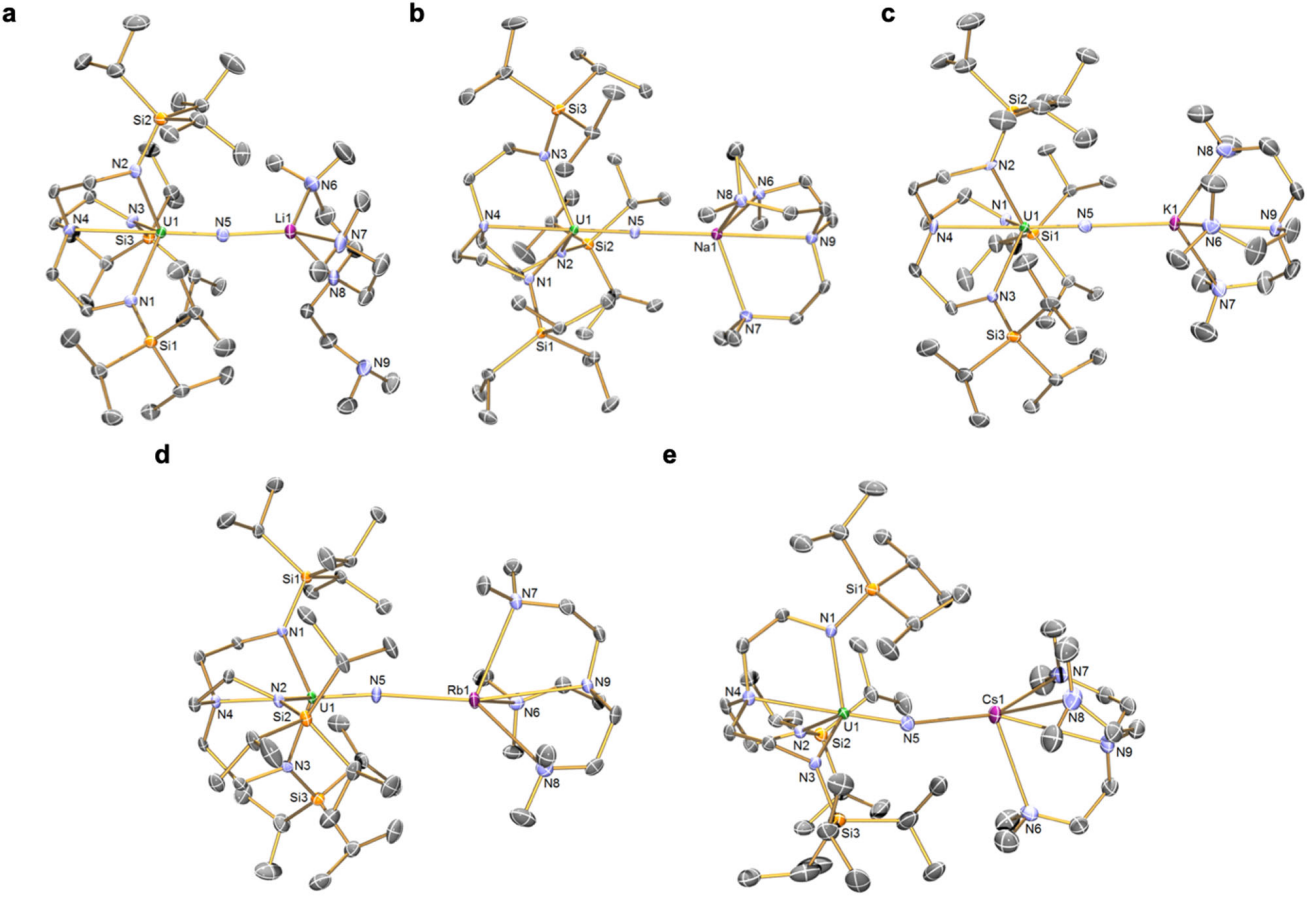
Solid-state single crystal structures of uranium(V)-nitrides (5M) at 150 K with 40% probability displacement ellipsoids. **a** complex **5Li**. **b** complex **5Na**. **c** complex **5K**. **d** complex **5Rb**. **e** complex **5Cs**. In each case all non-imido hydrogen atoms, lattice solvent molecules, and disorder components are omitted for clarity.

The characterisation data and crystallographic unit cell checks for **4K**/**Rb**/**Cs** produced by disproportionation match those of authentic samples^18–20^. The solid-state structures of the **3M** and **5M** series were determined, confirming their formulations, Figs. 2 and 3 (Supplementary Tables 1–6). For each complex, a triamidoamine-uranium component is bound to a central nitrogen, that resides in the pocket defined by the Tren^TIPS^ ligand, with that nitrogen atom bridging to a Me_6_-Tren-capped alkali metal. The salient, key difference between the **3M** and **5M** series is the presence of a hydrogen atom on the central nitrogen of the **3M** imido complexes, whereas no hydrogen atom is present in the **5M** nitride series, resulting in decisively different, and characteristic, U-N_imido_ bond lengths that average 2.007 Å (range: 1.979(4)–2.070(4) Å) for the **3M** series and shorter U-N_nitride_ distances averaging 1.810 Å (range: 1.792(4)–1.823(3) Å) for the **5M** complexes. For comparison, the U-N_imido_ distances in the **2M** series span the range 2.042(3)–2.135(3) Å^17^, uranium(V)-nitride complexes of the form [U^V^(Tren^TIPS^)(μ-N)(μ-M)(crown)] exhibit U-N_nitride_ distances spanning the range 1.803(5)–1.840(3) Å^18,19,21^, and the sum of the covalent radii for doubly and triply bonded uranium and nitrogen are 1.94 and 1.72 Å, respectively^22^. Notably, the U-N-M angles span a larger range for the **3M** series (141.01(19)–175.8(2)°) compared to the **5M** series (155.8(3)–177.66(18)°), consistent with the nitride ligand being a harder and less flexible ligand than imido.

### Magnetism and spectroscopic characterisation

**Fig. 4:**
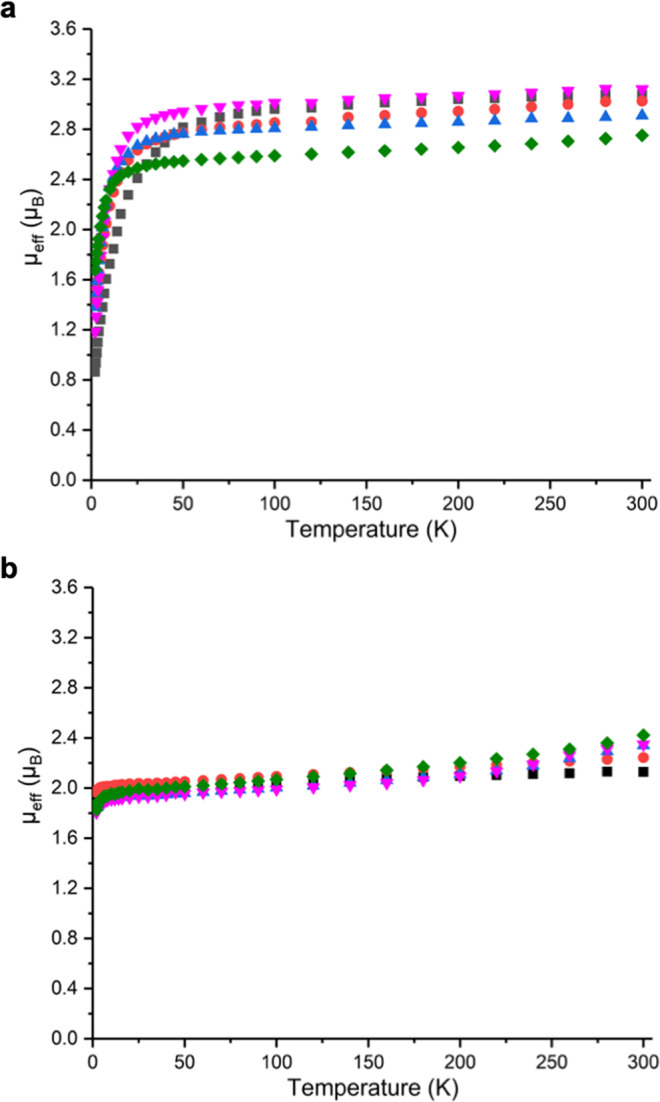
Variable-temperature effective magnetic moment SQUID data for uranium(IV)-imido (3M) and uranium(V)-nitride (5M) complexes. **a** μ_eff_ (μ_B_) vs T (K) data in a 0.5 T external field for **3Li** (black squares), **3Na** (red circles), **3K** (blue triangles), **3Rb** (violet triangles), and **3Cs** (green squares). **b** μ_eff_ (μ_B_) vs T (K) data in a 0.5 T external field for **5Li** (black squares), **5Na** (red circles), **5K** (blue triangles), **5Rb** (violet triangles), and **5Cs** (green squares).

Variable-temperature superconducting quantum interference device (SQUID) magnetic and UV/Vis/NIR and NMR spectroscopic data for the **3M** and **5M** series confirm their formal +4 and +5 uranium oxidation states, respectively (Supplementary Figs. 26–35 and Table 7). Specifically, the data for powdered samples of the **3M** series reveal magnetic moments over the range 2.75–3.12 μ_B_ at 300 K, Fig. 4a. These values are lower than the theoretical magnetic moment of 3.58 μ_B_ for a ^3^H_4_ uranium(IV) ion^9,23^, but well within the range of reported magnetic moments for uranium(IV)^24^. The magnetic moments of **3M** decrease little until ~50 K, then they decrease to magnetic moments spanning 0.86–1.68 μ_B_ at 2 K. This is not classical uranium(IV) magnetism behaviour, which tends to be a smooth decrease tending to zero with a net low temperature magnetic moment of ~0.5 μ_B_ due to temperature independent paramagnetism. However, it is characteristic of the magnetic response of uranium(IV) when bound to strong, multiply bonded donors, such as imidos^17^, carbenes^25,26^, phosphidos and arsenidos^27,28^, chalcogenidos^29^, and fluoride^30^, where the magnetic response of the ^3^H_4_ uranium ion, that is usually dominated by spin orbit coupling effects, is over-ridden by the ligand field^31–33^. In contrast, the data for powdered samples of the **5M**, Fig. 4b, series reveal magnetic moments that span 2.13–2.42 μ_B_ at 300 K. These data are slightly reduced from the theoretical magnetic moment of 2.54 μ_B_ for the ^2^F_5/2_ uranium(V) ion^9,23^, but are in agreement with uranium(V) magnetic moments generally^24^. The magnetic moment data for **5M** change little until ~20 K, at which point they fall sharply to magnetic moments over a narrow range of 1.81–1.97 μ_B_ at 2 K. This behaviour is typical of classical uranium(V) where this ^2^F_5/2_ ion is a magnetic doublet at all temperatures, with the low temperature decrease in magnetic moment due to depopulation of low-lying paramagnetic states ^9,23^.

**Fig. 5:**
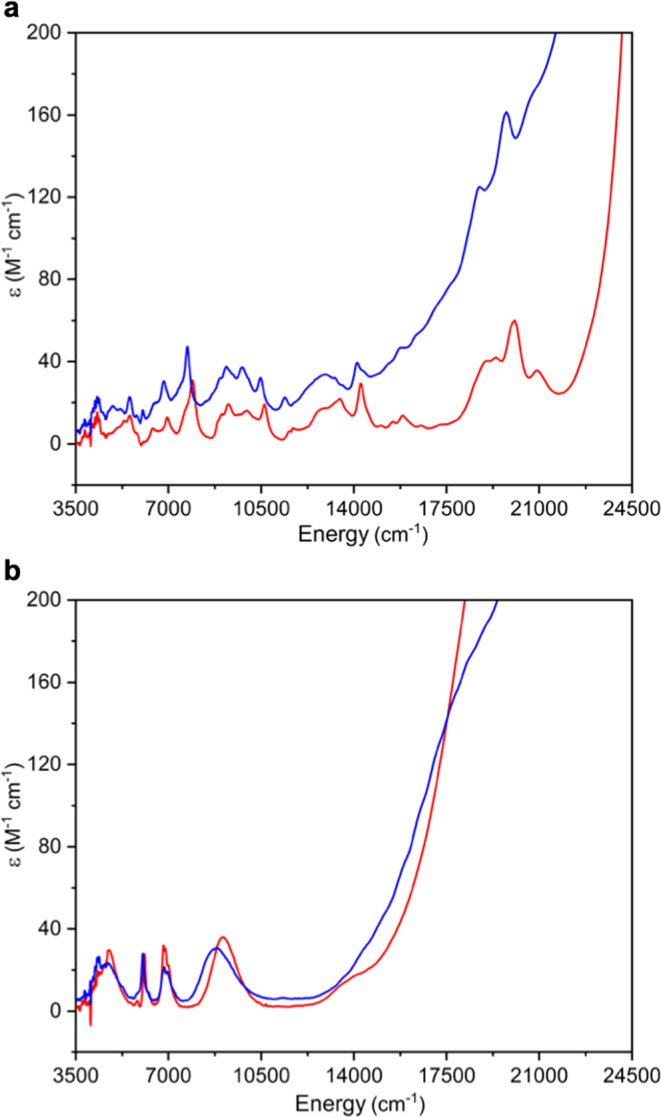
UV/Vis/NIR spectroscopic data for uranium(IV)-imido (3Li/Cs) and uranium(V)-nitrides (5Li/Cs) complexes. **a** ε (M^−1^ cm^−1^) vs energy (cm^−1^) of **3Li** (red trace) and **3Cs** (blue trace) in toluene. **b** ε (M^−1^ cm^−1^) vs energy (cm^−1^) of **5Li** (red trace) and **5Cs** (blue trace) in toluene.

The UV/Vis/NIR spectra of **3Li**, **3Cs**, **5Li**, and **5Cs** could be reliably acquired, Fig. 5, however the others could not due to limited solubility once isolated in crystalline form. For all complexes, strong charge transfer bands trail in from the UV region to ~17,000 cm^−1^. However, for the **3Li/Cs** complexes multiple and weak (ε = ≤60 M^−1^ cm^−1^) absorptions are present in the range 3500–17,000 cm^−1^ that are characteristic of intraconfigurational transitions of uranium(IV)^9,23,34^, whereas the **5Li/Cs** series exhibits the fingerprint absorptions for electronic transitions between the *J* = 5/2 and 7/2 multiplets of the ^2^F orbital manifold^35^, where *J* is the total angular momentum resulting from the interplay of the spin and unquenched orbital angular momenta. Where NMR data could be acquired, the ^29^Si resonances of **3Li**, **3Cs**, **5Li**, and **5Cs** fall in the expected ranges for uranium(IV) and (V), respectively ^36^.

### Disproportionation investigation

**Fig. 6:**
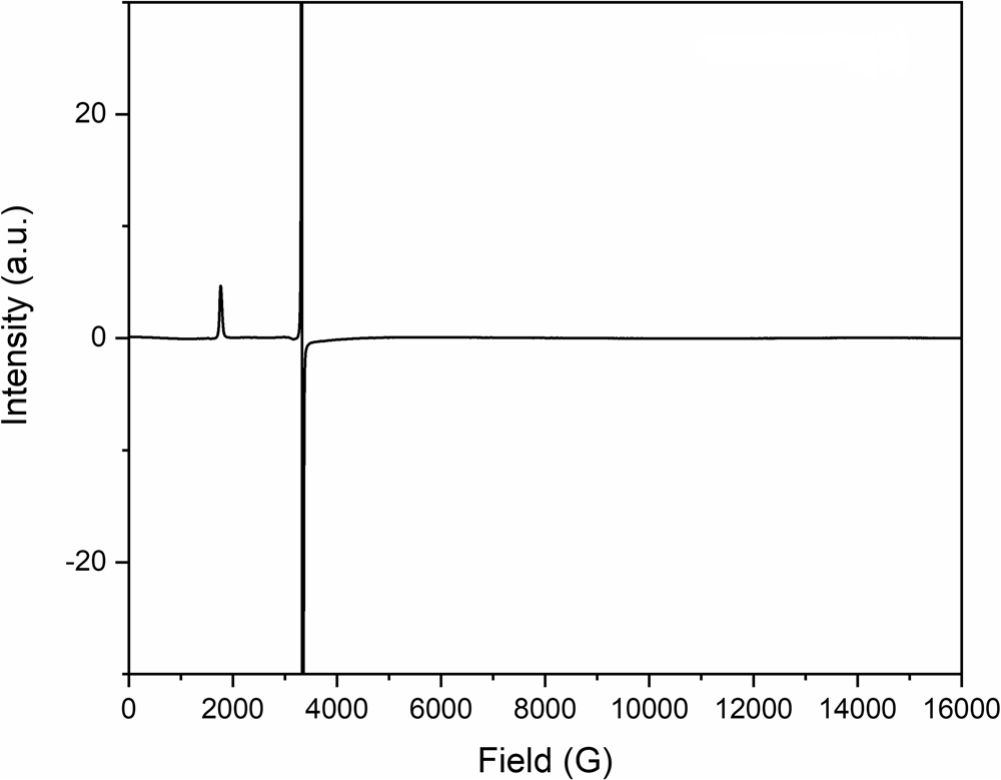
X-band EPR (9.44 GHz) spectrum of solid crude product from the conversion of 2K to 4K and 6K. The signal at *g* = 3.74 corresponds to **4K** and the one at *g* = 2.0023 is characteristic of the benzene radical anion (linewidth 14 Gauss).

Complexes **2M** and **3M** are stable indefinitely in toluene or benzene at room temperature or toluene at 80 °C; only in benzene at elevated temperatures do **4K/Rb/Cs** form. Complexes **4M** are stable in benzene and toluene at room temperature and elevated temperatures (up to 100 °C) for days, and the eventual onset of decomposition results in the formation of Tren^TIPS^H_3_ as the major identifiable product, but **1** is not formed. Since **4K/Rb/Cs** can be obtained by heating **2K/Rb/Cs** as well as in situ prepared samples, incomplete metalation of **1** and thence its presence in the reaction products can be ruled out. Thus, with the identities of **3K/Rb/Cs**-**5K/Rb/Cs** confirmed by the structural, spectroscopic, and magnetic characterisation data, the only plausible explanation for the formation of **4K/Rb/Cs** from **1** is disproportionation of **2K/Rb/Cs** and formation of the uranium(III)-amide complex [U^III^(Tren^TIPS^)(NH_2_)][M] (**6M**, M = K, Rb, Cs), which is subsequently oxidised to **1** by the benzene solvent (see below). In order to probe and confirm this aspect, we examined the disproportionation of crystalline **2K** in detail. As a pink suspension of pure **2K** in benzene is warmed the solution turns red as **2K** dissolves, and after 30 min at 80 °C the solution becomes dark red. As the solution cools, dark red crystals of **4K** grow (30% yield, 50% maximum). ^1^H NMR spectroscopy reveals the presence of **1** in the mother liquor (Supplementary Fig. 36), and after work up **1** was isolated in crystalline form (38% yield, 50% maximum). When **2Rb** or **2Cs** are used instead of **2K**, **4Rb** or **4Cs** along with **1** are similarly isolated. Notably, small quantities of insoluble and distinctly grey precipitates were transiently observed during reactions. The known uranium(III)-amide complex [U^III^(Tren^TIPS^)(NH_2_)][K(benzo-15-crown-5)_2_]^37^ is also distinctively grey and highly insoluble, and, notably, attempts to dissolve it in benzene by warming without exception always results in its oxidation to give solutions exclusively containing **1**, implying reduction of a sacrificial species. It is germane to note that the grey colour of [U^III^(Tren^TIPS^)(NH_2_)][K(benzo-15-crown-5)_2_] is unique for Tren^TIPS^U complexes^38^, with all other derivatives being green, yellow, red, light brown, or dark blue/purple and no metal halides, that can often appear to be grey, are present in these reactions. ATR-IR spectra of crude reaction mixtures (Supplementary Fig. 37) are not inconsistent with the formation of **6K** when compared to an authentic pure sample of [U^III^(Tren^TIPS^)(NH_2_)][K(benzo-15-crown-5)_2_]^37^, but the spectra are not definitive since small variances would be expected, and do manifest, due to the crude-pure comparison. Exhaustive attempts to isolate **6K** from the reaction were unsuccessful, only resulting the isolation of the oxidised product **1**. Although reaction concentrations are inherently low, immediately quenching a reaction mixture that produces **4K** and **6K** by freezing the benzene solution and recording an X-band (9.44 GHz) EPR spectrum reveals a weak signal at *g* = 2.0023 (Supplementary Fig. 38), characteristic of the benzene radical anion (linewidth 14 Gauss)^39^. Allowing the reaction to proceed longer then removing the benzene solvent and recording the X-band EPR spectrum on the solid crude product results in a spectrum with the characteristic absorption at *g* = 3.74 for **4** **K** and also a stronger signal at *g* = 2.0023, Fig. 6. Replacing the benzene solvent with toluene completely blocks the production of **4K** by disproportionation, and only **2K** is isolated when toluene is the reaction solvent. These observations suggest that heating mixtures of **1** with heavier alkali metal alkyls promotes disproportionation of **2M** to **4M** and **6M** (M = K, Rb, Cs). However, the latter when heated, as necessarily per the experimental conditions, spontaneously converts to **1** by reducing the benzene solvent. That this reactivity is not a viable reaction route with toluene is consistent with benzene being easier to reduce than toluene, or viewed from the opposite perspective toluene is more easily oxidised than benzene^40^. The finding that **2K**, **2Rb**, and **2Cs** disproportionate to isolable **4K**, **4Rb**, and **4Cs**, but **2Li** and **2Na** do not seem to produce **4Li** or **4Na**, is consistent with previous EPR spectroscopic observations that potassium, rubidium, and caesium reduce benzene, but lithium and sodium seemingly do not^39^, since the formation of the resulting lithium or sodium benzene radical anion complexes would not be favourable.

### Computational reaction profile analysis

In order to further probe and confirm our hypothesis of uranium(IV) disproportionation to uranium(III) and (V), we modelled reaction profiles computationally at the DFT level (B3PW91) including solvent effects (Supplementary Tables 8–47). Firstly, to confirm the validity of our approach^37,41–44^, we modelled the metallation of **1** to produce **2M** and **3M**, for M = Li, Na, K, Cs, (Supplementary Figs. 39–42) but omitted Rb from these computationally intensive full-structure calculations since it sits in-between K and Cs. Highlighting the formation of **2Cs** and **3Cs** as an exemplar (Supplementary Fig. 42), deprotonation of **1** with benzyl caesium proceeds via an adduct that is 3 kcal/mol lower in energy (**Cs-adduct-in**), and negotiation of a low-lying transition state (**Cs-TS-in**) that is 3.4 kcal/mol higher in energy than **Cs-adduct-in** produces [U^IV^(Tren^TIPS^)(μ-NH)(Cs)] (**Cs-product-in**) that is 14.4 kcal/mol more stable than the starting point. Dimerisation to give **2Cs** increases the energetic stabilisation to 25.2 kcal/mol from the starting point, and addition of Me_6_-Tren finally produces **3Cs** lying 27.5 kcal/mol below the starting point, which is in-line with experimental observations. Adding further reassurance, we note in passing that the final energetic stabilities of **2M** and **3M** are, as expected, ordered Li > Na > K > Cs.

**Fig. 7:**
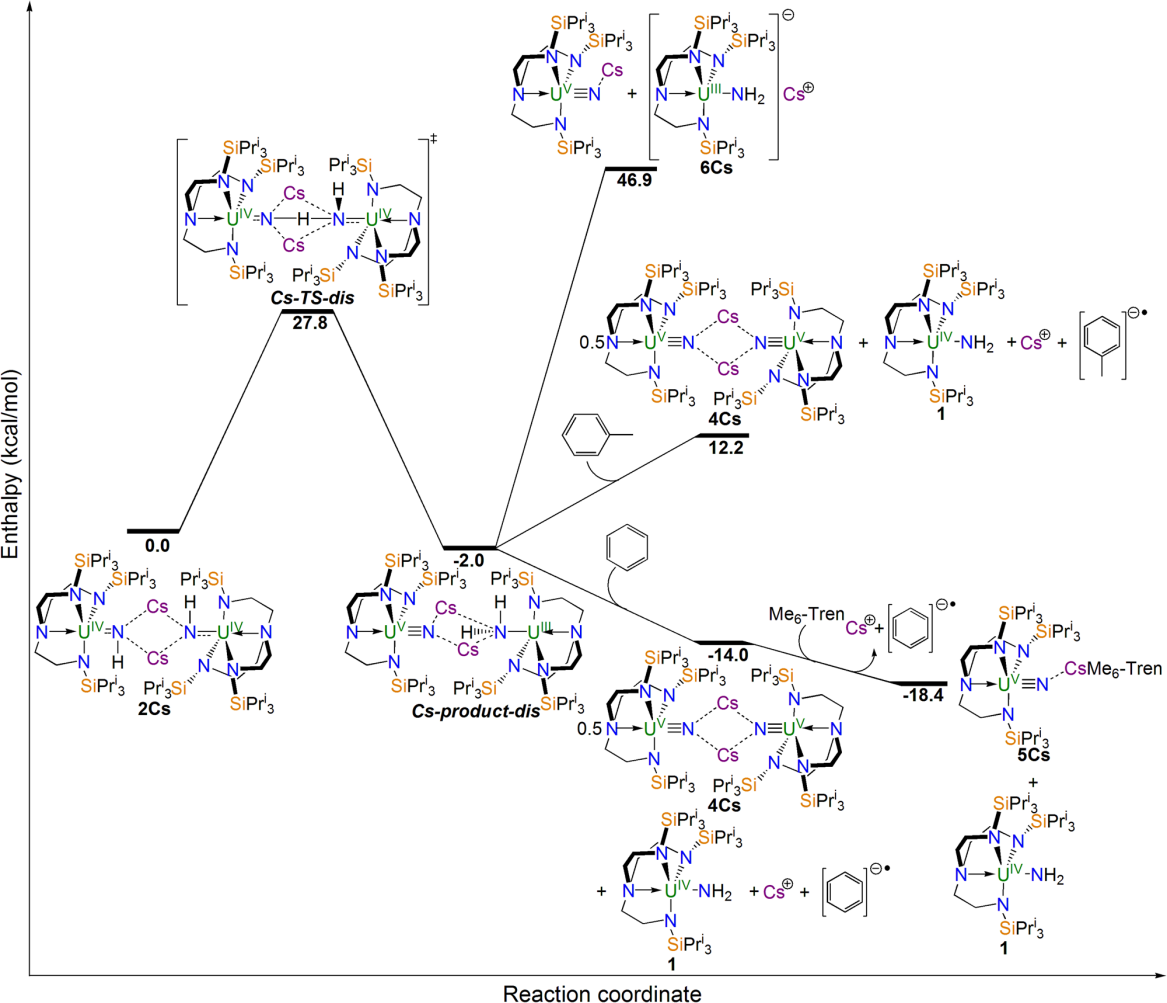
Computed reaction profile for the disproportionation of 2Cs to 4Cs/1/Cs^+^(C_6_H_6_)^−•^. Complex **2Cs** containing two uranium(IV) ions reacts via transition state **Cs-TS-dis** to give the disproportionation intermediate **Cs-product-dis** where uranium(V) and (III) ions are formed. Spontaneous cleavage of **Cs-product-dis** to two monomer units is clearly disfavoured, as is reaction with toluene to give **4Cs**, **1**, and the caesium toluene radical anion salt. **Cs-product-dis** however does react with benzene to give **4Cs**, **1**, and the caesium benzene radical anion salt. Complex **4Cs** is then converted to **5Cs** using the capping agent Me_6_-Tren.

**Fig. 8:**
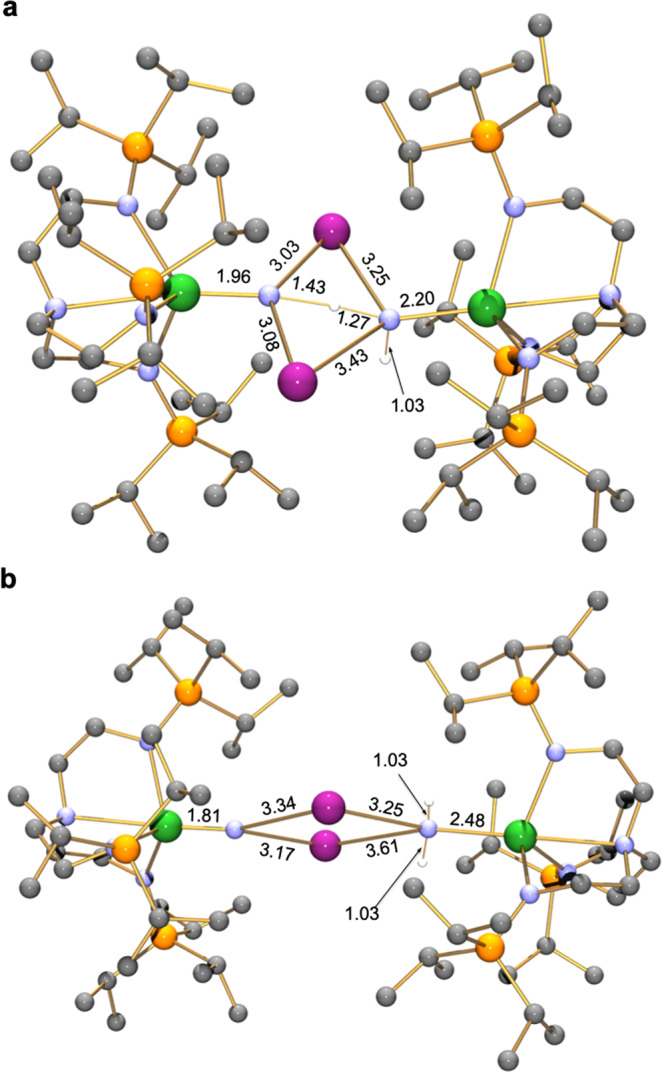
Computed structures, with C–H hydrogen atoms omitted for clarity, of the key transition state and intermediate in the disproportionation of 2Cs to 4Cs/1/Cs^+^(C_6_H_6_)^−•^. **a** The structure of **Cs-TS-dis** with key bond distances (Å). **b** The structure of **Cs-product-dis** with key bond distances (Å). Key: uranium, green; caesium, purple; carbon, grey; hydrogen, white; nitrogen, blue; silicon, orange.

With our reaction profile approach validated, we probed the disproportionation reactions. Again, using the Cs system as a representative example, Fig. 7, starting with **2Cs** set to zero, formation of the dinuclear, disproportionated aggregate [U^V^(Tren^TIPS^)(N)(Cs)_2_(H_2_N)(Tren^TIPS^)U^III^] (**Cs-product-dis**), that is 2 kcal/mol lower in energy than **2Cs**, is realised via a transition state (**Cs-TS-dis**) 27.8 kcal/mol above the starting point, so this intermediate is kinetically and thermodynamically accessible. Both uranium ions in **Cs-TS-dis** are still 5f^2^ uranium(IV) (computed uranium spin densities of 2.23 and 2.18, cf 2.19 for each uranium ion in **2Cs**), and this transition state is clearly an inner-sphere hydrogen atom transfer from one imido nitrogen, an incipient nitride, to the other, a nascent amide, as revealed by inspection of the key bond metrics in **Cs-TS-dis**, Fig. 8. The computed spin densities of the two uranium ions in **Cs-product-dis** are 1.19 and 3.12, which confirms 5f^1^ and 5f^3^ formulations and hence the formation of uranium(V) and (III) ions, respectively. The salient bond distances in **Cs-product-dis**, Fig. 8, confirms that the nitride and amide linkages are now fully formed, which is reflected in the distinctly asymmetric Cs_2_N_2_ four-membered ring of this mixed-valent complex, which contrasts to the inversion symmetric M_2_N_2_ rings of homovalent **2M** and **4M**. The energy barrier of 27.8 kcal/mol is experimentally accessible at 80 °C, accounting for the thermally promoted nature of the disproportionation. Simple dissociation of **Cs-product-dis** into half an equivalent of **4Cs** and **6Cs** is endothermic by 48.9 kcal/mol and so is not energetically feasible nor is it experimentally observed. However, addition of benzene and its reduction by [U^III^(Tren^TIPS^)(NH_2_)][Cs] to give **4M**, **1**, and the caesium salt of the benzene radical anion is energetically favourable, at 12.0 kcal/mol below the **Cs-product-dis**, and the formation of the benzene radical anion is experimentally observed by our EPR studies on the in-situ solution of the reaction. Further, addition of Me_6_-Tren is even more energetically stabilising by a further 4.4 kcal/mol. When benzene solvent is replaced by toluene, the production of **4M**, **1**, and the caesium salt of the toluene radical anion is now disfavoured, being 14.2 kcal/mol above **Cs-product-dis**, which can be related to the fact that benzene is far easier to reduce than toluene; whilst this route is in principle accessible, it is ultimately an endothermic product and so does not occur.

**Fig. 9:**
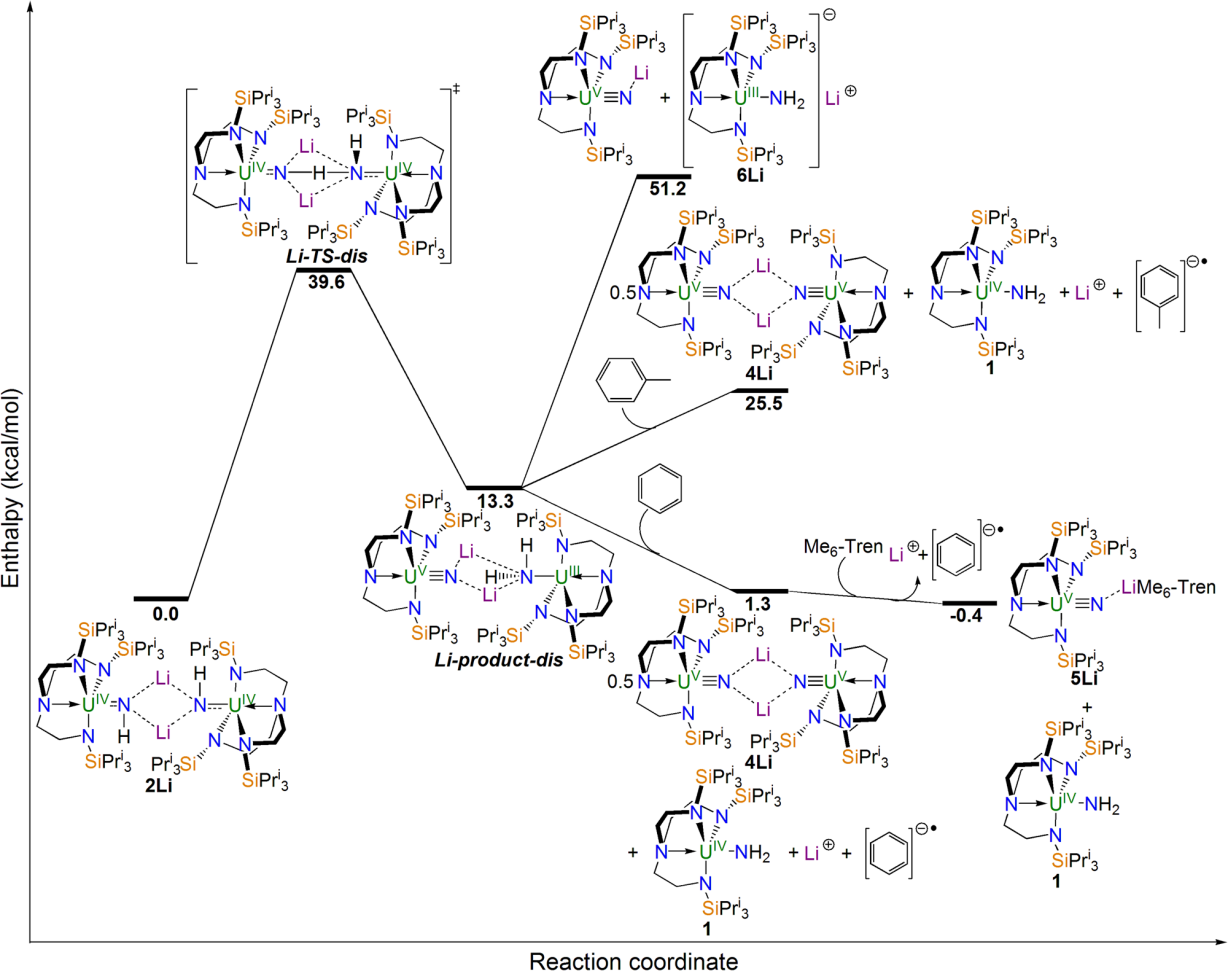
Computed reaction profile for the hypothetical disproportionation of 2Li to 4Li/1/Li^+^(C_6_H_6_)^−•^. Complex **2Li** containing two uranium(IV) ions reacts via transition state **Li-TS-dis** to give the disproportionation intermediate **Li-product-dis** where uranium(V) and (III) ions are formed, but this is an endothermic reaction. Spontaneous cleavage of **Li-product-dis** to two monomer units is clearly disfavoured, as is reaction with toluene to give **4Li**, **1**, and the lithium toluene radical anion salt. If **Li-product-dis** formed it would, however, react with benzene to give **4Li**, **1**, and the lithium toluene radical anion salt. Complex **4Li** would then converted to **5Li** using the capping agent Me_6_-Tren.

To assess whether the experimental observations are independently reproduced computationally, we examined the conversion of **2M** to **4M**+**1**+**M(arene)** for Li, Na, and K, again omitting Rb since it sits in-between K and Cs which both give the same product outcome. We find that, with some variations of exact enthalpies, essentially the same reaction profile and outcome is computed for K (Supplementary Fig. 43) as for Cs, consistent with experiment; Rb would then evidently give the same outcome, as experimentally observed. However, when the corresponding Li and Na profiles are computed, Fig. 9 and Supplementary Information Fig. 44, we find that whilst the final, hypothetical products **5Li**/**Na** reside at slightly lower energies than the starting points (−0.4 and −2.9 kcal/mol), the preceding **4Li**/**4Na**+**1**+**M(arene)** products are slightly positive with respect to the starting points (1.3 and 0.9 kcal/mol). As expected, for Li and Na, spontaneous cleavage of **M-product-dis** for Li and Na are both very unfavourable (>40 kcal/mol), underscoring the importance of the solvent in these reactions, and the toluene routes are also very unfavourable (>24 kcal/mol) compared to the starting points. All those reaction outcomes are therefore thermodynamically endothermic overall and so do not occur. Importantly, the **Li-product-dis** and **Na-product-dis** compounds are endothermic products compared to their respective starting points (13.3 and 4.5 kcal/mol) and the energy barriers are now >31 kcal/mol (cf the K and Cs congeners are <28 kcal/mol) so these intermediates are kinetically and thermodynamically disfavoured.

The reaction profile calculations thus independently reproduce and verify the experimental hypothesis, confirming that disproportionation of uranium(IV) to uranium(III) and (V) has occurred, and that the solvent blocks or facilitates the reactivity, in the latter case also accounting for the oxidation of the uranium(III) component to uranium(IV). The regeneration of **1** from this disproportionation reaction proposed computationally also nicely independently corroborates the experimental observation that addition of an increased excess of potassium alkyl to **1** produces increased yields of **4K**, because for each disproportionation event, the uranium(III) component is oxidised to **1** which under the action of further deprotonating agent in a thermolytic regime disproportionates again, generating more **4K**.

## Discussion

The reactivity disclosed here provides a complementary example of uranium(IV) disproportionation to the other example in the literature^14^. The prior report involved a diuranium(IV)-nitride complex disproportionating under the action of methyltriflate to a uranium(III)-triflate and uranium(V)-imido mixture^14^, whereas here we report a diuranium(IV)-imido disproportionating to uranium(V)-nitride and uranium(III)-amide under the action of solvent and temperature. Nitrides are typically regarded as being difficult linkages to access in the coordination sphere of actinides, so a reaction that produces a nitride, rather than a nitride reacting to give something else, is unusual. The results reported here now shed light on the factors that can induce uranium(IV) to disproportionate in as much as clearly the ligands play a key role, but the experimental and computational results both also point to the decisive role that the solvent plays in facilitating, or not, the uranium(IV) disproportionation. This constitutes an unusual level of modification of a fundamental thermodynamic property of uranium to promote a redox reaction, rather than just effecting kinetic control or blocking, in three examples by a common ligand class (imido) and widely used solvent (benzene) in combination. These external factors have shifted a fundamental redox phenomenon from unfavourable to favourable under relatively mild conditions, demonstrating a dependence on external drivers that is more reminiscent of transition metal chemistry rather than lanthanide and actinide chemistry. The establishment of two types of conceptually opposite (nitride to imido and imido to nitride) uranium(IV) disproportionation suggests the potential for elaboration, and indeed it may be a more widespread reaction pathway under more extreme conditions than is currently recognised, for example in highly radiolytic scenarios. For example, the inner-sphere proton migration during the uranium(IV) disproportionation reported here is reminiscent of proton-promoted disproportionation of di-actinyl(V) cation–cation complexes^45–50^ that drives actinide ion mobility and complex, problematic equilibria in nuclear fuel extraction processes, e.g. PUREX^16^, and bio-mediated deposition of uranium(IV)-oxides from uranyl(VI) in the environment^10,11^. Tellingly, the disproportionation of uranium(IV) reported here involves H_2_N^−^, HN^2−^, and N^3−^ ligands, which parallels the prevalence of isoelectronic and isolobal H_2_O, HO^−^, and O^2−^ ligands in the disproportionation chemistry of uranium(V) in cation–cation complexes^16,45^, so it would seem that the softer nitrogen-based ligands facilitate disproportionation with the lower oxidation state of uranium and analogously the harder oxygen-based ligands with the higher oxidation state of uranium. There are many uranium(V)-oxo complexes, including uranyl(V), and so H^+^-promoted disproportion of U^V^=O linkages is well-precedented. However, there are few U^IV^=NH linkages^17,51^, and so disproportionation of U^IV^=NH linkages could in principle be expanded with other ancillary ligands to Tren^TIPS^. Thus, the development of the third redox disproportionation reaction for uranium hints at the potential to have implications for the chemistry of uranium in a range of scientific, technological, radiochemical, and environmental scenarios.

## Methods

### General

All manipulations were carried out under an inert atmosphere of dry nitrogen using Schlenk techniques, or an MBraun UniLab glovebox operating under an atmosphere of dry nitrogen. THF, toluene and pentane solvents were dried by passage through activated alumina towers and degassed before use. Hexanes and benzene were distilled from potassium. All solvents were stored over potassium mirrors except for ethers which were stored over activated 4 Å sieves. Deuterated solvents were distilled from potassium, degassed by three freeze-pump-thaw cycles and stored under nitrogen prior to use. Bu^t^Li (1.0 M in pentane), and N(CH_2_CH_2_NMe_2_)_3_ were used as purchased. MCH_2_Ph (M = Na, K, Rb, Cs), [U(Tren^TIPS^)(NH_2_)] (**1**), [{U(Tren^TIPS^)(μ-NH)(μ-M)}_2_] (**2M**, M = Li, Na, K, Rb, Cs), and [{U(Tren^TIPS^)(μ-N)(μ-M)}_2_] (**4M**, M = Li, Na, K, Rb, Cs) were prepared using literature methods ^17–21,52^.

Single crystals were examined variously on either (a) an Oxford Diffraction SuperNova Atlas CCD diffractometer using mirror-monochromated MoKα radiation (λ = 0.71073 Å), (b) a Rigaku Xcalibur2 diffractometer equipped with an Atlas CCD area detector and a sealed tube source with graphite-monochromated MoKα radiation (λ = 0.71073 Å), or (c) a Rigaku FR-X diffractometer equipped with a Hypix 6HE photon counting pixel array detector with mirror-monochromated MoKα (λ = 0.71073 Å) or CuKα (λ = 1.5418 Å) radiation. Intensities were integrated from a sphere of data recorded on narrow (1.0°) frames by ω rotation. Cell parameters were refined from the observed positions of all strong reflections in each data set. Gaussian grid face-indexed absorption corrections with a beam profile correction were applied. The structures were solved either by dual methods using SHELXT^53^ and all non-hydrogen atoms were refined by full-matrix least-squares on all unique *F*^2^ values with anisotropic displacement parameters with exceptions noted in the respective cif files. Hydrogen atoms were refined with constrained geometries and riding thermal parameters; *U*_iso_(H) was set at 1.2 (1.5 for methyl groups) times *U*_eq_ of the parent atom. The largest features in final difference syntheses were close to heavy atoms and were of no chemical significance. CrysAlisPro was used for control and integration^54^, and SHELXL and Olex2 were employed for structure refinement^55,56^. ORTEP-3 and POV-Ray were employed for molecular graphics^57,58^. ^1^H, ^29^Si{^1^H}, and ^7^Li{^1^H} NMR spectra were recorded on a Bruker 400 spectrometer operating at 400, 79, 155 MHz, respectively; chemical shifts are quoted in ppm and are relative to TMS (^1^H, ^29^Si) and LiCl (^7^Li). ATR-IR spectra were recorded on a Bruker Alpha spectrometer with a Platinum-ATR module in the glovebox. UV-Vis-NIR spectra were recorded on a Perkin Elmer LMD^TM^ 750 spectrometer. Data were collected in a 1 mm path-length cuvette loaded in an MBraun glovebox and were run versus the appropriate solvent. EPR spectra were measured using a X-band (9.44 GHz) Bruker Elexsys E500 spectrometer equipped with an ER4118SMS5 resonator at 5 K. Solution samples were transferred to the quartz tubes under argon, and the tubes were flame sealed prior to experiments. Field corrections were applied to the raw data using Bruker strong pitch (*g* = 2.0026) as a reference. Static variable-temperature magnetic moment data were recorded in an applied dc field of 0.5 T on by Quantum Design MPMS XL7 or MPMS 3 (for **3Li** and **5Na**) SQUID magnetometers using recrystallised powdered samples. Care was taken to ensure complete thermalisation of the sample before each data point was measured and samples were immobilised in an eicosane matrix to prevent sample reorientation during measurements. Diamagnetic corrections were applied for using tabulated Pascal constants and measurements were corrected for the effect of the blank sample holders (flame sealed Wilmad NMR tube and straw) and eicosane matrix. CHN microanalyses were carried out by Martin Jennings and Anne Davies at the University of Manchester.

### Preparation of [U(Tren^TIPS^)(μ-NH)(μ-Li)(Me_6_-Tren)] (3Li)

Method A: A solution of Me_6_-Tren (0.17 g, 0.75 mmol) in hexanes (5 ml) was added slowly to a pre-cooled (−78 °C) solution of Bu^t^Li (0.035 g, 0.55 mmol) in hexanes (10 ml). After 10 min, a solution of **1** (0.43 g, 0.50 mmol) in hexanes (10 ml) was added at −78 °C. The mixture was allowed to warm to room temperature and stirred for further 16 h, forming a red solution. All the volatiles were removed in vacuo to afford a pink solid, which was extracted in pentane (5 ml) and filtered. Pink crystals of **3Li** were obtained by storing the resulting red solution at −30 °C for 2 days. Yield: 0.21 g, 38%. Method B: Me_6_-Tren (0.17 g, 0.75 mmol) was added to a pale pink suspension of **2Li** (0.44 g, 0.25 mmol) in toluene (20 ml) and the reaction was stirred for 24 h at room temperature. Volatiles were removed from the resulting red solution in vacuo. The resulting solid was extracted into pentane (5 ml) and filtered; the red filtrate was stored at −30 °C for 16 h to yield pink crystals of **3Li** which were isolated by decanting the mother liquor and dried in vacuo. Yield: 0.36 g, 65%. Anal. Calcd for C_45_H_106_LiN_9_Si_3_U: C, 49.02; H, 9.69; N, 11.43%. Found: C, 48.77; H, 9.99; N, 11.37%. ^1^H NMR (400 MHz, C_6_D_6_, 298 K): δ −24.03 (br, 54H, CH(C*H*_3_)_2_), −23.72 (br, 9H, C*H*(CH_3_)_2_), 13.71 (br, 6H, NC*H*_2_CH_2_), 18.39 (br, 18H, N(C*H*_3_)_2_), 25.04 (br, 6H, C*H*_2_-Me_6_-TREN), 37.50 (br, 6H, C*H*_2_-Me_6_-TREN), 105.44 (br, 6H, NCH_2_C*H*_2_). The NH resonance was not observed in the ^1^H NMR spectrum. ^7^Li{^1^H} NMR (155 MHz, 298 K, C_6_D_6_): δ 2.03 (br). ^29^Si{^1^H} NMR (79 MHz, C_6_D_6_, 298 K): δ −155.20 (br). ATR-IR *v*/cm^−^^1^: 2935 (br, w), 2857 (m), 2823 (w), 1457 (m), 1355 (w), 1273 (w), 1115 (w), 1061 (m), 1012 (w), 931 (s), 881 (s), 733 (vs), 670 (s), 624 (m), 561 (w), 510 (w), 441 (w). The NH stretching absorption was not observed. μ_eff_ (Evan’s method, C_6_D_6_, 298 K): 2.3 μ_B_.

### Preparation of [U(Tren^TIPS^)(μ-NH)(μ-Na)(Me_6_-Tren)] (3Na)

Method A: A solution of Me_6_-Tren (0.17 g, 0.75 mmol) in toluene (5 ml) was added slowly to a pre-cooled (−78 °C) mixture of NaCH_2_Ph (0.063 g, 0.55 mmol) in toluene (10 ml). After 30 min, a solution of **1** (0.43 g, 0.50 mmol) in toluene (10 ml) was added at −78 °C. The mixture was allowed to warm to room temperature and stirred for further 16 h, forming a red solution. The reaction was filtered and concentrated to 5 ml. Pink crystals of **3Na** were obtained by storing the resulting red solution at −30 °C for a few days. Yield: 0.26 g, 47%. Method B: Me_6_-Tren (0.17 g, 0.75 mmol) was added to a pink suspension of **2Na** (0.45 g, 0.25 mmol) in toluene (20 ml) and the reaction was stirred for 24 h at room temperature. Volatiles were removed from the resulting red solution in vacuo. The resulting solid was washed with pentane (2 × 5 ml) and dried in vacuo, giving **3Na** as a pink solid. Yield: 0.42 g, 76%. Anal. Calcd for C_45_H_106_NaN_9_Si_3_U: C, 48.32; H, 9.55; N, 11.27%. Found: C, 48.04; H, 9.67; N, 10.86%. ^1^H NMR (400 MHz, C_6_D_6_, 298 K): δ −24.88 (br, 54H, CH(C*H*_3_)_2_), −22.56 (br, 9H, C*H*(CH_3_)_2_), 14.42 (br, 6H, NC*H*_2_CH_2_), 22.29–24.13 (br, 30H, C*H*_2_C*H*_2_N(C*H*_3_)_2_), 107.21 (br, 6H, NCH_2_C*H*_2_). The NH resonance was not observed in the ^1^H NMR spectrum. Reliable ^29^Si NMR and UV/Vis/NIR spectra and solution magnetic moment (Evan’s method) could not be obtained, due to **3Na** being only partially soluble in aromatic solvent (benzene and toluene) once isolated, and it decomposes in polar solvents. ATR-IR *v*/cm^−1^: 2936 (br, w), 2858 (m), 2825 (w), 1461 (m), 1357 (w), 1273 (w), 1116 (w), 1063 (m), 1010 (w), 934 (s), 880 (s), 735 (vs), 671 (s), 624 (m), 561 (w), 509 (w), 440 (w). The NH stretching absorption was not observed.

### Preparation of [U(Tren^TIPS^)(μ-NH)(μ-K)(Me_6_-Tren)] (3K)

Method A: A solution of Me_6_-Tren (0.17 g, 0.75 mmol) in toluene (5 ml) was added slowly to a pre-cooled (−78 °C) mixture of KCH_2_Ph (0.072 g, 0.55 mmol) in toluene (10 ml). After 30 min, a solution of **1** (0.43 g, 0.50 mmol) in toluene (10 ml) was added at −78 °C. The mixture was allowed to warm to room temperature and stirred for further 16 h, forming a red solution. The mixture was filtered and concentrated to 5 ml. Pink crystals of **3K** were obtained by storing the resulting red solution at −30 °C for 2 days. Yield: 0.25 g, 43%. Method B*:* Me_6_-Tren (0.17 g, 0.75 mmol) was added to a pink suspension of **2K** (0.45 g, 0.25 mmol) in toluene (20 ml) and the reaction was stirred for 24 h at room temperature. Volatiles were removed from the resulting red solution in vacuo. The product was washed with pentane (2 × 5 ml) and dried in vacuo, giving **3K** as a pink solid. Yield: 0.36 g, 63%. Anal. Calcd for C_45_H_106_KN_9_Si_3_U: C, 47.63; H, 9.42; N, 11.11%. Found: C, 47.52; H, 9.54; N, 10.65%. ^1^H NMR (400 MHz, C_6_D_6_, 298 K): δ −23.74 (br, 54H, CH(C*H*_3_)_2_), −20.52 (br, 9H, C*H*(CH_3_)_2_), 15.78 (br, 6H, NC*H*_2_CH_2_), 22.42 (br, 30H, C*H*_2_C*H*_2_N(C*H*_3_)_2_), 106.55 (br, 6H, NCH_2_C*H*_2_). The NH resonance was not observed in the ^1^H NMR spectrum. Reliable ^29^Si NMR and UV/Vis/NIR spectra and solution magnetic moment (Evan’s method) could not be obtained, due to **3K** being only partially soluble in aromatic solvent once isolated, and it decomposes in polar solvents. ATR-IR *v*/cm^−1^: 2935 (br, w), 2857 (m), 2828 (w), 1460 (m), 1357 (w), 1272 (w), 1116 (w), 1066 (m), 1008 (w), 933 (s), 880 (s), 736 (vs), 670 (s), 626 (m), 558 (w), 507 (w), 442 (w). The NH stretching absorption was not observed.

### Preparation of [U(Tren^TIPS^)(μ-NH)(μ-Rb)(Me_6_-Tren)] (3Rb)

Method A: A solution of Me_6_-Tren (0.17 g, 0.75 mmol) in toluene (5 ml) was added slowly to a pre-cooled (−78 °C) mixture of RbCH_2_Ph (0.097 g, 0.55 mmol) in toluene (10 ml). After 30 min, a solution of **1** (0.43 g, 0.50 mmol) in toluene (10 ml) was added at −78 °C. The mixture was allowed to warm to room temperature and stirred for further 16 h, forming a red solution. The mixture was filtered and concentrated to 5 ml. Pink crystals of **3Rb** were obtained by storing the resulting red solution at −30 °C for 2 days. Yield: 0.33 g, 56%. Method B: Me_6_-Tren (0.17 g, 0.75 mmol) was added to the pink suspension of **2Rb** (0.48 g, 0.25 mmol) in toluene (20 ml) and the mixture was stirred for 24 h at room temperature. Volatiles were removed from the resulting red solution in vacuo. The pink residue was washed with pentane (2 × 5 mL) and dried in vacuo, giving **3Rb** as a pure pink solid. Yield: 0.40 g, 68%. Anal. Calcd for C_45_H_106_RbN_9_Si_3_U: C, 45.76; H, 9.05; N, 10.67%. Found: C, 45.71; H, 9.29; N, 10.54%. ^1^H NMR (400 MHz, C_6_D_6_, 298 K): δ −23.24 (br, 54H, CH(C*H*_3_)_2_), −21.08 (br, 9H, C*H*(CH_3_)_2_), 8.54 (br, 12H, C*H*_2_-Me_6_-TREN), 11.03 (br, 18H, N(C*H*_3_)_2_), 16.54 (br, 6H, NC*H*_2_CH_2_), 106.59 (br, 6H, NCH_2_C*H*_2_). The NH resonance was not observed in the ^1^H NMR spectrum. Reliable ^29^Si NMR and UV/Vis/NIR spectra and solution magnetic moment (Evan’s method) could not be obtained, due to **3Rb** being only partially soluble in aromatic solvent once isolated, and it decomposes in polar solvents. ATR-IR *v*/cm^−1^: 2934 (br, w), 2880 (m), 2821 (w), 1460 (m), 1339 (w), 1274 (w), 1117 (w), 1070 (m), 1010 (w), 931 (s), 881 (s), 736 (vs), 670 (s), 624 (m), 539 (w), 508 (w), 440 (w). The NH stretching absorption was not observed.

### Preparation of [U(Tren^TIPS^)(μ-NH)(μ-Cs)(Me_6_-Tren)] (3Cs)

Method A: A solution of Me_6_-Tren (0.17 g, 0.75 mmol) in toluene (5 ml) was added slowly to a pre-cooled (−78 °C) mixture of CsCH_2_Ph (0.12 g, 0.55 mmol) in toluene (10 ml). After 30 min, a solution of **1** (0.43 g, 0.50 mmol) in toluene (10 ml) was added at −78 °C. The mixture was allowed to warm up to room temperature and stirred for further 16 h, forming a dark red brown solution. The mixture was filtered and concentrated to 3 ml. Pink crystals of **3Cs** were obtained by storing the resulting dark red brown solution in at −30 °C for 2 days. Yield: 0.22 g, 35%. Method B: Me_6_-Tren (0.17 g, 0.75 mmol) was added to a pink suspension of **2Cs** (0.50 g, 0.25 mmol) in toluene (20 ml) and the reaction was stirred for 24 h at room temperature. Volatiles were removed from the resulting dark red brown solution in vacuo. The mixture was washed with pentane (2 × 5 mL) and dried in vacuo, giving **3Cs** as a pure pink solid. Yield: 0.38 g, 60%. Anal. Calcd for C_45_H_106_CsN_9_Si_3_U: C, 43.99; H, 8.70; N, 10.26%. Found: C, 43.75; H, 8.89; N, 9.78%. ^1^H NMR (400 MHz, C_6_D_6_, 298 K): δ −23.06 (br, 54H, CH(C*H*_3_)_2_), −22.03 (br, 9H, C*H*(CH_3_)_2_), 8.53 (br, 6H, C*H*_2_-Me_6_-TREN), 8.61 (br, 6H, C*H*_2_-Me_6_-TREN), 10.83 (br, 18H, N(C*H*_3_)_2_), 16.87 (br, 6H, NCH_2_C*H*_2_), 108.04 (br, 6H, NCH_2_C*H*_2_). The NH resonance was not observed in the ^1^H NMR spectrum. ^29^Si{^1^H} NMR (79 MHz, C_6_D_6_, 298 K): δ −166.29 (br). ATR-IR *v*/cm^−1^: 2937 (br, w), 2856 (m), 2823 (w), 1459 (m), 1357 (w), 1272 (w), 1118 (w), 1070 (m), 1010 (w), 932 (s), 880 (s), 733 (vs), 670 (s), 626 (m), 560 (w), 505 (w), 441 (w). The NH stretching absorption was not observed. μ_eff_ (Evans method, C_6_D_6_, 298 K): 2.9 μ_B_.

### Preparation of [U(Tren^TIPS^)(μ-N)(μ-Li)(Me_6_-Tren)] (5Li)

Me_6_-Tren (0.17 g, 0.75 mmol) was added to a red suspension of **4Li** (0.44 g, 0.25 mmol) in toluene (20 ml) and the mixture was stirred for 24 h at room temperature. Volatiles were removed from the resulting red solution in vacuo. The mixture was extracted into pentane (5 ml), filtered, and the dark red filtrate was stored at −30 °C for 2 days to yield dark red crystals of **5Li**, which was isolated by decanting the mother liquor and dried in vacuo. Yield: 0.38 g, 68%. Anal. Calcd for C_45_H_105_LiN_9_Si_3_U: C, 49.06; H, 9.61; N, 11.44%. Found: C, 49.13; H, 10.13; N, 11.23%. ^1^H NMR (400 MHz, C_6_D_6_, 298 K): δ −26.27 (s, 9H, C*H*(CH_3_)_2_), −8.01 (s, 54H, CH(C*H*_3_)_2_), 7.50 (br, 6H, NC*H*_2_CH_2_), 9.24 (br, 18H, N(C*H*_3_)_2_), 9.95 (br, 6H, C*H*_2_-Me_6_-TREN), 13.55 (br, 6H, C*H*_2_-Me_6_-TREN), 41.70 (br, 6H, NCH_2_C*H*_2_). ^7^Li{^1^H} (155 MHz, 298 K, C_6_D_6_): δ not observed. ^29^Si{^1^H} NMR (79 MHz, C_6_D_6_, 298 K): δ −15.38. FTIR *v*/cm^−1^: 2936 (br, w), 2856 (m), 2831 (w), 1456 (m), 1354 (w), 1293 (w), 1272 (w), 1114 (w), 1058 (m), 1011 (w), 930 (s), 880 (s), 782 (w), 728 (vs), 669 (s), 625 (m), 564 (w), 510 (w), 444 (w). μ_eff_ (Evan’s method, C_6_D_6_, 298 K): 1.6 μ_B_.

### Preparation of [U(Tren^TIPS^)(μ-N)(μ-Na)(Me_6_-Tren)] (5Na)

Me_6_-Tren (0.17 g, 0.75 mmol) was added to a red suspension of **4Na** (0.45 g, 0.25 mmol) in toluene (20 ml) and the reaction was stirred for 24 h at room temperature. Volatiles were removed from the resulting red solution in vacuo. The product was extracted into hot toluene (80 °C, 10 ml), filtered, and the dark red filtrate was stored at −30 °C for 16 h to yield dark red crystals of **5Na** which was isolated by decanting the mother liquor and dried in vacuo. Yield: 0.23 g, 41%. Anal. Calcd for C_45_H_105_NaN_9_Si_3_U: C, 48.36; H, 9.47; N, 11.28%. Found: C, 48.31; H, 9.79; N, 10.51%. ^1^H NMR (400 MHz, C_6_D_6_, 298 K): δ −25.05 (s, 9H, C*H*(CH_3_)_2_), −7.69 (s, 54H, CH(C*H*_3_)_2_), 7.89 (br, 6H, NC*H*_2_CH_2_), 8.83 (br, 6H, C*H*_2_-Me_6_-TREN), 9.60 (br, 18H, N(C*H*_3_)_2_), 9.95 (br, 6H, C*H*_2_-Me_6_-TREN), 41.21 (br, 6H, NCH_2_C*H*_2_). ATR-IR *v*/cm^−1^: 2935 (br, w), 2857 (m), 2826 (w), 1458 (m), 1356 (w), 1300 (w), 1274 (w), 1224 (w), 1161 (w), 1116 (m), 1026 (w), 934 (s), 878 (s), 783 (w), 732 (vs), 670 (s), 627 (m), 563 (w), 510 (w), 444 (w). Reliable ^29^Si NMR and UV/Vis/NIR spectra and solution magnetic moment (Evan’s method) could not be obtained as **5Na** is only partially soluble in aromatic solvent once isolated, and it decomposes in polar solvents.

### Preparation of [U(Tren^TIPS^)(μ-N)(μ-K)(Me_6_-Tren)] (5K)

Benzene (60 ml) was added slowly to a pre-cooled (−78 °C) mixture of **1** (0.87 g, 1.00 mmol) and KCH_2_Ph (0.13 g, 1.00 mmol). The frozen mixture was allowed to thaw to room temperature and stirred for 24 h, resulting in a pink suspension. The mixture was assayed by ^1^H NMR spectroscopy, confirming that **1** was completely consumed and converted to **2K**. The mixture was then heated to 80 °C for 30 min, and the mixture turned dark red. Dark red crystals of **4K** started to form upon cooling the mother liquor, which was left to further crystallise at 10 °C for 16 h. The identity of **4K** was confirmed by crystallographic unit cell check. Yield: 0.27 g, 30% (based on uranium). Removal of the volatiles from the mother liquor in vacuo gave a pale brown solid containing **1** as the main product by ^1^H NMR spectroscopy. Pentane (2 ml) was added to the brown residue, and after filtration, a brown solution was obtained. Storing the solution at −30 °C for 24 h gave brown crystals **1**, which were isolated by filtration and dried in vacuo. Yield: 0.41 g, 47% (based on uranium). If the ratio of KCH_2_Ph is increased (by 1.5, 2.0, or even 3.0) in the first step, **4K** can be isolated in higher yield (52%, based on uranium), accompanied by a lower yield of **1** (28%, based on uranium) isolated from the mother liquor. The crystalline **4K** was slurried in benzene (15 ml), and Me_6_-Tren (0.10 g, 0.45 mmol) was added. The mixture was stirred for 24 h at room temperature. The red solution was concentrated to 5 ml and filtered. Slow evaporation of the red solution in a glove box afforded dark red crystals of **5K** which were isolated by filtration and dried in vacuo. Yield: 0.21 g, 62%. Utilising authentic **4K** produced **5K** with an identical result. Anal. Calcd for C_45_H_105_KN_9_Si_3_U: C, 47.67; H, 9.34; N, 11.12%. Found: C, 47.44; H, 9.69; N, 10.92%. ^1^H NMR (400 MHz, C_6_D_6_, 298 K): δ −23.81 (s, 9H, C*H*(CH_3_)_2_), −6.92 (s, 54H, CH(C*H*_3_)_2_), 6.85 (br, 30H, C*H*_2_C*H*_2_N(C*H*_3_)_2_), 8.80 (br, 6H, NCH_2_CH_2_), 40.47 (br, 6H, NCH_2_C*H*_2_). FTIR *v*/cm^−1^: 2934 (br, w), 2855 (m), 2824 (w), 1460 (m), 1356 (w), 1310 (w), 1271 (w), 1251 (w), 1157 (w), 1068 (m), 1009 (w), 931 (s), 879 (s), 786 (w), 730 (vs), 668 (s), 626 (m), 561 (w), 507 (w), 443 (w). Reliable ^29^Si NMR and UV/Vis/NIR spectra and solution magnetic moment (Evan’s method) could not be obtained as **5K** is only partially soluble in aromatic solvent once isolated, and it decomposes in polar solvents.

### Preparation of [U(Tren^TIPS^)(μ-N)(μ-Rb)(Me_6_-Tren)] (5Rb)

Benzene (60 ml) was added slowly to a pre-cooled (−78 °C) mixture of **1** (0.87 g, 1.00 mmol) and RbCH_2_Ph (0.18 g, 1.00 mmol). The frozen mixture was left to thaw to room temperature and stirred for 24 h, resulting in a pink suspension. The mixture was then heated to 80 °C for 30 min, and the resulting red suspension was filtered to give a dark red solution. Dark red crystals of **4Rb** started to form upon cooling the mother liquor, which was left to further crystallise at 10 °C for 16 h. The identity of **4Rb** was confirmed by crystallographic unit cell check. Yield: 0.22 g, 24% (based on uranium). The crystalline **4Rb** was slurried in benzene (15 ml), and Me_6_-Tren (0.08 g, 0.36 mmol) was added. The mixture was stirred for 24 h at room temperature. The red solution was concentrated to 5 ml and filtered. Slow evaporation of the red solution in a glovebox afforded dark red crystals of **5Rb** which were isolated by filtration and dried in vacuo. Yield: 0.22 g, 76%. Utilising authentic **4Rb** produced **5Rb** with an identical result. Anal. Calcd for C_45_H_105_RbN_9_Si_3_U: C, 45.80; H, 8.97; N, 10.68%. Found: C, 45.94; H, 9.30; N, 10.51%. ^1^H NMR (400 MHz, C_6_D_6_, 298 K): δ −23.08 (s, 9H, C*H*(CH_3_)_2_), −6.46 (s, 54H, CH(C*H*_3_)_2_), 6.28 (br, 6H, C*H*_2_-Me_6_-TREN), 6.45 (br, 6H, C*H*_2_-Me_6_-TREN), 8.04 (br, 24H, NC*H*_2_CH_2_ and N(C*H*_3_)_2_), 39.27 (br, 6H, NCH_2_C*H*_2_). FTIR *v*/cm^−1^: 2934 (br, w), 2855 (m), 2824 (w), 1460 (m), 1357 (w), 1301 (w), 1156 (w), 1116 (w), 1069 (m), 1009 (w), 931 (s), 879 (s), 787 (w), 731 (vs), 668 (s), 625 (m), 561 (w), 506 (w), 443 (w). Reliable ^29^Si NMR and UV/Vis/NIR spectra and solution magnetic moment (Evan’s method) could not be obtained as **5Rb** is only partially soluble in aromatic solvent once isolated, and it decomposes in coordinative or polar solvents.

### Preparation of [U(Tren^TIPS^)(μ-N)(μ-Cs)(Me_6_-Tren)] (5Cs)

Benzene (30 ml) was added slowly to a pre-cooled (−78 °C) mixture of **1** (0.87 g, 1.00 mmol) and CsCH_2_Ph (0.34 g, 1.5 mmol). The frozen mixture was left to thaw to room temperature and stirred for 24 h, resulting in a pink suspension. The mixture was then heated to 80 °C for 30 min, and the resulting red suspension was filtered to give a red solution. Dark red crystals of **4Cs** started to form upon cooling the mother liquor, which was left to further crystallise at 10 °C for 16 h. The identity of **4Cs** was confirmed by crystallographic unit cell check. Yield: 0.18 g, 18% (based on uranium). The crystalline **4Cs** was slurried in benzene (15 ml), and Me_6_-Tren (0.06 g, 0.27 mmol) was added. The mixture was stirred for 24 h at room temperature. Volatiles were removed from the resulting red brown solution in vacuo. The red brown residue was washed with pentane (5 ml), and then extracted into toluene (5 ml), filtered, concentrated to 2 ml, and then stored at −30 °C for 16 h to yield dark red crystals of **5Cs** which were isolated by filtration and dried in vacuo. Yield: 0.10 g, 46%. Utilising authentic **4Cs** produced **5Cs** with an identical result. Anal. Calcd for C_45_H_105_CsN_9_Si_3_U: C, 44.03; H, 8.62; N, 10.27%. Found: C, 43.85; H, 8.92; N, 10.08%. ^1^H NMR (400 MHz, C_6_D_6_, 298 K): δ −22.57 (br, d, 9H, C*H*(CH_3_)_2_), −5.98 (s, 54H, CH(C*H*_3_)_2_), 4.03 (br, 6H, C*H*_2_-Me_6_-TREN), 4.20 (br, 6H, C*H*_2_-Me_6_-TREN), 4.45 (br, 18H, N(C*H*_3_)_2_), 8.14 (br, 6H, NC*H*_2_CH_2_), 37.45 (br, 6H, NCH_2_C*H*_2_). ^29^Si{^1^H} NMR (79 MHz, C_6_D_6_, 298 K): δ −17.00. FTIR *v*/cm^−1^: 2935 (br, w), 2880 (m), 2822 (w), 1460 (m), 1357 (w), 1303 (w), 1155 (w), 1118 (w), 1071 (m), 1010 (w), 930 (s), 880 (s), 786 (w), 729 (vs), 667 (m), 626 (m), 561 (w), 506 (w), 443 (w). μ_eff_ (Evan’s method, C_6_D_6_, 298 K): 2.3 μ_B_.

### Computational details

All calculations were carried out at the DFT level of theory using the hybrid functional B3PW91^59,60^, with the Gaussian 09 suite of programmes^61^. The U, Th, Si and Cs atoms were represented with a small-core Stuttgart–Dresden relativistic effective core potential associated with their adapted basis set^62–64^. All the other atoms Li, C, H, and N were described with a 6–31 G (d,p), double-ζ quality basis set^65–68^. The nature of the extrema (minimum) was established with analytical frequencies calculations and geometry optimisations were computed without any symmetry constraints. The SMD solvation model was used to evaluate solvation energies by a self-consistent reaction field approach based on accurate numerical solutions of the Poisson−Boltzmann equation^69^. Benzene was chosen as solvent. The enthalpy energy was computed at *T* = 298 K. Intrinsic Reaction Coordinates were carried out to verify the connections of the optimised transition states.

## Supplementary information

Supplementary Information

## Data Availability

The X-ray crystallographic data for **3M** and **5M** reported in this study have been deposited at the Cambridge Crystallographic Data Centre under accession codes 1997349-1997358. These data can be obtained free of charge from The Cambridge Crystallographic Data Centre (www.ccdc.cam.ac.uk/data_request/cif). All the other data supporting the findings of this study are available within the article, its Supplementary Information (Supplementary Figs. 1–45 and Tables 1–47), or from the corresponding authors upon reasonable request. Source data are available from the authors upon reasonable request.
