# Supplementary material for: Evidence for ligand- and solvent-induced disproportionation of uranium(IV)
Source: Nat Commun. 2021 Aug 10;12:4832. doi: 10.1038/s41467-021-25151-z (PMC8355312; doi:10.1038/s41467-021-25151-z)
Supplement: Supplementary file 1 — Supplementary Information [file 41467_2021_25151_MOESM1_ESM.pdf]

Supplementary Information

Evidence for Ligand- and Solvent-Induced Disproportionation of Uranium(IV)

Jingzhen Du,[1] Iskander Douair,[2] Erli Lu,[1] John A. Seed,[1] Floriana Tuna,[3] Ashley J. Wooles,[1] Laurent Maron,[2,*] and Stephen T. Liddle[1,*]

[1] Department of Chemistry, The University of Manchester, Oxford Road, Manchester, M13 9PL, UK. [2] LPCNO, CNRS & INSA, Université Paul Sabatier, 135 Avenue de Rangueil, Toulouse 31077, France. [3] Department of Chemistry and Photon Science Institute, The University of Manchester, Oxford Road, Manchester, M13 9PL, UK.

*For correspondance: steve.liddle@manchester.ac.uk; laurent.maron@irsamc.ups-tlse.fr.

Supplementary Figures

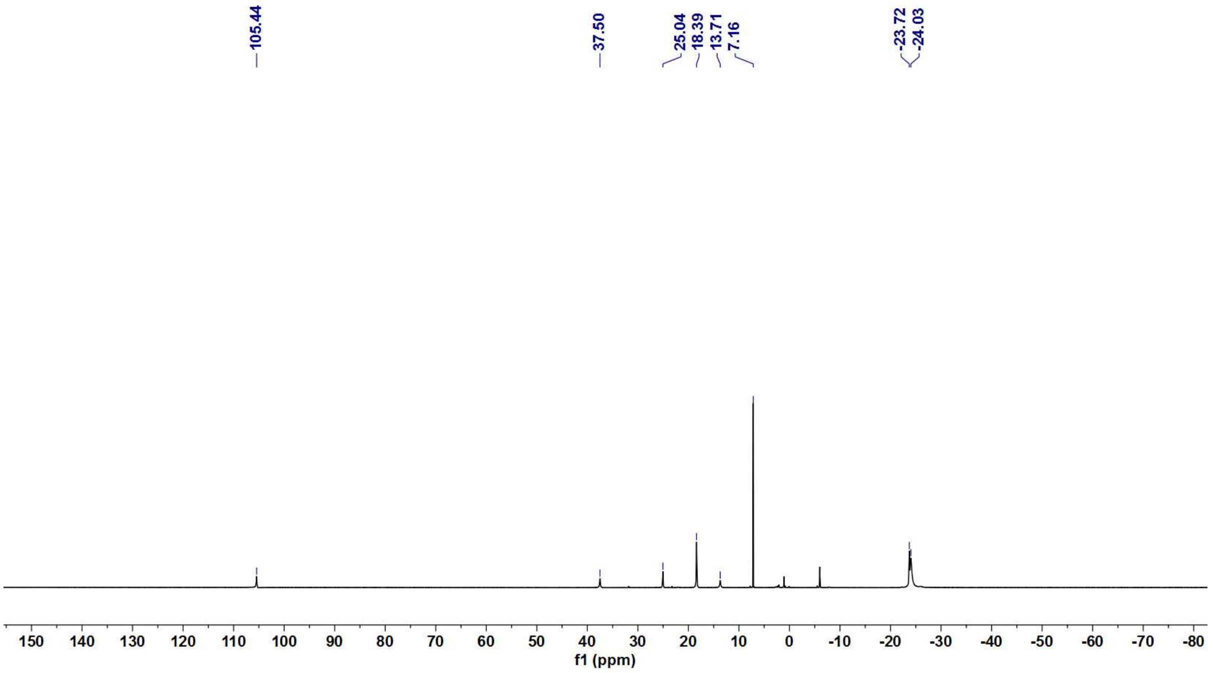

Supplementary Figure 1. ^{1}H NMR (400 MHz, C_6D_6, 298 K) of 3Li.

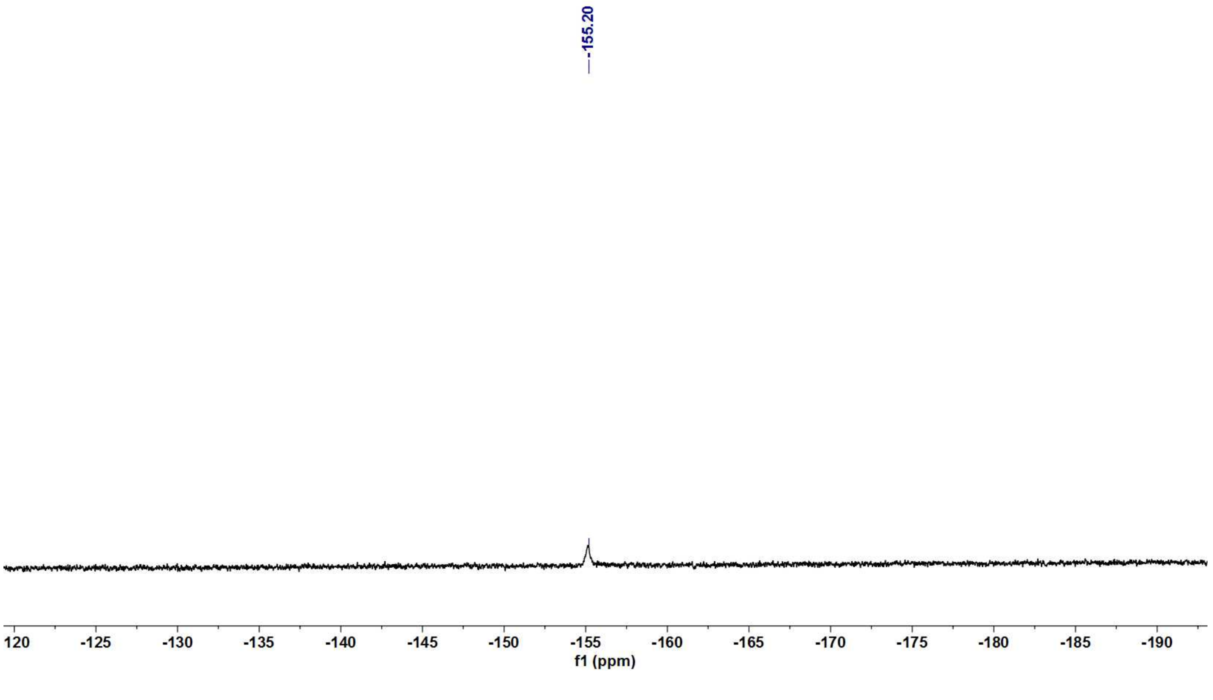

Supplementary Figure 2. $^{29}Si\{^{1}H\}$ NMR (79 MHz, C_6D_6, 298 K) of 3Li.

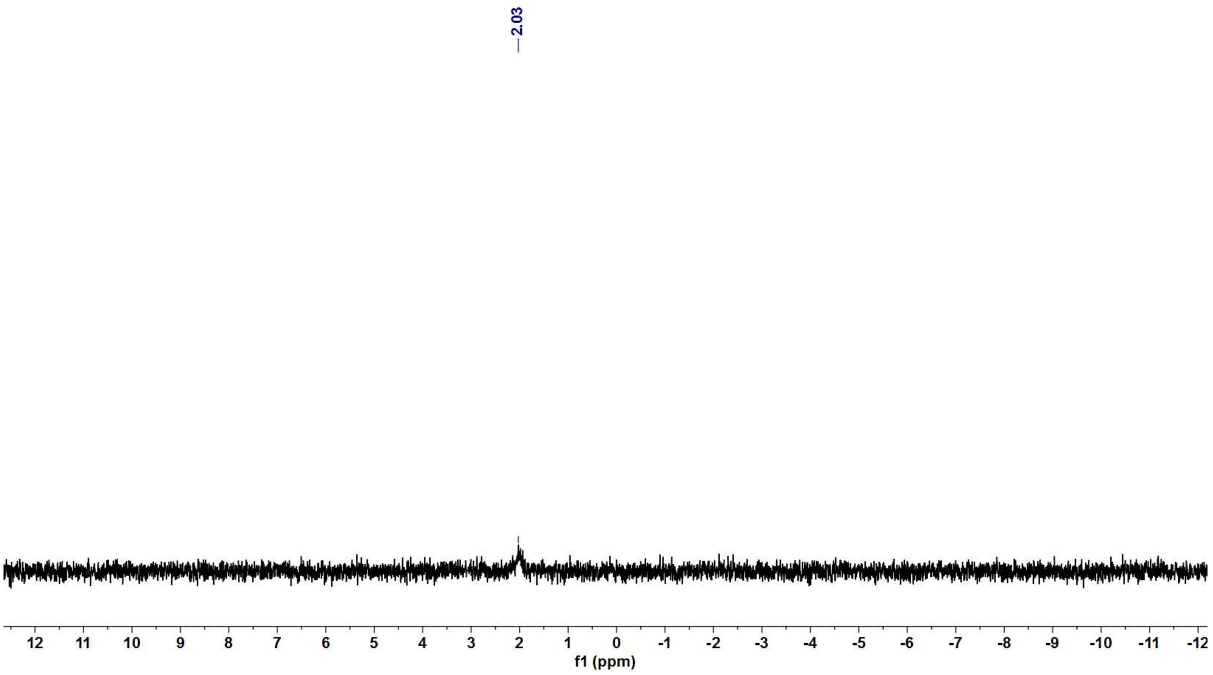

Supplementary Figure 3. $^{7}Li\{^{1}H\}$ NMR (155 MHz, 298 K, C_6D_6) of 3Li.

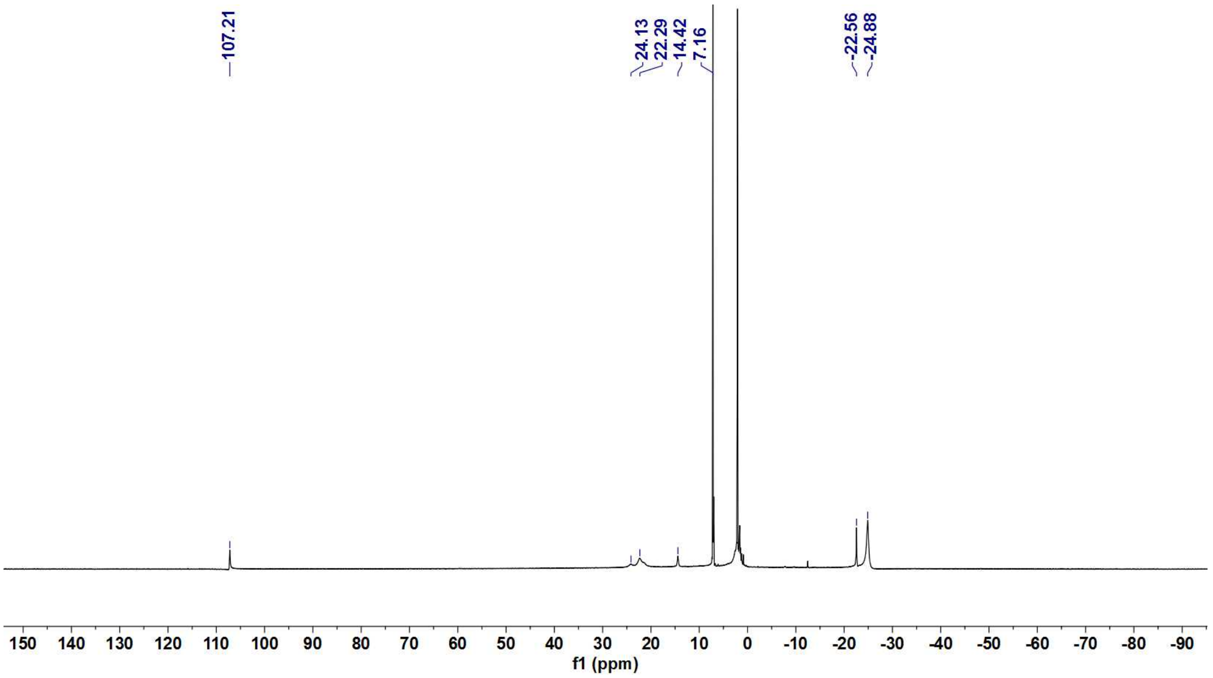

Supplementary Figure 4. ^{1}H NMR (400 MHz, C_6D_6, 298 K) of 3Na.

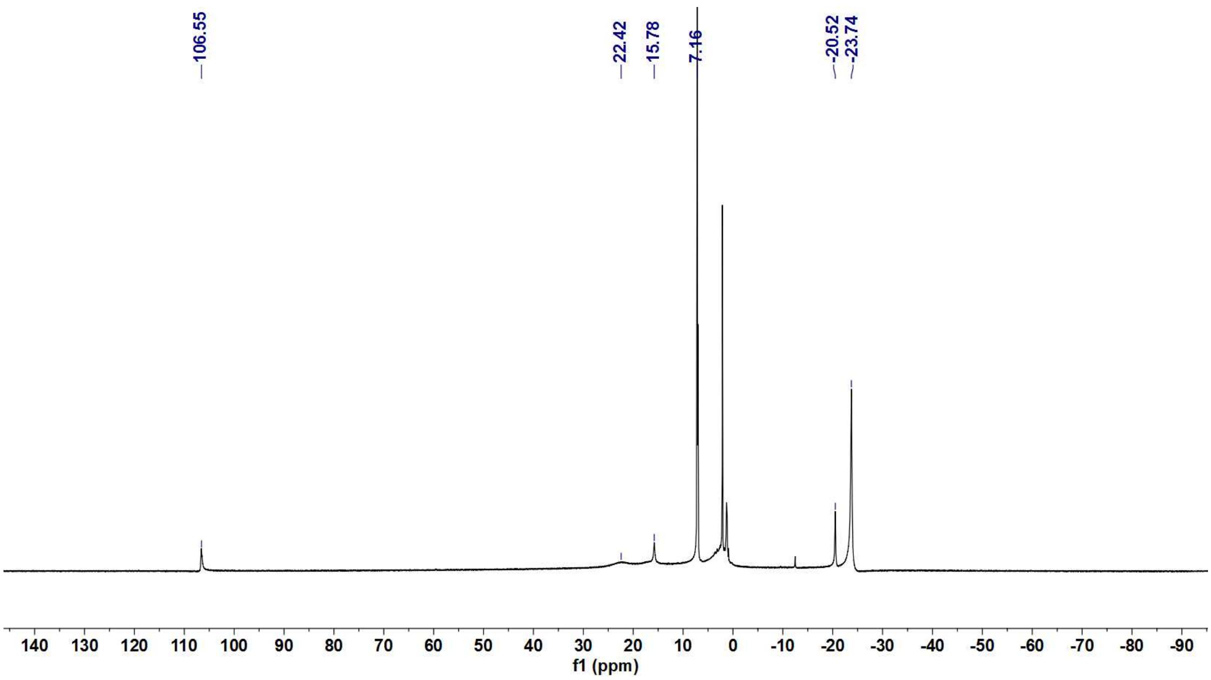

Supplementary Figure 5. ^{1}H NMR (400 MHz, C_6D_6, 298 K) of 3K.

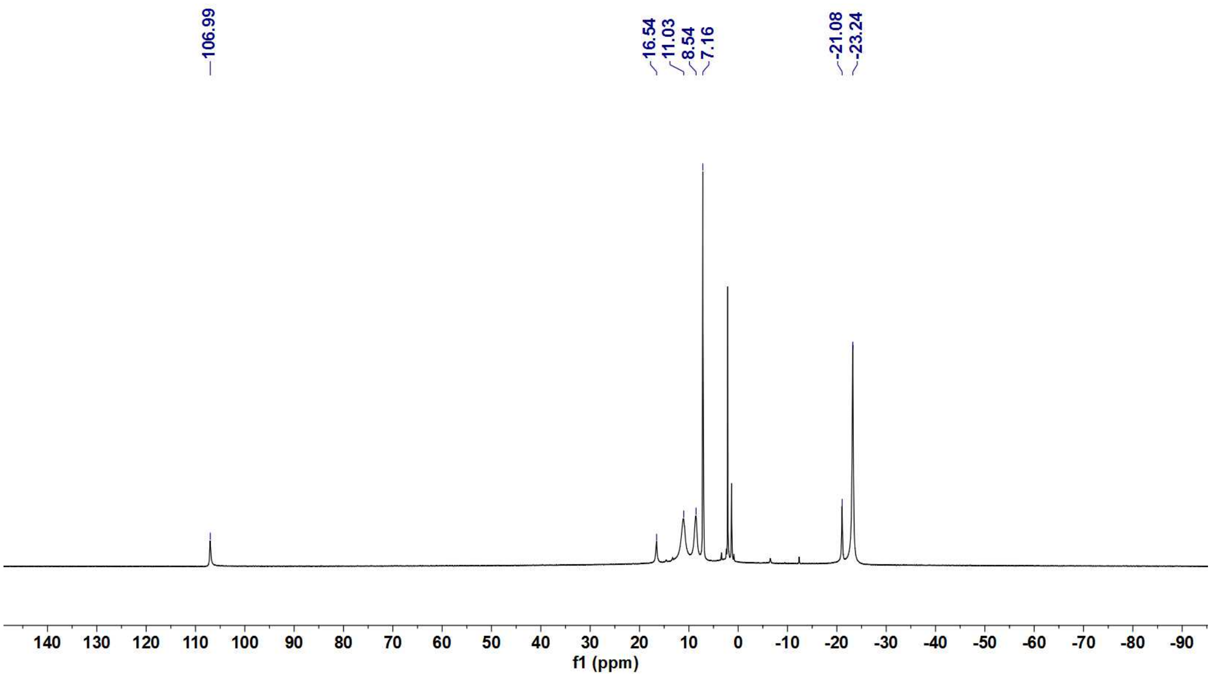

Supplementary Figure 6. ^{1}H NMR (400 MHz, C_6D_6, 298 K) of 3Rb.

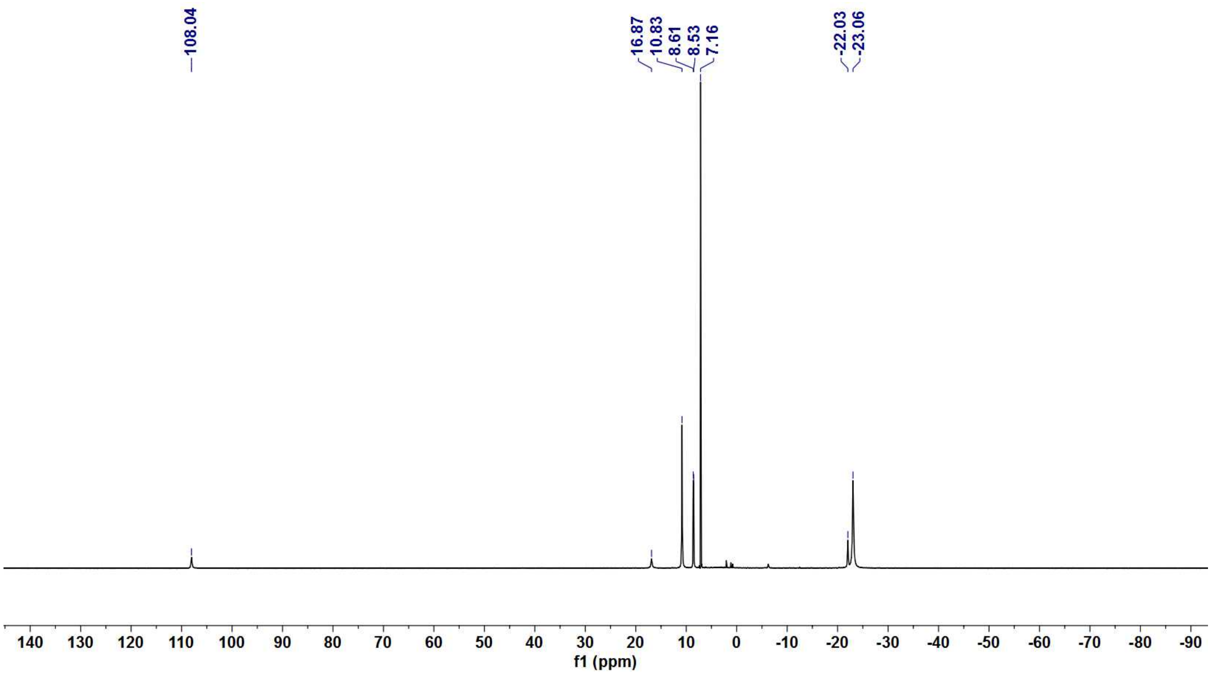

Supplementary Figure 7. ***^{1}H NMR (400 MHz, C_6D_6, 298 K) of 3Cs.***

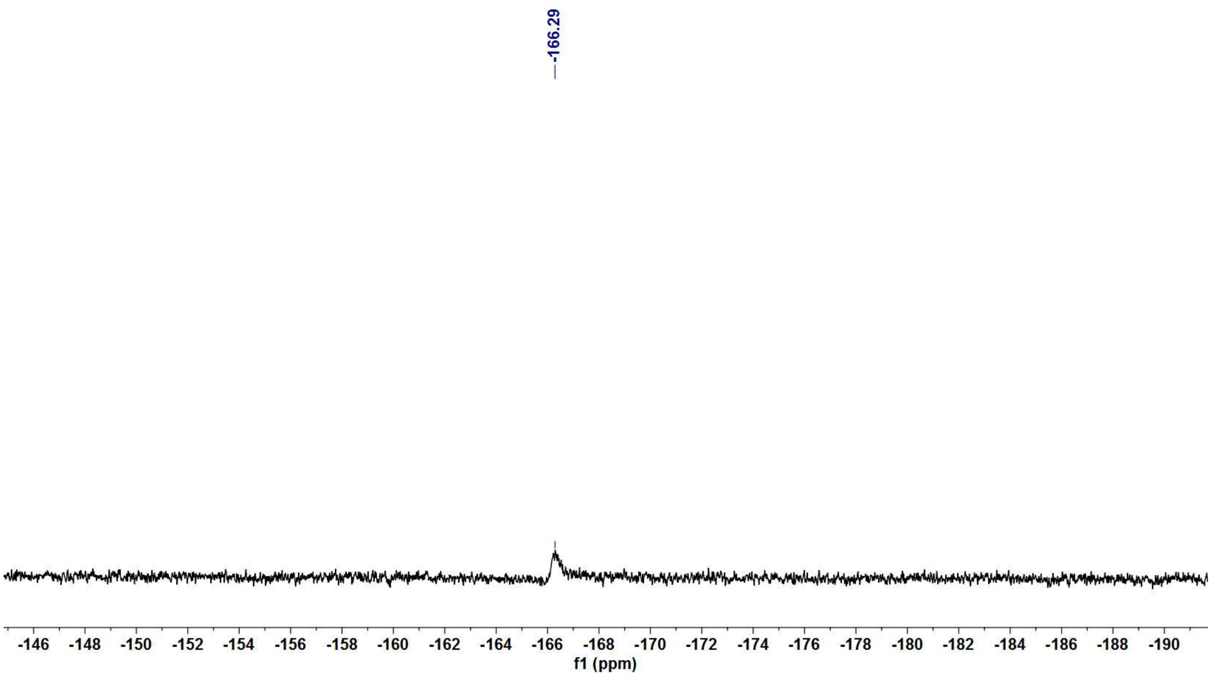

Supplementary Figure 8. ***$^{29}Si\{^{1}H\}$ NMR (79 MHz, C_6D_6, 298 K) of 3Cs.***

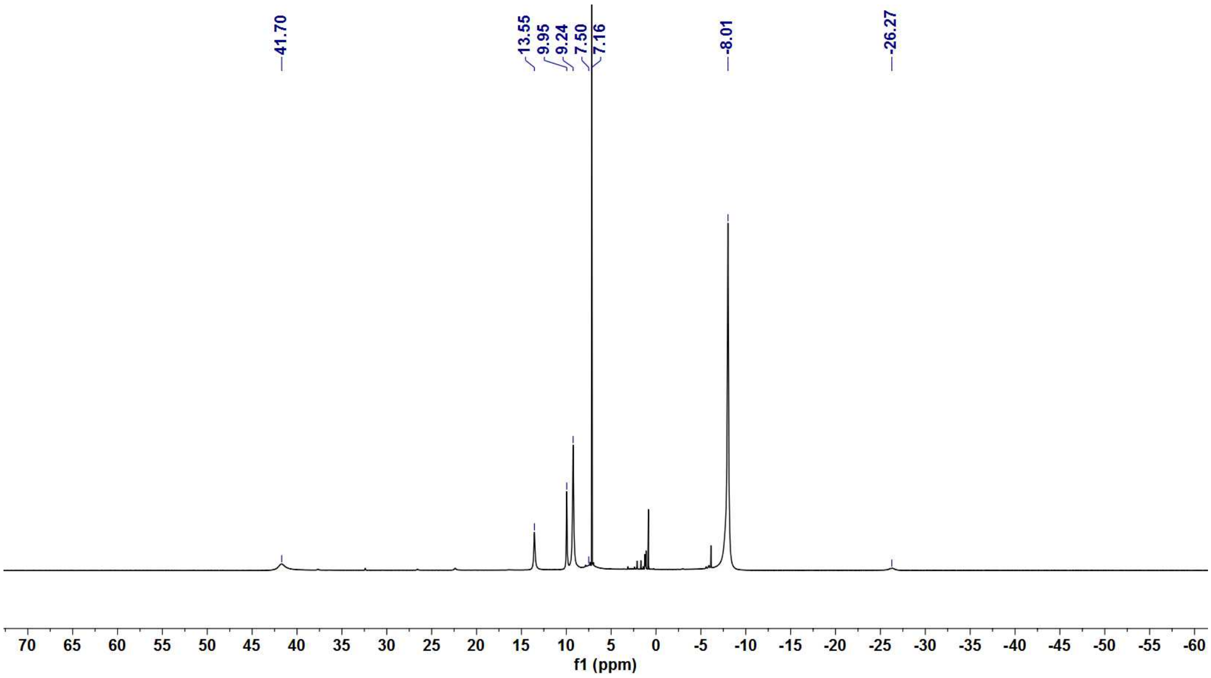

***Supplementary Figure 9.** ^{1}H NMR (400 MHz, C_6D_6, 298 K) of 5Li.*

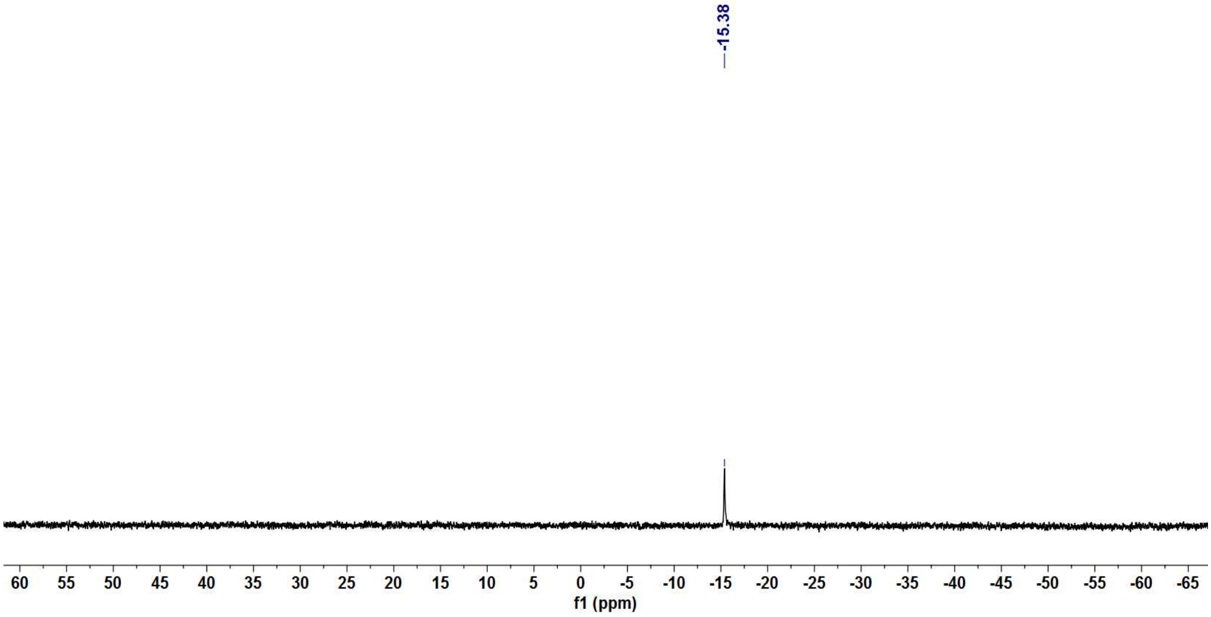

***Supplementary Figure 10.** ^{29}Si{^{1}H} NMR (79 MHz, C_6D_6, 298 K) of 5Li.*

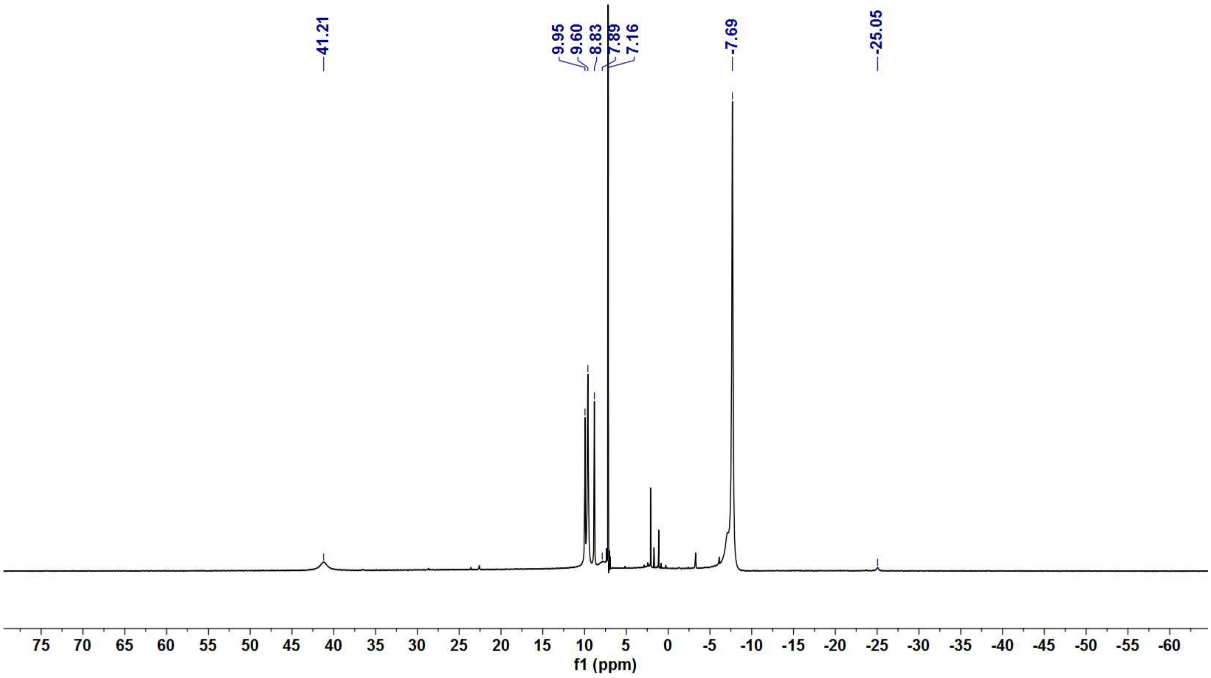

Supplementary Figure 11. ***^{1}H NMR (400 MHz, C_6D_6, 298 K) of 5Na.***

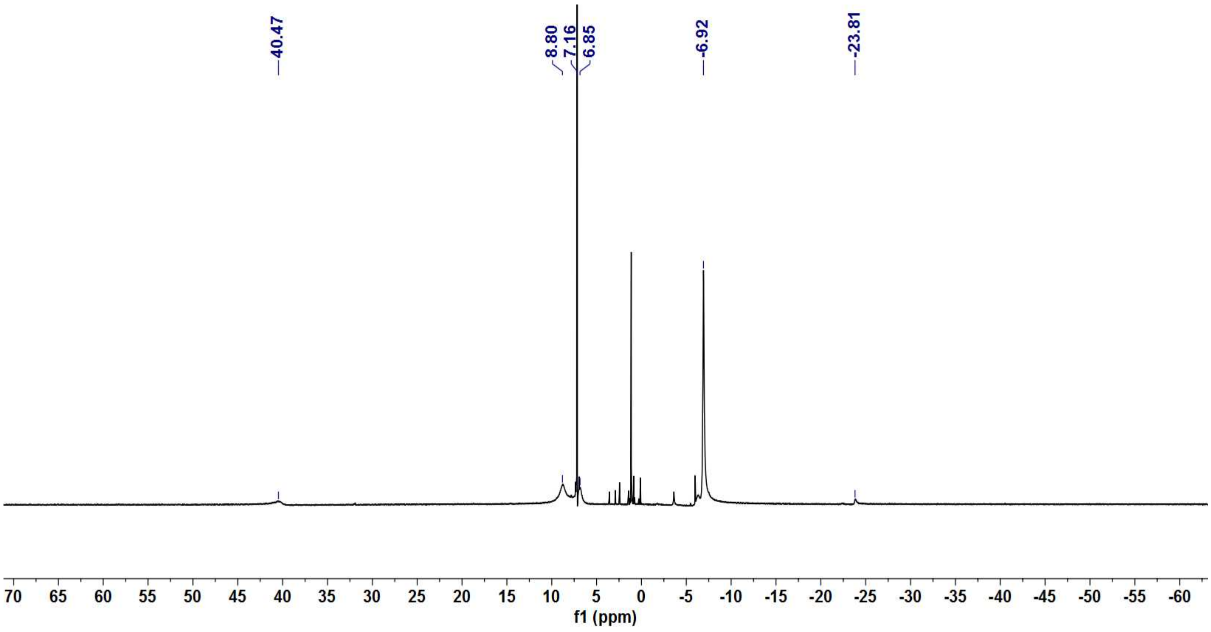

Supplementary Figure 12. ***^{1}H NMR (400 MHz, C_6D_6, 298 K) of 5K.***

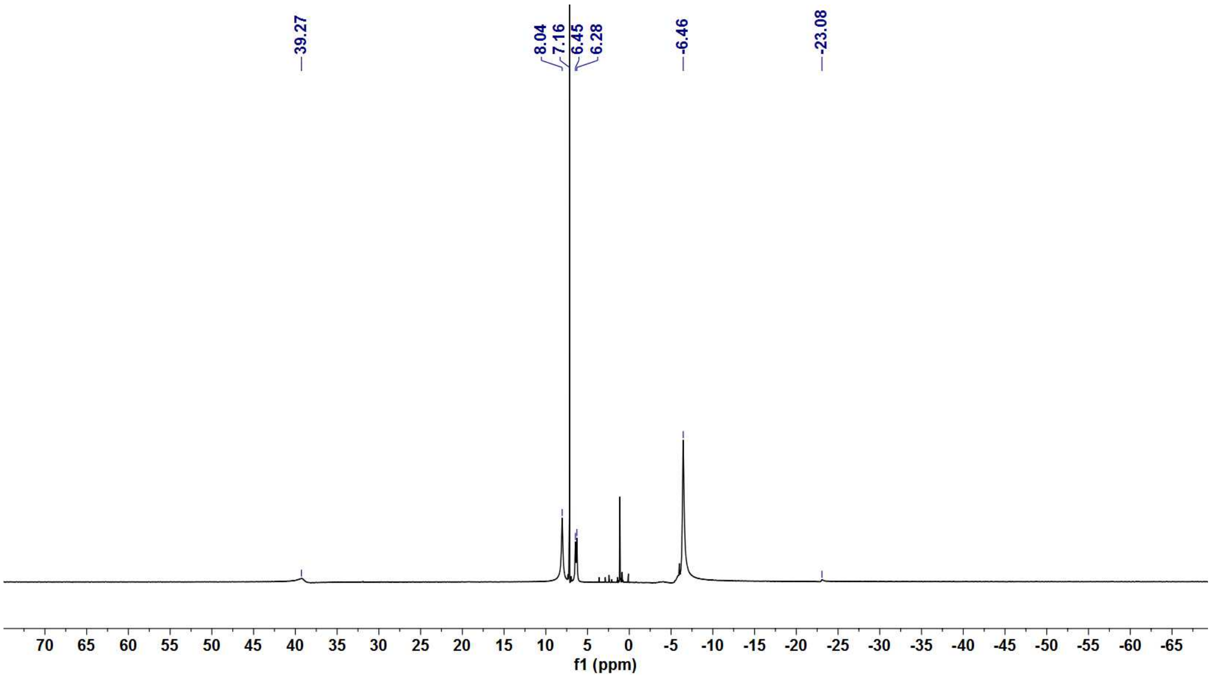

Supplementary Figure 13. ***^{1}H NMR (400 MHz, C_6D_6, 298 K) of 5Rb.***

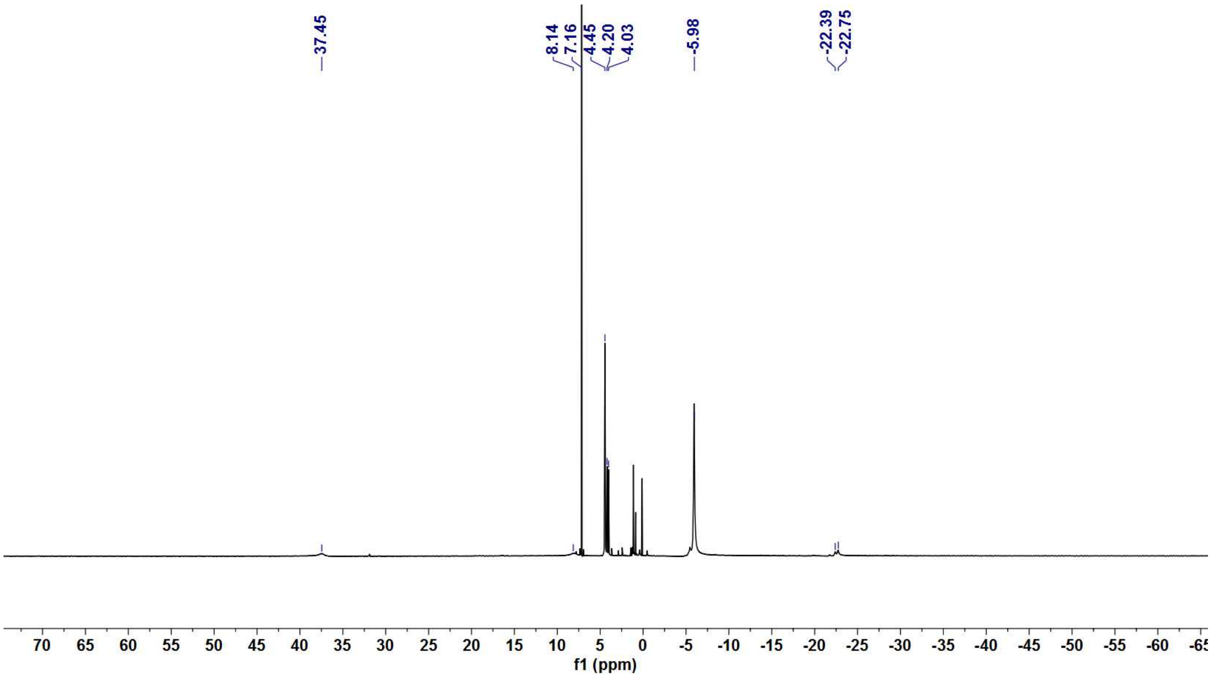

Supplementary Figure 14. ***^{1}H NMR (400 MHz, C_6D_6, 298 K) of 5Cs.***

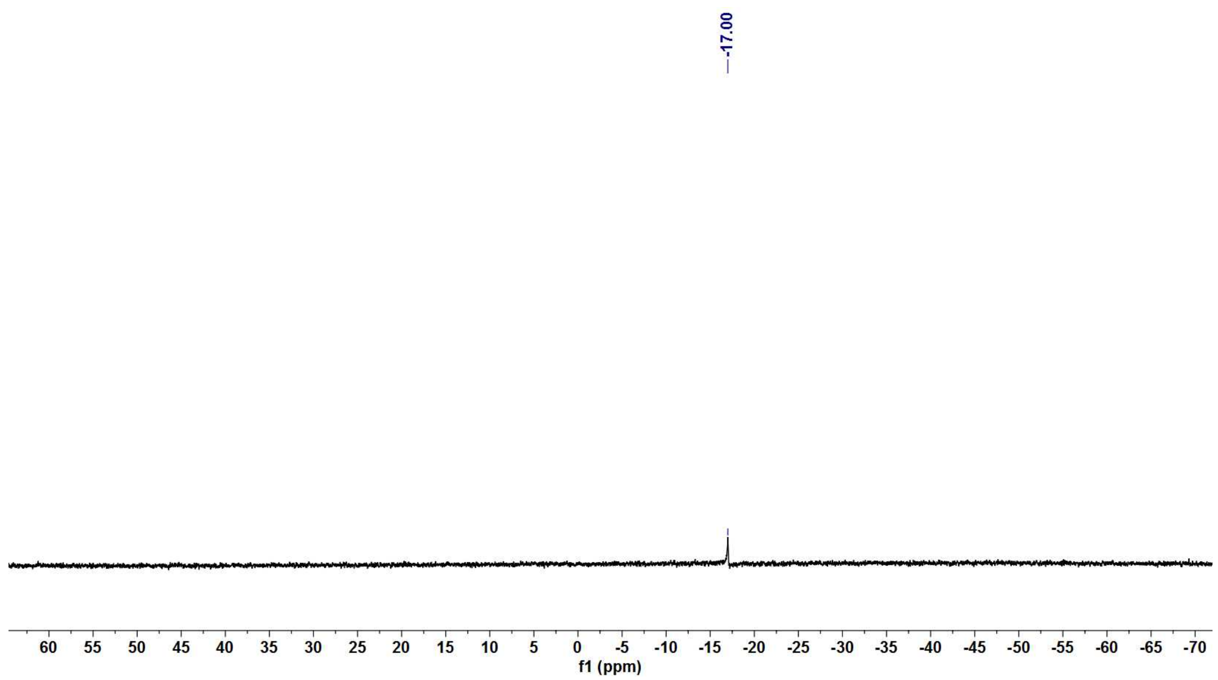

Supplementary Figure 15. ***$^{29}Si\{^1H\}$ NMR (79 MHz, C_6D_6, 298 K) of 5Cs.***

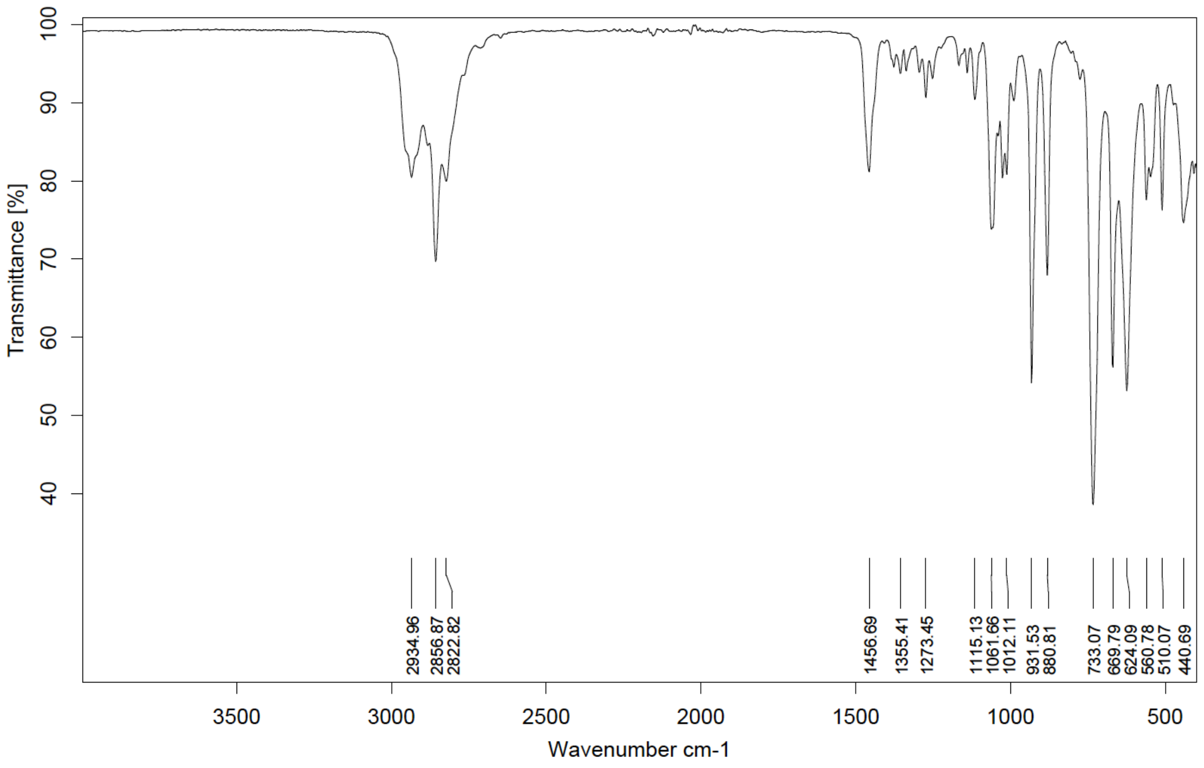

Supplementary Figure 16. ATR-IR spectrum of 3Li.

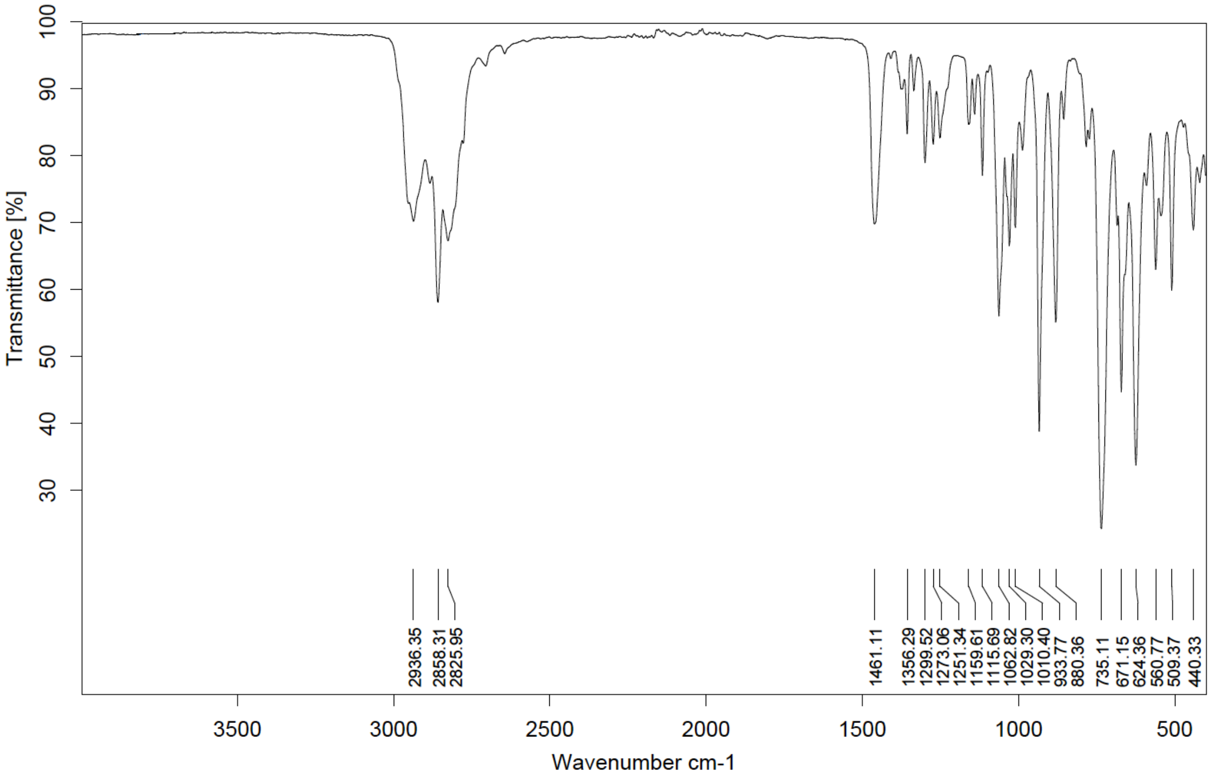

Supplementary Figure 17. ATR-IR spectrum of 3Na.

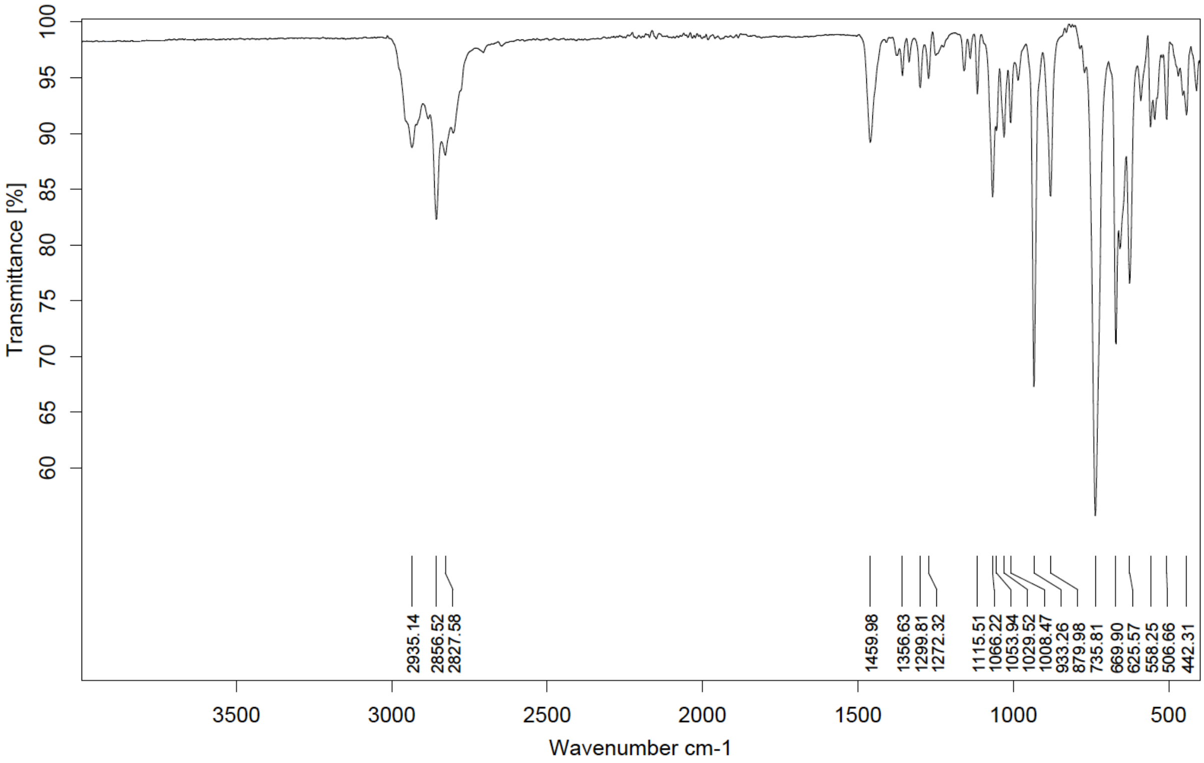

Supplementary Figure 18. ATR-IR spectrum of 3K.

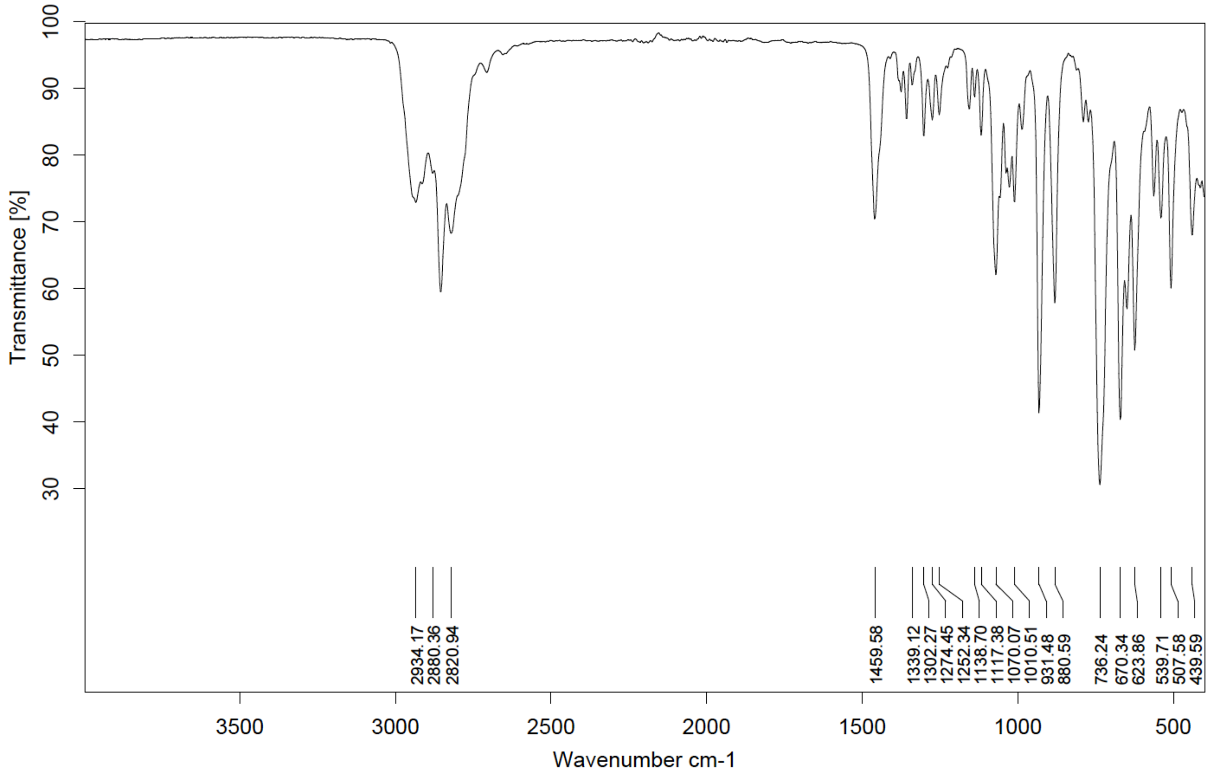

Supplementary Figure 19. ATR-IR spectrum of 3Rb.

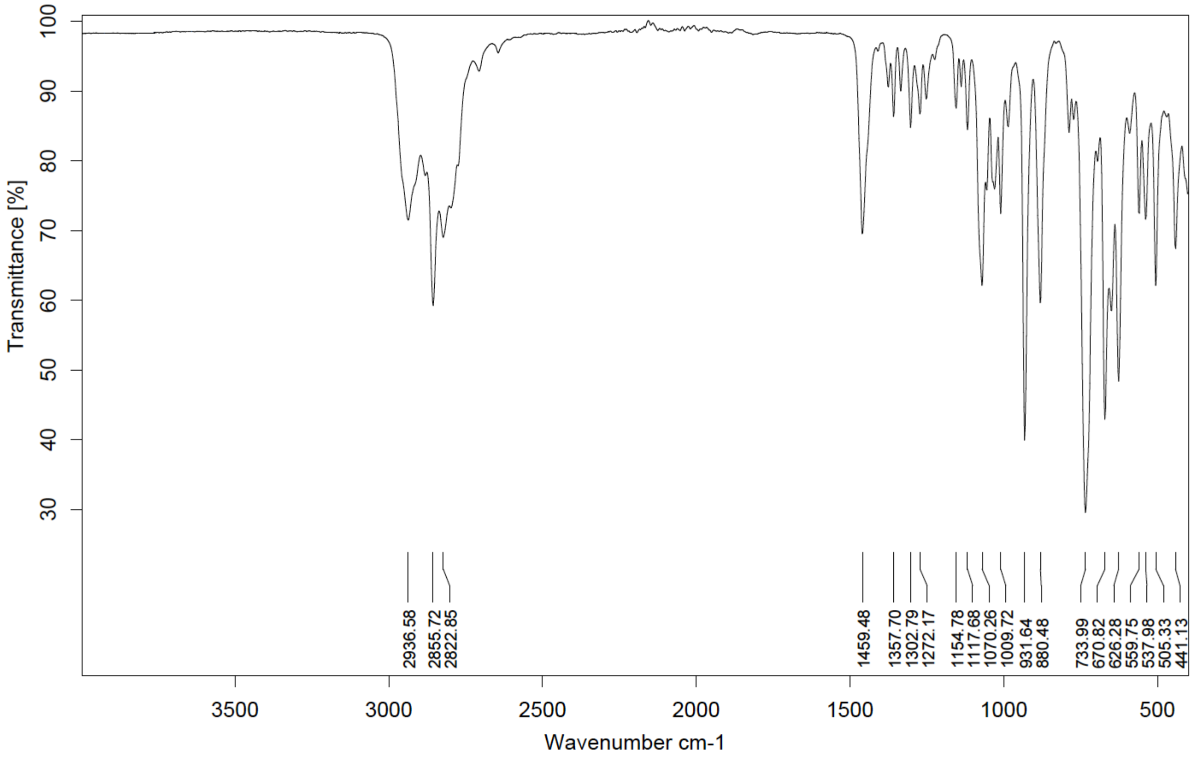

Supplementary Figure 20. ATR-IR spectrum of 3Cs.

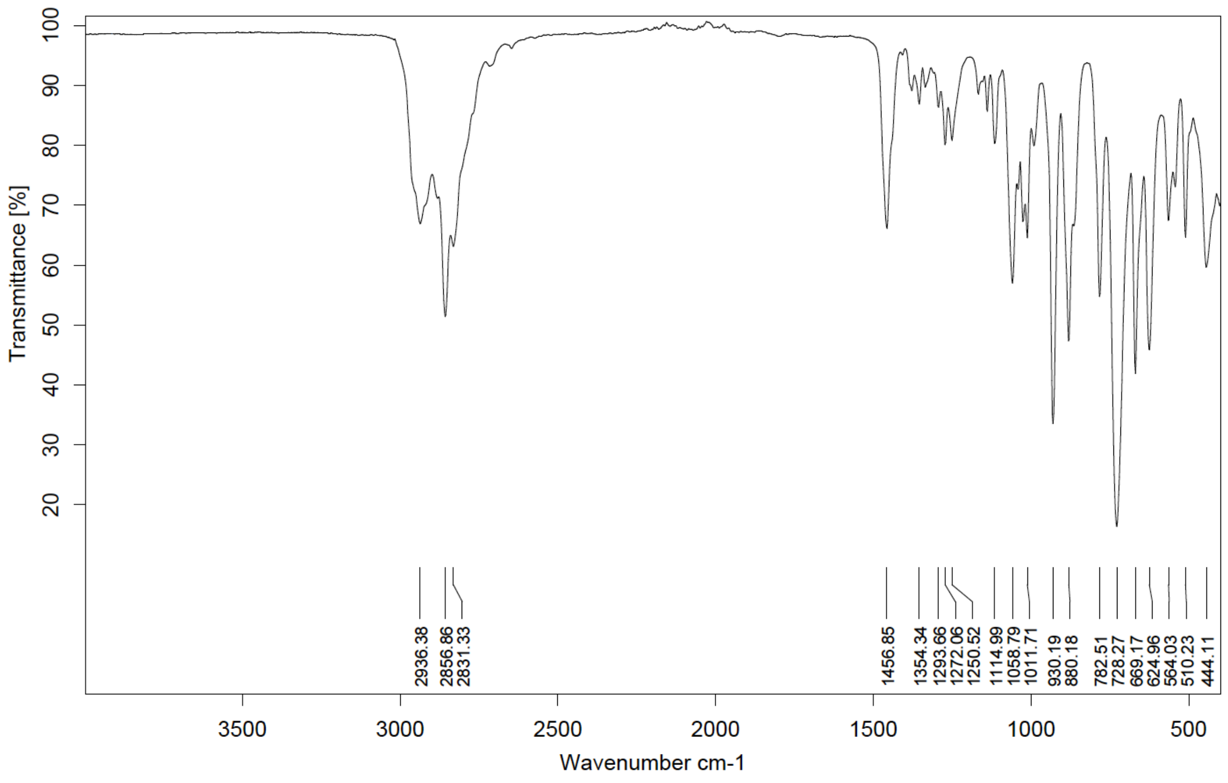

Supplementary Figure 21. ATR-IR spectrum of 5Li.

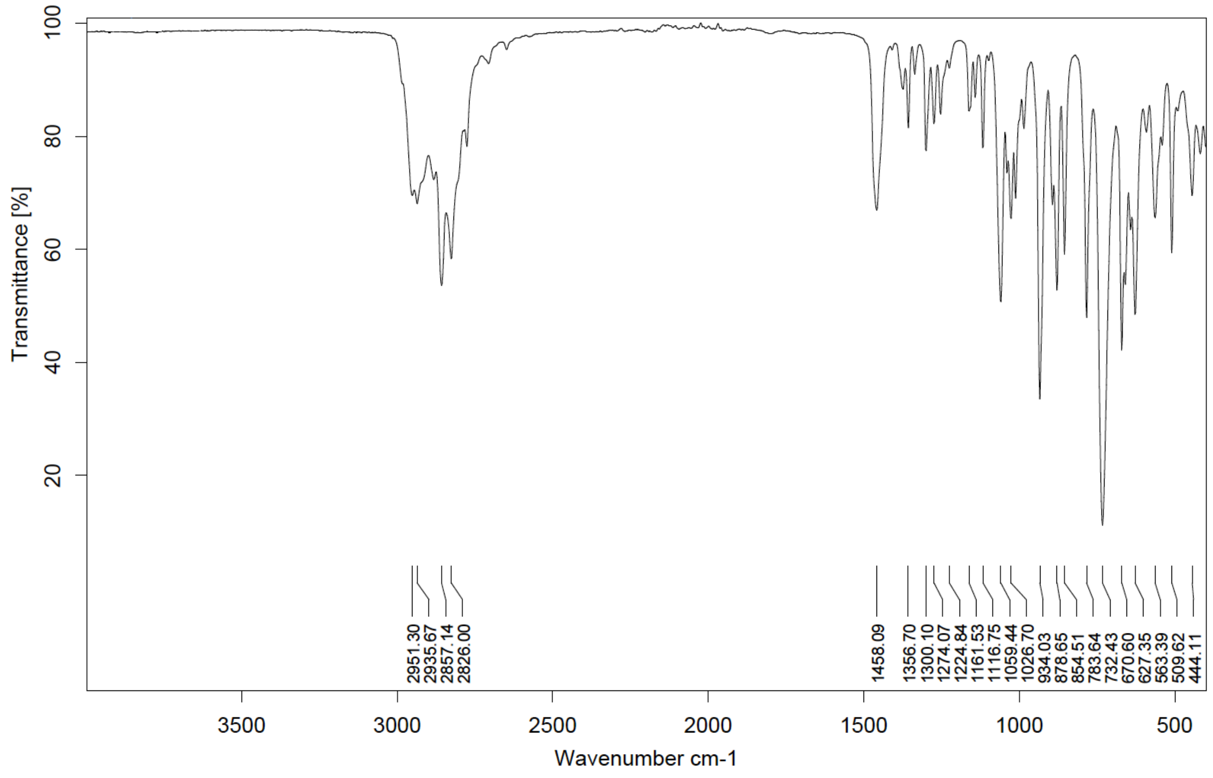

Supplementary Figure 22. ATR-IR spectrum of 5Na.

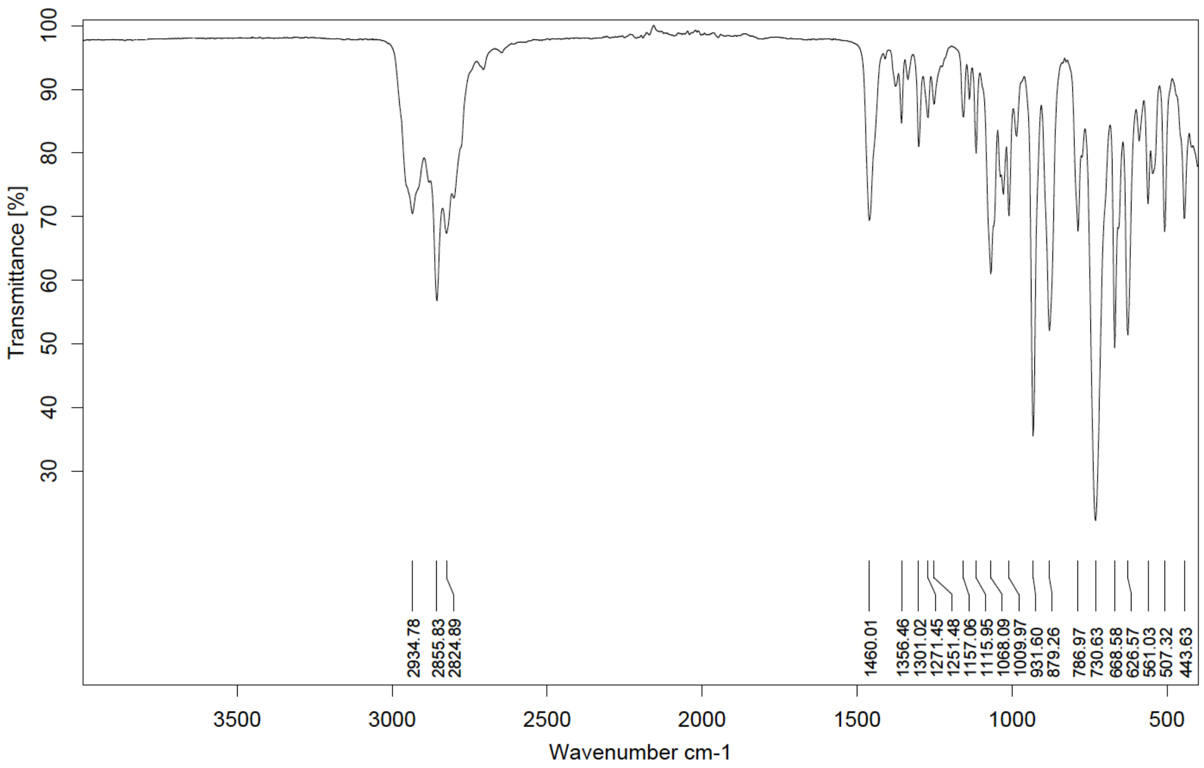

Supplementary Figure 23. ATR-IR spectrum of 5K.

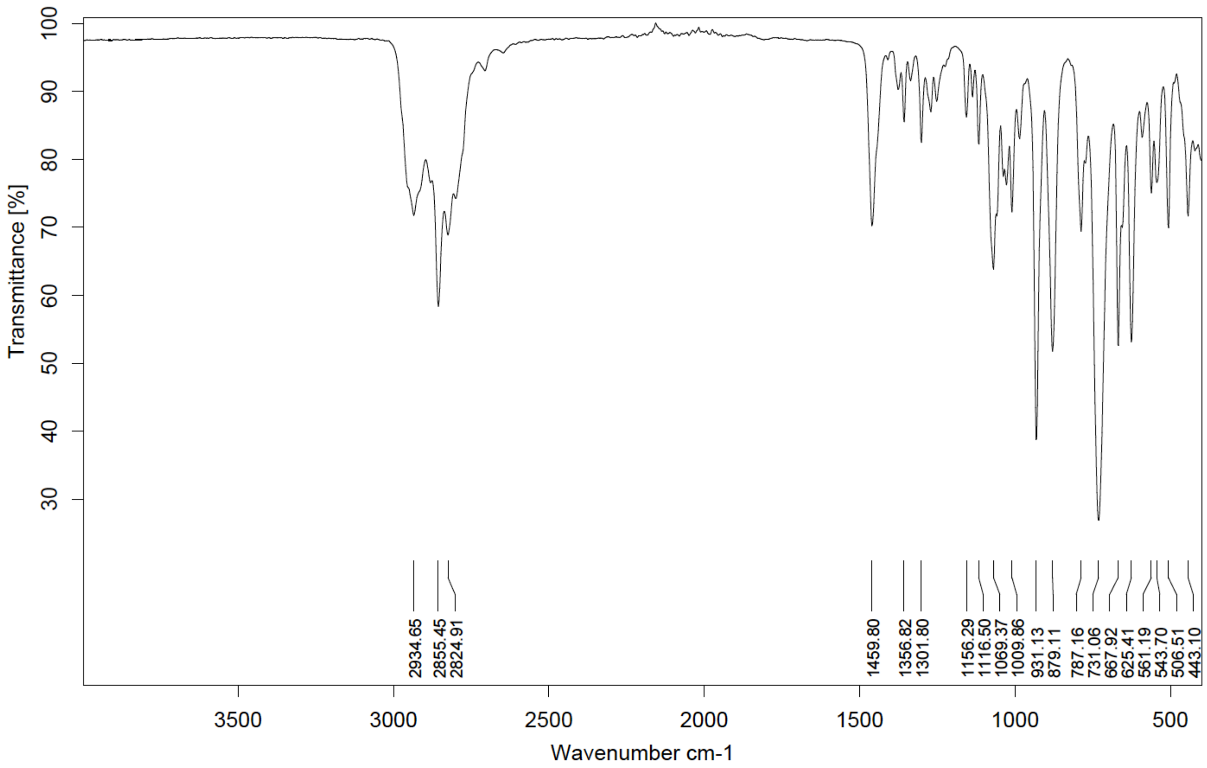

Supplementary Figure 24. ATR-IR spectrum of 5Rb.

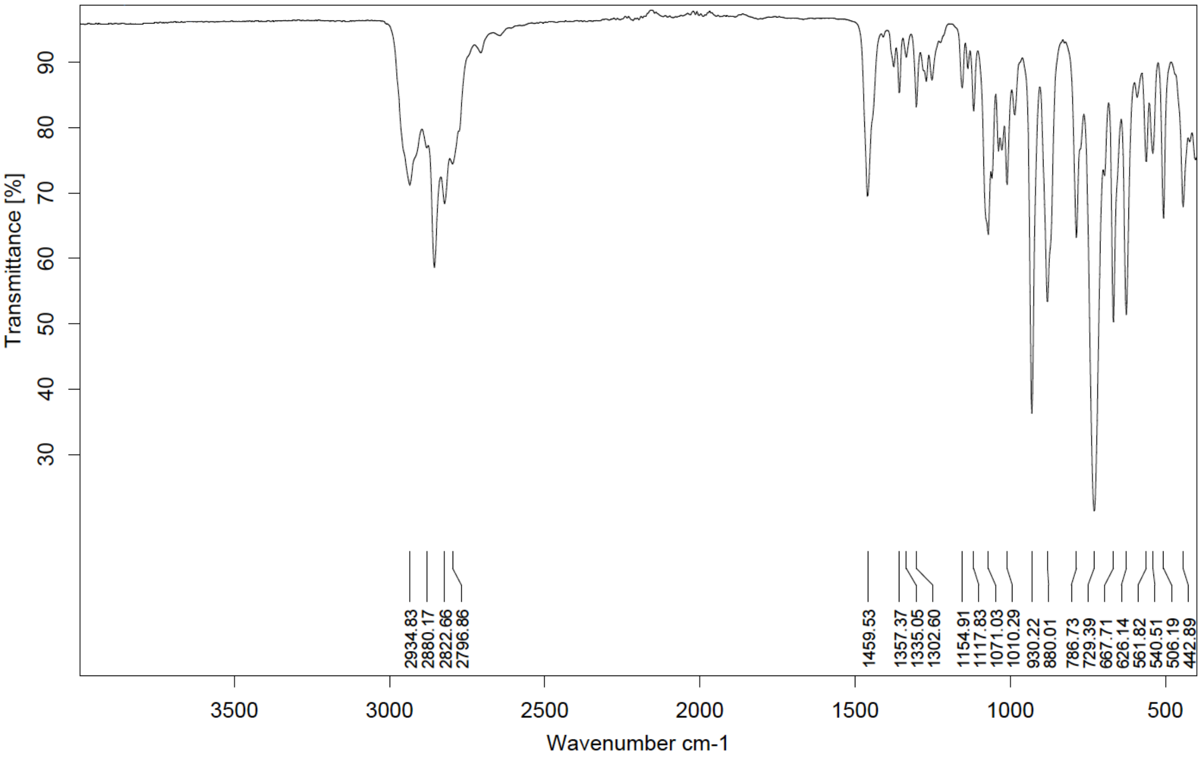

Supplementary Figure 25. ATR-IR spectrum of 5Cs.

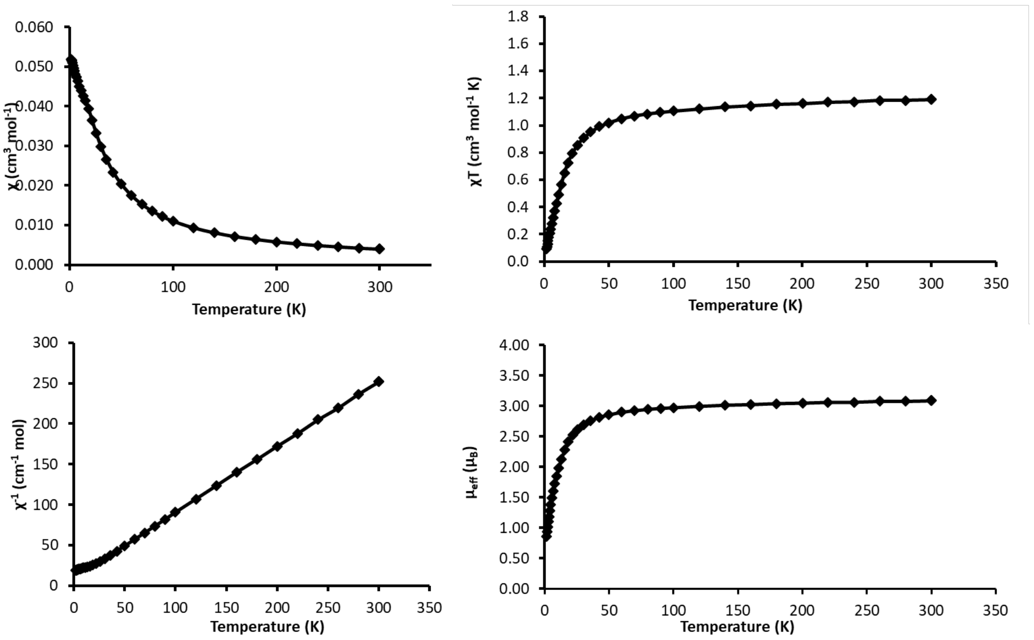

Supplementary Figure 26. Magnetic susceptibility χ, χT, magnetic moment μ_{eff} and $1/\chi$ data vs T for 3Li.

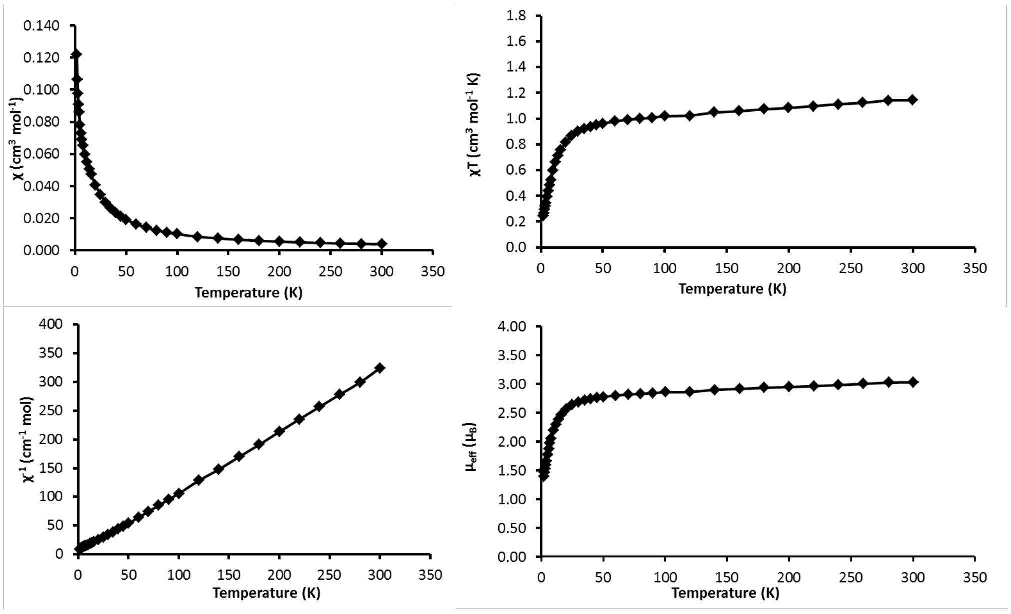

Supplementary Figure 27. Magnetic susceptibility χ, χT, magnetic moment μ_{eff} and 1/χ data vs T for 3Na.

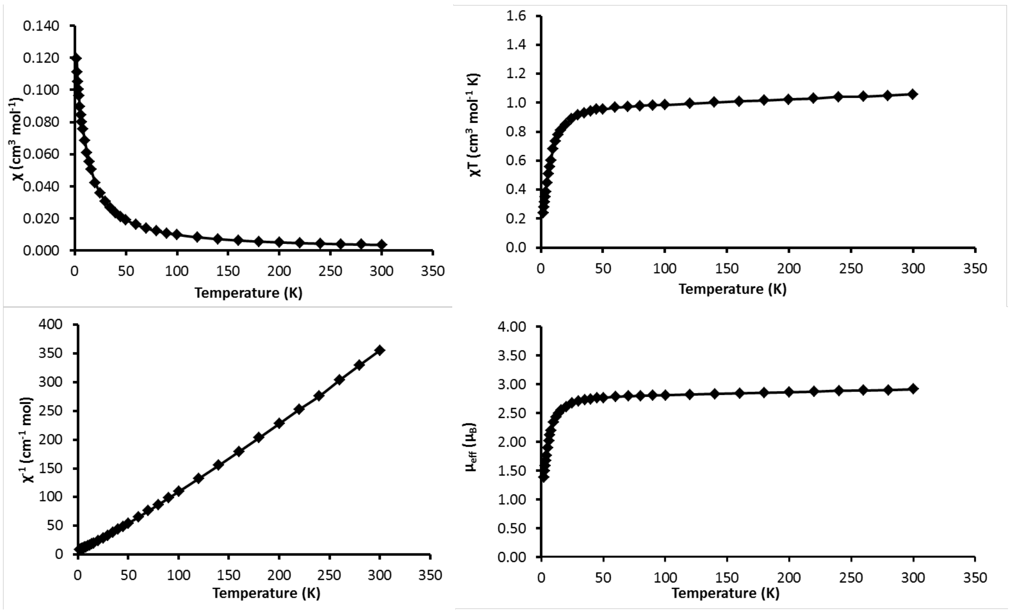

Supplementary Figure 28. Magnetic susceptibility χ, χT, magnetic moment μ_{eff} and 1/χ data vs T for 3K.

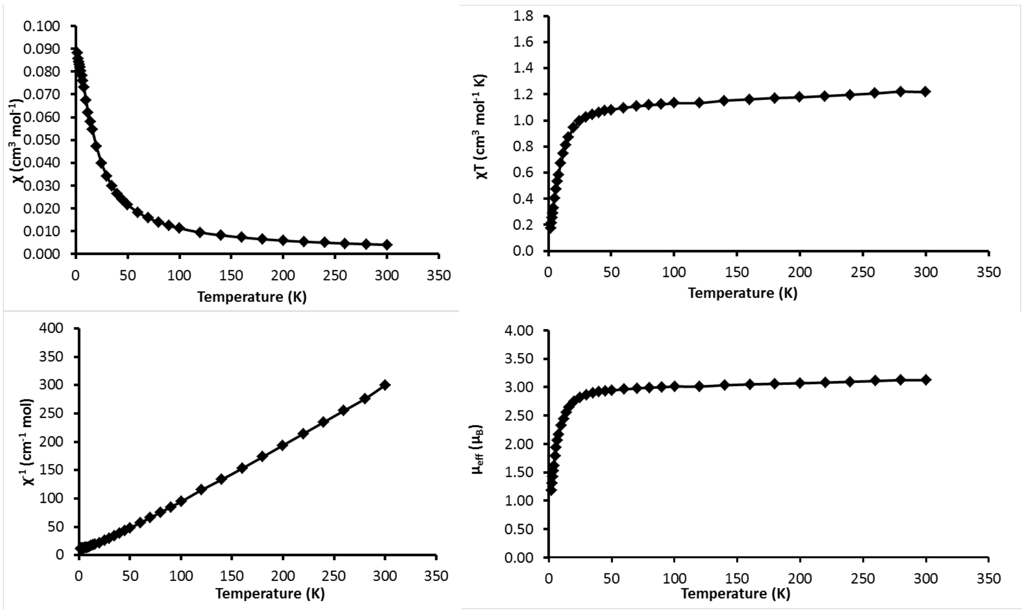

Supplementary Figure 29. Magnetic susceptibility χ, χT, magnetic moment μ_{eff} and 1/χ data vs T for 3Rb.

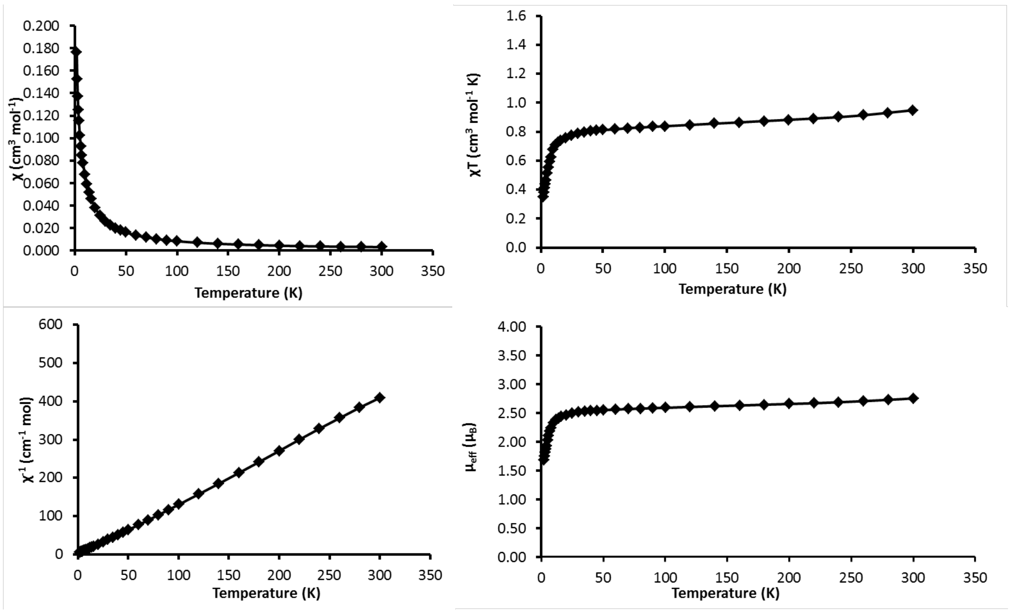

Supplementary Figure 30. Magnetic susceptibility χ, χT, magnetic moment μ_{eff} and 1/χ data vs T for 3Cs.

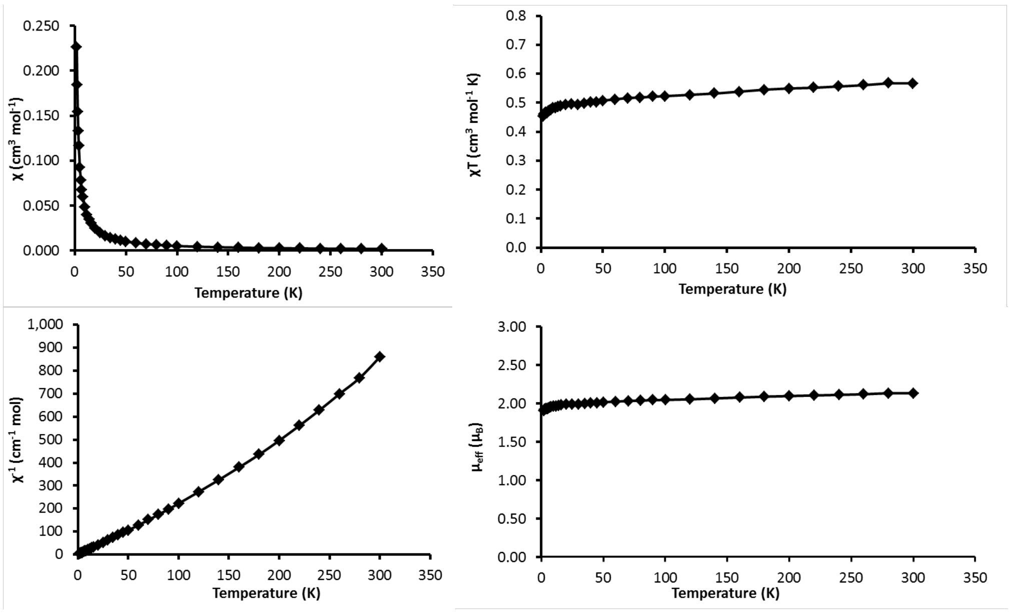

Supplementary Figure 31. Magnetic susceptibility χ, χT, magnetic moment μ_{eff} and 1/χ data vs T for 5Li.

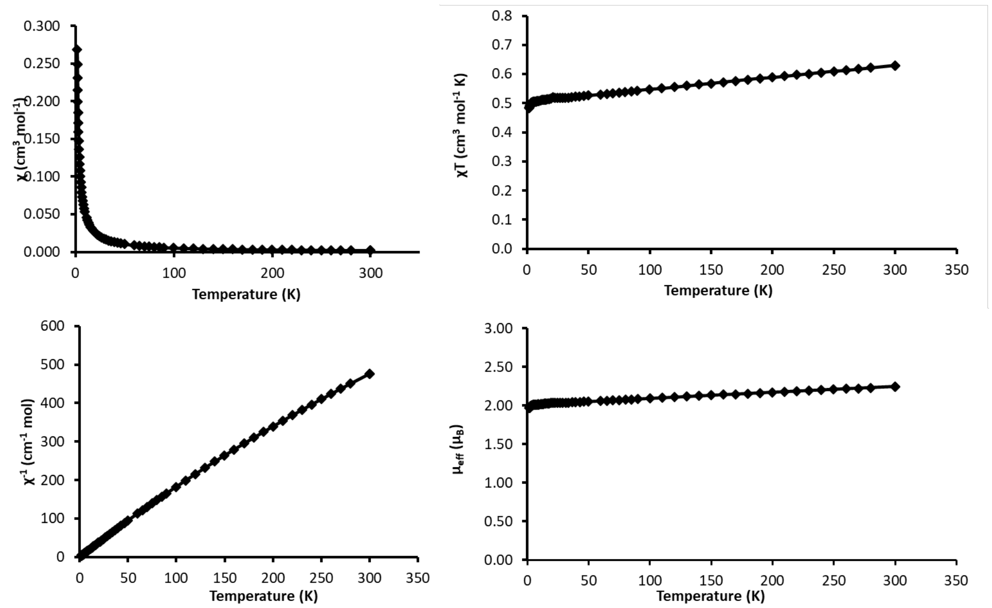

Supplementary Figure 32. Magnetic susceptibility χ, χT, magnetic moment μ_{eff} and 1/χ data vs T for 5Na.

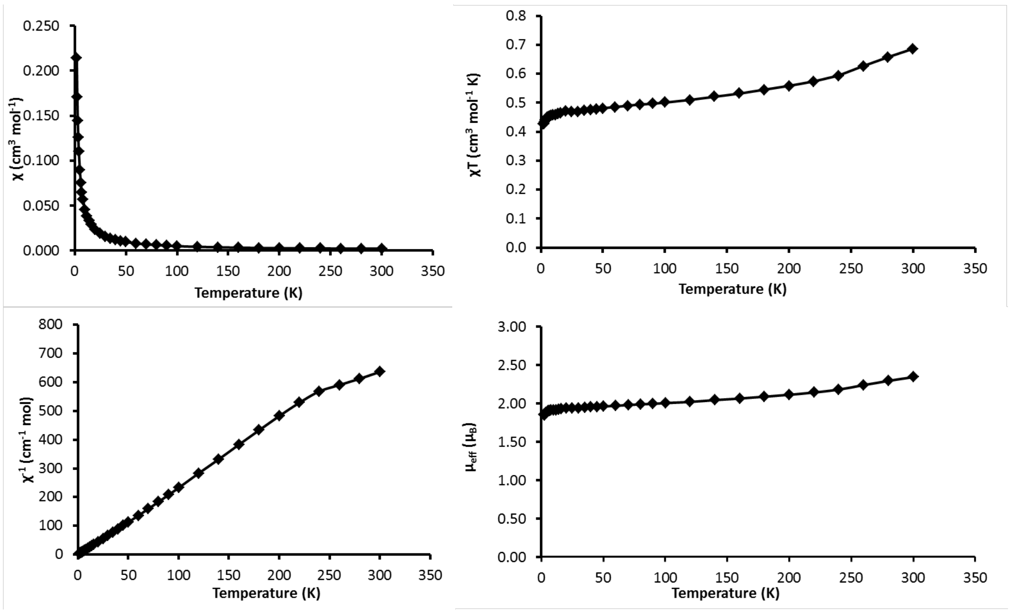

Supplementary Figure 33. Magnetic susceptibility χ, χT, magnetic moment μ_{eff} and 1/χ data vs T for 5K.

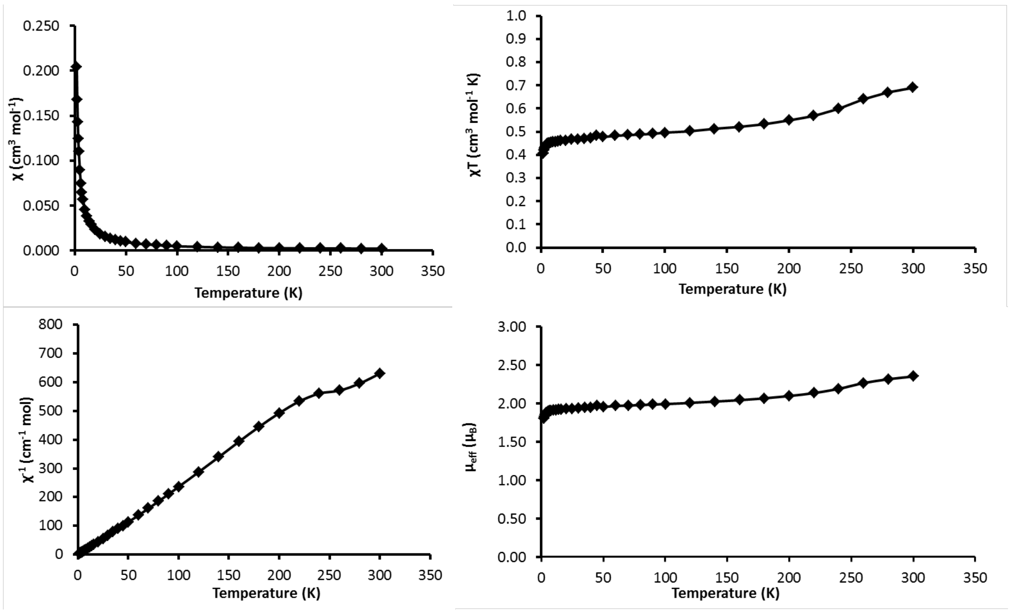

Supplementary Figure 34. Magnetic susceptibility χ, χT, magnetic moment μ_{eff} and 1/χ data vs T for 5Rb.

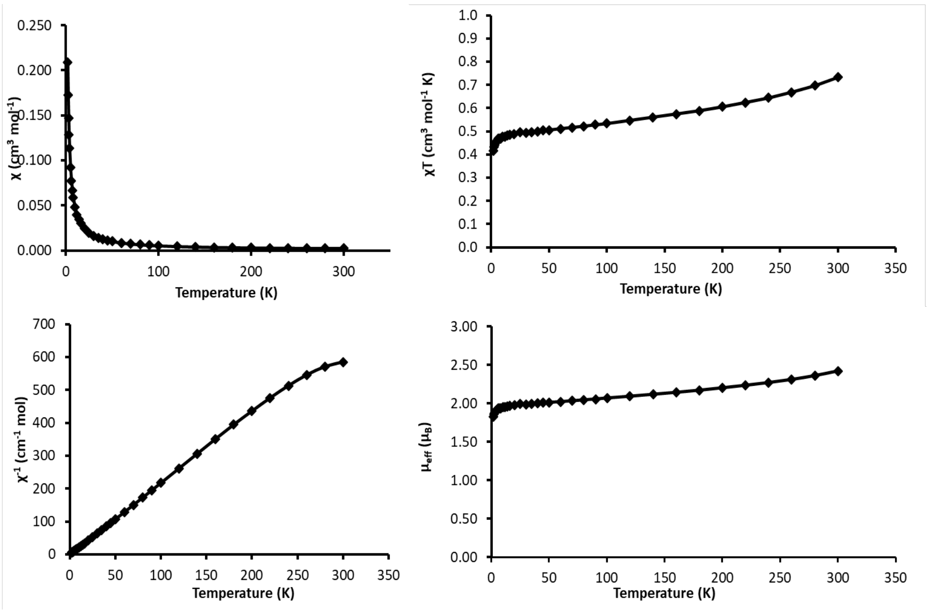

Supplementary Figure 35. Magnetic susceptibility χ, χT, magnetic moment μ_{eff} and $1/\chi$ data vs T for 5Cs.

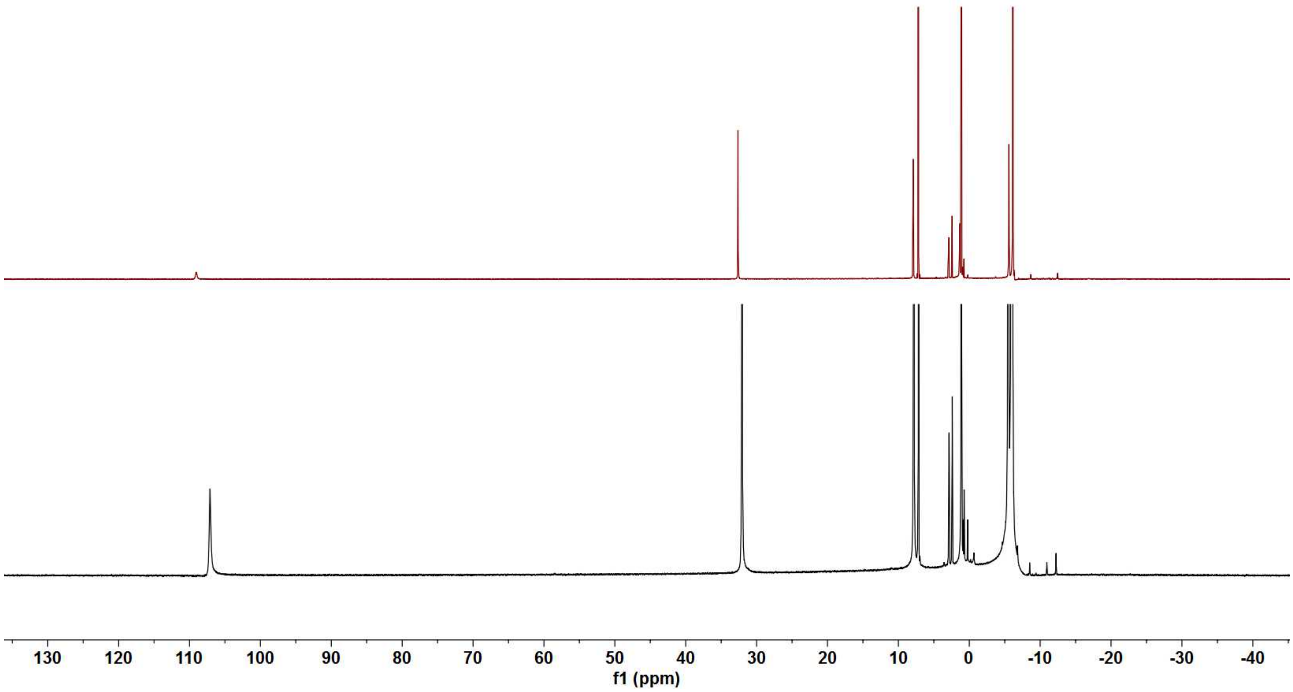

Supplementary Figure 36. ^1H NMR of the crude product from the mother liquor in the formation of 4K from 1 and KCH_2Ph in benzene (top) and comparison ^1H NMR spectrum of pure 1 (bottom).

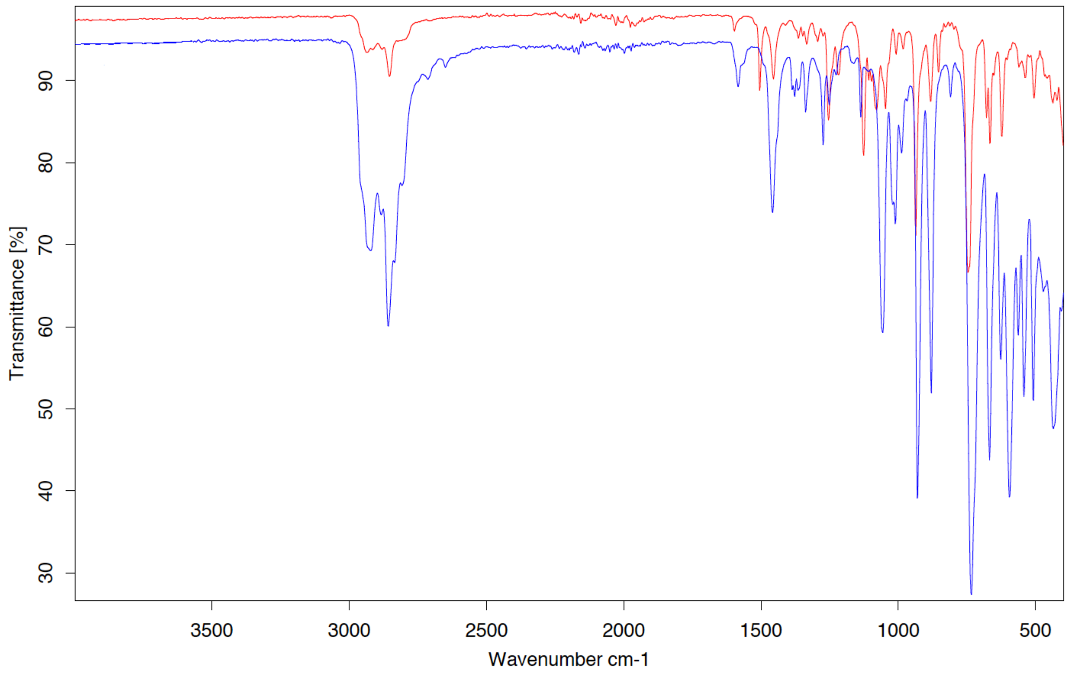

Supplementary Figure 37. ATR-IR spectra of [U(Tren^{TIPS})(NH_2)][K(benzo-15-crown-5)$_2$] (red), and the mixture from the reaction of 1 and 1.5 equivalents of KCH_2Ph after being heated at 80 ℃ for 30 min (blue).

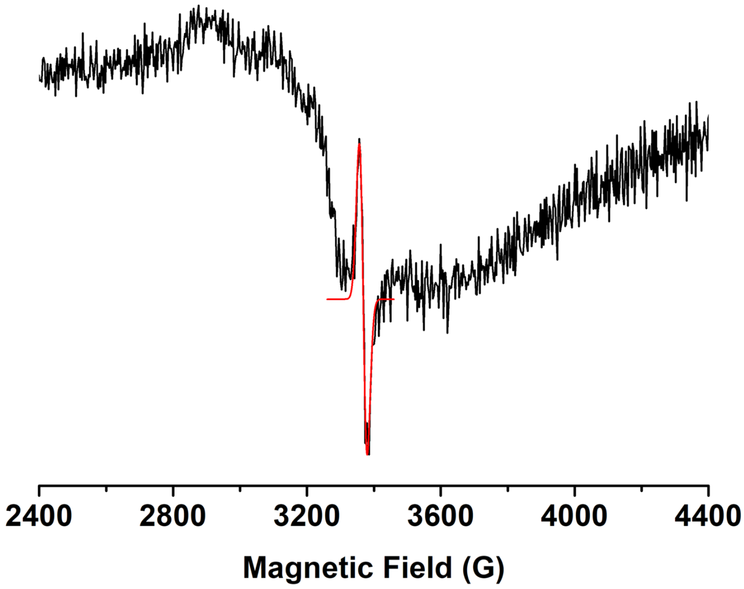

Supplementary Figure 38. Frozen solution X-band EPR Spectrum of the reaction mixture producing 4K and 6K from 2K, in benzene at 80 ^oC (black). Red: Simulated spectrum of the benzene anion radical with g = 2.0023, and line width 14 G.

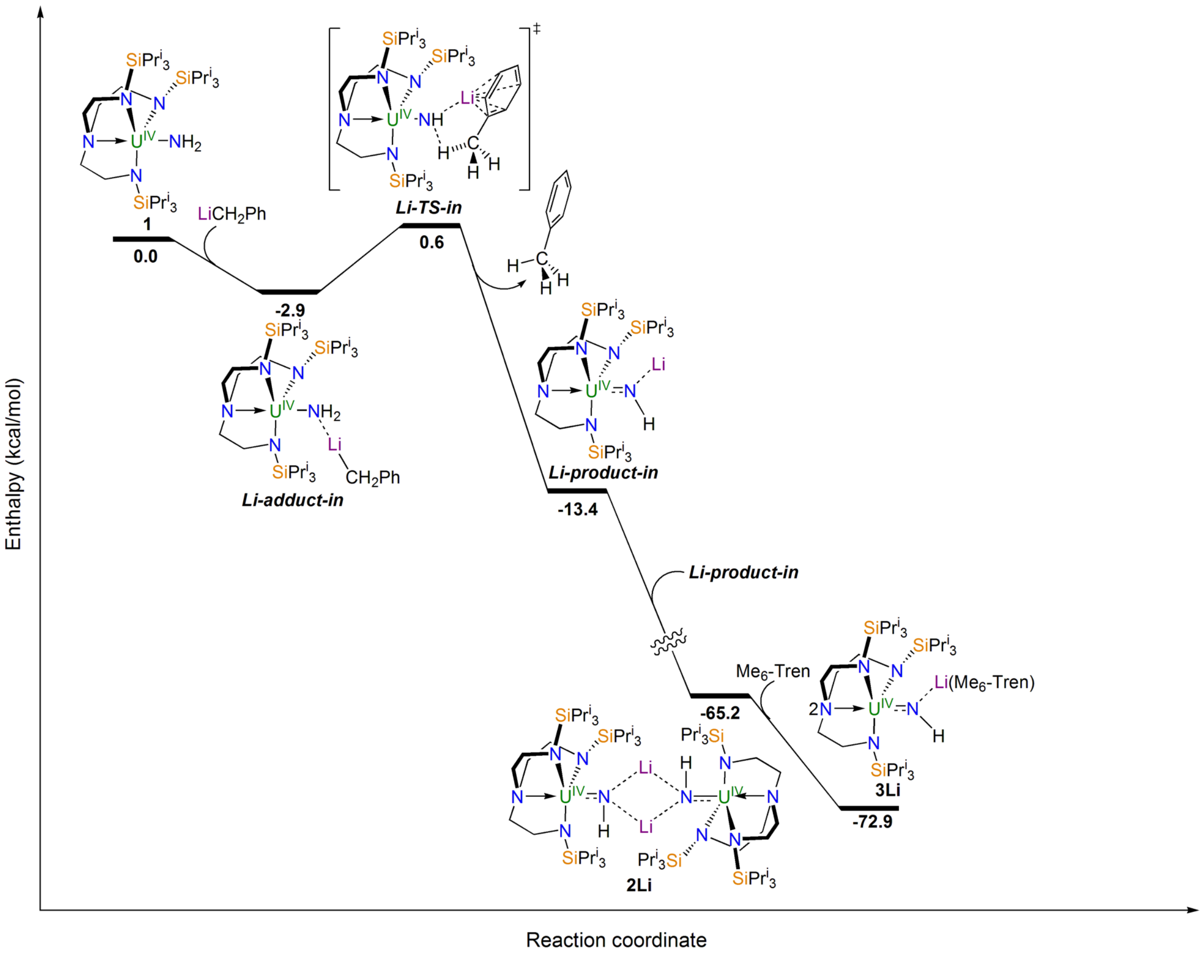

Supplementary Figure 39. Computed reaction profile for the conversion of 1 to 2Li then 3Li.

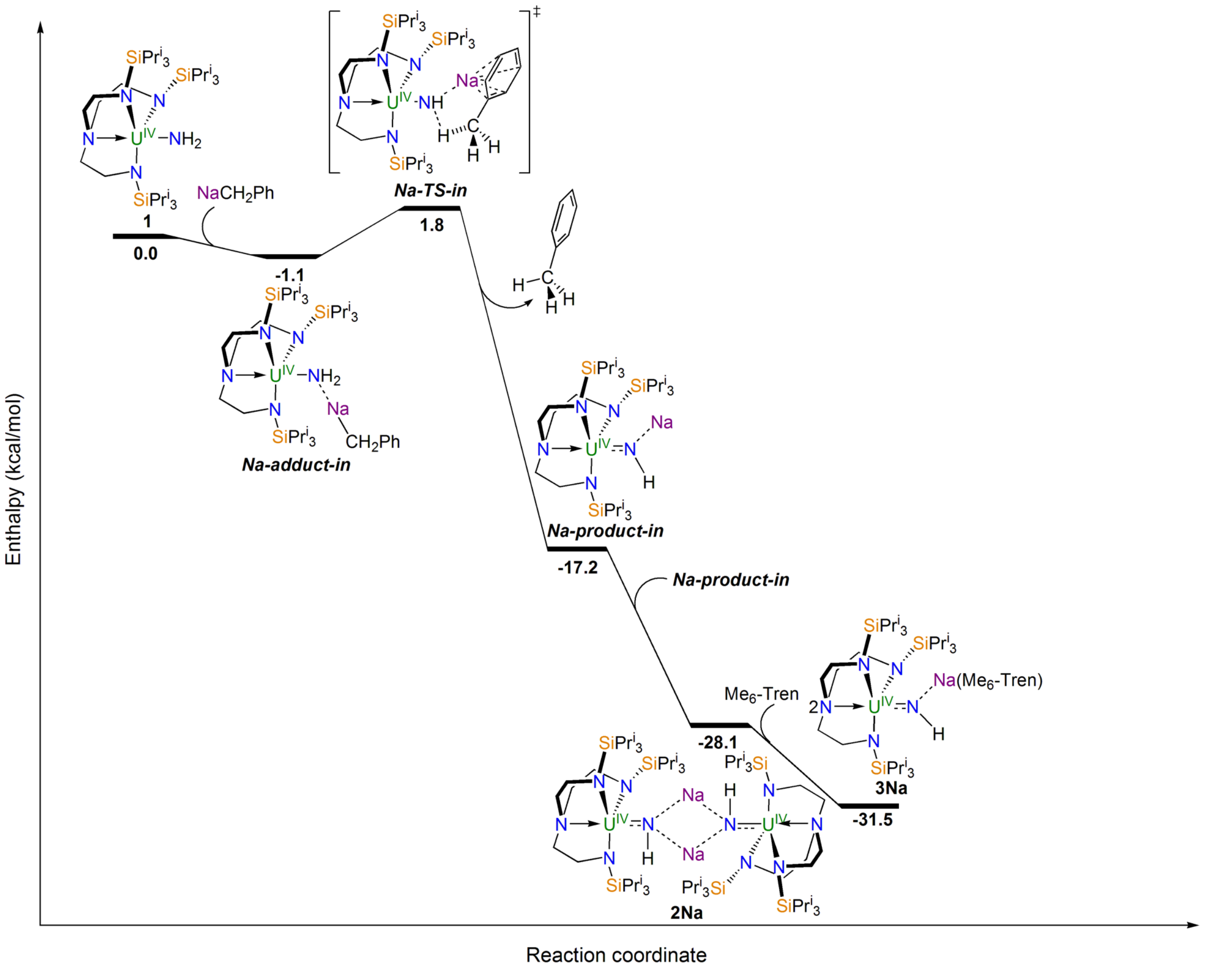

Supplementary Figure 40. Computed reaction profile for the conversion of 1 to 2Na then 3Na.

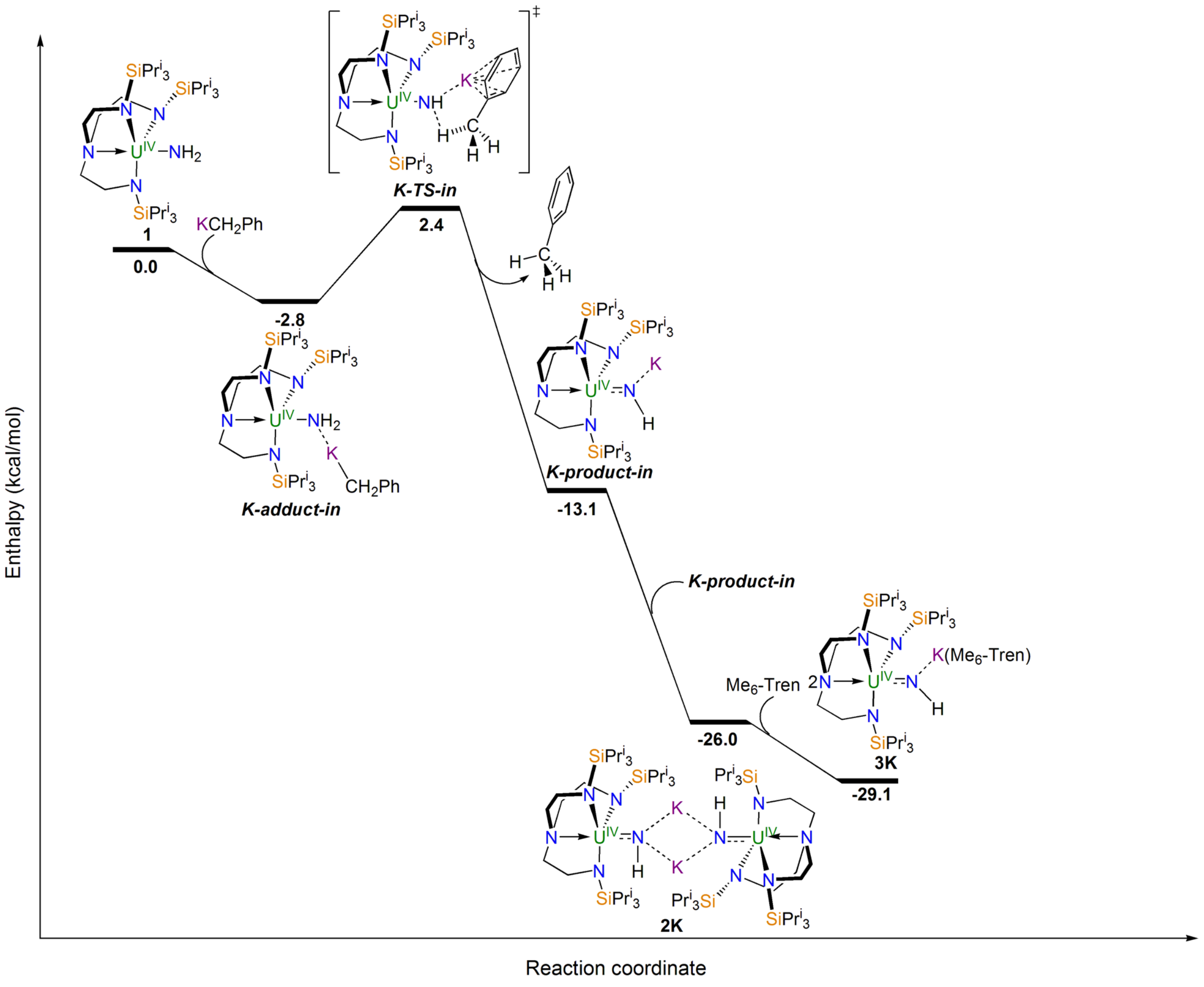

Supplementary Figure 41. Computed reaction profile for the conversion of 1 to 2K then 3K.

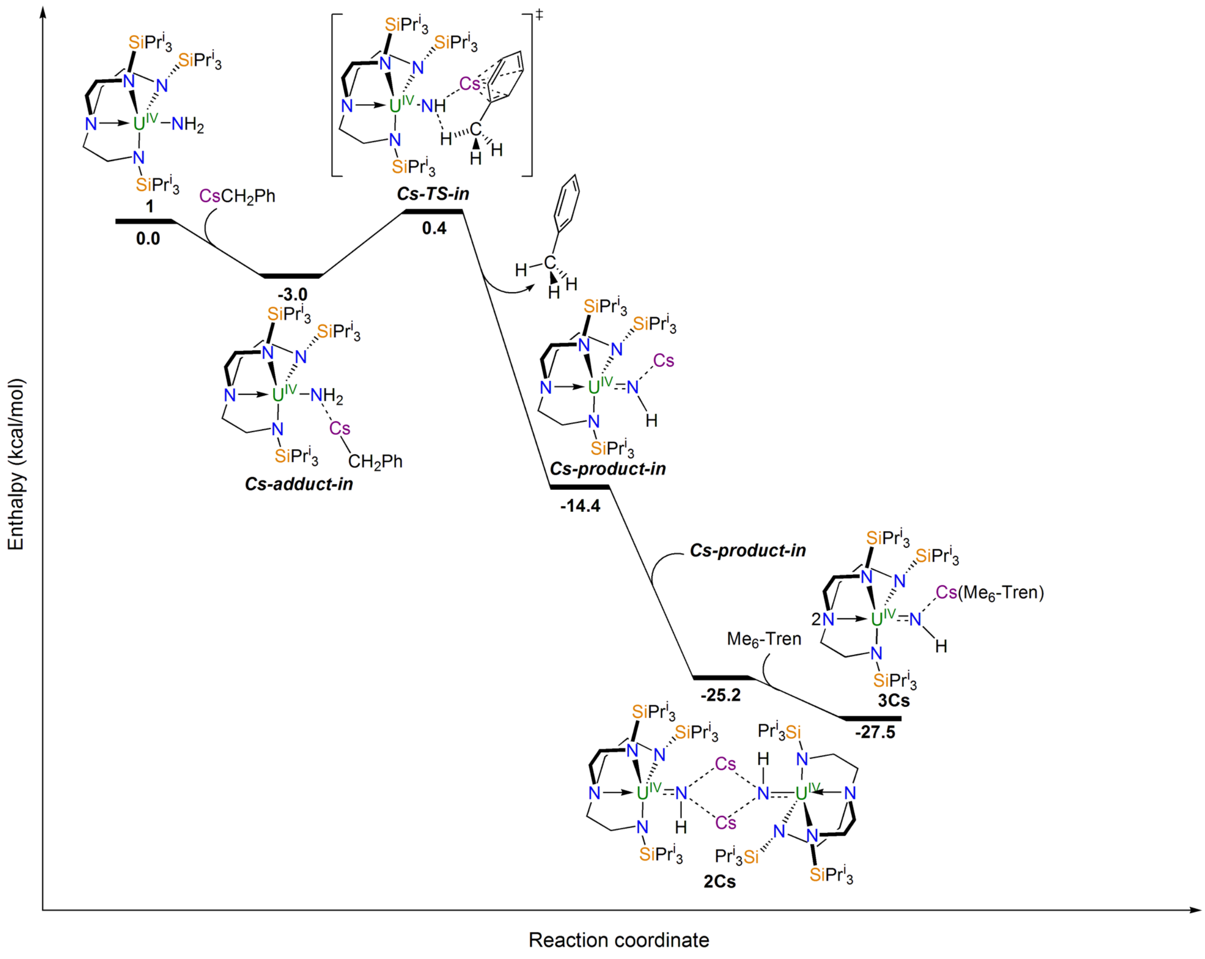

Supplementary Figure 42. Computed reaction profile for the conversion of 1 to 2Cs then 3Cs.

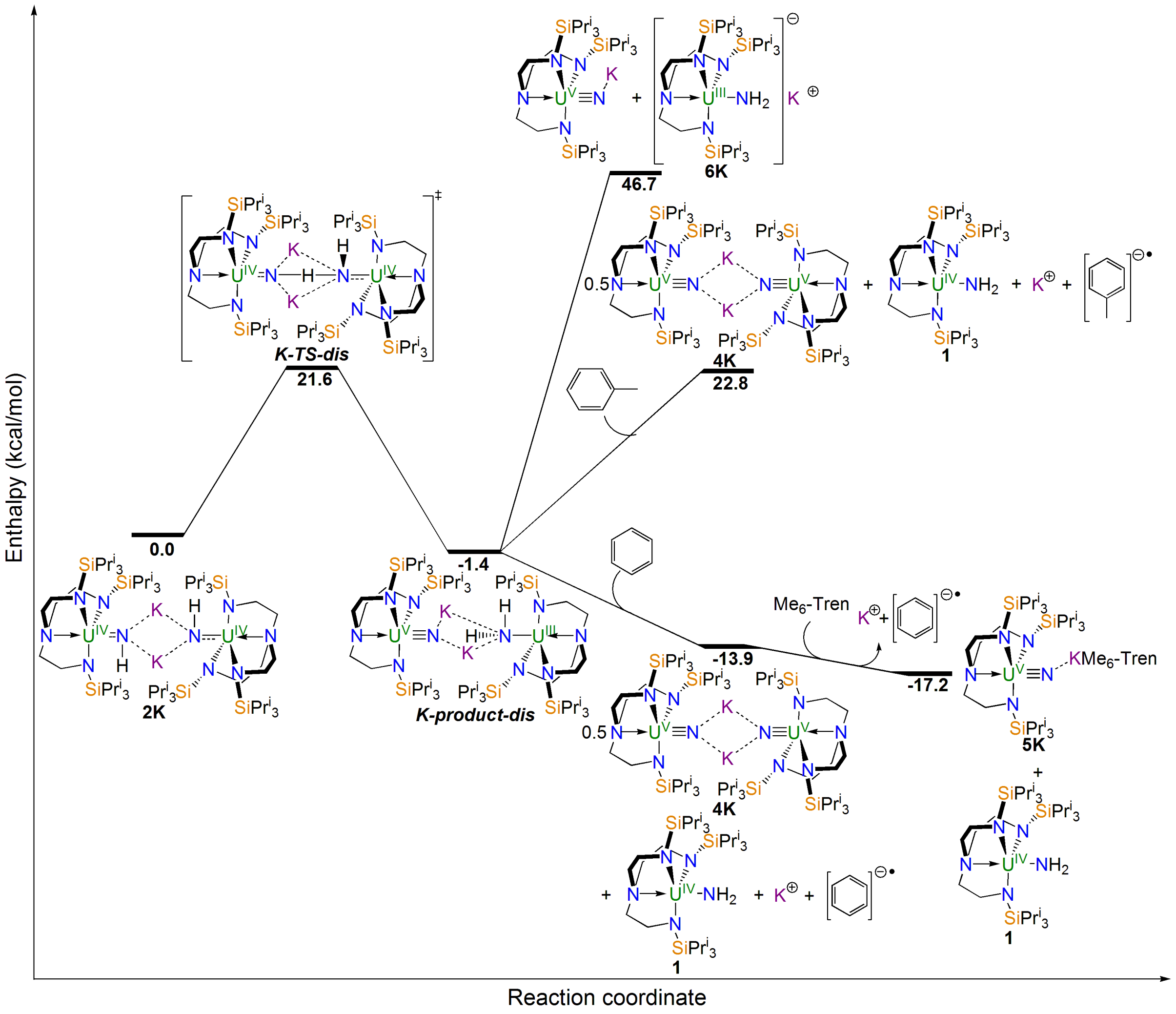

Supplementary Figure 43. Computed reaction profile for the disproportionation of 2K to 4K/1/K^{+}(C$_6$H$_6$)$^{-\bullet}$.

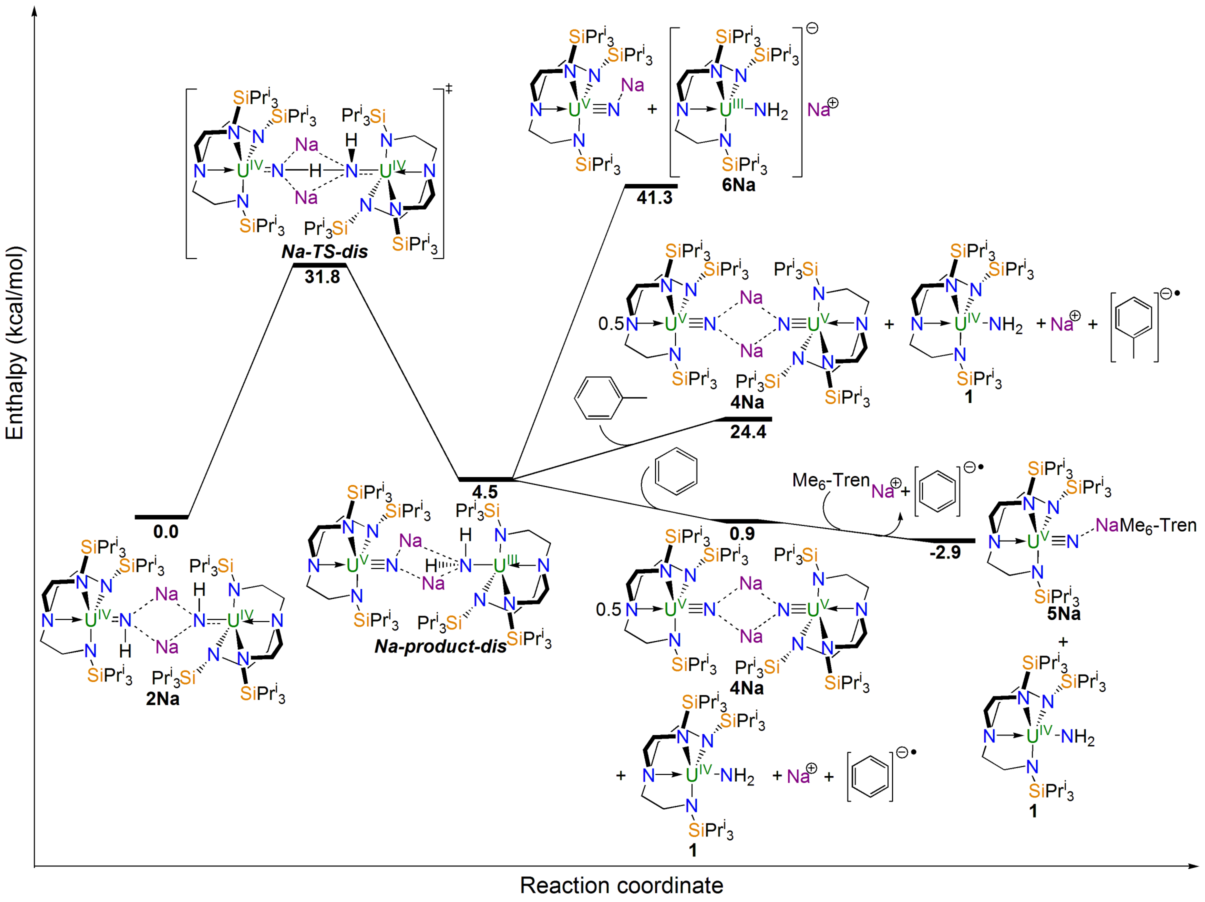

Supplementary Figure 44. Computed reaction profile for the disproportionation of 2Na to 4Na/1/Na(C_6H_6)$^{-\bullet}$.

Supplementary Tables

Supplementary Table 1. X-Ray crystallographic data for 3Li, 3Na and 3K

Compound	**$3Li\cdot(C_5H_{12})_{0.5}$**	**3Na**	**$3K\cdot(C_7H_8)_{0.5}$**
Formula	$C_{47.5}H_{112}LiN_9Si_3U$	$C_{45}H_{106}N_9NaSi_3U$	$C_{48.5}H_{110}KN_9Si_3U$
Mr	1138.70	1118.67	1180.85
Temperature/K	150(2)	150(2)	150(2)
Crystal system	monoclinic	monoclinic	monoclinic
Space group	$P2_1/n$	$P2_1/n$	$P2_1/n$
a/Å	14.8630(3)	13.4716(4)	14.1569(3)
b/Å	23.0214(5)	22.4968(8)	13.7150(3)
c/Å	18.6127(4)	19.4449(5)	32.0512(6)
α/°	90	90	90
β/°	96.983(2)	98.595(3)	99.408(2)
γ/°	90	90	90
V, $Å^3$	6321.4(2)	5826.9(3)	6139.4(2)
Z	4	4	4
ρ_{calc}, gcm^{-3}	1.1964	1.275	1.278
μ, mm^{-1}	2.660	2.890	2.807
F()	2348.9	2336.0	2468.0
Crystal size, mm^3	0.154 × 0.112 × 0.076	0.128 × 0.123 × 0.064	0.591 × 0.426 × 0.251
Radiation	Mo Kα (λ = 0.71073)	MoKα (λ = 0.71073)	MoKα (λ = 0.71073)
2Θ range, °	3.28 to 61.64	5.574 to 58.142	5.834 to 58.488
no. of reflns measd	44066	32365	48030
no. of unique reflns, R_{int}	15575, 0.0484	13567, 0.0601	14604, 0.0512
Data/restraints/ parameters	15575/180/677	13567/118/586	14604/39/585
Goodness-of-fit on F^2	1.049	1.003	1.118
R, R_w ($F^2 > 2\sigma(F^2)$)	0.0437, 0.0949	0.0466, 0.0584	0.0472, 0.0912
R, R_w (all data)	0.0791, 0.1052	0.0877, 0.0675	0.0652, 0.0975
max, min diff map, e $Å^{-3}$	2.47/−1.17	1.05/−0.78	1.34/−2.29

Supplementary Table 2. X-Ray crystallographic data for 3Rb, 3Cs and 5Li

Compound	**3Rb**	**3Cs·(C_7H_8)**	**5Li**
Formula	$C_{45}H_{106}N_9RbSi_3U$	$C_{52}H_{114}CsN_9Si_3U$	$C_{135}H_{315}Li_3N_{27}Si_9U_3$
Mr	1181.15	1320.73	3304.84
Temperature/K	150(2)	150(2)	150.0(2)
Crystal system	orthorhombic	triclinic	monoclinic
Space group	Pbca	P-1	$P2_1/c$
a/Å	22.6990(5)	11.9904(4)	31.5205(3)
b/Å	22.5154(4)	13.9169(3)	26.9533(4)
c/Å	23.0062(4)	20.0498(6)	20.4263(2)
α/°	90	97.210(2)	90
β/°	90	98.565(3)	102.1200(10)
γ/°	90	96.435(2)	90
V, $Å^3$	11757.9(4)	3252.93(16)	16967.0(3)
Z	8	2	4
$\rho_{calc,}$ gcm^{-3}	1.334	1.348	1.294
μ, mm^{-1}	3.680	3.139	2.970
F()	4880.0	1356.0	6900.0
Crystal size, mm^3	0.313 × 0.231 × 0.202	0.261 × 0.237 × 0.189	0.311 × 0.238 × 0.182
Radiation	MoKα (λ = 0.71073)	MoKα (λ = 0.71073)	MoKα (λ = 0.71073)
2Θ range, °	5.358 to 58.644	6.584 to 59.396	5.426 to 52.744
no. of reflns measd	106751	35223	87857
no. of unique reflns, R_{int}	14854, 0.0878	15420, 0.0511	34671, 0.0783
Data/restraints/ parameters	14854/0/556	15420/0/620	34671/6193/1869
Goodness-of-fit on F^2	1.047	1.025	1.049
R, R_w ($F^2 > 2\sigma(F^2)$)	0.0386, 0.0691	0.0444, 0.0818	0.0608, 0.1426
R, R_w (all data)	0.0734, 0.0805	0.0616, 0.0890	0.1074, 0.1772
max, min diff map, e $Å^{-3}$	1.11/−0.89	1.76/−1.35	2.14/−2.28

Supplementary Table 3. X-Ray crystallographic data for 5Na, 5K and 5Rb

Compound	**5Na**	**5K·(C$_6$H$_6$)$_{1.5}$**	**5Rb**
Formula	$C_{45}H_{105}N_9NaSi_3U$	$C_{54}H_{114}KN_9Si_3U$	$C_{45}H_{105}N_9RbSi_3U$
Mr	1117.66	1250.94	1180.14
Temperature/K	150(2)	150(2)	150(2)
Crystal system	orthorhombic	triclinic	triclinic
Space group	Pbca	P-1	P-1
a/Å	21.9311(3)	12.7238(3)	13.7571(3)
b/Å	22.6326(3)	13.5645(3)	20.2528(4)
c/Å	22.8392(3)	19.5348(5)	22.3210(6)
α/°	90	77.754(2)	88.8981(19)
β/°	90	83.279(2)	79.710(2)
γ/°	90	86.2985(19)	74.5748(19)
V, Å^3	11336.4(3)	3269.45(14)	5896.0(3)
Z	8	2	4
$\rho_{calc,}$ gcm^{-3}	1.310	1.271	1.329
μ, mm^{-1}	2.971	2.639	3.670
F()	4664.0	1308.0	2436.0
Crystal size, mm^3	0.269 × 0.246 × 0.215	0.279 × 0.203 × 0.168	0.272 × 0.24 × 0.207
Radiation	MoKα (λ = 0.71073)	MoKα (λ = 0.71073)	MoKα (λ = 0.71073)
2Θ range, °	5.398 to 58.632	5.636 to 52.744	5.302 to 52.744
no. of reflns measd	118689	30841	56660
no. of unique reflns, R_{int}	14421, 0.0929	13313, 0.0809	23901, 0.0669
Data/restraints/ parameters	14421/0/556	13313/407/592	23901/565/1216
Goodness-of-fit on F^2	1.116	1.067	1.053
R, R_w ($F^2 > 2\sigma(F^2)$)	0.0394, 0.0720	0.0560, 0.1183	0.0474, 0.0955
R, R_w (all data)	0.0669, 0.0841	0.0713, 0.1310	0.0685, 0.1077
max, min diff map, e Å^{-3}	1.88/−1.50	2.29/−2.00	2.21/−2.18

Supplementary Table 4. X-Ray crystallographic data for 5Cs

Compound	**$5Cs\cdot(C_7H_8)_{0.5}$**
Formula	$C_{48.5}H_{109}CsN_9Si_3U$
Mr	1273.65
Temperature/K	150(2)
Crystal system	monoclinic
Space group	$P2_1/n$
a/Å	14.4235(6)
b/Å	13.4600(5)
c/Å	32.3488(13)
α/°	90
β/°	96.359(4)
γ/°	90
V, $Å^3$	6241.6(4)
Z	4
ρ_{calc}, gcm^{-3}	1.355
μ, mm^{-1}	3.269
F()	2608.0
Crystal size, mm^3	0.298 × 0.237 × 0.15
Radiation	MoKα (λ = 0.71073)
2Θ range, °	6.652 to 51.362
no. of reflns measd	11841
no. of unique reflns, R_{int}	11841, NA
Data/restraints/ parameters	11841/775/736
Goodness-of-fit on F^2	1.089
R, R_w ($F^2 > 2\sigma(F^2)$)	0.0522, 0.0905
R, R_w (all data)	0.0742, 0.0981
max, min diff map, e $Å^{-3}$	1.12/−1.29

Supplementary Table 5. Selected bond lengths (Å) and angles (o) for 3M

Entry	**3Li**	**3Na**	**3K**	**3Rb**	**3Cs**
U1-N5	2.070(4)	1.979(4)	2.006(4)	1.996(3)	1.982(3)
U1-N4	2.718(3)	2.752(3)	2.692(4)	2.703(3)	2.700(3)
M1-N5	2.019(9)	2.397(4)	2.748(4)	2.903(3)	3.057(4)
N4-U1-N5	178.08(13)	179.08(14)	172.08(15)	178.03(11)	177.68(13)
U1-N5-M1	159.7(3)	175.8(2)	144.9(2)	143.93(16)	141.01(19)
U1-N5-H5	100.1	92.1	107.6	108.0	109.5

Supplementary Table 6. Selected bond lengths (Å) and angles (o) for 5M

Entry	**5Li**	**5Na**	**5K**	**5Rb**	**5Cs**
U1-N5	1.814(6)	1.823(3)	1.809(5)	1.792(4)	1.816(6)
U1-N4	2.698(6)	2.746(3)	2.685(5)	2.662(4)	2.658(5)
M1-N5	2.009(14)	2.403(3)	2.654(5)	2.736(4)	2.918(5)
N4-U1-N5	179.5(3)	179.49(12)	177.9(2)	179.59(18)	179.1(2)
U1-N5-M1	174.7(5)	177.66(18)	168.5(3)	168.3(2)	155.9(3)

For **5Li**, there are three independent molecules in the asymmetric unit, the listed values are extracted from the molecule with more certain metrical parameters as the others are highly disordered; For **5Rb**, there are two independent molecules in the unit cell; the listed values are extracted from a better one without any disordered components.

Supplementary Table 7. Magnetic moments (SQUID and Evan's method) for 3M and 5M

Entry	**μ_{eff} (μB, 2 K)**	**μ_{eff} (μB, 298 K)**	**μ_{eff} (μB, Evans' Method, 298K)**
**3Li**	0.86	3.08	2.3
**3Na**	1.39	3.02	-
**3K**	1.38	2.91	-
**3Rb**	1.19	3.12	-
**3Cs**	1.68	2.75	2.9
**5Li**	1.90	2.13	1.6
**5Na**	1.97	2.24	-
**5K**	1.85	2.34	-
**5Rb**	1.81	2.35	-
**5Cs**	1.83	2.42	2.3

The solution magnetic moments (Evan's method) could not be obtained for **3Na/K/Rb** and **5Na/K/Rb** as these complexes are only partially soluble in aromatic solvent (benzene, and toluene) once isolated, and they decompose in polar solvents (THF and pyridine). The solid state magnetic moments for **3M** and **5M** measured by SQUID magnetometry are characteristic of uranium(IV) and (V) magnetism, respectively, and they are internally consistent in each series. The discrepancies in solid and solution magnetic moments for **3Li** and **5Li** might be due to the structural change in different states since all the NMe_2 groups within Me_6-Tren ligand might be able to coordinate to the Li cation in solution, giving a more symmetric geometry, this could be suggested by their ^{1}H, ^{29}Si{^{1}H}, and ^{7}Li{^{1}H} NMR spectra, although the weighing errors for solution magnetic moments measurements cannot be fully ruled out.

Supplementary Table 8. Computed data for the conversion of 1 to 2Li then 3Li

Compound		Spin density	Atomic charge	Spin	multiplicity	U-N length (Å)	Contamination	TS imag (cm^{-1})
**1**	U	2.161	1.731	1	3	2.237	2.1	
	N	-0.046	-0.947					
**Li-TS-in**	U	2.174	1.742	1	3	2.212	2.1	-931.
	N	-0.034	-1.035					
**Li-product-in**	U	2.175	1.732	1	3	2.184	2.1	
	N	-0.033	-1.036					
**2Li**	U	2.169	1.645	2	5	2.189	6.3	
	N	-0.058	-0.991					
**3Li**	U	2.183	1.546	1	3	2.101	2.1	
	N	-0.029	-1.019					

Supplementary Table 9. Computed data for the conversion of 1 to 2Na then 3Na

Compound		Spin density	Atomic charge	Spin	multiplicity	U-N length (Å)	Contamination	TS imag (cm^{-1})
**1**	U	2.161	1.731	1	3	2.237	2.1	
	N	-0.046	-0.947					
**Na-TS-in**	U	2.141	1.743	1	3	2.230	2.1	-884.
	N	-0.055	-1.025					
**Na-product-in**	U	2.170	1.730	1	3	2.198	2.1	
	N	-0.031	-1.012					
**2Na**	U	2.165	1.612	2	5	2.193	6.3	
	N	-0.089	-0.968					
**3Na**	U	2.189	1.548	1	3	2.138	2.1	
	N	-0.064	-1.085					

Supplementary Table 10. Computed data for the conversion of 1 to 2K then 3K

Compound		Spin density	Atomic charge	Spin	multiplicity	U-N length (Å)	Contamination	TS imag (cm^{-1})
**1**	U	2.161	1.731	1	3	2.237	2.1	
	N	-0.046	-0.947					
**K-TS-in**	U	2.144	1.659	1	3	2.238	2.1	-1389.94
	N	-0.033	-0.989					
**K-product-in**	U	2.187	1.483	1	3	2.038	2.1	
	N	-0.025	-1.011					
**2K**	U	2.163	1.258	2	5	2.046	6.3	
	N	-0.081	-0.899					
**3K**	U	2.192	1.474	1	3	2.055	2.1	
	N	-0.101	-0.877					

Supplementary Table 11. Computed data for the conversion of 1 to 2Cs then 3Cs

Compound		Spin density	Atomic charge	Spin	multiplicity	U-N length (Å)	Contamination	TS imag (cm^{-1})
**1**	U	2.161	1.731	1	3	2.237	2.1	
	N	-0.046	-0.947					
**Cs-adduct-in**	U	2.165	1.673	1	3	2.241	2.1	
	N	-0.046	-0.962					
**Cs-TS-in**	U	2.183	1.583	1	3	2.152	2.1	-1329.65
	N	-0.072	-0.943					
**Cs-product-in**	U	2.191	1.362	1	3	2.042	2.1	
	N	-0.096	-0.901					
**2Cs**	U	2.188	1.451	2	5	2.058	6.3	
	N	-0.098	-0.933					
**3Cs**	U	2.193	1.346	1	3	2.010	2.1	
	N	-0.099	-0.874					

Supplementary Table 12. Computed data for the conversion of 1 to 4Li/6Li then 4Li/1/LiC$_6$H$_6$ and 5Li

Compound	Spin density	Atomic charge	Spin/ multiplicity		U-N length (Å)	Contamination	TS imag (cm^{-1})
**Li-TS-dis**	U1=2.167 U2=2.192	U1=1.778 U2=1.492	2	5	U1-N1=2.262 U2-N2=2.067	6.5	-1480.545
	N1=-0.055 N2=-0.099	N1=-1.023 N2=-1.008					
**Li-product-dis**	U1=2.165 U2=2.221	U1=1.758 U2=1.377	2	5	U1-N1=2.374 U2-N2=1.974	6.2	
	N1=-0.027 N2=-0.124	N1=-0.970 N2=-0.859					
**4Li**	U1=1.197 U2=1.196	U1=1.554 U2=1.539	1	3	U1-N1=1.865 U2-N2=1.865	2.7	
	N1=-0.111 N2=-0.111	N1=-0.776 N2=-0.779					
**5Li**	U= 1.198	U= 1.191	1/2	2	1.803	0.7502	
	N=-0.124	N= -0.628					
**6Li**	U=1.187	U=1.424	3/2	4	1.8305	0.7502	
	N=-1.015	N=-0.016					

Supplementary Table 13. Computed data for the conversion of 1 to 4Na/6Na then 4Na/1/NaC$_6$H$_6$ and 5Na

Compound	Spin density	Atomic charge	Spin/ multiplicity		U-N length (Å)	Contamination	TS imag (cm^{-1})
**Na-TS-dis**	U1=2.178 U2=2.201	U1=1.987 U2=1.786	2	5	U1-N1=2.291 U2-N2=2.090	6.5	-1350.545
	N1=-0.086 N2=-0.102	N1=-1.052 N2=-1.093					
**Na-product-dis**	U1=2.195 U2=2.266	U1=1.798 U2=1.405	2	5	U1-N1=2.401 U2-N2=2.005	6.2	
	N1=-0.087 N2=-0.164	N1=-0.984 N2=-0.891					
**4Na**	U1=1.218 U2=1.221	U1=1.532 U2=1.576	1	3	U1-N1=1.903 U2-N2=1.898	2.7	
	N1=-0.113 N2=-0.123	N1=-0.806 N2=-0.808					
**5Na**	U= 1.198	U= 1.191	1/2	2	1.803	0.7502	
	N=-0.124	N= -0.628					
**6Na**	U=1.213	U=1.489	3/2	4	1.835	0.7502	
	N=-1.059	N=-0.092					

Supplementary Table 14. Computed data for the conversion of 1 to 4K/6K then 4K/1/KC$_6$H$_6$ and 5K

Compound	Spin density	Atomic charge	Spin/ multiplicity		U-N length (Å)	Contamination	TS imag (cm^{-1})
**K-TS-dis**	U1= 2.207<br>U2= 2.176	U1= 1.362<br>U2= 1.683	2	5	U1-N1= 1.99531<br>U2-N2= 2.18584	6.5	-1394.41
	N1= -0.024<br>N2= -0.028	N1= -1.01<br>N2= -1.02					
**K-product-dis**	U1= 1.192<br>U2= 1.194	U1= 1.491<br>U2= 1.478	2	5	U1-N1= 1.82130<br>U2-N2= 1.82214	6.2	
	N1= -0.124<br>N2= -0.014	N1= -0.731<br>N2= -1.016					
**4K**	U1= 1.170<br>U2=1.181	U1= 1.206<br>U2= 1.209	1	3	U1-N1= 1.814<br>U2-N2= 1.802	2.6	
	N1=-0.119<br>N2= -0.123	N1= -0.614<br>N2= -0.686					
**5K**	U= 1.198	U= 1.191	1/2	2	1.803	0.7502	
	N=-0.124	N= -0.628					
**6K**	U= 1.204	U= 1.355	3/2	4	1.813	3.7500	
	N= -0.019	N= -1.010					

Supplementary Table 15. Computed data for the conversion of 1 to 4Cs/6Cs then 4Cs/1/CsC$_6$H$_6$ and 5Cs

Compound	Spin density	Atomic charge	Spin/ multiplicity		U-N length (Å)	Contamination	TS imag (cm^{-1})
**Cs-TS-dis**	U1=2.228<br>U2=2.180	U1= 1.085<br>U2= 1.595	2	5	U1-N1=1.960<br>U2-N2=2.196	6.5	-1380.76
	N1=-0.156<br>N2=-0.074	N1= -0.839<br>N2= -0.482					
**Cs-product-dis**	U1= 3.118<br>U2= 1.190	U1= 1.293<br>U2= 1.189	2	5	U1-N1=1.810<br>U2-N2=2.477	6.2	
	N1= -0.129<br>N2= -0.027	N1= -0.655<br>N2= -0.956					
**4Cs**	U1= 1.188<br>U2=1.190	U1= 1.206<br>U2= 1.209	1	3	U1-N1=1.809<br>U2-N2=1.809	2.6	
	N1=-0.129<br>N2= -0.129	N1= -0.660<br>N2= -0.659					
**5Cs**	U= 1.198	U= 1.191	1/2	2	1.803	0.7502	
	N=-0.124	N= -0.628					
**6Cs**	U=3.123	U=1.293	3/2	4	2.330	3.7500	
	N=-0.039	N= -0.948					

Supplementary Table 16. Final coordinates and energy for 1

Energy: -2064.946666 a.u

U	0.251610	0.067558	0.033915
Si	-0.708707	3.328312	-1.449401
Si	0.438828	-0.458914	3.745711
Si	0.818675	-2.817214	-2.288517
N	-1.924756	-0.425569	0.159227
N	0.510329	2.044144	-1.092263
N	2.838789	0.705677	-0.038996
N	1.046285	0.184902	2.182622
N	1.388061	-1.553741	-1.135688
C	-2.134976	0.842526	3.794413
C	-0.245879	2.204937	4.710860
C	-0.813507	0.788439	4.569147
C	0.429186	-3.257691	3.001265
C	-1.336906	-2.563396	4.637016
C	-0.512183	-2.123387	3.420755
C	-0.852004	6.175517	-2.007500
C	1.072248	5.504643	-0.529641
C	0.154807	5.066257	-1.675484
C	-0.733655	3.050242	-4.343405
C	-2.406618	1.655698	-3.075945
C	-1.650418	2.985388	-3.118133
C	1.192965	-5.163149	-0.608343
C	-1.057193	-4.044255	-0.454019
C	0.116194	-4.387390	-1.373733
C	-0.134439	-1.066356	-4.395151
C	-1.568065	-3.087443	-3.968655
C	-0.630699	-2.053380	-3.334191
C	-3.370196	3.950403	-0.400673
C	-1.507877	3.906150	1.285421
C	-2.028895	3.307584	-0.025288
C	1.834599	2.218422	-1.712133
C	2.936187	2.034097	-0.674273
C	2.170817	1.132558	2.294424
C	3.306467	0.725534	1.361920
C	3.510873	-0.341520	-0.833166
C	2.786121	-1.674965	-0.685879
C	3.153197	-1.423339	4.413536
C	3.131754	-2.439526	-4.119733
C	2.137236	0.100683	6.132105
C	1.841474	-0.925935	5.033475
C	2.281309	-3.490024	-3.398739
C	1.804209	-4.553232	-4.397278
H	-2.562687	-0.329237	-0.629311
H	-2.473093	-0.769347	0.944408
H	2.824548	-0.322595	6.878173
H	1.235040	0.413519	6.667531
H	2.618811	1.002680	5.736477
H	-1.018945	0.394927	5.576427
H	0.668736	2.242361	5.308980
H	1.380199	-1.790670	5.536827
H	-0.975025	2.873137	5.189749
H	-0.694440	-2.774970	5.501255
H	3.775830	-1.915645	5.173327
H	-2.068586	-1.813450	4.952892
H	-2.836926	1.547113	4.261505
H	-1.889294	-3.487777	4.420875
H	-0.016209	2.634728	3.729893
H	3.746711	-0.595173	4.011338
H	-2.639499	-0.127851	3.743596
H	2.999003	-2.140612	3.602377
H	1.102273	-3.534484	3.821355
H	2.578626	1.194611	3.314045
H	-1.976514	1.182330	2.764018
H	-1.209330	-1.935660	2.590514
H	-0.133812	-4.160827	2.732779
H	-2.196190	3.699125	2.114748
H	1.861472	2.162847	2.051036
H	1.050760	-2.988840	2.140941
H	3.619515	-0.287210	1.630181
H	-0.526577	3.507513	1.563345
H	4.182472	1.387434	1.466925
H	-1.412376	4.995893	1.218357
H	2.849964	-2.001673	0.366033
H	-4.088567	3.850194	0.424491
H	-0.740829	-3.386138	0.361353
H	1.650798	-4.549362	0.176423
H	-2.218501	2.236476	0.151183
H	0.502675	5.768038	0.367322
H	2.799617	2.788115	0.106429
H	-3.268758	5.022523	-0.604484
H	-1.480474	-4.949518	0.003638
H	4.573826	-0.418529	-0.549584
H	0.767124	-6.047205	-0.113587
H	1.785668	4.727109	-0.241680
H	3.358685	-2.424787	-1.250134
H	-1.869311	-3.530982	-0.979118
H	-3.829349	3.493597	-1.282976
H	1.652852	6.393677	-0.812620
H	1.998073	-5.517348	-1.261403
H	3.938486	2.182773	-1.110063
H	3.467255	-0.044076	-1.884704
H	-1.495590	6.408310	-1.151385
H	-3.135725	1.616323	-2.259092
H	-0.263614	-5.041802	-2.172064
H	1.975797	3.210820	-2.163796
H	-1.226685	-1.490980	-2.597853
H	1.992826	1.502766	-2.535452

H 0.786593 4.939492 -2.567262
H 2.943114 -4.004129 -2.685499
H -1.715070 0.815598 -2.944632
H -0.331565 7.105335 -2.275336
H -1.503209 5.917696 -2.849559
H -2.392539 3.792249 -3.209367
H 3.514172 -1.668935 -3.444528
H -2.002005 -3.772317 -3.234155
H 0.537662 -0.310479 -3.975426
H -2.956269 1.477100 -4.010377
H 1.227420 -5.354821 -3.923885
H 0.053269 2.288599 -4.297514
H -2.402858 -2.588667 -4.479801
H 4.752000 -2.910502 -4.600372
H -0.971469 -0.539590 -4.871018
H -0.243170 4.024459 -4.447220
H 2.570923 -1.928606 -4.908713
H -1.054219 -3.696619 -4.720670
H 0.407761 -1.585820 -5.193123
H 2.660465 -5.024863 -4.898760
H 1.178490 -4.116105 -5.184054
H -1.297456 2.873268 -5.269893

Supplementary Table 17. Final coordinates and energy for Li-TS

Energy: -2230.117145 a.u

U 3.455249 2.205081 4.816704
Si 1.726815 -0.028517 2.344035
Si 2.005971 3.030625 8.216637
Si 6.800397 3.720319 3.990494
N 2.068246 3.108898 6.419002
N 5.036130 3.837409 4.305125
N 2.232984 4.326845 3.760469
N 2.350010 1.563936 2.896621
N 4.388149 0.529260 5.923015
C -0.946366 1.162081 1.886605
C 1.163835 -2.617404 3.511576
C 3.517026 -0.060824 0.038290
C 7.576058 2.749710 6.613377
C 1.609363 5.958578 8.518818
C 3.956377 2.255054 10.267920
C 1.576232 0.158367 8.422799
C 6.700248 5.280289 1.493149
C 6.577476 1.723688 1.880660
C 0.295629 -0.643443 4.785641
C 1.432153 -1.156721 3.894663
C 4.218049 -1.439267 2.011503
C 3.022170 -0.930150 1.199503
C 0.028701 -0.010057 -0.108798
C -0.006724 0.043472 1.422084
C 4.623528 4.160826 8.771789
C 3.833283 2.851432 8.860663
C -0.496771 1.575570 8.526227
C 0.993700 1.499181 8.874456
C 0.997998 4.531217 10.495389
C 1.088905 4.588821 8.964599
C 8.940907 5.186082 2.636398
C 7.420734 5.174038 2.840209
C 8.640021 1.553588 3.299553
C 7.158048 1.948611 3.281204
C 1.351199 4.858332 4.818940
C 0.909252 3.744106 5.763411
C 7.692391 5.247809 6.302260
C 7.859365 3.884784 5.625022
C 4.473362 5.198182 4.316793
C 3.279234 5.292167 3.373956
C 1.467838 3.843380 2.592980
C 2.180638 2.666521 1.935283
C 5.377299 -2.611816 6.576481
C 7.086970 -1.235200 7.724879
C 6.344538 -1.447134 6.393653
C 7.354401 -1.785027 5.302978
Li 6.155848 0.652938 6.782225
H 8.314974 5.326682 7.204095
H 1.978929 4.663253 10.966732
H 0.353955 5.336184 10.875523
H 4.275837 4.883840 9.517926
H 3.513643 2.909847 11.026696
H 2.599942 6.172582 8.932037
H 7.979309 6.071959 5.640279
H 5.691343 3.997481 8.970415
H 0.939175 6.758348 8.863254
H 5.012319 2.123129 10.543527
H 6.655188 5.420428 6.610552
H 0.581520 3.586725 10.860574
H 8.162624 2.851714 7.536193
H 4.534493 4.632554 7.787956
H 3.474157 1.277410 10.360346
H 1.687089 6.045441 7.431674
H 8.908719 3.786992 5.312364
H 9.495754 5.160355 3.580223
H 9.250760 6.096740 2.105610
H 6.514224 2.763633 6.903047
H 0.059506 4.499511 8.587293
H 7.177201 6.084536 3.408377
H 4.151656 5.495095 5.328463
H 4.283298 2.135158 8.152964
H 5.191476 5.970733 4.006229
H 1.083901 1.557000 9.969837
H 7.849791 1.781111 6.169228

H 9.278992 4.337860 2.031197
H 1.920016 5.592854 5.395312
H 2.866748 6.314700 3.335292
H -0.972032 2.485736 8.907908
H 6.961349 6.217366 0.982656
H 9.104260 1.663960 4.284868
H 2.623613 0.032432 8.718971
H 0.196489 4.179750 6.478107
H 5.611791 5.256373 1.598560
H -1.044938 0.723898 8.952093
H 0.488065 5.380434 4.373960
H 1.017223 -0.684064 8.852988
H 9.225628 2.159012 2.599155
H 3.617425 5.032753 2.366548
H 6.977820 4.462833 0.820109
H 6.631136 1.262221 3.966782
H -0.658750 1.547765 7.442085
H 1.523046 0.051448 7.333947
H 8.765256 0.506522 2.995758
H 0.317731 3.012047 5.187226
H 7.135726 2.285716 1.123732
H 1.289055 4.667492 1.881690
H 5.528866 2.030293 1.808882
H 0.494776 3.494587 2.949635
H 0.439272 0.399473 5.090285
H 3.150223 3.011575 1.539049
H 6.635840 0.665760 1.597562
H 0.205715 -1.246220 5.698776
H 2.364599 -1.126644 4.477572
H 4.714155 -0.620118 2.544239
H 1.596522 2.377594 1.048549
H -0.671409 -0.703018 4.272091
H -0.984373 1.261925 2.975301
H 3.937450 -2.185881 2.760263
H -0.647729 2.132900 1.476547
H 4.045251 0.825059 0.406661
H 1.049430 -3.240133 4.408856
H 4.970020 -1.902819 1.357943
H 1.967108 -3.057969 2.913318
H -1.971940 0.975553 1.538911
H 0.234770 -2.720299 2.936612
H 4.225744 -0.616626 -0.591443
H -0.457350 -0.905756 1.752169
H 2.708575 0.284661 -0.612359
H 2.498479 -1.801554 0.777140
H 0.439055 0.908571 -0.544502
H 0.621292 -0.850099 -0.484720
H -0.986605 -0.125193 -0.514
H 3.703880 0.192396 6.611203
H 5.184888 -0.383280 5.925498
H 7.626273 -2.138429 8.072232
H 7.868491 -0.452589 7.676654
H 6.407599 -0.969207 8.553694
H 7.915096 -2.721165 5.503743
H 6.868213 -1.919438 4.327393
H 8.114042 -1.169000 5.171279
H 5.885781 -3.552944 6.865579
H 4.628693 -2.411475 7.357084
H 4.821278 -2.828527 5.654815

Supplementary Table 18. Final coordinates and energy for Li-Product

Energy: -2230.139339 a.U
U -0.187698 0.109422 -0.085161
Si -1.916583 -2.122063 -2.558271
Si -1.637416 0.935902 3.312588
Si 3.155446 1.626071 -0.912042
N -1.577859 1.016212 1.519006
N 1.396443 1.745353 -0.598681
N -1.411400 2.233510 -1.142802
N -1.296364 -0.533263 -2.009932
N 0.736334 -1.537368 1.014119
C -4.589823 -0.932720 -3.016123
C -2.479765 -4.712115 -1.390957
C -0.126492 -2.155635 -4.864284
C 3.932564 0.655284 1.711069
C -2.034285 3.863842 3.616221
C 0.312973 0.160555 5.365293
C -2.067644 -1.936407 3.520267
C 3.056762 3.185614 -3.409435
C 2.934058 -0.370920 -3.021970
C -3.347949 -2.738166 -0.117178
C -2.211498 -3.251692 -1.008191
C 0.574683 -3.533899 -2.891375
C -0.621659 -3.025452 -3.703463
C -3.615079 -2.104780 -5.011528
C -3.650421 -2.051773 -3.480775
C 0.979882 2.066079 3.869292
C 0.189606 0.756878 3.958482
C -4.140239 -0.519105 3.623638
C -2.649830 -0.595479 3.972422
C -2.645697 2.436820 5.592792
C -2.554670 2.494062 4.062385
C 5.297457 3.091660 -2.266465
C 3.777759 3.079216 -2.062491
C 4.996492 -0.541255 -1.603180
C 3.514995 -0.146008 -1.621542
C -2.292349 2.763676 -0.084042
C -2.734237 1.649538 0.861593
C 4.048838 3.152933 1.399631

C 4.216756 1.789979 0.722059
C 0.830473 3.103321 -0.586208
C -0.364625 3.197385 -1.528610
C -2.175503 1.748933 -2.309516
C -1.463077 0.571031 -2.967290
C 1.729885 -4.715870 1.675306
C 3.437621 -3.319770 2.821642
C 2.689943 -3.550895 1.485753
C 3.709097 -3.875709 0.395313
Li 2.499221 -1.421476 1.876724
H 4.671346 3.232571 2.301676
H -1.664505 2.568574 6.064039
H -3.289551 3.241686 5.974147
H 0.633029 2.790020 4.615267
H -0.129265 0.814636 6.125233
H -1.043457 4.077793 4.029178
H 4.335666 3.976745 0.737203
H 2.048308 1.903378 4.067713
H -2.704223 4.664198 3.960504
H 1.369221 0.028395 5.641972
H 3.011442 3.325064 1.707053
H -3.061851 1.492011 5.957843
H 4.519740 0.757093 2.633937
H 0.890543 2.536440 2.884945
H -0.169859 -0.817032 5.457518
H -1.956347 3.949826 2.529066
H 5.266817 1.692826 0.411133
H 5.852334 3.065733 -1.322518
H 5.608269 4.002074 -2.797430
H 2.870057 0.668855 1.997544
H -3.583989 2.405063 3.684599
H 3.533423 3.989499 -1.494346
H 0.507347 3.400201 0.425522
H 0.633891 0.041243 3.246104
H 1.546216 3.879065 -0.896755
H -2.560839 -0.545343 5.068179
H 4.207813 -0.312883 1.264955
H 5.635532 2.243259 -2.871734
H -1.723315 3.497987 0.492631
H -0.776371 4.220842 -1.567690
H -4.615504 0.391258 4.005127
H 3.317724 4.122766 -3.920173
H 5.461600 -0.429596 -0.617939
H -1.019773 -2.060834 3.815064
H -3.449732 2.087078 1.573385
H 1.968477 3.161198 -3.303419
H -4.689052 -1.370787 4.049312
H -3.155966 3.286393 -0.528782
H -2.627245 -2.778669 3.951180
H 5.582901 0.063702 -2.303270
H -0.026250 2.937766 -2.536040
H 3.334098 2.368096 -4.082659
H 2.983698 -0.829173 -0.936778
H -4.301441 -0.546642 2.539461
H -2.117721 -2.041576 2.431572
H 5.123316 -1.588349 -1.908377
H -3.325570 0.917712 0.283721
H 3.492129 0.191080 -3.779201
H -2.355042 2.572975 -3.021703
H 1.885564 -0.064598 -3.091963
H -3.148444 1.399675 -1.952719
H -3.202401 -1.695802 0.187315
H -0.493038 0.917962 -3.362795
H 2.991886 -1.428970 -3.305679
H -3.437856 -3.340902 0.796264
H -1.278943 -3.217563 -0.425616
H 1.068196 -2.714012 -2.357537
H -2.047173 0.286251 -3.856136
H -4.315338 -2.797854 -0.630472
H -4.627091 -0.832695 -1.927481
H 0.292622 -4.280272 -2.142544
H -4.290797 0.038078 -3.425852
H 0.400891 -1.269634 -4.495243
H -2.594751 -5.335645 -0.493846
H 1.326623 -3.997990 -3.545402
H -1.676701 -5.152849 -1.989847
H -5.615730 -1.118945 -3.363707
H -3.408830 -4.815547 -1.966064
H 0.582446 -2.710933 -5.494700
H -4.102404 -3.722 -3.151332
H -0.935111 -1.810016 -5.514681
H -1.143884 -3.897417 -4.126854
H -3.204424 -1.186115 -5.446913
H -3.022309 -2.944797 -5.387589
H -4.630280 -2.219747 -5.417630
H 0.071306 -1.935734 1.691774
H 1.947238 -2.778668 1.138186
H 3.962388 -4.231318 3.133772
H 4.221822 -2.544836 2.769513
H 2.760420 -3.062420 3.649824
H 4.255226 -4.802955 0.618563
H 3.221635 -4.012252 -0.576052
H 4.464369 -3.088945 0.270465
H 2.249035 -5.640155 1.963019
H 0.986662 -4.501569 2.452619
H 1.177978 -4.921162 0.752246

Supplementary Table 19. Final coordinates and energy for 2Li
Energy: -4143.819060 a.u

U -3.696324 -1.275622 0.141807
U -10.490567 -4.012114 -0.136672
Si -3.468768 -1.022917 3.920457
Si -2.816287 -4.549225 -1.607058
Si -4.366577 1.811621 -2.022508
Si -9.642461 -7.605960 0.749770
Si -11.179952 -1.490507 2.666057
Si -11.151155 -3.014463 -3.785631
N -2.916495 -0.691369 2.251165
N -2.483769 -3.146963 -0.540814
N -1.063745 -0.681801 0.005292
N -3.361440 0.470920 -1.378301
N -5.824040 -1.669889 0.336004
N -8.359380 -3.627788 -0.331362
N -10.716092 -6.305083 0.154288
N -11.594307 -3.305968 -2.071305
N -13.112462 -4.648953 0.036640
N -11.447702 -3.006015 1.748134
C -1.520193 -0.444828 6.107396
C -2.044737 -1.540147 5.172651
C -4.699675 -2.523789 3.887736
C -5.349919 -2.770988 5.253646
C -0.870347 -2.286591 4.531217
C -4.440538 0.487496 4.684331
C -3.697554 1.824557 4.582159
C -4.671495 -4.500219 -2.180629
C -5.036624 -5.713584 -3.043647
C -5.831995 0.632310 4.061743
C -1.763497 0.226306 2.188013
C -0.623876 -0.379408 1.379047
C -0.375226 -1.867222 -0.533494
C -1.082386 -3.134806 -0.079181
C -0.930763 0.483176 -0.884758
C -2.006336 0.461349 -1.963494
C -4.793131 1.606600 -3.918611
C -5.469664 0.266749 -4.213927
C -3.410330 3.515678 -1.900103
C -2.884648 3.875359 -0.507155
C -6.053032 1.843006 -1.061170
C -5.940793 2.461774 0.336920
C -4.207263 4.686058 -2.489147
C -2.600758 -6.275228 -0.715305
C -3.622728 -6.493085 0.402104
C -1.552571 -4.548587 -3.101416
C -1.403621 -5.893489 -3.823313
C -1.188717 -6.574310 -0.197859
C -1.806248 -3.426162 -4.112879
C -5.115473 -3.216604 -2.885239
C -4.053643 -3.811422 3.369008
C -7.199882 2.506686 -1.833702
C -3.618139 1.823692 -4.878292
C -8.156308 -6.808823 1.715339
C -7.090720 -7.843659 2.095937
C -13.017117 -3.018205 -1.803404
C -13.733381 -4.236473 -1.233809
C -13.183424 -6.105730 0.248149
C -11.992334 -6.808445 -0.390158
C -9.698589 -0.529414 1.865316
C -9.347034 0.748587 2.635162
C -11.232776 -1.122975 -4.270648
C -10.398305 -0.258266 -3.324402
C -12.405020 -3.898036 -5.003881
C -12.603139 -5.402188 -4.790258
C -9.321221 -3.605868 -4.070797
C -9.208774 -5.121674 -4.265379
C -12.098241 -3.607960 -6.478948
C -12.607540 -3.758170 2.264054
C -13.668300 -3.912127 1.185163
C -10.667055 -1.786118 4.527823
C -9.295513 -2.450292 4.662443
C -12.835749 -0.445761 2.723279
C -12.886182 0.610740 3.833508
C -11.699236 -2.543315 5.370719
C -13.218857 0.179275 1.377697
C -9.855556 -0.216170 0.375899
C -8.870330 -8.656343 -0.702756
C -9.890100 -9.237309 -1.688799
C -10.476327 -8.833839 2.038960
C -10.891561 -10.212061 1.514286
C -7.818466 -7.855863 -1.476533
C -11.616967 -8.215810 2.852491
C -8.609714 -6.038515 2.958973
C -8.606590 -2.879327 -5.216880
C -12.645809 -0.542417 -4.399011
Li -7.226964 -2.120368 -1.005872
Li -6.931197 -3.197972 0.991135
H -3.958967 1.801806 -5.923022
H -2.326007 -6.189235 -4.335871
H -0.618552 -5.837913 -4.590418
H -4.657113 -3.114146 -3.873458
H -4.527914 -5.677543 -4.013721
H -2.677791 -3.643319 -4.741097
H -3.116338 2.785550 -4.726074
H -6.203902 -3.221626 -3.053448
H -0.950532 -3.302615 -4.791063
H -6.113857 -5.736212 -3.250183
H -2.861796 1.036762 -4.777603
H -1.132086 -6.708797 -3.145476
H -5.822002 0.215463 -5.253442
H -4.866563 -2.306425 -2.328081

H -4.777811 -6.666245 -2.571996
H -1.982962 -2.460024 -3.627628
H -5.526407 2.402872 -4.114957
H -4.553319 4.491506 -3.509930
H -3.597251 5.599662 -2.523589
H -4.772881 -0.564373 -4.064243
H -0.585587 -4.336122 -2.620029
H -2.529465 3.371670 -2.543965
H -1.851162 -0.423034 -2.603250
H -5.243383 -4.573293 -1.242244
H -1.810634 1.321999 -2.621009
H -2.813733 -7.005584 -1.510201
H -6.337546 0.088374 -3.568361
H -5.089913 4.918414 -1.881472
H -0.422040 -1.825207 -1.625874
H 0.082130 0.522390 -1.321504
H -0.412954 -6.412644 -0.954849
H -2.150484 4.691066 -0.561996
H -7.414096 2.010392 -2.785324
H -4.654357 -6.380982 0.051494
H -0.488935 -3.983450 -0.450689
H -2.398547 3.027550 -0.014938
H -1.108395 -7.620672 0.128102
H 0.690170 -1.860755 -0.243951
H -3.534661 -7.500836 0.830682
H -6.987115 3.558864 -2.055263
H -1.064116 1.387382 -0.284602
H -3.686965 4.215015 0.155377
H -6.305699 0.778877 -0.928949
H -0.937266 -5.953246 0.669186
H -3.471270 -5.779102 1.219222
H -8.126656 2.489553 -1.244121
H -1.026398 -3.209125 1.018584
H -5.803241 3.547360 0.282186
H 0.262656 0.277664 1.365241
H -5.103524 2.057250 0.915193
H -0.332837 -1.319279 1.853947
H -3.597177 -3.686499 2.380477
H -2.041500 1.196293 1.742509
H -6.857833 2.292493 0.916194
H -4.788482 -4.625667 3.296747
H -5.487552 -2.229231 3.177200
H -5.760492 0.836595 2.986951
H -1.361348 0.476945 3.181864
H -3.266921 -4.164895 4.046226
H -1.189766 -3.044116 3.809304
H -6.447070 -0.263910 4.188195
H -0.199318 -1.596686 4.008256
H -3.556672 2.118228 3.535911
H -6.102205 -3.569133 5.196679
H -6.380244 1.471111 4.511872
H -5.848537 -1.882726 5.654256
H -0.266085 -2.793946 5.296066
H -4.611098 -3.092674 5.997887
H -4.271482 2.627620 5.065473
H -2.571478 -2.265342 5.812340
H -2.710956 1.803925 5.053570
H -4.568303 0.248227 5.751471
H -0.953556 0.322396 5.566035
H -2.322460 0.059635 6.655374
H -0.837731 -0.870860 6.856546
H -6.214152 -0.888624 0.889112
H -12.610905 0.490853 -4.772039
H -12.132841 1.394240 3.691791
H -13.864596 1.111221 3.848486
H -10.594760 0.573224 0.207012
H -10.137455 1.501627 2.536841
H -12.578419 1.034670 1.133918
H -13.277496 -1.111544 -5.089666
H -8.909007 0.139262 -0.054558
H -14.251847 0.553686 1.397055
H -8.427037 1.202336 2.245735
H -13.159346 -0.510899 -3.431312
H -12.729718 0.184357 4.829255
H -10.322829 0.776808 -3.684888
H -10.178517 -1.079247 -0.215982
H -9.194072 0.571780 3.704145
H -13.144137 -0.534314 0.550105
H -10.769476 -1.077637 -5.267357
H -12.001521 -2.537630 -6.689377
H -12.896012 -3.994629 -7.128342
H -10.851474 -0.225458 -2.328418
H -13.608779 -1.192610 2.958940
H -13.368959 -3.417230 -4.778537
H -13.126600 -2.180153 -1.095427
H -8.849075 -1.224154 1.967459
H -13.574388 -2.706044 -2.699277
H -10.585438 -0.771422 4.945685
H -9.375940 -0.636006 -3.204893
H -11.168478 -4.092224 -6.800669
H -13.957775 -2.912451 0.846785
H -14.812037 -4.048783 -1.094433
H -12.705499 -2.116079 5.296039
H -13.495429 -5.758352 -5.323459
H -8.544274 -1.797721 -5.061242
H -8.508596 -1.884097 4.152256
H -13.092560 -3.282952 3.129869
H -12.725369 -5.662257 -3.734684
H -11.420384 -2.530790 6.433618
H -14.576440 -4.413338 1.563251

H -8.999381 -2.538128 5.716613
H -9.109694 -3.041628 -6.176971
H -13.632744 -5.059522 -1.946285
H -11.755904 -5.982410 -5.168615
H -8.802498 -3.355526 -3.131383
H -11.764827 -3.595684 5.072622
H -9.303430 -3.462690 4.243208
H -7.579227 -3.250527 -5.332650
H -12.308473 -4.755983 2.622470
H -9.626888 -5.428721 -5.229986
H -14.140541 -6.504063 -0.129178
H -9.731316 -5.688049 -3.487760
H -13.149578 -6.291225 1.324164
H -9.333638 -5.250678 2.723586
H -12.046536 -6.680581 -1.484506
H -8.159082 -5.443079 -4.262399
H -7.756749 -5.566730 3.467248
H -7.702501 -6.086230 1.021208
H -8.274885 -6.996537 -1.981815
H -12.129623 -7.889134 -0.226023
H -9.079989 -6.701488 3.694835
H -11.381847 -7.210515 3.214720
H -7.017630 -7.476126 -0.834508
H -12.534578 -8.144901 2.258416
H -10.414092 -8.442470 -2.231512
H -6.235854 -7.364773 2.592244
H -7.347858 -8.471449 -2.255355
H -6.698926 -8.390451 1.232406
H -11.854647 -8.836075 3.728050
H -7.482681 -8.587536 2.800803
H -9.389328 -9.860726 -2.442810
H -9.643312 -9.006846 2.737660
H -10.647913 -9.861541 -1.206827
H -8.361544 -9.496998 -0.206231
H -11.746867 -10.152501 0.830580
H -10.079802 -10.723496 0.987120
H -11.196848 -10.865052 2.344341
H -7.982744 -4.438759 -0.851185

Supplementary Table 20. Final coordinates and energy for 3Li

Energy: -2765.544159 a.u
U -9.948728 -4.280018 -0.150417
Si -10.073985 -1.610538 2.593173
Si -10.793396 -3.454183 -3.826910
Si -9.516986 -7.963975 0.743471
N -10.669680 -3.075679 1.769473
N -12.637135 -4.576072 0.247128
N -11.146626 -3.598830 -2.081863
N -10.462163 -6.543976 0.230966
N -7.885957 -4.255312 -0.551830
N -4.877743 -3.948215 -2.407257
N -3.490754 -1.997127 -0.605489
N -4.165937 0.549923 -2.474032
N -4.765806 -4.035641 1.320797
C -7.485917 -0.233355 2.655000
C -8.401433 -1.109230 1.737644
C -8.585525 -0.478947 0.354146
C -11.417385 -0.186254 2.551754
C -11.928497 0.170412 1.153186
C -11.903632 -3.590127 2.384644
C -13.028552 -3.705569 1.366458
C -13.301578 -4.175666 -1.003706
C -12.490332 -3.108260 -1.728661
C -10.489216 -1.605247 -4.387676
C -11.750877 -0.736885 -4.446010
C -9.682837 -1.857329 4.497422
C -10.901022 -1.974642 5.420970
C -12.299822 -4.047777 -4.936417
C -12.021066 -3.892560 -6.436839
C -9.172875 -4.452411 -4.226341
C -8.426152 -3.955844 -5.469744
C -12.851418 -5.999026 0.557355
C -11.838718 -6.870017 -0.176981
C -7.984128 -7.380741 1.780898
C -8.395927 -6.712530 3.096112
C -8.831497 -8.985529 -0.776192
C -7.691059 -8.251115 -1.488946
C -10.471751 -9.215411 1.926900
C -11.507248 -8.559552 2.847179
C -8.728306 -3.025630 4.748268
C -10.995915 1.080859 3.305379
C -12.844776 -5.448213 -4.642082
C -9.409412 -5.962560 -4.327462
C -9.911686 -9.347003 -1.802210
C -11.070091 -10.469586 1.281133
C -6.989789 -8.514166 2.056533
C -9.426679 -0.923135 -3.524441
C -3.426688 -3.727514 -2.411508
C -2.990727 -2.345476 -1.937786
C -3.368182 -0.561281 -0.329257
C -4.365010 0.354747 -1.039236
C -5.317354 1.215404 -3.062880
C -5.150105 -5.315473 -2.853220
C -5.553314 -3.001782 -3.297859
C -2.798663 -2.761278 0.438709
C -3.686544 -3.087706 1.629566
C -4.246847 -5.392690 1.141515
C -5.732288 -4.038776 2.420882
C -2.952905 1.293437 -2.772270

Li -5.946711 -3.661124 -0.493859
H -11.518628 0.265818 -4.832729
H -10.166427 1.590498 2.800355
H -11.823599 1.802419 3.357789
H -8.994286 0.535742 0.422872
H -7.943424 0.736940 2.825777
H -11.193614 0.754220 0.588658
H -12.527486 -1.157693 -5.094038
H -7.626416 -0.401436 -0.175639
H -12.844268 0.775667 1.207419
H -6.541215 -0.021592 2.078894
H -12.191871 -0.596457 -3.451973
H -10.678812 0.879465 4.334211
H -9.171907 0.073328 -3.913229
H -9.255981 -1.059521 -0.287785
H -7.230213 -0.700523 3.556535
H -12.155540 -0.721783 0.561301
H -10.095532 -1.683835 -5.411706
H -11.675352 -2.887649 -6.702528
H -12.927328 -4.088758 -7.027308
H -9.789084 -0.791069 -2.499348
H -12.268291 -0.622409 3.098299
H -13.103158 -3.337569 -4.689954
H -12.438910 -2.210916 -1.088675
H -7.898276 -2.076352 1.587047
H -13.086195 -2.794512 -2.652
H -9.158117 -0.930078 4.773850
H -8.498670 -1.502793 -3.462666
H -11.261109 -4.603769 -6.781876
H -13.228240 -2.707291 0.966341
H -14.332948 -3.837134 -0.800844
H -11.624144 -1.164096 5.280894
H -13.810737 -5.606377 -5.142344
H -8.133737 -2.903412 -5.398339
H -7.808167 -2.946876 4.160291
H -12.287954 -2.953470 3.197968
H -12.998219 -5.618361 -3.572978
H -10.592011 -1.950792 6.475816
H -13.963209 -4.069552 1.829645
H -8.435915 -3.085079 5.806418
H -9.032347 -4.062625 -6.376731
H -13.367410 -5.053215 -1.651569
H -12.170849 -6.233454 -4.999001
H -8.533557 -4.275516 -3.347862
H -11.433316 -2.920886 5.271964
H -9.196608 -3.980042 4.484475
H -7.508981 -4.538854 -5.633786
H -11.738833 -4.576021 2.850940
H -9.989846 -6.215563 -5.221735
H -13.890127 -6.293164 0.323285
H -9.948582 -6.359094 -3.461257
H -12.700151 -6.136847 1.631075
H -9.081175 -5.874339 2.932248
H -11.984523 -6.741442 -1.263023
H -8.459706 -6.509842 -4.404957
H -7.522551 -6.325107 3.638465
H -7.490556 -6.619978 1.157474
H -8.044910 -7.296943 -1.895519
H -12.108660 -7.919890 0.020032
H -8.891521 -7.422606 3.768965
H -11.144577 -7.634080 3.303180
H -6.843293 -8.035421 -0.830471
H -12.427712 -8.318276 2.304737
H -10.337554 -8.444365 -2.254577
H -6.109430 -8.145238 2.601161
H -7.308409 -8.840318 -2.334627
H -6.628872 -8.998424 1.143103
H -11.791107 -9.239951 3.662800
H -7.435125 -9.298098 2.682423
H -9.490688 -9.947490 -2.621359
H -9.649467 -9.563747 2.572066
H -10.738489 -9.919643 -1.372737
H -8.429221 -9.920548 -0.355919
H -11.916811 -10.230927 0.626331
H -10.337916 -11.026839 0.688048
H -11.450246 -11.156851 2.050752
H -7.723055 -5.047467 -1.197701
H -6.113545 -3.026314 2.571851
H -6.575782 -4.684382 2.170156
H -5.281386 -4.383925 3.367507
H -3.538180 -5.434056 0.310322
H -3.735017 -5.762913 2.047029
H -5.073516 -6.071589 0.915749
H -6.226958 -5.499461 -2.846024
H -4.775019 -5.503446 -3.874442
H -4.673873 -6.033755 -2.178679
H -6.624489 -3.210576 -3.292730
H -5.403027 -1.976021 -2.953864
H -5.182807 -3.074303 -4.335320
H -2.951669 2.308683 -2.330579
H -2.068573 0.765456 -2.404557
H -2.845073 1.398161 -3.856702
H -5.183140 1.299177 -4.146320
H -6.225687 0.634233 -2.878671
H -5.480139 2.234415 -2.662192
H -5.366888 -0.065249 -0.896979
H -4.349574 1.328243 -0.507491
H -2.331061 -0.215982 -0.499430
H -3.553540 -0.422149 0.741363
H -4.151723 -2.166497 1.996962

H -3.057661 -3.472574 2.453542
H -2.419219 -3.691012 0.007209
H -1.907162 -2.219675 0.801164
H -2.976462 -4.511914 -1.791299
H -3.018410 -3.870901 -3.429635
H -1.884383 -2.315111 -1.978401
H -3.350255 -1.580977 -2.631073

Supplementary Table 21. Final coordinates and energy for Li-TS-dis

Energy: -4143.755983 a.u

U -4.793726 -2.206546 -0.115993
U -11.319662 -4.361500 -0.241751
Si -4.335123 -1.883459 3.664528
Si -12.149676 -3.324420 -3.841972
Si -3.821942 -5.433884 -2.002229
Si -5.667761 0.950548 -2.177115
Si -11.931156 -1.952289 2.611115
Si -10.652797 -8.050236 0.530489
N -3.502239 -4.017528 -0.971225
N -12.469010 -3.555113 -2.087154
N -4.590028 -0.328648 -1.575549
N -2.156720 -1.472309 -0.344401
N -3.875984 -1.548199 1.977841
N -6.749436 -2.878155 -0.134715
N -9.113079 -3.961236 -0.541397
N -12.239682 -3.453263 1.661706
N -13.966096 -4.987 -0.040277
N -11.591198 -6.656225 -0.092809
C -1.667889 -3.040618 4.178375
C -4.614073 0.956165 4.354074
C -3.753735 3.162580 -1.719075
C -13.340419 -5.806230 -4.891586
C -5.634887 -6.606103 -0.104817
C -2.507113 -7.369593 -3.749164
C -5.962894 -6.254007 -3.815744
C -6.149426 -3.640891 5.082989
C -6.429969 2.067670 0.391925
C -7.407560 -0.925299 -3.486310
C -9.988031 -3.001269 4.502694
C -13.591297 0.075755 3.945108
C -10.128795 0.301608 2.545697
C -10.973738 -9.529443 -1.999879
C -8.287239 -8.509124 2.131103
C -10.044594 -5.347922 -3.808404
C -11.208466 -0.575749 -3.752820
C -6.716430 -0.285002 3.826974
C -5.324820 -0.398364 4.454749
C -4.784987 -4.715488 3.273698
C -5.505990 -3.435130 3.707025
C -2.343956 -1.255641 5.806056
C -2.851326 -2.357006 4.869669
C -9.899701 -3.652546 -5.658053
C -10.334305 -3.897630 -4.207666
C -13.657850 -0.813518 -4.233949
C -12.276418 -1.457356 -4.400418
C -13.582593 -3.787187 -6.363447
C -13.482162 -4.280982 -4.914172
C -1.494202 -5.074600 -3.813694
C -2.234591 -6.093308 -2.942306
C -4.890108 -4.033063 -4.312258
C -5.286598 -5.020075 -3.208873
C -3.277331 -7.458826 0.013387
C -4.387081 -6.959114 -0.914744
C -8.251597 2.038731 -1.341441
C -7.004106 1.319816 -0.816028
C -5.698189 0.146549 -4.981505
C -6.622960 0.355004 -3.779204
C -1.453429 -2.635876 -0.907644
C -2.124502 -3.929614 -0.458743
C -14.611900 -4.465723 -1.260214
C -13.871972 -3.230904 -1.752206
C -5.623573 3.636390 -3.329660
C -4.703095 2.552295 -2.754502
C -3.282400 -0.393053 -2.251775
C -2.132351 -0.315767 -1.253274
C -1.654903 -1.137074 0.997266
C -2.774520 -0.579873 1.864691
C -13.986064 -0.184109 1.479756
C -13.581747 -0.908038 2.768042
C -10.694226 -0.636172 0.291882
C -10.487932 -0.972053 1.771154
C -12.520787 -10.294771 1.144711
C -9.478317 -6.346832 2.586621
C -9.121417 -7.379244 1.515692
C -12.362580 -3.097966 5.297584
C -11.356230 -2.321442 4.440199
C -13.377961 -4.216830 2.206685
C -14.484234 -4.321618 1.170594
C -14.049565 -6.448402 0.050513
C -12.878070 -7.071830 -0.691923
C -8.689934 -8.637551 -1.534159
C -9.962434 -9.201181 -0.894990
C -11.624026 -9.217187 1.767445
C -12.364873 -8.451428 2.887525
Li -7.183715 -4.693433 0.339781
Li -8.005523 -2.304846 -1.481218
H -6.262255 -0.173576 -5.868878
H -3.110345 -7.164109 -4.640826
H -1.565318 -7.812586 -4.101708

H -4.247160 -4.509161 -5.061251
H -5.273850 -6.827996 -4.444522
H -2.085998 -4.776333 -4.685337
H -5.163111 1.062053 -5.255137
H -5.773202 -3.660106 -4.848898
H -0.554514 -5.498604 -4.194930
H -6.803758 -5.965419 -4.460665
H -4.947276 -0.626757 -4.782950
H -3.027005 -8.139179 -3.169095
H -7.873758 -1.341104 -4.388658
H -4.350333 -3.166548 -3.916696
H -6.354800 -6.941282 -3.058452
H -1.239565 -4.162391 -3.268297
H -7.348440 1.138208 -4.038681
H -6.307707 3.254514 -4.095082
H -5.033806 4.438484 -3.794945
H -6.728775 -1.691265 -3.079553
H -1.547896 -6.379355 -2.131648
H -4.079157 2.197553 -3.588423
H -3.167988 -1.323596 -2.835918
H -6.017999 -4.510730 -2.559062
H -3.131695 0.414476 -2.984391
H -4.654284 -7.772486 -1.602861
H -8.222477 -0.706064 -2.778998
H -6.232064 4.106139 -2.548583
H -1.518989 -2.577281 -1.997670
H -1.154770 -0.242686 -1.761965
H -2.396433 -7.798359 -0.541829
H -3.144263 3.957586 -2.171111
H -8.751002 1.496301 -2.151609
H -6.467465 -6.355834 -0.779478
H -1.483249 -4.764526 -0.779029
H -3.066824 2.425187 -1.294819
H -3.616387 -8.304556 0.627988
H -0.383285 -2.609305 -0.636121
H -5.962697 -7.436003 0.533528
H -8.009293 3.035721 -1.725290
H -2.261156 0.588718 -0.652427
H -4.298085 3.613921 -0.883475
H -7.309755 0.321169 -0.460297
H -2.948674 -6.670985 0.701359
H -5.397947 -5.755308 0.552498
H -8.991147 2.180023 -0.542117
H -2.100873 -3.964091 0.643854
H -6.192979 3.107626 0.140074
H -0.800407 -0.440820 0.928829
H -5.514937 1.600441 0.769280
H -1.294372 -2.059015 1.460732
H -4.304324 -4.613061 2.294405
H -3.113766 0.377101 1.432374
H -7.152791 2.098983 1.217734
H -5.475656 -5.569712 3.223718
H -6.305325 -3.227121 2.978995
H -6.645785 -0.115271 2.745857
H -2.327238 -0.309577 2.836092
H -4.001977 -4.995348 3.988909
H -1.979599 -3.805389 3.460675
H -7.317935 -1.186785 3.974092
H -1.052815 -2.314451 3.636165
H -4.501158 1.262627 3.308253
H -6.843177 -4.492659 5.074820
H -7.275414 0.558029 4.255606
H -6.713393 -2.766703 5.421956
H -1.009919 -3.525214 4.913682
H -5.395400 -3.858339 5.850374
H -5.198185 1.741276 4.855216
H -3.326876 -3.114510 5.512409
H -3.618412 0.955334 4.806449
H -5.446766 -0.643802 5.521321
H -1.817846 -0.463105 5.260140
H -3.150908 -0.785692 6.377598
H -1.628832 -1.664246 6.534742
H -8.003005 -3.321622 -0.162629
H -13.692320 0.163780 -4.734744
H -12.822890 0.850226 3.840838
H -14.558183 0.594651 4.007129
H -11.448327 0.144503 0.150778
H -10.928733 1.048738 2.481211
H -13.331756 0.671555 1.279433
H -14.467412 -1.419633 -4.655504
H -9.760187 -0.263044 -0.148522
H -15.008925 0.210465 1.555216
H -9.224248 0.765729 2.134453
H -13.892232 -0.635846 -3.178312
H -13.429752 -0.416062 4.908849
H -11.266287 0.457399 -4.122559
H -11.015047 -1.493839 -0.309967
H -9.939976 0.112296 3.606694
H -13.951837 -0.836493 0.601243
H -12.062812 -1.497568 -5.478802
H -13.798076 -2.716811 -6.430842
H -14.387713 -4.312491 -6.895762
H -11.329655 -0.537686 -2.663918
H -14.359730 -1.659224 2.971575
H -14.439440 -4.038764 -4.427755
H -13.925029 -2.449634 -0.979058
H -9.624576 -1.651580 1.830986
H -14.436472 -2.824269 -2.603105
H -11.249717 -1.321282 4.886634
H -10.198795 -0.937277 -3.966650

H -12.659153 -3.970820 -6.924506
H -14.798424 -3.307784 0.903885
H -15.680722 -4.262352 -1.077358
H -13.372177 -2.673347 5.266941
H -14.232041 -6.289360 -5.314466
H -10.050859 -2.616458 -5.979500
H -9.217742 -2.436714 3.968652
H -13.813992 -3.769929 3.111857
H -13.203019 -6.203060 -3.880387
H -12.048193 -3.109277 6.350426
H -15.369694 -4.855080 1.556095
H -9.650836 -3.113812 5.542005
H -10.452787 -4.294828 -6.352959
H -14.550293 -5.234128 -2.036385
H -12.487237 -6.139411 -5.493056
H -9.719829 -3.247572 -3.564470
H -12.435444 -4.143491 4.977846
H -10.024706 -4.003794 4.063533
H -8.834865 -3.883562 -5.794864
H -13.067349 -5.227962 2.511976
H -10.512058 -6.054033 -4.501447
H -15.019927 -6.806519 -0.333051
H -10.413142 -5.601967 -2.807330
H -13.983506 -6.735643 1.103110
H -10.037173 -5.491178 2.190380
H -12.936143 -6.778084 -1.752036
H -8.966749 -5.553139 -3.825156
H -8.576481 -5.948349 3.071404
H -8.502187 -6.882122 0.753025
H -8.882073 -7.661638 -1.995878
H -13.019275 -8.160832 -0.696347
H -10.091756 -6.788607 3.379064
H -11.916249 -8.627023 3.871630
H -7.875741 -8.515811 -0.813984
H -12.380302 -7.368011 2.731035
H -11.257145 -8.629686 -2.557326
H -7.369683 -8.118909 2.591707
H -8.321675 -9.300222 -2.329473
H -7.989746 -9.267858 1.399247
H -13.410774 -8.773823 2.959437
H -8.838159 -9.023381 2.927351
H -10.537692 -10.230666 -2.725008
H -10.790891 -9.765566 2.230637
H -11.893092 -9.987555 -1.623479
H -9.699037 -10.143481 -0.389202
H -13.404887 -9.872636 0.653388
H -11.988943 -10.905845 0.408180
H -12.889320 -10.979756 1.920846
H -8.833238 -4.540967 -1.350163

Supplementary Table 22. Final coordinates and energy for Li-Product-dis

Energy: -4143.797793 a.u
U -4.473107 -1.867567 -0.265830
U -11.929632 -4.628775 0.004266
Si -4.217695 -1.696727 3.569508
Si -12.435467 -3.464362 -3.522725
Si -3.487289 -5.282580 -2.238071
Si -4.923232 1.226817 -2.452010
Si -12.332518 -2.030803 2.691474
Si -11.229019 -8.283337 0.606517
N -3.374609 -4.021377 -0.966815
N -12.994982 -3.794742 -1.834765
N -3.940523 -0.105194 -1.821051
N -1.783127 -1.500114 -0.347882
N -3.636098 -1.383214 1.924874
N -6.117277 -2.935998 -0.037582
N -9.569321 -4.396472 0.099102
N -12.772760 -3.542148 1.803537
N -14.550575 -5.113779 0.153188
N -12.262967 -6.886039 0.089673
C -1.610621 -2.846146 4.344918
C -4.563311 1.147903 4.131503
C -2.901919 3.284466 -1.811099
C -13.458474 -6.019067 -4.556860
C -4.861588 -7.051920 -0.384761
C -1.933019 -6.747343 -4.221510
C -5.727920 -6.246944 -3.860502
C -6.138407 -3.440621 4.877054
C -5.723290 2.243644 0.160860
C -6.820387 -0.411482 -3.884718
C -10.545035 -3.230213 4.643461
C -13.785117 0.254790 3.815852
C -10.308944 0.026869 2.559713
C -11.561933 -9.573770 -1.998256
C -8.775034 -8.753143 2.070618
C -10.193123 -5.319221 -3.312906
C -11.839227 -0.632851 -3.206211
C -6.628316 -0.123473 3.524452
C -5.281154 -0.201214 4.248040
C -4.641409 -4.542991 3.193631
C -5.382247 -3.253132 3.558399
C -2.385352 -0.977593 5.827734
C -2.836263 -2.117949 4.908195
C -9.868768 -3.394308 -4.894623
C -10.538062 -3.854205 -3.594175
C -14.084065 -1.117257 -4.228646
C -12.639084 -1.605635 -4.074827
C -13.275446 -4.159850 -6.244618

C	-13.521228	-4.504325	-4.770591
C	-1.569417	-4.272485	-4.278854
C	-1.904979	-5.466308	-3.375721
C	-4.972985	-3.897925	-4.323266
C	-5.125965	-4.977815	-3.246259
C	-2.440411	-7.590610	-0.705644
C	-3.699676	-7.043159	-1.385755
C	-7.494235	2.385939	-1.615599
C	-6.286207	1.591989	-1.106208
C	-4.917325	0.489721	-5.261702
C	-5.872307	0.769961	-4.097243
C	-1.207466	-2.770471	-0.820474
C	-2.034083	-3.969195	-0.347440
C	-15.172520	-4.674556	-1.109173
C	-14.424012	-3.473369	-1.661341
C	-4.673424	3.959008	-3.466453
C	-3.843353	2.794143	-2.914243
C	-2.599625	-0.295893	-2.376209
C	-1.539218	-0.378942	-1.276055
C	-1.343598	-1.184835	1.024740
C	-2.457562	-0.513998	1.826278
C	-14.272720	-0.255339	1.399777
C	-13.887991	-0.850769	2.757948
C	-10.946164	-0.940864	0.338857
C	-10.786522	-1.235909	1.832021
C	-12.783822	-10.783923	1.089215
C	-10.100754	-6.752856	2.809457
C	-9.708751	-7.624621	1.613615
C	-12.916316	-2.958993	5.427748
C	-11.811093	-2.377212	4.538526
C	-13.966428	-4.212441	2.362993
C	-15.054463	-4.343819	1.308172
C	-14.699287	-6.565484	0.360049
C	-13.598430	-7.323274	-0.368962
C	-9.271814	-8.671189	-1.533226
C	-10.522505	-9.295261	-0.906441
C	-12.153003	-9.564588	1.772324
C	-13.141896	-8.947607	2.766549
Li	-5.150962	-4.570494	-0.070638
Li	-7.977984	-3.080019	-0.051333
H	-5.468007	0.240914	-6.180267
H	-2.724695	-6.709574	-4.978892
H	-0.984729	-6.874836	-4.761905
H	-4.379602	-4.259413	-5.170157
H	-5.041034	-6.719671	-4.572267
H	-2.213139	-4.245596	-5.163884
H	-4.276645	1.348144	-5.493482
H	-5.951717	-3.606178	-4.725370
H	-0.534754	-4.339645	-4.641663
H	-6.645555	-6.012711	-4.417367
H	-4.260076	-0.360165	-5.042493
H	-2.089528	-7.653227	-3.627902
H	-7.347018	-0.677047	-4.812296
H	-4.492081	-2.991103	-3.943461
H	-5.987895	-7.002078	-3.111397
H	-1.678100	-3.306037	-3.778574
H	-6.478542	1.649050	-4.362206
H	-5.338757	3.655829	-4.282050
H	-4.023362	4.753974	-3.858753
H	-6.273396	-1.301177	-3.553661
H	-1.079650	-5.571558	-2.655095
H	-3.208614	2.437496	-3.739940
H	-2.534385	-1.215586	-2.987469
H	-5.841524	-4.581781	-2.506233
H	-2.281848	0.511986	-3.056836
H	-3.966084	-7.728186	-2.202719
H	-7.582486	-0.200948	-3.126039
H	-5.296047	4.416445	-2.688542
H	-1.208789	-2.761389	-1.912612
H	-0.522467	-0.447559	-1.702900
H	-1.578331	-7.628161	-1.379633
H	-2.228403	4.067718	-2.187693
H	-7.976767	1.920258	-2.480922
H	-5.789679	-6.625822	-0.795112
H	-1.427709	-4.870725	-0.532753
H	-2.276502	2.480982	-1.411705
H	-2.602580	-8.612185	-0.333277
H	-0.155719	-2.858597	-0.493411
H	-5.117467	-8.068994	-0.059723
H	-7.212619	3.403342	-1.910133
H	-1.589231	0.546197	-0.693783
H	-3.451348	3.715050	-0.967387
H	-6.650941	0.587754	-0.826790
H	-2.152472	-6.980934	0.159049
H	-4.588438	-6.519963	0.541000
H	-8.258574	2.482526	-0.831565
H	-2.121237	-3.908543	0.749280
H	-5.444034	3.287685	-0.021896
H	-0.428151	-0.567447	1.274000
H	-4.833062	1.725199	0.531860
H	-1.093161	-2.123638	1.525268
H	-4.065291	-4.431935	2.266855
H	-2.695612	0.451639	1.343740
H	-6.467301	2.250814	0.968685
H	-5.342316	-5.381504	3.064185
H	-6.105154	-3.057801	2.753776
H	-6.483610	-0.004421	2.443454
H	-2.029629	-0.239297	2.804867
H	-3.935030	-4.843228	3.977870
H	-1.875667	-3.653422	3.655516

H -7.237158 -1.020907 3.671218
H -0.952539 -2.157530 3.803734
H -4.352960 1.388362 3.083335
H -6.850785 -4.275825 4.812811
H -7.217452 0.736427 3.873216
H -6.707859 -2.552594 5.169214
H -1.009982 -3.286939 5.153694
H -5.458646 -3.673547 5.706932
H -5.185627 1.962709 4.529131
H -3.368170 -2.838538 5.549520
H -3.612141 1.174956 4.671007
H -5.472848 -0.401549 5.313541
H -1.827348 -0.206002 5.283646
H -3.224803 -0.486968 6.331218
H -1.715496 -1.355373 6.614193
H -9.228578 -4.705498 1.014789
H -14.110358 -0.133721 -4.717065
H -12.978455 0.960651 3.591454
H -14.716319 0.836307 3.858508
H -11.623449 -0.098031 0.170064
H -11.032860 0.843511 2.459101
H -13.567368 0.522541 1.086727
H -14.699820 -1.792634 -4.833119
H -9.982827 -0.669079 -0.113430
H -15.263925 0.216082 1.444905
H -9.363637 0.385824 2.134609
H -14.575977 -1.530000 -3.256570
H -13.605109 -0.138674 4.820900
H -11.871739 0.384673 -3.618870
H -11.349615 -1.780989 -0.238896
H -10.142948 -0.137972 3.628615
H -14.302447 -1.009345 0.606074
H -12.189432 -1.601330 -5.079450
H -13.359090 -3.087729 -6.449231
H -14.003938 -4.670726 -6.888862
H -12.249702 -0.582963 -2.192580
H -14.712529 -1.511831 3.065695
H -14.550816 -4.190827 -4.537100
H -14.555904 -2.613669 -0.986624
H -9.997477 -1.996176 1.939510
H -14.921874 -3.183340 -2.597194
H -11.571408 -1.376620 4.929167
H -10.783679 -0.913292 -3.122435
H -12.281235 -4.480055 -6.576080
H -15.319338 -3.340302 0.962921
H -16.244423 -4.462497 -0.960661
H -13.857111 -2.401066 5.360316
H -14.256641 -6.529418 -5.113096
H -10.020060 -2.328664 -5.091856
H -9.702871 -2.802367 4.089821
H -14.400484 -3.673473 3.216282
H -13.565726 -6.291120 -3.501785
H -12.610677 -2.950784 6.482786
H -15.967571 -4.808804 1.715316
H -10.223204 -3.334653 5.688337
H -10.245312 -3.946668 -5.762664
H -15.093311 -5.491579 -1.832190
H -12.509519 -6.438787 -4.908595
H -10.108008 -3.250953 -2.779117
H -13.130923 -4.002314 5.170165
H -10.715156 -4.241154 4.255831
H -8.785296 -3.563984 -4.851962
H -13.714850 -5.209636 2.758090
H -10.486753 -5.970755 -4.142100
H -15.701684 -6.900708 0.045986
H -10.694890 -5.711291 -2.417777
H -14.602840 -6.765125 1.430401
H -10.790609 -5.944655 2.536435
H -13.713476 -7.183179 -1.454266
H -9.112370 -5.447085 -3.169484
H -9.218494 -6.292693 3.274046
H -9.144545 -6.998419 0.908992
H -9.492490 -7.698060 -1.985212
H -13.773312 -8.395107 -0.198984
H -10.597471 -7.343752 3.587603
H -12.748230 -8.056769 3.265205
H -8.461555 -8.526585 -0.811731
H -14.078656 -8.664690 2.274874
H -11.878730 -8.645815 -2.487298
H -7.856383 -8.342622 2.510320
H -8.879573 -9.310342 -2.335905
H -8.474412 -9.417514 1.253586
H -13.404011 -9.671259 3.550406
H -9.241053 -9.376318 2.842753
H -11.141947 -10.221065 -2.780236
H -11.307175 -9.950181 2.362293
H -12.460508 -10.068484 -1.617346
H -10.226160 -10.261325 -0.468792
H -13.657155 -10.517542 0.482051
H -12.079012 -11.312988 0.440429
H -13.131874 -11.504956 1.841729
H -9.134321 -5.047880 -0.560971

Supplementary Table 23. Final coordinates and energy for 6Li

Energy: -2764.935568 a.u
U -10.388152 -9.592586 5.916004
Si -7.509557 -11.217706 3.985745
Si -13.669626 -11.515038 6.264825
Si -10.037649 -6.125351 7.515624

N -12.099699 -10.907743 6.869683
N -9.790961 -7.899013 7.483860
N -9.503004 -10.697973 8.151792
N -8.545515 -10.983590 5.432300
N -10.985186 -8.865717 4.345603
N -12.340577 -11.085604 -0.333283
N -13.271687 -7.082681 2.122834
N -11.867865 -7.824929 -0.598585
N -10.082932 -5.144955 -0.077532
C -7.576749 -8.564095 2.815341
C -4.678232 -11.088764 3.277668
C -7.926838 -5.626115 5.611018
C -9.118613 -13.412441 3.054118
C -11.460292 -6.322539 10.097598
C -12.854423 -5.981009 6.867690
C -15.236741 -11.491570 3.835583
C -15.217243 -10.641160 8.632447
C -14.732251 -13.957947 7.638141
C -5.337520 -9.732992 5.305952
C -5.627651 -10.987142 4.476739
C -6.915851 -14.125814 4.034529
C -7.651335 -13.028472 3.258133
C -7.671833 -10.461582 1.167985
C -8.056287 -9.998133 2.577288
C -15.381815 -9.201461 6.599263
C -15.209674 -10.638758 7.099634
C -12.540034 -14.213926 6.438195
C -13.873706 -13.461624 6.470580
C -12.789100 -11.963378 3.538632
C -13.827139 -11.193816 4.359810
C -10.754299 -4.007877 9.384573
C -10.416700 -5.502703 9.333930
C -11.408127 -4.238955 5.761128
C -11.469524 -5.677696 6.287018
C -10.477387 -11.731792 8.545538
C -11.901753 -11.270467 8.281259
C -7.313443 -5.121019 7.993881
C -8.446643 -5.131296 6.962312
C -8.901665 -8.342297 8.566267
C -9.391950 -9.644960 9.177228
C -8.185351 -11.289401 7.862657
C -8.155410 -11.931824 6.486353
C -12.716073 -5.783704 2.521608
C -11.426915 -12.203120 -0.141420
C -13.676051 -11.592283 -0.613103
C -11.855373 -10.225107 -1.410480
C -12.502903 -8.840802 -1.452960
C -9.178297 -4.590118 0.918524
C -14.346488 -7.443500 3.055943
C -13.827200 -7.028608 0.759630
C -12.816844 -6.734289 -0.342399
C -10.660241 -7.324472 -1.268608
C -9.691304 -6.516259 -0.405650
C -10.123114 -4.283264 -1.248539
Li -11.770306 -8.455494 2.642258
H -6.466949 -4.515829 7.639396
H -4.795126 -10.238025 2.596129
H -3.628197 -11.092802 3.602935
H -6.486622 -8.483561 2.734551
H -6.585339 -10.511660 1.030867
H -5.410789 -8.819995 4.705744
H -7.623831 -4.704305 8.958196
H -8.132000 -7.878056 2.069004
H -4.321240 -9.760999 5.723735
H -8.051992 -9.759579 0.412338
H -6.925437 -6.129301 8.180380
H -4.837772 -12.001826 2.693068
H -7.091347 -5.009759 5.249792
H -7.869495 -8.187059 3.799275
H -8.076054 -11.449217 0.924764
H -6.038152 -9.624473 6.140326
H -8.784379 -4.092033 6.835242
H -10.005091 -3.390574 8.876278
H -10.813278 -3.653787 10.423478
H -7.561534 -6.656407 5.689387
H -5.413041 -11.850428 5.124503
H -9.463681 -5.624681 9.869499
H -7.860123 -8.470663 8.222780
H -9.153513 -9.989662 2.650158
H -8.831864 -7.623445 9.398500
H -7.177548 -12.964919 2.267271
H -8.702330 -5.617067 4.837089
H -11.725280 -3.794005 8.922703
H -7.445466 -10.484307 7.882469
H -8.737567 -9.985581 9.998387
H -5.855241 -13.898468 4.186882
H -11.515302 -6.006304 11.149076
H -10.486718 -4.037543 5.205598
H -9.663339 -12.669778 2.461814
H -7.130073 -12.318570 6.365998
H -11.230852 -7.392622 10.087460
H -6.964597 -15.082343 3.494746
H -7.914053 -12.011458 8.652393
H -9.216650 -14.380495 2.542391
H -11.470105 -3.500961 6.569180
H -10.388376 -9.473567 9.593261
H -12.463039 -6.205692 9.674027
H -11.310697 -6.365930 5.442482
H -7.361987 -14.296908 5.020383
H -9.636481 -13.494617 4.016068

H -12.247495 -4.030513 5.082987
H -8.795366 -12.831492 6.486276
H -13.111018 -5.292024 7.680562
H -10.329279 -12.005178 9.604721
H -12.916720 -6.999315 7.265388
H -10.284558 -12.621463 7.940340
H -11.767142 -11.770156 3.877358
H -12.142255 -10.434097 8.958471
H -13.638487 -5.873788 6.105283
H -12.836690 -11.673716 2.480156
H -13.622075 -10.121611 4.234249
H -14.525293 -8.581403 6.886295
H -12.556040 -12.094404 8.611765
H -12.959250 -13.046223 3.586814
H -11.890016 -13.885504 5.621967
H -15.477756 -9.141601 5.510966
H -11.982946 -14.074358 7.371116
H -14.416854 -10.008535 9.031508
H -15.311074 -11.279092 2.759698
H -16.278256 -8.734564 7.031673
H -16.013988 -10.904758 4.335772
H -12.700703 -15.295149 6.321716
H -15.500748 -12.549440 3.961224
H -16.164155 -10.236636 9.017419
H -14.417193 -13.729428 5.550728
H -15.093953 -11.639452 9.061538
H -16.078154 -11.224537 6.759015
H -14.256744 -13.770092 8.608321
H -15.722203 -13.490968 7.659236
H -14.889862 -15.044121 7.570212
H -10.435880 -11.830953 0.133024
H -11.784212 -12.844874 0.668907
H -11.319345 -12.830765 -1.047210
H -14.396437 -10.774945 -0.711935
H -13.718792 -12.193325 -1.542573
H -14.011443 -12.225831 0.213385
H -13.944006 -7.522544 4.069266
H -15.155717 -6.693718 3.059476
H -14.776608 -8.409036 2.774342
H -12.335733 -5.855824 3.543050
H -11.889352 -5.495479 1.867953
H -13.480175 -4.988079 2.489150
H -9.146613 -4.212437 -1.765020
H -10.861386 -4.639425 -1.972413
H -10.418888 -3.272909 -0.948336
H -9.501880 -3.580941 1.193492
H -9.186293 -5.206193 1.822633
H -8.132428 -4.525313 0.562376
H -9.551213 -7.058005 0.536423
H -8.710177 -6.522294 -0.922693
H -10.923345 -6.766601 -2.187335
H -10.091106 -8.195025 -1.609849
H -10.773363 -10.115526 -1.284678
H -12.002918 -10.714319 -2.394130
H -13.550765 -8.931900 -1.155631
H -12.519587 -8.488045 -2.501658
H -14.327060 -7.986236 0.575169
H -14.610859 -6.250410 0.712995
H -13.387393 -6.486184 -1.257697
H -12.239228 -5.842899 -0.087635

Supplementary Table 24. Final coordinates and energy for Na-TS-in

Energy: -2226.550548 a.u

U	-9.597930	-0.890669	2.842051
Na	-8.647823	-2.288873	-1.897592
Si	-13.249269	-1.699659	3.513626
Si	-6.752787	-3.417130	3.370676
Si	-8.652697	2.577977	1.709338
N	-8.061852	-2.329409	3.909269
N	-8.705255	1.272846	2.937743
N	-9.317019	-0.127064	5.403277
N	-11.679705	-0.923399	3.901610
N	-9.902471	-1.701346	0.785106
C	-15.284197	-1.726819	5.701326
C	-14.671580	0.864536	3.419638
C	-8.404365	-5.165344	1.724048
C	-5.210057	-3.581198	0.872144
C	-4.848299	-2.547149	5.486648
C	-5.025398	-1.423206	2.148592
C	-5.938414	-2.601891	1.799294
C	-4.315048	-4.699291	4.304685
C	-5.440011	-3.771989	4.782721
C	-11.492410	2.806644	1.174414
C	-9.654037	4.633948	3.594889
C	-14.281960	-3.729564	1.725771
C	-14.093184	-0.170624	1.216636
C	-14.495792	-0.458989	2.665899
C	-12.116514	-4.286342	2.857865
C	-12.965463	-3.157500	2.265208
C	-13.186808	-3.036267	6.144506
C	14.141705	-2.522192	5.062485
C	-6.944497	1.134352	-0.111606
C	-8.562631	5.479370	1.491359
C	-8.588590	4.352052	2.531638
C	-5.760840	2.769549	1.392323
C	-7.042838	2.481324	0.606169
C	-8.324482	-1.038507	6.002023
C	-8.391861	-2.412737	5.343441
C	-7.903415	-6.007194	4.034117

C	-7.368696	-5.197908	2.848223
C	-10.020905	3.001093	-0.848191
C	-10.179296	2.348062	0.530774
C	-7.973372	1.547557	4.182942
C	-8.853968	1.271963	5.397235
C	-10.636941	-0.272011	6.045136
C	-11.759908	-0.005333	5.051088
C	-11.460622	-1.337437	-1.329220
C	-10.958464	-3.038249	-3.110444
C	-10.963470	-1.693377	-2.642346
C	-10.343996	-0.745480	-3.505153
C	-9.749411	-1.114007	-4.707860
C	-9.735589	-2.450048	-5.126999
C	-10.361857	-3.402445	-4.313351
H	-10.351962	0.303488	-3.213677
H	-9.303707	-0.345646	-5.338016
H	-9.288946	-2.733424	-6.076102
H	-10.398416	-4.443434	-4.631128
H	-11.450365	-3.799738	-2.506581
H	-10.50596	-1.441312	-0.2097788
H	-9.975451	-2.696452	0.940152
H	-15.815852	-2.339729	6.443344
H	-16.025650	-1.396031	4.967000
H	-14.920789	-0.837719	6.230558
H	-15.467378	-0.976791	2.656707
H	-14.983572	0.731474	4.459759
H	-14.602897	-3.407848	4.597620
H	-15.429505	1.492914	2.930960
H	-14.876348	-4.196782	2.521244
H	-13.711996	-3.698904	6.846912
H	-14.913610	-2.972437	1.251126
H	-14.783528	0.546052	0.749605
H	-14.091590	-4.509206	0.975389
H	-13.738976	1.439464	3.426790
H	-12.773590	-2.212744	6.737010
H	-14.082612	-1.065874	0.588776
H	-12.343358	-3.597630	5.731764
H	-12.644701	-4.805253	3.667319
H	-12.707048	-0.096190	5.606385
H	-13.090994	0.267856	1.169617
H	-12.409147	-2.721387	1.423777
H	-11.881282	-5.045422	2.098881
H	-12.354047	2.523390	0.556704
H	-11.716174	1.051361	4.738105
H	-11.168124	-3.925383	3.271866
H	-10.730082	-1.304967	6.391176
H	-11.641133	2.374529	2.169285
H	-10.707965	0.384753	6.929351
H	-11.521720	3.897218	1.278890
H	-9.399986	-2.830584	5.507440
H	-10.895826	2.789143	-1.477371
H	-9.331958	-4.691435	2.059610
H	-8.767227	-5.516597	4.498672
H	-10.250162	1.260455	0.369607
H	-10.655673	4.712438	3.160756
H	-9.736942	1.914152	5.328875
H	-9.941225	4.091712	-0.775985
H	-8.662622	-6.178812	1.385731
H	-8.469321	-1.099617	7.094370
H	-8.234855	-7.005670	3.716403
H	-9.695012	3.855151	4.361305
H	-7.721191	-3.079694	5.904356
H	-8.052958	-4.607314	0.848312
H	-9.137734	2.646763	-1.389100
H	-9.451580	5.585766	4.106056
H	-7.149490	-6.152706	4.815086
H	-8.336488	1.512503	6.342314
H	-7.330409	-0.620652	5.820967
H	-9.516786	5.560156	0.958286
H	-7.825639	0.941633	-0.734114
H	-6.474896	-5.713799	2.466241
H	-7.628572	2.589635	4.266963
H	-6.810737	-2.192392	1.264970
H	-7.057173	0.937340	4.262144
H	-7.613930	4.373000	3.041792
H	-6.008083	-4.332281	5.540596
H	-6.868892	0.317232	0.615266
H	-8.386773	6.450540	1.974568
H	-7.776335	5.346656	0.740193
H	-7.153831	3.263261	-0.158980
H	-5.616557	-1.856053	5.842796
H	-5.850730	-4.397340	0.520613
H	-5.516617	-0.713613	2.821491
H	-6.056168	1.089945	-0.759818
H	-4.690321	-5.621183	3.848384
H	-5.612676	2.042887	2.199759
H	-4.803303	-3.065972	-0.012027
H	-4.252764	-2.851373	6.358653
H	-4.720893	-0.870253	1.249233
H	-5.766027	3.766804	1.845368
H	-4.182472	-1.978426	4.829844
H	-4.353944	-4.047191	1.371735
H	-4.104360	-1.763360	2.634570
H	-3.670396	-4.993966	5.144429
H	-3.668660	-4.204929	3.570170
H	-4.873852	2.715253	0.744921
H	-11.768303	-0.290038	-1.249517
H	-12.264348	-1.994591	-0.981907

Supplementary Table 25. Final coordinates and energy for Na-Product-in

Energy: -2226.573818 a.u

U	-11.214051	-4.320457	-0.146739
Si	-11.861492	-3.213393	-3.827250
Si	-11.477370	-1.907360	2.822642
Si	-10.469488	-7.958501	0.461656
N	-12.274771	-3.482780	-2.115732
N	-9.255046	-3.785371	-0.321310
N	-12.078978	-3.218786	1.780014
N	-13.845000	-4.834280	0.035776
N	-11.582814	-6.636004	0.024157
C	-13.199851	-5.670028	-4.787835
C	-9.051434	-3.133253	3.847458
C	-12.526591	0.118662	4.684907
C	-9.518355	0.260364	2.562414
C	-11.706616	-10.672589	0.637727
C	-7.948975	-8.266499	1.865630
C	-9.828526	-5.239264	-4.318755
C	-11.201805	-0.376525	-3.527550
C	-9.277060	-2.970905	-5.220468
C	-10.001754	-3.731537	-4.102857
C	-13.475700	-0.817701	-4.469676
C	-12.034240	-1.333065	-4.383505
C	-12.805520	-3.882156	-6.514584
C	-13.080418	-4.162135	-5.031781
C	-14.437715	-4.381915	-1.234992
C	-13.688164	-3.181494	-1.811258
C	-13.962745	-0.328066	2.664938
C	-12.959387	-0.930016	3.653490
C	-11.049447	-0.069190	0.595566
C	-10.319927	-0.761948	1.750471
C	-9.664434	-6.851677	3.014789
C	-9.096772	-7.271808	1.654745
C	-11.133707	-3.491669	5.221160
C	-10.375020	-2.520823	4.310853
C	-13.271690	-3.926786	2.271636
C	-14.338233	-4.054986	1.186369
C	-14.007252	-6.285539	0.240057
C	-12.899095	-7.060432	-0.465835
C	-12.459683	-9.013618	2.367487
C	-11.304885	-9.437174	1.450554
C	-8.492890	-7.724410	-1.635423
C	-9.568468	-8.683627	-1.112452
C	-10.549767	-9.018801	-2.241038
H	-13.507070	0.182551	-4.924604
H	-12.002463	0.961342	4.219558
H	-13.400463	0.538160	5.203069
H	-11.795254	0.645053	0.958224
H	-10.164099	1.038976	2.982874
H	-13.565113	0.575243	2.188597
H	-14.119856	-1.464878	-5.073739
H	-10.363457	0.533451	-0.026228
H	-14.890852	-0.032398	3.173539
H	-8.776068	0.775154	1.933252
H	-13.934059	-0.729773	-3.478243
H	-11.865552	-0.294117	5.454319
H	-11.324643	0.667792	-3.847340
H	-11.581674	-0.778958	-0.048828
H	-8.973131	-0.194492	3.394776
H	-14.230652	-1.025392	1.864992
H	-11.618172	-1.310452	-5.401339
H	-12.792880	-2.812621	-6.750704
H	-13.574250	-4.343468	-7.150269
H	-11.503277	-0.428871	-2.473842
H	-13.495880	-1.709121	4.215928
H	-14.063640	-3.725894	-4.797286
H	-13.773474	-2.341637	-1.099505
H	-9.614341	-1.501837	1.335986
H	-14.263719	-2.860000	-2.693206
H	-10.145138	-1.620671	4.899795
H	-10.129799	-0.597842	-3.613267
H	-11.842588	-4.300438	-6.832194
H	-14.584860	-3.049441	0.832435
H	-15.509385	-4.151701	-1.098273
H	-12.061628	-3.058705	5.612531
H	-14.083771	-6.080543	-5.295435
H	-9.292143	-1.882884	-5.093516
H	-8.428707	-2.416501	3.300731
H	-13.757766	-3.429481	3.125485
H	-13.285926	-5.913656	-3.724506
H	-10.523507	-3.786205	6.086515
H	-15.265378	-4.503872	1.584187
H	-8.459822	-3.494627	4.700685
H	-9.721377	-3.180616	-6.200379
H	-14.369433	-5.203489	-1.952895
H	-12.331663	-6.213857	-5.172656
H	-9.513836	-3.509133	-3.140164
H	-11.475000	-4.412062	4.688914
H	-9.217940	-3.985562	3.180710
H	-8.223167	-3.278157	-5.279848
H	-13.025732	-4.938480	2.640028
H	-10.208841	-5.546720	-5.299049
H	-15.010538	-6.610683	-0.087777
H	-10.349015	-5.831308	-3.560711
H	-13.926192	-6.484807	1.312445
H	-10.487753	-6.135804	2.919254
H	-13.001568	-6.913616	-1.555439
H	-8.767633	-5.520643	-4.283435

H	-8.891224	-6.386564	3.640432
H	-8.689771	-6.373354	1.166328
H	-8.915160	-6.737031	-1.855749
H	-13.099895	-8.132290	-0.307627
H	-10.042720	-7.717270	3.571902
H	-12.243660	-8.100616	2.929737
H	-7.676691	-7.572124	-0.922003
H	-13.378592	-8.835486	1.798066
H	-11.063393	-8.116064	-2.590721
H	-7.161671	-7.829526	2.495372
H	-8.044583	-8.102062	-2.565599
H	-7.476771	-8.585936	0.931529
H	-12.689361	-9.804095	3.096032
H	-8.294376	-9.172756	2.379224
H	-10.025687	-9.447631	-3.107045
H	-10.477860	-9.753882	2.105972
H	-11.318991	-9.736023	-1.938709
H	-9.075239	-9.614808	-0.794236
H	-12.546012	-10.466109	-0.037099
H	-10.881755	-11.061316	0.031745
H	-12.028973	-11.486525	1.303114
H	-8.285457	-4.097503	-0.217389
Na	-8.322234	-1.425771	-1.565330

Supplementary Table 26. Final coordinates and energy for 3Na

Energy: 2920.226801 a.u

U	-10.001190	-4.063743	-0.273548
Na	-6.117282	-2.373560	-0.383595
Si	-9.142664	-7.695028	0.542642
Si	-10.682772	-2.888274	-3.866865
Si	-10.317613	-1.581340	2.629361
N	-3.634226	-2.651996	1.830232
N	-4.319717	-0.207849	-1.801420
N	-2.251302	-2.397665	-0.969434
N	-3.831905	-4.698203	-2.374661
N	-7.938044	-3.872771	-0.669372
N	-10.270028	-6.397768	0.082936
N	-11.190988	-3.390076	-2.233827
N	-12.617318	-4.648458	0.013373
N	-10.908255	-2.933126	1.637131
C	-4.049000	-0.054290	-3.225685
C	-4.180031	-1.832898	2.907613
C	-3.733992	-4.061217	2.196983
C	-2.249723	-2.260885	1.561063
C	-1.643801	-2.843139	0.287899
C	-4.164251	-4.290927	-3.735100
C	-4.328873	-6.049434	-2.134904
C	-5.350424	0.743630	-1.399341
C	-3.126457	-0.004623	-0.979582
C	-1.972064	-0.975501	-1.230575
C	-1.791980	-3.243116	-2.080347
C	-2.391444	-4.646339	-2.129357
C	-10.169997	-0.158848	-3.074440
C	-6.419925	-7.994740	1.495101
C	-10.382143	-10.213798	1.583533
C	-9.823994	-9.362815	-1.780754
C	-8.582126	-4.819341	-4.392771
C	-12.018907	-5.221142	-5.083057
C	-11.176644	1.079477	3.517442
C	-8.989486	-3.238993	4.600543
C	-10.770912	-8.141077	2.949174
C	-9.809768	-8.852137	1.991061
C	-7.651073	-8.134432	-1.893478
C	-8.637782	-8.842449	-0.962057
C	-7.727240	-6.101116	2.496711
C	-7.493951	-6.933090	1.233925
C	-11.617882	-6.899939	-0.219454
C	-12.701921	-6.054687	0.439097
C	-8.054710	-2.462750	-5.103127
C	-8.804398	-3.334122	-4.090355
C	-11.327682	-3.382622	-6.667218
C	-11.802719	-3.714739	-5.248022
C	-11.182556	-2.287355	5.369661
C	-9.947224	-2.050523	4.493792
C	-12.293830	-0.470909	-4.376478
C	-10.856382	-0.962501	-4.180229
C	-12.639024	-3.242391	-2.029449
C	-13.242567	-4.439166	-1.301812
C	-13.171793	-3.739942	1.028427
C	-12.173196	-3.477803	2.148397
C	-12.262879	0.255726	1.395072
C	-11.654458	-0.151355	2.739656
C	-8.832089	-0.198682	0.557815
C	-8.638188	-0.969439	1.866590
C	-7.767499	-0.162726	2.835992
H	-2.029740	-2.727986	-3.015761
H	-0.690841	-3.357948	-2.068405
H	-1.838758	-5.210225	-2.906661
H	-2.197461	-5.170427	-1.186955
H	-0.563302	-2.599271	0.303696
H	-1.702688	-3.933840	0.335081
H	-1.590353	-2.554649	2.401232
H	-2.211601	-1.167267	1.517081
H	-1.125409	-0.629775	-0.615453
H	-1.634413	-0.860263	-2.265884
H	-2.721763	1.018519	-1.122090
H	-3.441358	-0.069903	0.067719
H	-5.049640	1.794757	-1.566786
H	-5.574142	0.630474	-0.333685

H	-6.269694	0.563007	-1.963845
H	-4.977670	-0.179751	-3.790234
H	-3.345351	-0.813892	-3.575612
H	-3.630884	0.938889	-3.475766
H	-3.676004	-4.924397	-4.499316
H	-3.871129	-3.253857	-3.917451
H	-5.245671	-4.358909	-3.884878
H	-4.137869	-6.344723	-1.098332
H	-3.860965	-6.802048	-2.796284
H	-5.408129	-6.082283	-2.303156
H	-4.777023	-4.314158	2.409789
H	-3.135541	-4.309393	3.093504
H	-3.398397	-4.707262	1.381014
H	-3.624904	-1.949851	3.856802
H	-5.221372	-2.109235	3.093123
H	-4.151618	-0.774578	2.630331
H	-7.624290	-4.516153	-1.372449
H	-10.590524	-10.830079	2.470314
H	-9.695839	-10.785175	0.949634
H	-11.329131	-10.114063	1.039635
H	-8.125346	-9.709027	-0.515510
H	-10.566751	-9.887898	-1.173178
H	-8.888051	-9.059593	2.557334
H	-9.487726	-10.062159	-2.559815
H	-6.720556	-8.689373	2.289841
H	-10.916621	-8.728171	3.867451
H	-6.188532	-8.598697	0.611189
H	-7.367763	-8.776518	-2.740216
H	-5.479790	-7.530510	1.827439
H	-10.337857	-8.539896	-2.289716
H	-11.759695	-8.010824	2.495980
H	-6.729582	-7.845891	-1.378953
H	-10.417567	-7.148191	3.243272
H	-8.092477	-6.717981	3.326926
H	-11.782096	-7.934607	0.125876
H	-8.095405	-7.223649	-2.312242
H	-7.133138	-6.246061	0.455096
H	-6.794891	-5.629459	2.839779
H	-7.513808	-5.078128	-4.353241
H	-11.809924	-6.936112	-1.305591
H	-8.454150	-5.299502	2.334443
H	-12.551473	-6.087301	1.521596
H	-9.103888	-5.466879	-3.680597
H	-13.708598	-6.459291	0.228054
H	-8.928759	-5.087137	-5.397654
H	-12.013544	-4.412302	2.714478
H	-6.992597	-2.746768	-5.149547
H	-9.464058	-4.158728	4.241846
H	-11.745027	-3.171916	5.049488
H	-8.383285	-3.141244	-3.091510
H	-11.100175	-5.789126	-5.261684
H	-13.057576	-5.332197	-1.906351
H	-8.452787	-2.568036	-6.119441
H	-8.680789	-3.415188	5.641058
H	-14.123617	-4.138850	1.424477
H	-10.892843	-2.458442	6.416523
H	-12.361169	-5.475216	-4.075427
H	-12.689645	-2.805555	2.855166
H	-8.079411	-3.095530	4.007603
H	-8.092002	-1.398391	-4.848052
H	-12.770879	-5.593384	-5.793702
H	-11.874792	-1.438008	5.364669
H	-14.337303	-4.331151	-1.194165
H	-13.392432	-2.785908	0.540974
H	-10.358769	-3.845811	-6.888383
H	-9.134533	-0.477424	-2.908731
H	-9.431718	-1.166007	4.897780
H	-13.204807	-3.151601	-2.971936
H	-8.100698	-1.898571	1.620756
H	-12.882447	-2.323957	-1.466254
H	-12.787668	-3.239763	-5.125407
H	-12.466515	-0.605067	3.328121
H	-10.698540	-0.281149	-2.122625
H	-12.037170	-3.756593	-7.419384
H	-11.221473	-2.304637	-6.834082
H	-10.315349	-0.779537	-5.120904
H	-12.568692	-0.615393	0.807429
H	-7.508203	-0.725197	3.738698
H	-9.469201	-0.734545	-0.151505
H	-10.155394	0.916526	-3.304301
H	-10.758852	0.824269	4.497806
H	-12.901204	-0.625329	-3.477211
H	-6.824430	0.140795	2.358860
H	-13.147747	0.892511	1.536888
H	-7.872227	-0.020452	0.055172
H	-12.801746	-0.973870	-5.206466
H	-11.554892	0.824339	0.782452
H	-8.263245	0.759286	3.159757
H	-9.285824	0.783860	0.732849
H	-12.002896	1.783240	3.692855
H	-10.406202	1.631161	2.965395
H	-12.314634	0.607145	-4.592911

Supplementary Table 27. Final coordinates and energy for Na-TS-Dis

Energy: -4453.133275 a.u

U -6.941702 -7.625179 -1.966565
U -1.351 -7.579919 2.982554
K -4.481153 -9.150469 1.136068
K -3.491404 -5.897253 -0.104031

Si -8.197874 -4.086882 -1.412078
Si -8.891961 -10.451257 -0.223696
Si -4.093094 -8.371396 -4.448061
Si -3.335618 -5.306477 5.019844
Si -0.938117 -11.393677 3.476251
Si 1.693385 -6.128003 0.702887
N -8.307706 -9.527413 -1.638244
N -5.706506 -7.770327 -3.980457
N -8.629537 -7.867884 -4.027009
N -8.329302 -5.737843 -2.074131
N -5.704090 -7.463942 -0.638971
N -1.756203 -5.970319 4.523095
N -2.250319 -7.619259 1.658476
N -0.791327 -9.693992 4.011258
N 0.732661 -7.419961 5.022965
N 1.129837 -6.950800 2.180901
C -8.364927 -4.575924 1.429271
C -10.234216 -2.137144 -2.382744
C -2.457914 -6.094451 -3.698173
C -6.151673 -2.336059 -0.338016
C -1.900715 -9.865695 -3.293033
C -2.926083 -9.633862 -6.847660
C -11.705979 -9.583952 -0.558622
C -6.560052 -12.188028 -0.036971
C -9.394837 -8.310826 1.678884
C -8.619708 -2.973106 -4.114040
C -8.817721 -2.668796 -2.625001
C -5.448728 -3.731982 -2.299967
C -6.330522 -3.682349 -1.049614
C -10.570382 -4.435645 0.246811
C -9.134827 -3.899754 0.289816
C -8.546426 -13.219373 -1.159869
C -8.088016 -12.232820 -0.080232
C -8.203631 -10.300288 2.627959
C -8.413498 -9.447260 1.371318
C -11.330864 -11.634490 0.849263
C -10.813650 -10.808330 -0.334649
C -4.163265 -10.394128 -2.333632
C -3.285402 -9.306109 -2.950148
C -4.814890 -10.894246 -5.765470
C -4.225387 -9.506265 -6.042177
C -9.478753 -9.046961 -3.765052
C -8.731253 -10.083511 -2.933785
C -3.358503 -6.034845 -6.039308
C -2.850174 -6.939057 -4.912203
C -6.505644 -7.262596 -5.107134
C -7.806400 -8.042789 -5.241388
C -9.423733 -6.624052 -4.096373
C -9.624025 -6.029605 -2.708116
C 1.957192 -11.991422 3.734344
C -3.825556 -11.604101 3.773499
C 0.248049 -11.182845 0.830939
C -3.835668 -3.461210 2.830193
C -6.146289 -5.886698 4.614433
C -4.519145 -4.225937 7.495726
C 0.558273 -4.546371 -1.449486
C 3.310679 -8.413480 -0.112316
C -4.628805 -7.892268 4.605435
C -4.745317 -6.405045 4.272023
C -3.518017 -6.529047 7.677005
C -3.430722 -5.171318 6.972113
C -2.580534 -2.498421 4.782761
C -3.655425 -3.500669 4.349905
C 0.178690 -6.433593 5.973531
C -0.606405 -5.369849 5.218402
C -2.501084 -12.498121 5.705807
C -2.524953 -12.294312 4.187343
C -1.779036 -12.651926 0.969627
C -1.092055 -11.403493 1.538851
C 1.147619 -8.095810 -1.334300
C 2.319180 -7.379232 -0.656282
C 0.411603 -13.972133 3.782583
C 0.559 -12.482951 4.122121
C -0.469981 -9.535097 5.438792
C 0.831924 -8.764522 5.626150
C 2.027443 -6.963699 4.476559
C 2.212584 -7.447621 3.044054
C -0.368975 -4.078505 0.831242
C 0.203273 -5.159323 -0.089930
C 4.526928 -5.202649 0.662800
C 3.091838 -4.776493 0.991881
C 3.041924 -4.098333 2.364780
H -10.429264 -1.256701 -3.011419
H -10.396553 -1.833139 -1.343801
H -11.452 -2.880128 -2.635073
H -9.179885 -2.819450 0.496947
H -11.182849 -3.955846 -0.522545
H -8.129963 -1.846391 -2.372979
H -11.076145 -4.278027 1.209879
H -6.473158 -1.500729 -0.972197
H -8.739942 -2.062038 -4.716728
H -6.716009 -2.269354 0.597183
H -8.934640 -4.536218 2.368210
H -5.095359 -2.152661 -0.095977
H -10.583198 -5.513871 0.052734
H -9.363625 -3.690297 -4.477875
H -7.396872 -4.102463 1.618831
H -7.631321 -3.386352 -4.336500
H -5.741432 -2.961106 -3.022636
H -10.258333 -5.136750 -2.826993

H -8.171841 -5.631522 1.207451
H -6.018544 -4.483279 -0.363008
H -4.392892 -3.529210 -2.058667
H -9.467 -7.668243 2.476393
H -10.228683 -6.727715 -2.104837
H -5.497823 -4.696630 -2.816625
H -8.866559 -5.900892 -4.697317
H -9.579764 -7.674805 0.808295
H -10.382428 -6.817070 -4.606514
H -10.360635 -8.695762 2.023934
H -6.745624 -6.193412 -4.982073
H -7.901593 -9.673723 3.478902
H -3.346738 -5.625893 -3.256742
H -4.227074 -5.448642 -5.717533
H -7.459759 -8.966244 1.153
H -11.794015 -8.971179 0.344469
H -10.351577 -8.715798 -3.196928
H -9.127514 -10.808376 2.928478
H -1.770843 -5.285199 -3.980640
H -8.382924 -7.745837 -6.134101
H -2.585050 -5.319176 -6.351318
H -11.325240 -8.934291 -1.352356
H -5.990155 -7.317949 -6.077529
H -1.954956 -6.693087 -2.929849
H -7.440477 -11.075710 2.505003
H -12.724250 -9.887384 -0.839211
H -3.655398 -6.599555 -6.930403
H -9.843022 -9.469413 -4.716647
H -7.559469 -9.104260 -5.340318
H -11.275232 -11.070853 1.788226
H -6.194121 -11.563789 0.787315
H -1.944187 -7.446904 -5.273765
H -9.397383 -10.955476 -2.840324
H -3.153900 -8.529108 -2.182413
H -7.868838 -10.450404 -3.517887
H -10.908190 -11.439113 -1.231690
H -4.943932 -8.981125 -6.688593
H -6.150639 -11.790416 -0.970449
H -12.385601 -11.907924 0.705900
H -10.773737 -12.566496 0.991765
H -8.438171 -12.619929 0.888395
H -5.729259 -10.850330 -5.163524
H -1.227545 -9.107273 -3.705914
H -5.137998 -10.008322 -2.016373
H -6.131560 -13.189387 0.110836
H -2.537238 -8.664506 -7.176422
H -8.202110 -12.916060 -2.155232
H -1.413208 -10.282021 -2.401579
H -5.063868 -11.409352 -6.703967
H -3.673681 -10.839975 -1.454363
H -9.636095 -13.321459 -1.204247
H -4.102471 -11.536234 -5.234476
H -1.966429 -10.678232 -4.025754
H -4.352581 -11.215840 -3.031167
H -3.090361 -10.237790 -7.751052
H -2.131620 -10.127546 -6.276768
H -8.136677 -14.222131 -0.973136
H 5.208563 -4.341107 0.704156
H 4.616859 -5.634378 -0.339352
H 4.910140 -5.941340 1.376845
H 2.839363 -6.771890 -1.412877
H 4.186508 -7.958707 0.360574
H 2.816677 -4.011864 0.248743
H 3.676305 -9.066755 -0.916832
H 1.346133 -3.788455 -1.357221
H 3.652616 -3.184599 2.375284
H 0.906984 -5.287759 -2.174798
H 1.505591 -8.852479 -2.046362
H -0.309842 -4.038504 -1.892674
H 2.833018 -9.059626 0.632133
H 3.440214 -4.753056 3.147205
H 0.502490 -7.408856 -1.890399
H 2.026584 -3.816808 2.661000
H 0.368078 -3.289916 1.025236
H 3.214042 -7.122507 2.718884
H 0.519600 -8.610122 -0.598757
H -0.564752 -5.932472 -0.247654
H -1.234138 -3.573223 0.373280
H 0.102105 -11.032377 -0.246480
H 2.259165 -8.549793 3.044841
H -0.685250 -4.471438 1.803468
H 2.021296 -5.870819 4.466801
H 0.782910 -10.308041 1.213940
H 2.852035 -7.288598 5.133266
H 0.905832 -12.051407 0.944077
H 0.073976 -4.866007 4.510584
H -1.854609 -12.589930 -0.124652
H -2.925984 -3.808806 2.325040
H -1.611893 -2.728756 4.324070
H -1.715440 -10.523475 1.311903
H 2.177768 -12.185157 2.680149
H 1.628781 -9.308448 5.113707
H -1.210619 -13.562018 1.192514
H -4.031674 -2.438320 2.480091
H 0.988593 -6.001796 6.585308
H -2.845419 -1.476109 4.478276
H 2.083635 -10.917053 3.896019
H -0.883489 -4.586169 5.939123
H -4.677393 -4.082274 2.501900
H -2.791014 -12.803483 1.358459

H	2.730126	-12.506012	4.321933
H	-2.433257	-2.483125	5.868749
H	1.115349	-8.683179	6.689037
H	-0.510507	-6.951443	6.646804
H	0.506865	-14.153269	2.705299
H	-3.920159	-11.553073	2.681938
H	-4.606403	-3.188267	4.805472
H	-0.362217	-10.491353	5.972927
H	-4.602636	-6.304163	3.185181
H	-1.274795	-9.002677	5.974400
H	0.486204	-12.398930	5.217350
H	-2.466059	-4.724177	7.255644
H	-3.875415	-10.587170	4.175802
H	1.198064	-14.563281	4.272293
H	-0.549129	-14.388473	4.103063
H	-2.514976	-13.290254	3.719787
H	-2.789950	-7.248622	7.286926
H	-6.271872	-4.822710	4.389287
H	-3.642534	-8.297808	4.351695
H	-4.706946	-12.146968	4.142874
H	-4.417967	-3.209391	7.102331
H	-2.559915	-11.544376	6.243073
H	-6.917307	-6.429617	4.051935
H	-3.335883	-6.427730	8.755806
H	-5.389656	-8.476250	4.065209
H	-1.596562	-13.014095	6.045227
H	-4.513166	-6.975928	7.565271
H	-6.373771	-6.025284	5.677628
H	-4.794790	-8.088992	5.669678
H	-4.475111	-4.151931	8.591498
H	-5.526173	-4.575133	7.241902
H	-3.358637	-13.101727	6.035148

Supplementary Table 28. Final coordinates and energy for Na-Product-Dis

Energy: -4453.134846 a.u

U	-11.253387	-4.498721	-0.286183
U	-4.926128	-2.201967	-0.085773
Na	-7.837987	-1.812629	-2.291582
Na	-8.065537	-5.075390	2.065278
Si	-10.851926	-8.271230	0.522231
Si	-11.836694	-2.054992	2.541544
Si	-5.512173	1.165478	-2.190062
Si	-4.024394	-5.533216	-1.831459
Si	-12.157714	-3.509432	-3.913614
Si	-4.170121	-1.585482	3.708488
N	-11.619335	-6.799471	-0.137693
N	-13.940249	-4.998149	-0.082967
N	-12.127959	-3.581839	1.648913
N	-9.106724	-4.131350	-0.472620
N	-6.804558	-2.871646	-0.020130
N	-3.872428	-1.435105	1.970219
N	-2.277188	-1.549565	-0.516532
N	-4.780205	-0.387756	-1.734123
N	-12.380174	-3.606270	-2.139857
N	-3.685559	-4.106413	-0.837366
C	-12.706541	-8.954070	2.744150
C	-11.997071	-9.526313	1.514066
C	-10.012682	-9.382376	-0.858509
C	-8.600452	-8.918767	-1.229607
C	-12.883765	-7.069769	-0.853744
C	-14.072811	-6.465912	-0.116270
C	-14.394462	-4.416801	1.192646
C	-13.239568	-4.388420	2.185401
C	-10.995922	-2.381214	4.270723
C	-11.829441	-3.264931	5.204097
C	-9.431654	-7.729830	1.737850
C	-9.853671	-6.668885	2.755889
C	-12.982862	-10.357430	0.684122
C	-10.603349	-0.952475	1.531940
C	-11.030217	-0.634961	0.095701
C	-13.522299	-1.135792	2.938757
C	-14.160511	-0.436680	1.733077
C	-2.847000	-0.477244	1.655351
C	-1.724604	-1.097112	0.769149
C	-2.285911	-0.492761	-1.540176
C	-3.508177	-0.621765	-2.448740
C	-4.295965	2.702427	-2.038008
C	-4.885172	3.999719	-2.608448
C	-13.717277	-3.162940	-1.699005
C	-14.565364	-4.347946	-1.249908
C	-2.292741	-4.760000	-0.380613
C	-1.613164	-2.776764	-0.985771
C	-6.057894	1.240236	-4.083683
C	-4.914653	1.360375	-5.099768
C	-7.117142	1.413189	-1.108866
C	-8.209745	2.261787	-1.769384
C	-4.235687	-7.138447	-0.732871
C	-2.959444	-7.494694	0.038388
C	-5.702176	-5.279021	-2.781033
C	-5.755798	-4.006853	-3.632226
C	-2.554563	-5.962078	-3.061727
C	-2.411961	-4.989174	-4.238208
C	-13.695022	-4.251113	-4.878872
C	-13.837239	-3.732157	-6.316302
C	-12.80600	-1.668840	-4.557496
C	-13.207415	-0.785347	-4.223058
C	-10.489127	-4.395207	-4.358692
C	-10.137209	-4.298063	-5.847738

C	-2.573165	-1.864421	4.828965	H	-15.034360	-6.760192	-0.569529
C	-2.021014	-0.633511	5.557521	H	-11.085825	-6.493769	-4.437679
C	-5.286705	-3.150892	4.036026	H	-12.915384	-5.422451	2.383589
C	-4.649622	-4.449537	3.534337	H	-9.137185	-4.708114	-6.043402
C	-5.148274	-0.066182	4.460429	H	-9.640644	-3.952351	3.668581
C	-6.630492	-0.090587	4.074546	H	-11.896088	-4.290216	4.822219
C	-10.713953	-0.986095	-4.096110	H	-9.727096	-3.828793	-3.799669
C	-10.399201	-5.847478	-3.883241	H	-13.021717	-6.222119	-5.540864
C	-8.790922	-8.926262	2.450956	H	-14.604183	-5.070822	-2.069976
C	-10.846382	-9.517990	-2.138027	H	-10.840051	-4.873229	-6.461124
C	-10.278140	0.345461	2.280343	H	-9.106748	-3.079376	5.108745
C	-13.441508	-0.162833	4.122939	H	-15.267739	-4.965937	1.584045
C	-9.586851	-2.957962	4.128570	H	-11.374386	-3.323890	6.202731
C	-6.974000	0.080129	-4.484180	H	-13.618272	-6.212396	-3.879369
C	-6.806753	1.962143	0.287075	H	-13.628637	-4.022003	3.147340
C	-5.691150	-3.298834	5.506556	H	-8.937694	-2.321763	3.517023
C	-6.093881	-6.496065	-3.624140	H	-10.145871	-3.267618	-6.218956
C	-2.557901	-7.406891	-3.577878	H	-14.752050	-6.125476	-5.231754
C	-5.400184	-7.002733	0.249625	H	-12.852621	-2.896274	5.337078
C	-13.771757	-5.781707	-4.873998	H	-15.603048	-4.052614	-1.019746
C	-3.700743	2.975997	-0.652357	H	-14.709711	-3.385455	1.008066
C	-4.546055	1.289027	4.072774	H	-12.984326	-4.016880	-6.943144
C	-1.430478	-2.607609	4.129644	H	-9.826404	-1.523705	-4.450884
H	-8.727747	-4.864173	-1.094270	H	-10.908154	-1.388888	4.735273
H	-13.446877	-11.132579	1.309885	H	-14.276417	-2.616656	-2.472907
H	-12.500372	-10.871299	-0.152495	H	-9.674626	-1.539718	1.470380
H	-13.801481	-9.751873	0.278520	H	-13.634543	-2.454667	-0.858678
H	-9.928176	-10.381433	-0.402991	H	-14.567597	-3.879945	-4.320100
H	-11.848263	-9.920775	-1.961473	H	-14.204026	-1.945541	3.238977
H	-11.238184	-10.235144	1.882895	H	-10.688868	-0.912864	-3.944000
H	-10.351073	-10.189313	-2.853220	H	-14.734660	-4.153336	-6.790402
H	-9.484099	-9.381002	3.168040	H	-13.931369	-2.643587	-6.365489
H	-13.458666	-8.204759	2.474240	H	-11.946841	-1.760421	-5.652179
H	-8.476874	-9.712735	1.756564	H	-14.202829	-1.077389	0.845955
H	-8.169262	-9.567365	-2.004764	H	-9.925600	0.167385	3.301207
H	-7.901466	-8.620538	3.019633	H	-11.268103	-1.526033	-0.498602
H	-10.964949	-8.549422	-2.635945	H	-10.641959	0.038045	-4.486446
H	-12.015459	-8.484283	3.449379	H	-13.089522	-0.641370	5.041885
H	-7.911754	-8.935201	-0.379764	H	-13.266003	-0.582801	-3.147710
H	-13.231617	-9.748827	3.292438	H	-9.491910	0.907249	1.761851
H	-10.645003	-7.026945	3.420509	H	-15.189448	-0.125137	1.960454
H	-13.073968	-8.140173	-1.007117	H	-10.230637	-0.090196	-0.427090
H	-8.606780	-7.900313	-1.636592	H	-14.158674	-1.236056	-4.526631
H	-8.668345	-7.292364	1.075067	H	-13.610765	0.470344	1.456160
H	-9.019923	-6.395626	3.423273	H	-11.153560	1.002463	2.342327
H	-9.389537	-6.249365	-4.034685	H	-11.916579	0.006509	0.061722
H	-12.859973	-6.643451	-1.870972	H	-14.431137	0.262643	4.340085
H	-10.242419	-5.756200	2.286831	H	-12.772196	0.680215	3.917389
H	-14.064818	-6.837725	0.911767	H	-13.134010	0.187470	-4.728782
H	-10.637611	-5.964139	-2.819759	H	-7.982987	-3.458476	-0.209376

H	-1.214991	-0.921938	6.247786
H	-2.782178	-0.118276	6.152225
H	-1.592587	0.096997	4.860976
H	-5.078947	-0.169163	5.554361
H	-3.496246	1.390717	4.363126
H	-2.959031	-2.536745	5.611141
H	-5.096363	2.112883	4.549341
H	-4.820530	-3.498324	6.142906
H	-0.688281	-2.960669	4.859749
H	-6.183575	-2.405645	5.903408
H	-7.153197	0.793850	4.465888
H	-6.389000	-4.144034	5.649597
H	-4.603311	1.449409	2.990383
H	-0.899624	-1.951379	3.432050
H	-7.151244	-0.971005	4.463338
H	-1.772650	-3.477383	3.561091
H	-3.728377	-4.674902	4.083471
H	-2.283433	-0.086344	2.548984
H	-6.759005	-0.085390	2.985518
H	-6.200398	-2.946185	3.455688
H	-5.305027	-5.322284	3.696748
H	-7.700778	1.944456	0.923473
H	-3.192262	0.420157	1.147300
H	-4.381253	-4.406517	2.472318
H	-1.324420	-1.971913	1.288200
H	-6.028388	1.386894	0.797158
H	-0.885861	-0.396959	0.605008
H	-6.474131	3.004161	0.238584
H	-2.238316	-3.915398	0.718141
H	-9.093819	2.337448	-1.121311
H	-5.183692	-6.182733	0.946119
H	-2.715317	-6.718467	0.773477
H	-7.514358	0.398060	-0.951451
H	-4.418264	3.473340	0.007885
H	-2.335206	0.475837	-1.036597
H	-7.865174	3.286111	-1.951848
H	-5.537231	-7.916688	0.844855
H	-0.532730	-2.742476	-0.756563
H	-3.078534	-8.436805	0.592729
H	-3.379445	2.063344	-0.142697
H	-1.675027	-4.880362	-0.622826
H	-6.345738	-6.790802	-0.262841
H	-8.543923	1.859865	-2.731402
H	-2.826845	3.638204	-0.727106
H	-2.086737	-7.612135	-0.613136
H	-1.346813	-0.512446	-2.122281
H	-1.716763	-2.834186	-2.073301
H	-5.714231	4.368929	-1.992707
H	-7.903741	0.087858	-3.909473
H	-4.462298	-7.965340	-1.421005
H	-3.349144	0.064843	-3.295131
H	-6.444255	-5.156875	-1.977676
H	-3.489602	-1.627542	-2.904245
H	-3.456012	2.416705	-2.690903
H	-1.649362	-5.850867	-2.446510
H	-6.463017	-0.883944	-4.344765
H	-4.128648	4.796874	-2.626992
H	-5.260474	3.888761	-3.630654
H	-6.652267	2.163038	-4.154794
H	-2.464081	-3.940732	-3.926348
H	-6.089874	-7.428911	-3.050895
H	-5.509512	-3.108596	-3.053552
H	-7.263043	0.138919	-5.542048
H	-2.554769	-8.146153	-2.770768
H	-4.353625	0.423024	-5.188894
H	-7.101802	-6.379227	-4.045168
H	-1.451742	-5.131386	-4.753911
H	-6.756724	-3.876403	-4.075136
H	-4.198736	2.148942	-4.846608
H	-3.196177	-5.147251	-4.988303
H	-5.414257	-6.630579	-4.473290
H	-5.054819	-4.043364	-4.471924
H	-1.668224	-7.599561	-4.194250
H	-3.430016	-7.616352	-4.207056
H	-5.306616	1.589687	-6.101117

Supplementary Table 29. Final coordinates and energy for 4Na

Energy: -4451.95650 a.u

U	-6.941702	-7.625179	-1.966565
U	-0.546191	-7.549940	3.151874
K	-3.755374	-9.438202	1.916452
K	-3.515648	-5.770153	-0.330502
Si	-8.197874	-4.086882	-1.412078
Si	-8.891961	-10.451257	-0.223696
Si	-4.093094	-8.371396	-4.448061
Si	-2.866578	-5.276695	5.206315
Si	-0.447573	-11.358821	3.676331
Si	2.100787	-6.097459	0.818431
N	-8.307706	-9.527413	-1.638244
N	-5.706506	-7.770327	-3.980457
N	-8.629537	-7.867884	-4.027009
N	-8.329302	-5.737843	-2.074131
N	-5.704090	-7.463942	-0.638971
N	-1.290117	-5.933007	4.690541
N	-1.816250	-7.609725	1.847831
N	-0.305293	-9.653633	4.194788
N	1.217060	-7.359912	5.163596
N	1.566484	-6.911875	2.311868
C	-8.364927	-4.575924	1.429271

C	-10.234216	-2.137144	-2.382744
C	-2.457914	-6.094451	-3.698173
C	-6.151673	-2.336059	-0.338016
C	-1.900715	-9.865695	-3.293033
C	-2.926083	-9.633862	-6.847660
C	-11.705979	-9.583952	-0.558622
C	-6.560052	-12.188028	-0.036971
C	-9.394837	-8.310826	1.678884
C	-8.619708	-2.973106	-4.114040
C	-8.817721	-2.668796	-2.625001
C	-5.448728	-3.731982	-2.299967
C	-6.330522	-3.682349	-1.049614
C	-10.570382	-4.435645	0.246811
C	-9.134827	-3.899754	0.289816
C	-8.546426	-13.219373	-1.159869
C	-8.088016	-12.232820	-0.080232
C	-8.203631	-10.300288	2.627959
C	-8.413498	-9.447260	1.371318
C	-11.330864	-11.634490	0.849263
C	-10.813650	-10.808330	-0.334649
C	-4.163265	-10.394128	-2.333632
C	-3.285402	-9.306109	-2.950148
C	-4.814890	-10.894246	-5.765470
C	-4.225387	-9.506265	-6.042177
C	-9.478753	-9.046961	-3.765052
C	-8.731253	-10.083511	-2.933785
C	-3.358503	-6.034845	-6.039308
C	-2.850174	-6.939057	-4.912203
C	-6.505644	-7.262596	-5.107134
C	-7.806400	-8.042789	-5.241388
C	-9.423733	-6.624052	-4.096373
C	-9.624025	-6.029605	-2.708116
C	2.455815	-11.932919	3.894200
C	-3.328379	-11.588087	4.020332
C	0.695725	-11.161595	1.011170
C	-3.414472	-3.453762	3.009417
C	-5.678759	-5.881108	4.849654
C	-4.019597	-4.184044	7.691260
C	0.920490	-4.542529	-1.329088
C	3.722386	-8.377717	-0.002812
C	-4.146536	-7.875397	4.833711
C	-4.279412	-6.391950	4.489765
C	-2.998456	-6.478067	7.876102
C	- 2.932352	-5.125739	7.158634
C	-2.136438	-2.465233	4.934069
C	-3.210346	-3.479033	4.526408
C	0.670512	-6.369667	6.114429
C	-0.134209	-5.318186	5.362793
C	-1.967348	-12.455908	5.939132
C	-2.016341	-12.265129	4.419578
C	-1.317838	-12.644412	1.193712
C	-0.631517	-11.386160	1.741000
C	1.538269	-8.086408	-1.193510
C	2.714794	-7.355472	-0.539851
C	0.926123	-13.924535	3.983064
C	1.067558	-12.431485	4.307829
C	0.036988	-9.480298	5.615761
C	1.335806	-8.698552	5.776352
C	2.499723	-6.898717	4.593271
C	2.666223	-7.393353	3.162138
C	0.025293	-4.062267	0.961846
C	0.591283	-5.146572	0.040905
C	4.926315	-5.151533	0.726428
C	3.493343	-4.733241	1.074291
C	3.459661	-4.043883	2.442098
H	-10.429264	-1.256701	-3.011419
H	-10.396553	-1.833139	-1.343801
H	-11.452000	-2.880128	-2.635073
H	-9.179885	-2.819450	0.496947
H	-11.182849	-3.955846	-0.522545
H	-8.129963	-1.846391	-2.372979
H	-11.076145	-4.278027	1.209879
H	-6.473158	-1.500729	-0.972197
H	-8.739942	-2.062038	-4.716728
H	-6.716009	-2.269354	0.597183
H	-8.934640	-4.536218	2.368210
H	-5.095359	-2.152661	-0.095977
H	-10.583198	-5.513871	0.052734
H	-9.363625	-3.690297	-4.477875
H	-7.396872	-4.102463	1.618831
H	-7.631321	-3.386352	-4.336500
H	-5.741432	-2.961106	-3.022636
H	-10.258333	-5.136750	-2.826993
H	-8.171841	-5.631522	1.207451
H	-6.018544	-4.483279	-0.363008
H	-4.392892	-3.529210	-2.058667
H	-9.467000	-7.668243	2.476393
H	-10.228683	-6.727715	-2.104837
H	-5.497823	-4.696630	-2.816625
H	-8.866559	-5.900892	-4.697317
H	-9.579764	-7.674805	0.808295
H	-10.382428	-6.817070	-4.606514
H	-10.360635	-8.695762	2.023934
H	-6.745624	-6.193412	-4.982073
H	-7.901593	-9.673723	3.478902
H	-3.346738	-5.625893	-3.256742
H	-4.227074	-5.448642	-5.717533
H	-7.459759	-8.966244	1.153000
H	-11.794015	-8.971179	0.344469
H	-10.351577	-8.715798	-3.196928

H	-9.127514	-10.808376	2.928478	H	1.890823	-8.846402	-1.904734
H	-1.770843	-5.285199	-3.980640	H	0.041793	-4.044866	-1.762899
H	-8.382924	-7.745837	-6.134101	H	3.261229	-9.021061	0.754360
H	-2.585050	-5.319176	-6.351318	H	3.874979	-4.689001	3.223648
H	-11.325240	-8.934291	-1.352356	H	0.879422	-7.408975	-1.745187
H	-5.990155	-7.317949	-6.077529	H	2.446939	-3.767393	2.750764
H	-1.954956	-6.693087	-2.929849	H	0.759312	-3.2666334	1.137734
H	-7.440477	-11.075710	2.505003	H	3.660031	-7.063598	2.818684
H	-12.724250	-9.887384	-0.839211	H	0.925646	-8.599115	-0.443995
H	-3.655398	-6.599555	-6.930403	H	-0.173254	-5.926690	-0.098364
H	-9.843022	-9.469413	-4.716647	H	-0.850661	-3.567293	0.513238
H	-7.559469	-9.104260	-5.340318	H	0.531929	-11.021328	-0.065058
H	-11.275232	-11.070853	1.788226	H	2.721110	-8.495104	3.171391
H	-6.194121	-11.563789	0.787315	H	-0.272868	-4.44929	1.942131
H	-1.944187	-7.446904	-5.273765	H	2.485194	-5.806034	4.574495
H	-9.397383	-10.955476	-2.840324	H	1.229869	-10.279649	1.378471
H	-3.153900	-8.529108	-2.182413	H	3.336842	-7.211940	5.239721
H	-7.868838	-10.450404	-3.517887	H	1.361709	-12.024277	1.121273
H	-10.908190	-11.439113	-1.231690	H	0.531277	-4.815327	4.640270
H	-4.943932	-8.981125	-6.688593	H	1.410869	-12.592233	0.100266
H	-6.150639	-11.790416	-0.970449	H	-2.510155	-3.798876	2.493051
H	-12.385601	-11.907924	0.705900	H	-1.173334	-2.692264	4.462260
H	-10.773737	-12.566496	0.991765	H	-1.264963	-10.512730	1.517190
H	-8.438171	-12.619929	0.888395	H	2.661437	-12.133925	2.838346
H	-5.729259	-10.850330	-5.163524	H	2.128679	-9.240880	5.256095
H	-1.227545	-9.107273	-3.705914	H	-0.739188	-13.548355	1.415287
H	-5.137998	-10.008322	-2.016373	H	-3.623592	-2.435348	2.653896
H	-6.131560	-13.189387	0.110836	H	1.486548	-5.926730	6.709874
H	-2.537238	-8.664506	-7.176422	H	-2.413714	-1.447521	4.625237
H	-8.202110	-12.916060	-2.155232	H	2.576660	-10.856315	4.044918
H	-1.413208	-10.282021	-2.401579	H	-0.405955	-4.530511	6.081188
H	-5.063868	-11.409352	-6.703967	H	-4.256495	-4.083792	2.69948
H	-3.673681	-10.839975	-1.454363	H	-2.322482	-12.800160	1.599534
H	-9.636095	-13.321459	-1.204247	H	3.241639	-12.436788	4.473932
H	-4.102471	-11.536234	-5.234476	H	-1.972424	-2.439664	6.017462
H	-1.966429	-10.678232	-4.025754	H	1.635091	-8.606129	6.833973
H	-4.352581	-11.215840	-3.031167	H	-0.0042204	-6.886891	6.802667
H	-3.090361	-10.237790	-7.751052	H	1.005997	-14.114065	2.905973
H	-2.131620	-10.127546	-6.276768	H	-3.440312	-11.546993	2.929992
H	8.136677	-14.222131	-0.973136	H	-4.156457	-3.169836	4.994134
H	5.602003	-4.284651	0.749959	H	0.160236	-10.431179	6.156106
H	5.003913	-5.591044	-0.273368	H	-4.154399	-6.299209	3.426073
H	5.326128	-5.881303	1.440544	H	-0.763393	-8.949338	6.159399
H	3.218569	-6.750717	-1.309508	H	1.011158	-12.338746	5.403322
H	4.602007	-7.912492	0.452547	H	-1.966795	-4.669089	7.423343
H	3.200918	-3.976981	0.329185	H	-3.379638	-10.568187	4.414869
H	4.080377	-9.035048	-0.807457	H	1.724528	-14.505685	4.465359
H	1.703958	-3.778050	-1.255449	H	-0.026350	-14.345249	4.321954
H	4.063543	-3.125599	2.435453	H	-2.006130	-13.264889	3.960246
H	1.263463	-5.287423	-2.053543	H	-2.271139	-7.195507	7.480727

H	-5.815834	-4.820020	4.617628
H	-3.161301	-8.275759	4.568007
H	-4.199810	-12.134319	4.407927
H	-3.932202	-3.170140	7.287870
H	-2.025005	-11.498115	6.469277
H	-6.454313	-6.434455	4.303800
H	-2.800350	-6.366285	8.951048
H	-4.911270	-8.469542	4.310310
H	-1.053801	-12.962285	6.268692
H	-3.991827	-6.933229	7.783637
H	-5.888644	-6.012378	5.917387
H	-4.294479	-8.064337	5.902018
H	-3.959101	-4.100450	8.785557
H	-5.027788	-4.542817	7.456098
H	-2.815110	-13.063037	6.286834

Supplementary Table 30. Final coordinates and energy for 6Na

Energy: -2919.628584 a.u

U	-3.594475	4.970237	-7.809619
Na	-1.677592	1.080140	-5.627226
Si	-1.514443	7.190107	-5.493657
Si	-7.273476	4.479638	-6.901688
Si	-2.219557	3.562344	-11.058483
N	-5.964424	5.150893	-7.912246
N	-2.801577	4.905866	-10.049178
N	-4.329196	7.193619	-9.169209
N	-2.777934	6.938473	-6.725229
N	-2.911951	3.437338	-6.862279
N	-3.175102	-0.938621	-6.101463
N	1.285033	0.602970	-5.622949
N	-0.804889	-1.194123	-4.098538
N	-1.976616	1.200866	-2.460594
C	0.905833	8.772000	-5.068350
C	-2.650022	9.287948	-3.745855
C	0.022168	5.373522	-3.776132
C	-4.444509	1.808926	-10.450338
C	-5.845895	4.249232	-4.390077
C	-7.647216	7.036232	-5.471551
C	-9.460027	4.013451	-8.845820
C	-4.172617	4.284203	-13.156501
C	0.232983	2.806031	-9.728971
C	-3.321491	6.919846	-3.250937
C	-2.201720	7.823002	-3.771933
C	0.344985	5.097670	-6.254650
C	-0.642588	5.487006	-5.151657
C	0.287146	8.403739	-7.487079
C	-0.244701	8.570750	-6.061296
C	-9.576002	5.419325	-5.351218
C	-8.402226	5.921224	-6.200459
C	-7.787960	2.162637	-8.570404
C	-8.513788	3.311078	-7.866105
C	-7.408544	2.346009	-4.898596
C	-6.473308	3.401309	-5.499581
C	-1.788926	4.216766	-13.947270
C	-2.794186	3.647631	-12.939745
C	0.432893	4.759771	-11.275676
C	-0.268871	3.417867	-11.040120
C	-5.789237	7.342944	-9.033472
C	-6.476497	5.985201	-9.006863
C	-2.311196	0.664892	-11.113991
C	-2.916348	1.875394	-10.393183
C	-2.650542	6.217048	-10.686729
C	-3.944515	7.009854	-10.581261
C	-3.617046	8.340689	-8.578524
C	-3.465314	8.187949	-7.072567
C	-2.693113	-1.063626	-7.472841
C	-4.618505	-0.725074	-6.111410
C	-2.837604	-2.113921	-5.353000
C	-1.358385	-2.251866	-4.950514
C	-3.118952	2.092417	-2.287057
C	2.003528	1.404757	-4.637884
C	1.830799	0.863257	-6.952041
C	1.383113	-0.827342	-5.327922
C	0.660816	-1.289053	-4.064746
C	-1.376051	-1.241715	-2.742250
C	-2.438564	-0.187209	-2.434330
C	-0.985198	1.476794	-1.428501
H	1.241315	5.729299	-6.236459
H	1.019636	-0.691977	-3.221629
H	0.990439	-2.326738	-3.863323
H	2.443540	-1.131124	-5.221034
H	1.003750	-1.371571	-6.199701
H	-1.229226	-3.242372	-4.471221
H	-0.777890	-2.289555	-5.876342
H	-3.141215	-3.045593	-5.816407
H	-3.428785	-2.077074	-4.378760
H	-1.833052	-2.226090	-2.550208
H	-0.568468	-1.157601	-2.007668
H	-2.872455	-0.437455	-1.444647
H	-3.253141	-0.274000	-3.161680
H	-3.620236	1.960301	-1.309902
H	-3.863290	1.908282	-3.067845
H	-2.797566	3.134990	-2.362213
H	-0.703249	2.532954	-1.464562
H	-0.077552	0.888547	-1.585649
H	-1.359553	1.259836	-0.410311
H	3.067479	1.114246	-4.550074
H	1.544902	1.319173	-3.649228
H	1.969069	2.459081	-4.926627

H	1.278526	0.292943	-7.704949
H	2.899293	0.589960	-7.035126
H	1.736062	1.925125	-7.193482
H	-2.962783	-0.167503	-8.038884
H	-3.122852	-1.939415	-7.994301
H	-1.603743	-1.160312	-7.501237
H	-5.172673	-1.570473	-6.559811
H	-4.854984	0.176168	-6.683990
H	-4.986902	-0.584722	-5.090298
H	-2.145244	4.069414	-14.977294
H	-0.806955	3.737857	-13.874833
H	1.640894	5.295882	-13.818288
H	0.994000	2.738679	-11.863666
H	0.126647	5.243211	-12.208048
H	-2.897384	2.577927	-13.180520
H	1.524501	4.633889	-11.316676
H	-2.600758	0.639346	-12.172096
H	-4.555356	4.053690	-14.160962
H	-1.216548	0.647579	-11.081008
H	1.331867	2.761696	-9.711425
H	-2.667135	-0.277882	-10.674438
H	0.220715	5.458266	-10.458302
H	-4.125384	5.376335	-13.081735
H	-0.140061	1.789211	-9.571064
H	-4.918350	3.940556	-12.433378
H	-4.807511	1.821728	-11.485212
H	-2.396916	6.157004	-11.757758
H	-0.088891	3.407841	-8.871396
H	-2.623472	1.842456	-9.333665
H	-4.821046	0.883145	-9.992737
H	0.689407	4.060547	-6.128664
H	-1.831594	6.805076	-10.237536
H	-4.910469	2.647340	-9.923305
H	-4.734210	6.437896	-11.074664
H	-0.094636	5.172120	-7.253635
H	-3.873497	7.991140	-11.082948
H	-6.346839	5.494915	-9.987218
H	0.476618	4.380268	-3.643087
H	-7.200813	2.533447	-9.416881
H	-8.918043	4.429859	-9.702375
H	-1.476787	4.770909	-5.198597
H	0.979841	7.559487	-7.569034
H	-2.617454	8.378401	-9.020496
H	0.828334	6.104780	-3.643967
H	-8.496801	1.420271	-8.964725
H	-6.180166	7.984277	-9.843041
H	-10.197507	3.306665	-9.253169
H	-0.520243	8.225733	-8.204104
H	-7.557029	6.191590	-8.936035
H	-7.093831	1.636268	-7.906191
H	-0.685226	5.517506	-2.953230
H	0.832899	9.300186	-7.814370
H	-10.022517	4.831233	-8.381781
H	-4.132488	9.281247	-8.841822
H	-5.992190	7.848066	-8.085293
H	1.573215	7.899877	-5.044064
H	-3.034994	5.862356	-3.240928
H	-9.137677	2.877309	-7.069712
H	-2.935121	9.093896	-6.732525
H	-5.650074	2.877700	-6.008420
H	-4.461557	8.253519	-6.600571
H	-0.837510	9.497985	-6.052557
H	-8.833368	6.372103	-7.107607
H	-4.212487	7.002271	-3.882207
H	1.524988	9.635179	-5.345971
H	0.553142	8.942404	-4.045224
H	-1.346599	7.733212	-3.085014
H	-6.756823	7.356134	-6.021516
H	-7.771611	1.629293	-5.642058
H	-5.176538	5.018807	-4.785623
H	-3.617954	7.192564	-2.227797
H	-10.170472	4.654363	-5.862508
H	-3.522076	9.456277	-4.387981
H	-6.890343	1.767446	-4.119656
H	-8.286244	7.918970	-5.327151
H	-5.256054	3.626716	-3.704861
H	-1.862906	9.976674	-4.071490
H	-7.314151	6.718940	-4.478047
H	-8.287465	2.794605	-4.420697
H	-6.608966	4.750505	-3.783671
H	-10.259560	6.241361	-5.094397
H	-9.232780	4.988364	-4.402991
H	-2.941967	9.590487	-2.729828

Supplementary Table 31. Final coordinates and energy for K-TS-in

Energy: -2664.179654

U	-9.597930	-0.890669	2.842051
K	-8.122839	-2.202233	-1.528541
Si	-13.249269	-1.699659	3.513626
Si	-6.752787	-3.417130	3.370676
Si	-8.652697	2.577977	1.709338
N	-8.061852	-2.329409	3.909269
N	-8.705255	1.272846	2.937743
N	-9.317019	-0.127064	5.403277
N	-11.679705	-0.923399	3.901610
N	-9.902471	-1.701346	0.785106
C	-15.284197	-1.726819	5.701326
C	-14.671580	0.864536	3.419638
C	-8.404365	-5.165344	1.724048

C	-5.210057	-3.581198	0.872144
C	-4.848299	-2.547149	5.486648
C	-5.025398	-1.423206	2.148592
C	-5.938414	-2.601891	1.799294
C	-4.315048	-4.699291	4.304685
C	-5.440011	-3.771989	4.782721
C	-11.492410	2.806644	1.174414
C	-9.654037	4.633948	3.594889
C	-14.281960	-3.729564	1.725771
C	-14.093184	-0.170624	1.216636
C	-14.495792	-0.458989	2.665899
C	-12.116514	-4.286342	2.857865
C	-12.965463	-3.157500	2.265208
C	-13.186808	-3.036267	6.144506
C	-14.141705	-2.522192	5.062485
C	-6.944497	1.134352	-0.111606
C	-8.562631	5.479370	1.491359
C	-8.588590	4.352052	2.531638
C	-5.760840	2.769549	1.392323
C	-7.042838	2.481324	0.606169
C	-8.324482	-1.038507	6.002023
C	-8.391861	-2.412737	5.343441
C	-7.903415	-6.007194	4.034117
C	-7.368696	-5.197908	2.848223
C	-10.020905	3.001093	-0.848191
C	-10.179296	2.348062	0.530774
C	-7.973372	1.547557	4.182942
C	-8.853968	1.271963	5.397235
C	-10.636941	-0.272011	6.045136
C	-11.759908	-0.005333	5.051088
C	-11.460622	-1.337437	-1.329220
C	-10.958464	-3.038249	-3.110444
C	-10.963470	-1.693377	-2.642346
C	-10.343996	-0.745480	-3.505153
C	-9.749411	-1.114007	-4.707860
C	-9.735589	-2.450048	-5.126999
C	-10.361857	-3.402445	-4.313351
H	-10.351962	0.303488	-3.213677
H	-9.303707	-0.345646	-5.338016
H	-9.288946	-2.733424	-6.076102
H	-10.398416	-4.443434	-4.631128
H	-11.450365	-3.799738	-2.506581
H	-10.505966	-1.441312	-0.209778
H	-9.975451	-2.696452	0.940152
H	-15.815852	-2.339729	6.443344
H	-16.025650	-1.396031	4.967000
H	-14.920789	-0.837719	6.230558
H	-15.467378	-0.976791	2.656707
H	-14.983572	0.731474	4.459759
H	-14.602897	-3.407848	4.597620
H	-15.429505	1.492914	2.930960
H	-14.876348	-4.196782	2.521244
H	-13.711996	-3.698904	6.846912
H	-14.913610	-2.972437	1.251126
H	-14.783528	0.546052	0.749605
H	-14.091590	-4.509206	0.975389
H	-13.738976	1.439464	3.426790
H	-12.773590	-2.212744	6.737010
H	-14.082612	-1.065874	0.588776
H	-12.343358	-3.597630	5.731764
H	-12.644701	-4.805253	3.667319
H	-12.707048	-0.096190	5.606385
H	-13.090994	0.267856	1.169617
H	-12.409147	-2.721387	1.423777
H	-11.881282	-5.045422	2.098881
H	-12.354047	2.523390	0.556704
H	-11.716174	1.051361	4.738105
H	-11.168124	-3.925383	3.271866
H	-10.730082	-1.304967	6.391176
H	-11.641133	2.374529	2.169285
H	-10.707965	0.384753	6.929351
H	-11.521720	3.897218	1.278890
H	-9.399986	-2.830584	5.507440
H	-10.895826	2.789143	-1.477371
H	-9.331958	-4.691435	2.059610
H	-8.767227	-5.516597	4.498672
H	-10.250162	1.260455	0.369607
H	-10.655673	4.712438	3.160756
H	-9.736942	1.914152	5.328875
H	-9.941225	4.091712	-0.775985
H	-8.662622	-6.178812	1.385731
H	-8.469321	-1.099617	7.094370
H	-8.234855	-7.005670	3.716403
H	-9.695012	3.855151	4.361305
H	-7.721191	-3.079694	5.904356
H	-8.052958	-4.607314	0.848312
H	-9.137734	2.646763	-1.389100
H	-9.451580	5.585766	4.106056
H	-7.149490	-6.152706	4.815086
H	-8.336488	1.512503	6.342314
H	-7.330409	-0.620652	5.820967
H	-9.516786	5.560156	0.958286
H	-7.825639	0.941633	-0.734114
H	-6.474896	-5.713799	2.466241
H	-7.628572	2.589635	4.266963
H	-6.810737	-2.192392	1.264970
H	-7.057173	0.937340	4.262144
H	-7.613930	4.373000	3.041792
H	-6.008083	-4.332281	5.540596
H	-6.868892	0.317232	0.615266

H	-8.386773	6.450540	1.974568
H	-7.776335	5.346656	0.740193
H	-7.153831	3.263261	-0.158980
H	-5.616557	-1.856053	5.842796
H	-5.850730	-4.397340	0.520613
H	-5.516617	-0.713613	2.821491
H	-6.056168	1.089945	-0.759818
H	-4.690321	-5.621183	3.848384
H	-5.612676	2.042887	2.199759
H	-4.803303	-3.065972	-0.012027
H	-4.252764	-2.851373	6.358653
H	-4.720893	-0.870253	1.249233
H	-5.766027	3.766804	1.845368
H	-4.182472	-1.978426	4.829844
H	-4.353944	-4.047191	1.371735
H	-4.104360	-1.763360	2.634570
H	-3.670396	-4.993966	5.144429
H	-3.668660	-4.204929	3.570170
H	-4.873852	2.715253	0.744921
H	-11.768303	-0.290038	-1.249517
H	-12.264348	-1.994591	-0.981907

Supplementary Table 32. Final coordinates and energy for K-Product-in

Energy: -2664.202924 a.U

U	-11.214051	-4.320457	-0.146739
K	-8.865729	-1.394763	-1.342858
Si	-11.861492	-3.213393	-3.827250
Si	-11.477370	-1.907360	2.822642
Si	-10.469488	-7.958501	0.461656
N	-12.274771	-3.482780	-2.115732
N	-9.255046	-3.785371	-0.321310
N	-12.078978	-3.218786	1.780014
N	-13.845000	-4.834280	0.035776
N	-11.582814	-6.636004	0.024157
C	-13.199851	-5.670028	-4.787835
C	-9.051434	-3.133253	3.847458
C	-12.526591	0.118662	4.684907
C	-9.518355	0.260364	2.562414
C	-11.706616	-10.672589	0.637727
C	-7.948975	-8.266499	1.865630
C	-9.828526	-5.239264	-4.318755
C	-11.201805	-0.376525	-3.527550
C	-9.277060	-2.970905	-5.220468
C	-10.001754	-3.731537	-4.102857
C	-13.475700	-0.817701	-4.469676
C	-12.034240	-1.333065	-4.383505
C	-12.805520	-3.882156	-6.514584
C	-13.080418	-4.162135	-5.031781
C	-14.437715	-4.381915	-1.234992

C	-13.688164	-3.181494	-1.811258
C	-13.962745	-0.328066	2.664938
C	-12.959387	-0.930016	3.653490
C	-11.049447	-0.069190	0.595566
C	-10.319927	-0.761948	1.750471
C	-9.664434	-6.851677	3.014789
C	-9.096772	-7.271808	1.654745
C	-11.133707	-3.491669	5.221160
C	-10.375020	-2.520823	4.310853
C	-13.271690	-3.926786	2.271636
C	-14.338233	-4.054986	1.186369
C	-14.007252	-6.285539	0.240057
C	-12.899095	-7.060432	-0.465835
C	-12.459683	-9.013618	2.367487
C	-11.304885	-9.437174	1.450554
C	-8.492890	-7.724410	-1.635423
C	-9.568468	-8.683627	-1.112452
C	-10.549767	-9.018801	-2.241038
H	-13.507070	0.182551	-4.924604
H	-12.002463	0.961342	4.219558
H	-13.400463	0.538160	5.203069
H	-11.795254	0.645053	0.958224
H	-10.164099	1.038976	2.982874
H	-13.565113	0.575243	2.188597
H	-14.119856	-1.464878	-5.073739
H	-10.363457	0.533451	-0.026228
H	-14.890852	-0.032398	3.173539
H	-8.776068	0.775154	1.933252
H	-13.934059	-0.729773	-3.478243
H	-11.865552	-0.294117	5.454319
H	-11.324643	0.667792	-3.847340
H	-11.581674	-0.778958	-0.048828
H	-8.973131	-0.194492	3.394776
H	-14.230652	-1.025392	1.864992
H	-11.618172	-1.310452	-5.401339
H	-12.792880	-2.812621	-6.750704
H	-13.574250	-4.343468	-7.150269
H	-11.503277	-0.428871	-2.473842
H	-13.495880	-1.709121	4.215928
H	-14.063640	-3.725894	-4.797286
H	-13.773474	-2.341637	-1.099505
H	-9.614341	-1.501837	1.335986
H	-14.263719	-2.860000	-2.693206
H	-10.145138	-1.620671	4.899795
H	-10.129799	-0.597842	-3.613267
H	-11.842588	-4.300438	-6.832194
H	-14.584860	-3.049441	0.832435
H	-15.509385	-4.151701	-1.098273
H	-12.061628	-3.058705	5.612531
H	-14.083771	-6.080543	-5.295435

H -9.292143 -1.882884 -5.093516
H -8.428707 -2.416501 3.300731
H -13.757766 -3.429481 3.125485
H -13.285926 -5.913656 -3.724506
H -10.523507 -3.786205 6.086515
H -15.265378 -4.503872 1.584187
H -8.459822 -3.494627 4.700685
H -9.721377 -3.180616 -6.200379
H -14.369433 -5.203489 -1.952895
H -12.331663 -6.213857 -5.172656
H -9.513836 -3.509133 -3.140164
H -11.475000 -4.412062 4.688914
H -9.217940 -3.985562 3.180710
H -8.223167 -3.278157 -5.279848
H -13.025732 -4.938480 2.640028
H -10.208841 -5.546720 -5.299049
H -15.010538 -6.610683 -0.087777
H -10.349015 -5.831308 -3.560711
H -13.926192 -6.484807 1.312445
H -10.487753 -6.135804 2.919254
H -13.001568 -6.913616 -1.555439
H -8.767633 -5.520643 -4.283435
H -8.891224 -6.386564 3.640432
H -8.689771 -6.373354 1.166328
H -8.915160 -6.737031 -1.855749
H -13.099895 -8.132290 -0.307627
H -10.042720 -7.717270 3.571902
H -12.243660 -8.100616 2.929737
H -7.676691 -7.572124 -0.922003
H -13.378592 -8.835486 1.798066
H -11.063393 -8.116064 -2.590721
H -7.161671 -7.829526 2.495372
H -8.044583 -8.102062 -2.565599
H -7.476771 -8.585936 0.931529
H -12.689361 -9.804095 3.096032
H -8.294376 -9.172756 2.379224
H -10.025687 -9.447631 -3.107045
H -10.477860 -9.753882 2.105972
H -11.318991 -9.736023 -1.938709
H -9.075239 -9.614808 -0.794236
H -12.546012 -10.466109 -0.037099
H -10.881755 -11.061316 0.031745
H -12.028973 -11.486525 1.303114
H -8.285457 -4.097503 -0.217389

Supplementary Table 33. Final coordinates and energy for 3K

Energy: -3357.855907 a.u
U -10.001190 -4.063743 -0.273548
K -5.345346 -2.795155 -0.655853
Si -9.142664 -7.695028 0.542642
Si -10.682772 -2.888274 -3.866865
Si -10.317613 -1.581340 2.629361
N -3.634226 -2.651996 1.830232
N -4.319717 -0.207849 -1.801420
N -2.251302 -2.397665 -0.969434
N -3.831905 -4.698203 -2.374661
N -8.001112 -3.839189 -0.690523
N -10.270028 -6.397768 0.082936
N -11.190988 -3.390076 -2.233827
N -12.617318 -4.648458 0.013373
N -10.908255 -2.933126 1.637131
C -4.049000 -0.054290 -3.225685
C -4.180031 -1.832898 2.907613
C -3.733992 -4.061217 2.196983
C -2.249723 -2.260885 1.561063
C -1.643801 -2.843139 0.287899
C -4.164251 -4.290927 -3.735100
C -4.328873 -6.049434 -2.134904
C -5.350424 0.743630 -1.399341
C -3.126457 -0.004623 -0.979582
C -1.972064 -0.975501 -1.230575
C -1.791980 -3.243116 -2.080347
C -2.391444 -4.646339 -2.129357
C -10.169997 -0.158848 -3.074440
C -6.419925 -7.994740 1.495101
C -10.382143 -10.213798 1.583533
C -9.823994 -9.362815 -1.780754
C -8.582126 -4.819341 -4.392771
C -12.018907 -5.221142 -5.083057
C -11.176644 1.079477 3.517442
C -8.989486 -3.238993 4.600543
C -10.770912 -8.141077 2.949174
C -9.809768 -8.852137 1.991061
C -7.651073 -8.134432 -1.893478
C -8.637782 -8.842449 -0.962057
C -7.727240 -6.101116 2.496711
C -7.493951 -6.933090 1.233925
C -11.617882 -6.899939 -0.219454
C -12.701921 -6.054687 0.439097
C -8.054710 -2.462750 -5.103127
C -8.804398 -3.334122 -4.090355
C -11.327682 -3.382622 -6.667218
C -11.802719 -3.714739 -5.248022
C -11.182556 -2.287355 5.369661
C -9.947224 -2.050523 4.493792
C -12.293830 -0.470909 -4.376478
C -10.856382 -0.962501 -4.180229
C -12.639024 -3.242391 -2.029449
C -13.242567 -4.439166 -1.301812

C -13.171793 -3.739942 1.028427
C -12.173196 -3.477803 2.148397
C -12.262879 0.255726 1.395072
C -11.654458 -0.151355 2.739656
C -8.832089 -0.198682 0.557815
C -8.638188 -0.969439 1.866590
C -7.767499 -0.162726 2.835992
H -2.029740 -2.727986 -3.015761
H -0.690841 -3.357948 -2.068405
H -1.838758 -5.210225 -2.906661
H -2.197461 -5.170427 -1.186955
H -0.563302 -2.599271 0.303696
H -1.702688 -3.933840 0.335081
H -1.590353 -2.554649 2.401232
H -2.211601 -1.167267 1.517081
H -1.125409 -0.629775 -0.615453
H -1.634413 -0.860263 -2.265884
H -2.721763 1.018519 -1.122090
H -3.441358 -0.069903 0.067719
H -5.049640 1.794757 -1.566786
H -5.574142 0.630474 -0.333685
H -6.269694 0.563007 -1.963845
H -4.977670 -0.179751 -3.790234
H -3.345351 -0.813892 -3.575612
H -3.630884 0.938889 -3.475766
H -3.676004 -4.924397 -4.499316
H -3.871129 -3.253857 -3.917451
H -5.245671 -4.358909 -3.884878
H -4.137869 -6.344723 -1.098332
H -3.860965 -6.802048 -2.796284
H -5.408129 -6.082283 -2.303156
H -4.777023 -4.314158 2.409789
H -3.135541 -4.309393 3.093504
H -3.398397 -4.707262 1.381014
H -3.624904 -1.949851 3.856802
H -5.221372 -2.109235 3.093123
H -4.151618 -0.774578 2.630331
H -7.624290 -4.516153 -1.372449
H -10.590524 -10.830079 2.470314
H -9.695839 -10.785175 0.949634
H -11.329131 -10.114063 1.039635
H -8.125346 -9.709027 -0.515510
H -10.566751 -9.887898 -1.173178
H -8.888051 -9.059593 2.557334
H -9.487726 -10.062159 -2.559815
H -6.720556 -8.689373 2.289841
H -10.916621 -8.728171 3.867451
H -6.188532 -8.598697 0.611189
H -7.367763 -8.776518 -2.740216
H -5.479790 -7.530510 1.827439
H -10.337857 -8.539896 -2.289716
H -11.759695 -8.010824 2.495980
H -6.729582 -7.845891 -1.378953
H -10.417567 -7.148191 3.243272
H -8.092477 -6.717981 3.326926
H -11.782096 -7.934607 0.125876
H -8.095405 -7.223649 -2.312242
H -7.133138 -6.246061 0.455096
H -6.794891 -5.629459 2.839779
H -7.513808 -5.078128 -4.353241
H -11.809924 -6.936112 -1.305591
H -8.454150 -5.299502 2.334443
H -12.551473 -6.087301 1.521596
H -9.103888 -5.466879 -3.680597
H -13.708598 -6.459291 0.228054
H -8.928759 -5.087137 -5.397654
H -12.013544 -4.412302 2.714478
H -6.992597 -2.746768 -5.149547
H -9.464058 -4.158728 4.241846
H -11.745027 -3.171916 5.049488
H -8.383285 -3.141244 -3.091510
H -11.100175 -5.789126 -5.261684
H -13.057576 -5.332197 -1.906351
H -8.452787 -2.568036 -6.119441
H -8.680789 -3.415188 5.641058
H -14.123617 -4.138850 1.424477
H -10.892843 -2.458442 6.416523
H -12.361169 -5.475216 -4.075427
H -12.689645 -2.805555 2.855166
H -8.079411 -3.095530 4.007603
H -8.092002 -1.398391 -4.848052
H -12.770879 -5.593384 -5.793702
H -11.874792 -1.438008 5.364669
H -14.337303 -4.331151 -1.194165
H -13.392432 -2.785908 0.540974
H -10.358769 -3.845811 -6.888383
H -9.134533 -0.477424 -2.908731
H -9.431718 -1.166007 4.897780
H -13.204807 -3.151601 -2.971936
H -8.100698 -1.898571 1.620756
H -12.882447 -2.323957 -1.466254
H -12.787668 -3.239763 -5.125407
H -12.466515 -0.605067 3.328121
H -10.698540 -0.281149 -2.122625
H -12.037170 -3.756593 -7.419384
H -11.221473 -2.304637 -6.834082
H -10.315349 -0.779537 -5.120904
H -12.568692 -0.615393 0.807429
H -7.508203 -0.725197 3.738698
H -9.469201 -0.734545 -0.151505

H -10.155394 0.916526 -3.304301
H -10.758852 0.824269 4.497806
H -12.901204 -0.625329 -3.477211
H -6.824430 0.140795 2.358860
H -13.147747 0.892511 1.536888
H -7.872227 -0.020452 0.055172
H -12.801746 -0.973870 -5.206466
H -11.554892 0.824339 0.782452
H -8.263245 0.759286 3.159757
H -9.285824 0.783860 0.732849
H -12.002896 1.783240 3.692855
H -10.406202 1.631161 2.965395
H -12.314634 0.607145 -4.592911

Supplementary Table 34. Final coordinates and energy for K-TS-Dis

Energy: -5328.391487 a.u

U -11.253387 -4.498721 -0.286183
U -4.926128 -2.201967 -0.085773
K -8.129743 -1.981598 -2.148774
K -7.449352 -4.727305 1.788289
Si -10.851926 -8.271230 0.522231
Si -11.836694 -2.054992 2.541544
Si -5.512173 1.165478 -2.190062
Si -4.024394 -5.533216 -1.831459
Si -12.157714 -3.509432 -3.913614
Si -4.170121 -1.585482 3.708488
N -11.619335 -6.799471 -0.137693
N -13.940249 -4.998149 -0.082967
N -12.127959 -3.581839 1.648913
N -9.106724 -4.131350 -0.472620
N -6.804558 -2.871646 -0.020130
N -3.872428 -1.435105 1.970219
N -2.277188 -1.549565 -0.516532
N -4.780205 -0.387756 -1.734123
N -12.380174 -3.606270 -2.139857
N -3.685559 -4.106413 -0.837366
C -12.706541 -8.954070 2.744150
C -11.997071 -9.526313 1.514066
C -10.012682 -9.382376 -0.858509
C -8.600452 -8.918767 -1.229607
C -12.883765 -7.069769 -0.853744
C -14.072811 -6.465912 -0.116270
C -14.394462 -4.416801 1.192646
C -13.239568 -4.388420 2.185401
C -10.995922 -2.381214 4.270723
C -11.829441 -3.264931 5.204097
C -9.431654 -7.729830 1.737850
C -9.853671 -6.668885 2.755889
C -12.982862 -10.357430 0.684122
C -10.603349 -0.952475 1.531940
C -11.030217 -0.634961 0.095701
C -13.522299 -1.135792 2.938757
C -14.160511 -0.436680 1.733077
C -2.847000 -0.477244 1.655351
C -1.724604 -1.097112 0.769149
C -2.285911 -0.492761 -1.540176
C -3.508177 -0.621765 -2.448740
C -4.295965 2.702427 -2.038008
C -4.885172 3.999719 -2.608448
C -13.717277 -3.162940 -1.699005
C -14.565364 -4.347946 -1.249908
C -2.292741 -4.760000 -0.380613
C -1.613164 -2.776764 -0.985771
C -6.057894 1.240236 -4.083683
C -4.914653 1.360375 -5.099768
C -7.117142 1.413189 -1.108866
C -8.209745 2.261787 -1.769384
C -4.235687 -7.138447 -0.732871
C -2.959444 -7.494694 0.038388
C -5.702176 -5.279021 -2.781033
C -5.755798 -4.006853 -3.632226
C -2.554563 -5.962078 -3.061727
C -2.411961 -4.989174 -4.238208
C -13.695022 -4.251113 -4.878872
C -13.837239 -3.732157 -6.316302
C -12.806 -1.668840 -4.557496
C -13.207415 -0.785347 -4.223058
C -10.489127 -4.395207 -4.358692
C -10.137209 -4.298063 -5.847738
C -2.573165 -1.864421 4.828965
C -2.021014 -0.633511 5.557521
C -5.286705 -3.150892 4.036026
C -4.649622 -4.449537 3.534337
C -5.148274 -0.066182 4.460429
C -6.630492 -0.090587 4.074546
C -10.713953 -0.986095 -4.096110
C -10.399201 -5.847478 -3.883241
C -8.790922 -8.926262 2.450956
C -10.846382 -9.517990 -2.138027
C -10.278140 0.345461 2.280343
C -13.441508 -0.162833 4.122939
C -9.586851 -2.957962 4.128570
C -6.974000 0.080129 -4.484180
C -6.806753 1.962143 0.287075
C -5.691150 -3.298834 5.506556
C -6.093881 -6.496065 -3.624140
C -2.557901 -7.406891 -3.577878
C -5.400184 -7.002733 0.249625
C -13.771757 -5.781707 -4.873998

C -3.700743 2.975997 -0.652357
C -4.546055 1.289027 4.072774
C -1.430478 -2.607609 4.129644
H -8.727747 -4.864173 -1.094270
H -13.446877 -11.132579 1.309885
H -12.500372 -10.871299 -0.152495
H -13.801481 -9.751873 0.278520
H -9.928176 -10.381433 -0.402991
H -11.848263 -9.920775 -1.961473
H -11.238184 -10.235144 1.882895
H -10.351073 -10.189313 -2.853220
H -9.484099 -9.381002 3.168040
H -13.458666 -8.204759 2.474240
H -8.476874 -9.712735 1.756564
H -8.169262 -9.567365 -2.004764
H -7.901466 -8.620538 3.019633
H -10.964949 -8.549422 -2.635945
H -12.015459 -8.484283 3.449379
H -7.911754 -8.935201 -0.379764
H -13.231617 -9.748827 3.292438
H -10.645003 -7.026945 3.420509
H -13.073968 -8.140173 -1.007117
H -8.606780 -7.900313 -1.636592
H -8.668345 -7.292364 1.075067
H -9.019923 -6.395626 3.423273
H -9.389537 -6.249365 -4.034685
H -12.859973 -6.643451 -1.870972
H -10.242419 -5.756200 2.286831
H -14.064818 -6.837725 0.911767
H -10.637611 -5.964139 -2.819759
H -15.034360 -6.760192 -0.569529
H -11.085825 -6.493769 -4.437679
H -12.915384 -5.422451 2.383589
H -9.137185 -4.708114 -6.043402
H -9.640644 -3.952351 3.668581
H -11.896088 -4.290216 4.822219
H -9.727096 -3.828793 -3.799669
H -13.021717 -6.222119 -5.540864
H -14.604183 -5.070822 -2.069976
H -10.840051 -4.873229 -6.461124
H -9.106748 -3.079376 5.108745
H -15.267739 -4.965937 1.584045
H -11.374386 -3.323890 6.202731
H -13.618272 -6.212396 -3.879369
H -13.628637 -4.022003 3.147340
H -8.937694 -2.321763 3.517023
H -10.145871 -3.267618 -6.218956
H -14.752050 -6.125476 -5.231754
H -12.852621 -2.896274 5.337078
H -15.603048 -4.052614 -1.019746
H -14.709711 -3.385455 1.008066
H -12.984326 -4.016880 -6.943144
H -9.826404 -1.523705 -4.450884
H -10.908154 -1.388888 4.735273
H -14.276417 -2.616656 -2.472907
H -9.674626 -1.539718 1.470380
H -13.634543 -2.454667 -0.858678
H -14.567597 -3.879945 -4.320100
H -14.204026 -1.945541 3.238977
H -10.688868 -0.912864 -3.944000
H -14.734660 -4.153336 -6.790402
H -13.931369 -2.643587 -6.365489
H -11.946841 -1.760421 -5.652179
H -14.202829 -1.077389 0.845955
H -9.925600 0.167385 3.301207
H -11.268103 -1.526033 -0.498602
H -10.641959 0.038045 -4.486446
H -13.089522 -0.641370 5.041885
H -13.266003 -0.582801 -3.147710
H -9.491910 0.907249 1.761851
H -15.189448 -0.125137 1.960454
H -10.230637 -0.090196 -0.427090
H -14.158674 -1.236056 -4.526631
H -13.610765 0.470344 1.456160
H -11.153560 1.002463 2.342327
H -11.916579 0.006509 0.061722
H -14.431137 0.262643 4.340085
H -12.772196 0.680215 3.917389
H -13.134010 0.187470 -4.728782
H -7.982987 -3.458476 -0.209376
H -1.214991 -0.921938 6.247786
H -2.782178 -0.118276 6.152225
H -1.592587 0.096997 4.860976
H -5.078947 -0.169163 5.554361
H -3.496246 1.390717 4.363126
H -2.959031 -2.536745 5.611141
H -5.096363 2.112883 4.549341
H -4.820530 -3.498324 6.142906
H -0.688281 -2.960669 4.859749
H -6.183575 -2.405645 5.903408
H -7.153197 0.793850 4.465888
H -6.389000 -4.144034 5.649597
H -4.603311 1.449409 2.990383
H -0.899624 -1.951379 3.432050
H -7.151244 -0.971005 4.463338
H -1.772650 -3.477383 3.561091
H -3.728377 -4.674902 4.083471
H -2.283433 -0.086344 2.548984
H -6.759005 -0.085390 2.985518
H -6.200398 -2.946185 3.455688

H -5.305027 -5.322284 3.696748
H -7.700778 1.944456 0.923473
H -3.192262 0.420157 1.147300
H -4.381253 -4.406517 2.472318
H -1.324420 -1.971913 1.288200
H -6.028388 1.386894 0.797158
H -0.885861 -0.396959 0.605008
H -6.474131 3.004161 0.238584
H -2.238316 -3.915398 0.718141
H -9.093819 2.337448 -1.121311
H -5.183692 -6.182733 0.946119
H -2.715317 -6.718467 0.773477
H -7.514358 0.398060 -0.951451
H -4.418264 3.473340 0.007885
H -2.335206 0.475837 -1.036597
H -7.865174 3.286111 -1.951848
H -5.537231 -7.916688 0.844855
H -0.532730 -2.742476 -0.756563
H -3.078534 -8.436805 0.592729
H -3.379445 2.063344 -0.142697
H -1.675027 -4.880362 -0.622826
H -6.345738 -6.790802 -0.262841
H -8.543923 1.859865 -2.731402
H -2.826845 3.638204 -0.727106
H -2.086737 -7.612135 -0.613136
H -1.346813 -0.512446 -2.122281
H -1.716763 -2.834186 -2.073301
H -5.714231 4.368929 -1.992707
H -7.903741 0.087858 -3.909473
H -4.462298 -7.965340 -1.421005
H -3.349144 0.064843 -3.295131
H -6.444255 -5.156875 -1.977676
H -3.489602 -1.627542 -2.904245
H -3.456012 2.416705 -2.690903
H -1.649362 -5.850867 -2.446510
H -6.463017 -0.883944 -4.344765
H -4.128648 4.796874 -2.626992
H -5.260474 3.888761 -3.630654
H -6.652267 2.163038 -4.154794
H -2.464081 -3.940732 -3.926348
H -6.089874 -7.428911 -3.050895
H -5.509512 -3.108596 -3.053552
H -7.263043 0.138919 -5.542048
H -2.554769 -8.146153 -2.770768
H -4.353625 0.423024 -5.188894
H -7.101802 -6.379227 -4.045168
H -1.451742 -5.131386 -4.753911
H -6.756724 -3.876403 -4.075136
H -4.198736 2.148942 -4.846608
H -3.196177 -5.147251 -4.988303
H -5.414257 -6.630579 -4.473290
H -5.054819 -4.043364 -4.471924
H -1.668224 -7.599561 -4.194250
H -3.430016 -7.616352 -4.207056
H -5.306616 1.589687 -6.101117

Supplementary Table 35. Final coordinates and energy for K-Product-Dis

Energy: -5328.4065537 a.u

U	-4.124993	-1.688668	-0.522335
U	-11.929632	-4.628775	0.004266
Si	-3.709090	-1.414123	3.292882
Si	-12.435467	-3.464362	-3.522725
Si	-3.182411	-5.143336	-2.446053
Si	-4.705040	1.341945	-2.766468
Si	-12.332518	-2.030803	2.691474
Si	-11.229019	-8.283337	0.606517
N	-3.031183	-3.847941	-1.213777
N	-12.994982	-3.794742	-1.834765
N	-3.680338	0.037920	-2.143905
N	-1.445579	-1.293866	-0.728780
N	-3.201681	-1.137617	1.617338
N	-5.744903	-2.768783	-0.196482
N	-9.569321	-4.396472	0.099102
N	-12.772760	-3.542148	1.803537
N	-14.550575	-5.113779	0.153188
N	-12.262967	-6.886039	0.089673
C	-1.057691	-2.513919	3.985553
C	-4.065037	1.440330	3.795477
C	-2.683465	3.437913	-2.265922
C	-13.458474	-6.019067	-4.556860
C	-4.455221	-6.878323	-0.490600
C	-1.696078	-6.642576	-4.455632
C	-5.478026	-6.174718	-3.945756
C	-5.551257	-3.144065	4.725956
C	-5.405741	2.418316	-0.148919
C	-6.641281	-0.354316	-4.0739627
C	-10.545035	-3.230213	4.643461
C	-13.785117	0.254790	3.815852
C	-10.308944	0.026869	2.559713
C	-11.561933	-9.573770	-1.998256
C	-8.775034	-8.753143	2.070618
C	-10.193123	-5.319221	-3.312906
C	-11.839227	-0.632851	-3.206211
C	-6.138368	0.130725	3.310307
C	-4.7608593	0.086916	3.977405
C	-4.113802	-4.273794	3.009107
C	-4.854088	-2.983026	3.371776
C	-1.791399	-0.615636	5.451376

C	-2.26706	-1.7847047	4.581718	H	-4.816288	-6.658416	-4.673789
C	-9.868768	-3.394308	-4.894623	H	-2.046293	-4.169743	-5.449472
C	-10.538062	-3.854205	-3.594175	H	-4.189669	1.390291	-5.834921
C	-14.084065	-1.117257	-4.228646	H	-5.770371	-3.560262	-4.868186
C	-12.639084	-1.605635	-4.074827	H	-0.346234	-4.231502	-4.997230
C	-13.275446	-4.159850	-6.244618	H	-6.421249	-5.965357	-4.468744
C	-13.521228	-4.504325	-4.770591	H	-4.133238	-0.305267	-5.341079
C	-1.365302	-4.166227	-4.592374	H	-1.816240	-7.534191	-3.832675
C	-1.647709	-5.339490	-3.645186	H	-7.203552	-0.649995	-4.971008
C	-4.771982	-3.830510	-4.500892	H	-4.286430	-2.908762	-4.165544
C	-4.865987	-4.883301	-3.390796	H	-5.696783	-6.912692	-3.166967
C	-2.043489	-7.398546	-0.900681	H	-1.464372	-3.188202	-4.113034
C	-3.337042	-6.883104	-1.540246	H	-6.345048	1.696576	-4.618691
C	-7.252098	2.494266	-1.851122	H	-5.227328	3.717214	-4.639199
C	-6.013988	1.727367	-1.373507	H	-3.908574	4.840557	-4.301032
C	-4.809467	0.531255	-5.553867	H	-6.069972	-1.228903	-3.743724
C	-5.717487	0.831504	-4.357271	H	-0.791316	-5.416629	-2.958026
C	-0.875123	-2.569799	-1.192581	H	-3.061462	2.537133	-4.157367
C	-1.666304	-3.764697	-0.653835	H	-2.311610	-1.087219	-3.340303
C	-15.172520	-4.674556	-1.109173	H	-5.554240	-4.475838	-2.631331
C	-14.424012	-3.473369	-1.661341	H	-2.083232	0.640607	-3.465002
C	-4.531700	4.049074	-3.860896	H	-3.629557	-7.592294	-2.327107
C	-3.664859	2.908371	-3.314775	H	-7.372986	-0.132299	-3.289086
C	-2.362897	-0.152530	-2.750772	H	-5.126245	4.519937	-3.069152
C	-1.254917	-0.194894	-1.695182	H	-0.922917	-2.589484	-2.283534
C	-0.952036	-0.937738	0.615193	H	-0.256445	-0.263509	-2.163191
C	-2.039030	-0.258357	1.446028	H	-1.210405	-7.444318	-1.609715
C	-14.272720	-0.255339	1.399777	H	-2.036099	4.218358	-2.691072
C	-13.887991	-0.850769	2.757948	H	-7.765241	2.676967	-2.682707
C	-10.946164	-0.940864	0.338857	H	-5.404999	-6.473432	-0.871766
C	-10.786522	-1.235909	1.832021	H	-1.057446	-4.664052	-0.841555
C	-12.783822	-10.783923	1.089215	H	-2.031951	2.652165	-1.873012
C	-10.100754	-6.752856	2.809457	H	-2.177294	-8.411691	-0.495459
C	-9.708751	-7.624621	1.613615	H	0.190551	-2.637684	-0.908616
C	-12.916316	-2.958993	5.427748	H	-4.684708	-7.889247	-0.128758
C	-11.811093	-2.377212	4.538526	H	-6.995608	3.506606	-2.183608
C	-13.966428	-4.212441	2.362993	H	-1.291392	0.744643	-1.135394
C	-15.054463	-4.343819	1.308172	H	-3.201768	3.884473	-1.410895
C	-14.699287	-6.565484	0.360049	H	-6.354316	0.726875	-1.052905
C	-13.598430	-7.323274	-0.368962	H	-1.726524	-6.763182	-0.065125
C	-9.271814	-8.671189	-1.533226	H	-4.149537	-6.319228	0.407589
C	-10.522505	-9.295261	-0.906441	H	-7.983581	2.603025	-1.037884
C	-12.153003	-9.564588	1.772320	H	-1.707553	-3.676161	0.443675
C	-13.141896	-8.947607	2.766549	H	-5.147187	3.460200	-0.370353
K	-5.999018	-5.452677	0.583192	H	-0.046014	-0.311152	0.535672
K	-8.676034	-1.747384	-0.466575	H	-4.494339	1.919663	0.196948
H	-5.395586	0.252298	-6.441315	H	-0.669201	-1.860222	1.128638
H	-2.519592	-6.633488	-5.179198	H	-3.578936	-4.180827	2.055988
H	-0.770105	-6.773787	-5.032602	H	-2.309052	0.691559	0.949328
H	-4.210742	-4.207606	-5.362758	H	-6.114821	2.438545	0.689520

H	-4.809344	-5.123100	2.931446
H	-5.612818	-2.816925	2.594019
H	-6.041137	0.222869	2.221397
H	-1.573306	0.046709	2.398000
H	-3.371176	-4.545477	3.769922
H	-1.341947	-3.341935	3.329194
H	-6.729490	-0.769192	3.506100
H	-0.431582	-1.832573	3.399123
H	-3.902305	1.655419	2.733413
H	-6.255531	-3.988474	4.713822
H	-6.722560	0.992960	3.661613
H	-6.118529	-2.254987	5.019160
H	-0.417950	-2.926620	4.779026
H	-4.834103	-3.347556	5.531753
H	-4.679756	2.258410	4.198168
H	-2.762482	-2.493983	5.263595
H	-3.092221	1.492061	4.292943
H	-4.904703	-0.087403	5.054946
H	-1.266409	0.147470	4.864181
H	-2.614612	-0.121213	5.977448
H	- 1.084220	-0.965175	6.217973
H	-9.228578	-4.705498	1.014789
H	-14.110358	-0.133721	-4.717065
H	-12.978455	0.960651	3.591454
H	-14.716319	0.836307	3.858508
H	-11.623449	-0.098031	0.170064
H	-11.032860	0.843511	2.459101
H	-13.567368	0.522541	1.086727
H	-14.699820	-1.792634	-4.833119
H	-9.982827	-0.669079	-0.113430
H	-15.263925	0.216082	1.444905
H	-9.363637	0.385824	2.134609
H	-14.575977	-1.530000	-3.256570
H	-13.605109	-0.138674	4.820900
H	-11.871739	0.384673	-3.618870
H	-11.349615	-1.780989	-0.238896
H	-10.142948	-0.137972	3.628615
H	-14.302447	-1.009345	0.606074
H	-12.189432	-1.601330	-5.079450
H	-13.359090	-3.087729	-6.449231
H	-14.003938	-4.670726	-6.888862
H	-12.249702	-0.582963	-2.192580
H	-14.712529	-1.511831	3.065695
H	-14.550816	-4.190827	-4.537100
H	-14.555904	-2.613669	-0.98624
H	-9.997477	-1.996176	1.939510
H	-14.921874	-3.183340	-2.597194
H	-11.571408	-1.376620	4.929167
H	-10.783679	-0.913292	-3.122435
H	-12.281235	-4.480055	-6.576080

H	-15.319338	-3.340302	0.962921
H	-16.244423	-4.462497	-0.960661
H	-13.857111	-2.401066	5.360316
H	-14.256641	-6.529418	-5.113096
H	-10.020060	-2.328664	-5.091856
H	-9.702871	-2.802367	4.089821
H	-14.400484	-3.673473	3.216282
H	-13.565726	-6.291120	-3.501785
H	-12.610677	-2.950784	6.482786
H	-15.967571	-4.808804	1.715316
H	-10.223204	-3.334653	5.688337
H	-10.245312	-3.946668	-5.762664
H	-15.093311	-5.491579	-1.832190
H	-12.509519	-6.438787	-4.908595
H	-10.108008	-3.250953	-2.779117
H	-13.130923	-4.002314	5.170165
H	-10.715156	-4.241154	4.255831
H	-8.785296	-3.563984	-4.851962
H	-13.714850	-5.209636	2.758090
H	-10.486750	-5.970755	-4.142100
H	-15.701684	-6.900708	0.045986
H	-10.694890	-5.711291	-2.417777
H	-14.602840	-6.765125	1.430401
H	-10.790609	-5.944655	2.536435
H	-13.713476	-7.183179	-1.454266
H	-9.112370	-5.447085	-3.169484
H	9.218494	-6.292693	3.274046
H	-9.144545	-6.998419	0.908992
H	-9.492490	-7.698060	-1.985212
H	-13.773312	-8.395107	-0.198984
H	-10.597471	-7.343752	3.587603
H	-12.748230	-8.056769	3.265205
H	-8.461555	-8.526585	-0.811731
H	-14.078656	-8.664690	2.274874
H	-11.878730	-8.645815	-2.487298
H	-7.856383	-8.342622	2.510320
H	-8.879573	-9.310342	-2.335905
H	-8.474412	-9.417514	1.253586
H	-13.404011	-9.671259	3.550406
H	-9.241053	-9.376318	2.842753
H	-11.141947	-10.221065	-2.780236
H	-11.307175	-9.950181	2.362293
H	-12.4605080	-10.068484	-1.61734
H	-10.226160	-10.261325	-0.468792
H	-13.657155	-10.517542	0.482051
H	-12.079012	-11.312988	0.440429
H	-13.131874	-11.504956	1.841729
H	-9.134321	-5.047880	-0.560971

Supplementary Table 36. Final coordinates and energy for 4K

Energy: -5327.214712

U -6.941702 -7.625179 -1.966565
U -1.351 -7.579919 2.982554
K -4.481153 -9.150469 1.136068
K -3.491404 -5.897253 -0.104031
Si -8.197874 -4.086882 -1.412078
Si -8.891961 -10.451257 -0.223696
Si -4.093094 -8.371396 -4.448061
Si -3.335618 -5.306477 5.019844
Si -0.938117 -11.393677 3.476251
Si 1.693385 -6.128003 0.702887
N -8.307706 -9.527413 -1.638244
N -5.706506 -7.770327 -3.980457
N -8.629537 -7.867884 -4.027009
N -8.329302 -5.737843 -2.074131
N -5.704090 -7.463942 -0.638971
N -1.756203 -5.970319 4.523095
N -2.250319 -7.619259 1.658476
N -0.791327 -9.693992 4.011258
N 0.732661 -7.419961 5.022965
N 1.129837 -6.950800 2.180901
C -8.364927 -4.575924 1.429271
C -10.234216 -2.137144 -2.382744
C -2.457914 -6.094451 -3.698173
C -6.151673 -2.336059 -0.338016
C -1.900715 -9.865695 -3.293033
C -2.926083 -9.633862 -6.847660
C -11.705979 -9.583952 -0.558622
C -6.560052 -12.188028 -0.036971
C -9.394837 -8.310826 1.678884
C -8.619708 -2.973106 -4.114040
C -8.817721 -2.668796 -2.625001
C -5.448728 -3.731982 -2.299967
C -6.330522 -3.682349 -1.049614
C -10.570382 -4.435645 0.246811
C -9.134827 -3.899754 0.289816
C -8.546426 -13.219373 -1.159869
C -8.088016 -12.232820 -0.080232
C -8.203631 -10.300288 2.627959
C -8.413498 -9.447260 1.371318
C -11.330864 -11.634490 0.849263
C -10.813650 -10.808330 -0.334649
C -4.163265 -10.394128 -2.333632
C -3.285402 -9.306109 -2.950148
C -4.814890 -10.894246 -5.765470
C -4.225387 -9.506265 -6.042177
C -9.478753 -9.046961 -3.765052
C -8.731253 -10.083511 -2.933785
C -3.358503 -6.034845 -6.039308
C -2.850174 -6.939057 -4.912203
C -6.505644 -7.262596 -5.107134
C -7.806400 -8.042789 -5.241388
C -9.423733 -6.624052 -4.096373
C -9.624025 -6.029605 -2.708116
C 1.957192 -11.991422 3.734344
C -3.825556 -11.604101 3.773499
C 0.248049 -11.182845 0.830939
C -3.835668 -3.461210 2.830193
C -6.146289 -5.886698 4.614433
C -4.519145 -4.225937 7.495726
C 0.558273 -4.546371 -1.449486
C 3.310679 -8.413480 -0.112316
C -4.628805 -7.892268 4.605435
C -4.745317 -6.405045 4.272023
C -3.518017 -6.529047 7.677005
C -3.430722 -5.171318 6.972113
C -2.580534 -2.498421 4.782761
C -3.655425 -3.500669 4.349905
C 0.178690 -6.433593 5.973531
C -0.606405 -5.369849 5.218402
C -2.501084 -12.498121 5.705807
C -2.524953 -12.294312 4.187343
C -1.779036 -12.651926 0.969627
C -1.092055 -11.403493 1.538851
C 1.147619 -8.095810 -1.334300
C 2.319180 -7.379232 -0.656282
C 0.411603 -13.972133 3.782583
C 0.559000 -12.482951 4.122121
C -0.469981 -9.535097 5.438792
C 0.831924 -8.764522 5.626150
C 2.027443 -6.963699 4.476559
C 2.212584 -7.447621 3.044054
C -0.368975 -4.078505 0.831242
C 0.203273 -5.159323 -0.089930
C 4.526928 -5.202649 0.662800
C 3.091838 -4.776493 0.991881
C 3.041924 -4.098333 2.364780
H -10.429264 -1.256701 -3.011419
H -10.396553 -1.833139 -1.343801
H -11.452 -2.880128 -2.635073
H -9.179885 -2.819450 0.496947
H -11.182849 -3.955846 -0.522545
H -8.129963 -1.846391 -2.372979
H -11.076145 -4.278027 1.209879
H -6.473158 -1.500729 -0.972197
H -8.739942 -2.062038 -4.716728
H -6.716009 -2.269354 0.597183
H -8.934640 -4.536218 2.368210

H -5.095359 -2.152661 -0.095977
H -10.583198 -5.513871 0.052734
H -9.363625 -3.690297 -4.477875
H -7.396872 -4.102463 1.618831
H -7.631321 -3.386352 -4.336500
H -5.741432 -2.961106 -3.022636
H -10.258333 -5.136750 -2.826993
H -8.171841 -5.631522 1.207451
H -6.018544 -4.483279 -0.363008
H -4.392892 -3.529210 -2.058667
H -9.467000 -7.668243 2.476393
H -10.228683 -6.727715 -2.104837
H -5.497823 -4.696630 -2.816625
H -8.866559 -5.900892 -4.697317
H -9.579764 -7.674805 0.808295
H -10.382428 -6.817070 -4.606514
H -10.360635 -8.695762 2.023934
H -6.745624 -6.193412 -4.982073
H -7.901593 -9.673723 3.478902
H -3.346738 -5.625893 -3.256742
H -4.227074 -5.448642 -5.717533
H -7.459759 -8.966244 1.153000
H -11.794015 -8.971179 0.344469
H -10.351577 -8.715798 -3.196928
H -9.127514 -10.808376 2.928478
H -1.770843 -5.285199 -3.980640
H -8.382924 -7.745837 -6.134101
H -2.585050 -5.319176 -6.351318
H -11.325240 -8.934291 -1.352356
H -5.990155 -7.317949 -6.077529
H -1.954956 -6.693087 -2.929849
H -7.440477 -11.075710 2.505003
H -12.724250 -9.887384 -0.839211
H -3.655398 -6.599555 -6.930403
H -9.843022 -9.469413 -4.716647
H -7.559469 -9.104260 -5.340318
H -11.275232 -11.070853 1.788226
H -6.194121 -11.563789 0.787315
H -1.944187 -7.446904 -5.273765
H -9.397383 -10.955476 -2.840324
H -3.153900 -8.529108 -2.182413
H -7.868838 -10.450404 -3.517887
H -10.908190 -11.439113 -1.231690
H -4.943932 -8.981125 -6.688593
H -6.150639 -11.790416 -0.970449
H -12.385601 -11.907924 0.705900
H -10.773737 -12.566496 0.991765
H -8.438171 -12.619929 0.888395
H -5.729259 -10.850330 -5.163524
H -1.227545 -9.107273 -3.705914
H -5.137998 -10.008322 -2.016373
H -6.131560 -13.189387 0.110836
H -2.537238 -8.664506 -7.176422
H -8.202110 -12.916060 -2.155232
H -1.413208 -10.282021 -2.401579
H -5.063868 -11.409352 -6.703967
H -3.673681 -10.839975 -1.454363
H -9.636095 -13.321459 -1.204247
H -4.102471 -11.536234 -5.234476
H -1.966429 -10.678232 -4.025754
H -4.352581 -11.215840 -3.031167
H -3.090361 -10.237790 -7.751052
H -2.131620 -10.127546 -6.276768
H -8.136677 -14.222131 -0.973136
H 5.208563 -4.341107 0.704156
H 4.616859 -5.634378 -0.339352
H 4.910140 -5.941340 1.376845
H 2.839363 -6.771890 -1.412877
H 4.186508 -7.958707 0.360574
H 2.816677 -4.011864 0.248743
H 3.676305 -9.066755 -0.916832
H 1.346133 -3.788455 -1.357221
H 3.652616 -3.184599 2.375284
H 0.906984 -5.287759 -2.174798
H 1.505591 -8.852479 -2.046362
H -0.309842 -4.038504 -1.892674
H 2.833018 -9.059626 0.632133
H 3.440214 -4.753056 3.147205
H 0.502490 -7.408856 -1.890399
H 2.026584 -3.816808 2.661000
H 0.368078 -3.289916 1.025236
H 3.214042 -7.122507 2.718884
H 0.519600 -8.610122 -0.598757
H -0.564752 -5.932472 -0.247654
H -1.234138 -3.573223 0.373280
H 0.102105 -11.032377 -0.246480
H 2.259165 -8.549793 3.044841
H -0.685250 -4.471438 1.803468
H 2.021296 -5.870819 4.466801
H 0.782910 -10.308041 1.213940
H 2.852035 -7.288598 5.133266
H 0.905832 -12.051407 0.944077
H 0.073976 -4.866007 4.510584
H -1.854609 -12.589930 -0.124652
H -2.925984 -3.808806 2.325040
H -1.611893 -2.728756 4.324070
H -1.715440 -10.523475 1.311903
H 2.177768 -12.185157 2.680149
H 1.628781 -9.308448 5.113707
H -1.210619 -13.562018 1.192514

H -4.031674 -2.438320 2.480091
H 0.988593 -6.001796 6.585308
H -2.845419 -1.476109 4.478276
H 2.083635 -10.917053 3.896019
H -0.883489 -4.586169 5.939123
H -4.677393 -4.082274 2.501900
H -2.791014 -12.803483 1.358459
H 2.730126 -12.506012 4.321933
H -2.433257 -2.483125 5.868749
H 1.115349 -8.683179 6.689037
H -0.510507 -6.951443 6.646804
H 0.506865 -14.153269 2.705299
H -3.920159 -11.553073 2.681938
H -4.606403 -3.188267 4.805472
H -0.362217 -10.491353 5.972927
H -4.602636 -6.304163 3.185181
H -1.274795 -9.002677 5.974400
H 0.486204 -12.398930 5.217350
H -2.466059 -4.724177 7.255644
H -3.875415 -10.587170 4.175802
H 1.198064 -14.563281 4.272293
H -0.549129 -14.388473 4.103063
H -2.514976 -13.290254 3.719787
H -2.789950 -7.248622 7.286926
H -6.271872 -4.822710 4.389287
H -3.642534 -8.297808 4.351695
H -4.706946 -12.146968 4.142874
H -4.417967 -3.209391 7.102331
H -2.559915 -11.544376 6.243073
H -6.917307 -6.429617 4.051935
H -3.335883 -6.427730 8.755806
H -5.389656 -8.476250 4.065209
H -1.596562 -13.014095 6.045227
H -4.513166 -6.975928 7.565271
H -6.373771 -6.025284 5.677628
H -4.794790 -8.088992 5.669678
H -4.475111 -4.151931 8.591498
H -5.526173 -4.575133 7.241902
H -3.358637 -13.101727 6.035148

Supplementary Table 37. Final coordinates and energy for 6K
Energy: -3357.257690 a.u
U -3.594475 4.970237 -7.809619
K -1.626085 1.520917 -5.444017
Si -1.514443 7.190107 -5.493657
Si -7.273476 4.479638 -6.901688
Si -2.219557 3.562344 -11.058483
N -5.964424 5.150893 -7.912246
N -2.801577 4.905866 -10.049178
N -4.329196 7.193619 -9.169209
N -2.777934 6.938473 -6.725229
N -2.983413 3.499994 -6.942182
N -3.175102 -0.938621 -6.101463
N 1.285033 0.602970 -5.622949
N -0.804889 -1.194123 -4.098538
N -1.976616 1.200866 -2.460594
C 0.905833 8.772000 -5.068350
C -2.650022 9.287948 -3.745855
C 0.022168 5.373522 -3.776132
C -4.444509 1.808926 -10.450338
C -5.845895 4.249232 -4.390077
C -7.647216 7.036232 -5.471551
C -9.460027 4.013451 -8.845820
C -4.172617 4.284203 -13.156501
C 0.232983 2.806031 -9.728971
C -3.321491 6.919846 -3.250937
C -2.201720 7.823002 -3.771933
C 0.344985 5.097670 -6.254650
C -0.642588 5.487006 -5.151657
C 0.287146 8.403739 -7.487079
C -0.244701 8.570750 -6.061296
C -9.576002 5.419325 -5.351218
C -8.402226 5.921224 -6.200459
C -7.787960 2.162637 -8.570404
C -8.513788 3.311078 -7.866105
C -7.408544 2.346009 -4.898596
C -6.473308 3.401309 -5.499581
C -1.788926 4.216766 -13.947270
C -2.794186 3.647631 -12.939745
C 0.432893 4.759771 -11.275676
C -0.268871 3.417867 -11.040120
C -5.789237 7.342944 -9.033472
C -6.476497 5.985201 -9.006863
C -2.311196 0.664892 -11.113991
C -2.916348 1.875394 -10.393183
C -2.650542 6.217048 -10.686729
C -3.944515 7.009854 -10.581261
C -3.617046 8.340689 -8.578524
C -3.465314 8.187949 -7.072567
C -2.693113 -1.063626 -7.472841
C -4.618505 -0.725074 -6.111410
C -2.837604 -2.113921 -5.353000
C -1.358385 -2.251866 -4.950514
C -3.118952 2.092417 -2.287057
C 2.003528 1.404757 -4.637884
C 1.830799 0.863257 -6.952041
C 1.383113 -0.827342 -5.327922
C 0.660816 -1.289053 -4.064746
C -1.376051 -1.241715 -2.742250

C -2.438564 -0.187209 -2.434330
C -0.985198 1.476794 -1.428501
H 1.241315 5.729299 -6.236459
H 1.019636 -0.691977 -3.221629
H 0.990439 -2.326738 -3.863323
H 2.443540 -1.131124 -5.221034
H 1.003750 -1.371571 -6.199701
H -1.229226 -3.242372 -4.471221
H -0.777890 -2.289555 -5.876342
H -3.141215 -3.045593 -5.816407
H -3.428785 -2.077074 -4.378760
H -1.833052 -2.226090 -2.550208
H -0.568468 -1.157601 -2.007668
H -2.872455 -0.437455 -1.444647
H -3.253141 -0.274 -3.161680
H -3.620236 1.960301 -1.309902
H -3.863290 1.908282 -3.067845
H -2.797566 3.134990 -2.362213
H -0.703249 2.532954 -1.464562
H -0.077552 0.888547 -1.585649
H -1.359553 1.259836 -0.410311
H 3.067479 1.114246 -4.550074
H 1.544902 1.319173 -3.649228
H 1.969069 2.459081 -4.926627
H 1.278526 0.292943 -7.704949
H 2.899293 0.589960 -7.035126
H 1.736062 1.925125 -7.193482
H -2.962783 -0.167503 -8.038884
H -3.122852 -1.939415 -7.994301
H -1.603743 -1.160312 -7.501237
H -5.172673 -1.570473 -6.559811
H -4.854984 0.176168 -6.683990
H -4.986902 -0.584722 -5.090298
H -2.145244 4.069414 -14.977294
H -0.806955 3.737857 -13.874833
H -1.640894 5.295882 -13.818288
H 0.994000 2.738679 -11.863666
H 0.126647 5.243211 -12.208048
H -2.897384 2.577927 -13.180520
H 1.524501 4.633889 -11.316676
H -2.600758 0.639346 -12.172096
H -4.555356 4.053690 -14.160962
H -1.216548 0.647579 -11.081008
H 1.331867 2.761696 -9.711425
H -2.667135 -0.277882 -10.674438
H 0.220715 5.458266 -10.458302
H -4.125384 5.376335 -13.081735
H -0.140061 1.789211 -9.571064
H -4.918350 3.940556 -12.433378
H -4.807511 1.821728 -11.485212
H -2.396916 6.157004 -11.757758
H -0.088891 3.407841 -8.871396
H -2.623472 1.842456 -9.333665
H -4.821046 0.883145 -9.992737
H 0.689407 4.060547 -6.128664
H -1.831594 6.805076 -10.237536
H -4.910469 2.647340 -9.923305
H -4.734210 6.437896 -11.074664
H -0.094636 5.172120 -7.253635
H -3.873497 7.991140 -11.082948
H -6.346839 5.494915 -9.987218
H 0.476618 4.380268 -3.643087
H -7.200813 2.533447 -9.416881
H -8.918043 4.429859 -9.702375
H -1.476787 4.770909 -5.198597
H 0.979841 7.559487 -7.569034
H -2.617454 8.378401 -9.020496
H 0.828334 6.104780 -3.643967
H -8.496801 1.420271 -8.964725
H -6.180166 7.984277 -9.843041
H -10.197507 3.306665 -9.253169
H -0.520243 8.225733 -8.204104
H -7.557029 6.191590 -8.936035
H -7.093831 1.636268 -7.906191
H -0.685226 5.517506 -2.953230
H 0.832899 9.300186 -7.814370
H -10.022517 4.831233 -8.381781
H -4.132488 9.281247 -8.841822
H -5.992190 7.848066 -8.085293
H 1.573215 7.899877 -5.044064
H -3.034994 5.862356 -3.240928
H -9.137677 2.877309 -7.069712
H -2.935121 9.093896 -6.732525
H -5.650074 2.877700 -6.008420
H -4.461557 8.253519 -6.600571
H -0.837510 9.497985 -6.052557
H -8.833368 6.372103 -7.107607
H -4.212487 7.002271 -3.882207
H 1.524988 9.635179 -5.345971
H 0.553142 8.942404 -4.045224
H -1.346599 7.733212 -3.085014
H -6.756823 7.356134 -6.021516
H -7.771611 1.629293 -5.642058
H -5.176538 5.018807 -4.785623
H -3.617954 7.192564 -2.227797
H -10.170472 4.654363 -5.862508
H -3.522076 9.456277 -4.387981
H -6.890343 1.767446 -4.119656
H -8.286244 7.918970 -5.327151
H -5.256054 3.626716 -3.704861

H -1.862906 9.976674 -4.071490
H -7.314151 6.718940 -4.478047
H -8.287465 2.794605 -4.420697
H -6.608966 4.750505 -3.783671
H -10.259560 6.241361 -5.094397
H -9.232780 4.988364 -4.402991
H -2.941967 9.590487 -2.729828

Supplementary Table 38. Final coordinates and energy for Cs-adduct-in

Energy: -2355.873474 a.u

C	-0.564351	-2.313782	-6.581903
C	0.075736	-1.332376	-7.361077
C	-0.175025	0.011228	-7.026718
C	-0.959579	0.362145	-5.940230
C	-1.564070	-0.618312	-5.066830
C	-1.352150	-1.984128	-5.491049
C	-2.223466	-0.283423	-3.880382
N	-0.355926	-0.681734	-0.984164
U	-0.182409	0.197528	1.069596
N	-2.217759	0.213942	2.114195
C	-2.301237	1.126628	3.269414
C	-1.185673	0.828607	4.262231
N	0.132149	0.953805	3.609433
C	0.613927	2.347864	3.603398
C	1.468341	2.613424	2.370097
N	0.700203	2.332187	1.145623
Si	0.719404	3.650025	-0.085817
C	-0.829549	3.408997	-1.230883
C	-0.707220	4.060219	-2.614750
N	1.321854	-1.236733	2.082401
Si	2.598624	-2.344382	1.470394
C	1.928023	-4.122728	1.028537
C	1.442646	-4.898194	2.257750
C	1.048469	-1.337953	3.530042
C	1.123371	0.031448	4.195736
C	2.314237	3.571740	-1.206657
C	3.608189	3.829517	-0.429397
C	0.776974	5.419486	0.745477
C	0.754191	6.546901	-0.295707
C	2.397995	2.252793	-1.976407
Si	-3.786811	-0.585941	1.708804
C	-4.739687	-1.295443	3.272688
C	-3.844763	-1.756601	4.429024
C	-3.448120	-2.136883	0.591297
C	-2.707806	-3.241940	1.351451
C	-4.964936	0.615151	0.730571
C	-4.480633	0.824558	-0.707645
C	-4.735282	-2.708769	-0.016608
C	-0.261479	5.685649	1.839002
C	-2.126784	3.869424	-0.556989
C	3.983781	-2.664581	2.815800
C	5.096087	-3.582737	2.291209
C	3.311173	-1.556838	-0.161737
C	4.244970	-0.376444	0.122697
C	4.591067	-1.425454	3.479664
C	3.996634	-2.558703	-1.098535
C	0.840369	-4.100394	-0.046943
C	-5.166619	1.972259	1.413408
C	-5.901285	-0.453438	3.812081
Cs	1.509409	-0.935496	-3.962520
H	-2.818033	-1.040670	-3.372353
H	-2.521740	0.748885	-3.706224
H	4.482953	3.805756	-1.094430
H	5.708197	-3.082304	1.532152
H	5.775318	-3.872103	3.104718
H	5.182622	-0.715263	0.576391
H	4.878561	-3.011829	-0.633032
H	5.216459	-0.852231	2.788012
H	3.609686	4.806163	0.066402
H	4.515860	0.154080	-0.800157
H	5.232408	-1.715175	4.323423
H	4.356079	-2.062047	-2.012531
H	3.772140	3.068884	0.342813
H	4.710363	-4.507320	1.849068
H	3.282683	2.231151	-2.634000
H	3.791860	0.353112	0.801403
H	3.339847	-3.380258	-1.401824
H	3.830688	-0.743421	3.869064
H	2.195006	4.380663	-1.941796
H	1.522507	6.429218	-1.067468
H	0.926082	7.519971	0.184442
H	2.471006	1.404257	-1.287108
H	3.462740	-3.225946	3.605926
H	1.765736	5.457486	1.226293
H	2.382915	1.999481	2.426776
H	2.416543	-1.165447	-0.673169
H	1.818804	3.654026	2.433822
H	2.795975	-4.659433	0.616637
H	1.513431	2.095687	-2.602916
H	-0.215180	6.611268	-0.802682
H	2.117488	0.447245	4.010451
H	1.155640	2.574678	4.537237
H	2.225098	-5.015414	3.014830
H	-0.063152	6.645516	2.335983
H	0.175128	3.726924	-3.169252
H	1.178000	-3.604264	-0.966911
H	1.756048	-1.995606	4.054184
H	-0.260805	4.913393	2.613001
H	1.108686	-5.907313	1.980343

H	0.987246	-0.037903	5.287879
H	0.528932	-5.117537	-0.321954
H	-0.655225	5.152506	-2.545893
H	-0.261309	3.001852	3.559071
H	-1.277519	5.739243	1.436115
H	-0.901994	2.321100	-1.394117
H	0.591267	-4.401464	2.738168
H	-0.054315	-3.578809	0.308596
H	-1.584378	3.828000	-3.229449
H	0.056897	-1.778318	3.728461
H	-2.150313	4.960265	-0.456604
H	-1.238661	1.479359	5.151224
H	-2.251906	3.443165	0.443612
H	-1.298641	-0.205352	4.599158
H	-1.771602	-2.889986	1.799573
H	-2.238569	2.182103	2.960014
H	-3.003017	3.585664	-1.152464
H	-2.463797	-4.082464	0.688165
H	-2.804547	-1.795448	-0.231753
H	-3.482834	1.276185	-0.726448
H	-3.255829	1.043438	3.809849
H	-3.323885	-3.648904	2.162052
H	-2.981487	-2.339467	4.094621
H	-4.423898	-0.104241	-1.281671
H	-3.465732	-0.906676	5.007036
H	-4.226515	2.532282	1.464618
H	-4.510113	-3.573940	-0.654656
H	-5.150683	1.502288	-1.254076
H	-5.276238	-1.986312	-0.634944
H	-4.412041	-2.384687	5.129829
H	-5.428525	-3.060677	0.757983
H	-5.875898	2.591527	0.847157
H	-5.192116	-2.202875	2.842259
H	-5.554095	1.885075	2.432499
H	-5.938656	0.102972	0.694080
H	-5.554658	0.468436	4.294073
H	-6.613797	-0.170960	3.030618
H	-6.463058	-1.014313	4.572415
H	-0.323562	-1.701838	-1.029413
H	-0.914413	-0.388849	-1.802237
H	-1.834530	-2.778937	-4.921980
H	-0.451065	-3.365619	-6.847015
H	0.661556	-1.597652	-8.236499
H	0.247801	0.804216	-7.644616
H	-1.132161	1.416658	-5.726056

Supplementary Table 39. Final coordinates and energy for Cs-TS-in

Energy: -2355.867972 a.u

Imaginary Frequency : -1329.6510

C	-10.361857	-3.402445	-4.313351
C	-9.735589	-2.450048	-5.126999
C	-9.749411	-1.114007	-4.707860
C	-10.343996	-0.745480	-3.505153
C	-10.963470	-1.693377	-2.642346
C	-10.958464	-3.038249	-3.110444
C	-11.460622	-1.337437	-1.329220
N	-9.838264	-1.706331	0.865204
U	-9.597930	-0.890669	2.842051
N	-11.679705	-0.923399	3.901610
C	-11.759908	-0.005333	5.051088
C	-10.636941	-0.272011	6.045136
N	-9.317019	-0.127064	5.403277
C	-8.853968	1.271963	5.397235
C	-7.973372	1.547557	4.182942
N	-8.705255	1.272846	2.937743
Si	-8.652697	2.577977	1.709338
C	-10.179296	2.348062	0.530774
C	-10.020905	3.001093	-0.848191
N	-8.061852	-2.329409	3.909269
Si	-6.752787	-3.417130	3.370676
C	-7.368696	-5.197908	2.848223
C	-7.903415	-6.007194	4.034117
C	-8.391861	-2.412737	5.343441
C	-8.324482	-1.038507	6.002023
C	-7.042838	2.481324	0.606169
C	-5.760840	2.769549	1.392323
C	-8.588590	4.352052	2.531638
C	-8.562631	5.479370	1.491359
C	-6.944497	1.134352	-0.111606
Si	-13.249269	-1.699659	3.513626
C	-14.141705	-2.522192	5.062485
C	-13.186808	-3.036267	6.144506
C	-12.965463	-3.157500	2.265208
C	-12.116514	-4.286342	2.857865
C	-14.495792	-0.458989	2.665899
C	-14.093184	-0.170624	1.216636
C	-14.281960	-3.729564	1.725771
C	-9.654037	4.633948	3.594889
C	-11.492410	2.806644	1.174414
C	-5.440011	-3.771989	4.782721
C	-4.315048	-4.699291	4.304685
C	-5.938414	-2.601891	1.799294
C	-5.025398	-1.423206	2.148592
C	-4.848299	-2.547149	5.486648
C	-5.210057	-3.581198	0.872144
C	-8.404365	-5.165344	1.724048
C	-14.671580	0.864536	3.419638
C	-15.284197	-1.726819	5.701326
Cs	-7.819652	-2.319692	-1.694499

H	-12.264348	-1.994591	-0.981907
H	-11.768303	-0.290038	-1.249517
H	-4.873852	2.715253	0.744921
H	-3.668660	-4.204929	3.570170
H	-3.670396	-4.993966	5.144429
H	-4.104360	-1.763360	2.634570
H	-4.353944	-4.047191	1.371735
H	-4.182472	-1.978426	4.829844
H	-5.766027	3.766804	1.845368
H	-4.720893	-0.870253	1.249233
H	-4.252764	-2.851373	6.358653
H	-4.803303	-3.065972	-0.012027
H	-5.612676	2.042887	2.199759
H	-4.690321	-5.621183	3.848384
H	-6.056168	1.089945	-0.759818
H	-5.516617	-0.713613	2.821491
H	-5.850730	-4.397340	0.520613
H	-5.616557	-1.856053	5.842796
H	-7.153831	3.263261	-0.158980
H	-7.776335	5.346656	0.740193
H	-8.386773	6.450540	1.974568
H	-6.868892	0.317232	0.615266
H	-6.008083	-4.332281	5.540596
H	-7.613930	4.373000	3.041792
H	-7.057173	0.937340	4.262144
H	-6.810737	-2.192392	1.264970
H	-7.628572	2.589635	4.266963
H	-6.474896	-5.713799	2.466241
H	-7.825639	0.941633	-0.734114
H	-9.516786	5.560156	0.958286
H	-7.330409	-0.620652	5.820967
H	-8.336488	1.512503	6.342314
H	-7.149490	-6.152706	4.815086
H	-9.451580	5.585766	4.106056
H	-9.137734	2.646763	-1.389100
H	-8.052958	-4.607314	0.848312
H	-7.721191	-3.079694	5.904356
H	-9.695012	3.855151	4.361305
H	-8.234855	-7.005670	3.716403
H	-8.469321	-1.099617	7.094370
H	-8.662622	-6.178812	1.385731
H	-9.941225	4.091712	-0.775985
H	-9.736942	1.914152	5.328875
H	-10.655673	4.712438	3.160756
H	-10.250162	1.260455	0.369607
H	-8.767227	-5.516597	4.498672
H	-9.331958	-4.691435	2.059610
H	-10.895826	2.789143	-1.477371
H	-9.399986	-2.830584	5.507440
H	-11.521720	3.897218	1.278890
H	-10.707965	0.384753	6.929351
H	-11.641133	2.374529	2.169285
H	-10.730082	-1.304967	6.391176
H	-11.168124	-3.925383	3.271866
H	-11.716174	1.051361	4.738105
H	-12.354047	2.523390	0.556704
H	-11.881282	-5.045422	2.098881
H	-12.409147	-2.721387	1.423777
H	-13.090994	0.267856	1.169617
H	-12.707048	-0.096190	5.606385
H	-12.644701	-4.805253	3.667319
H	-12.343358	-3.597630	5.731764
H	-14.082612	-1.065874	0.588776
H	-12.773590	-2.212744	6.737010
H	-13.738976	1.439464	3.426790
H	-14.091590	-4.509206	0.975389
H	-14.783528	0.546052	0.749605
H	-14.913610	-2.972437	1.251126
H	-13.711996	-3.698904	6.846912
H	-14.876348	-4.196782	2.521244
H	-15.429505	1.492914	2.930960
H	-14.602897	-3.407848	4.597620
H	-14.983572	0.731474	4.459759
H	-15.467378	-0.976791	2.656707
H	-14.920789	-0.837719	6.230558
H	-16.025650	-1.396031	4.967000
H	-15.815852	-2.339729	6.443344
H	-9.986769	-2.721132	0.966203
H	-10.522747	-1.480920	-0.197311
H	-11.450365	-3.799738	-2.506581
H	-10.398416	-4.443434	-4.631128
H	-9.288946	-2.733424	-6.076102
H	-9.303707	-0.345646	-5.338016
H	-10.351962	0.303488	-3.213677

Supplementary Table 40. Final coordinates and energy for Cs-product-in

Energy: -2084.546695 a.u

C	-12.738393	-0.686608	-4.404050
C	-11.296417	-1.185996	-4.270858
C	-10.542349	-0.358307	-3.227906
Si	-11.117804	-3.105376	-3.927721
C	-12.288025	-3.962263	-5.243367
C	-12.494301	-5.461745	-5.017188
N	-11.570948	-3.582733	-2.270298
C	-12.999456	-3.380145	-1.993995
C	-13.590949	-4.519885	-1.172331
N	-12.879418	-4.702673	0.103130
C	-12.984253	-6.089353	0.586888
C	-12.001973	-7.005057	-0.136528

N	-10.620258	-6.534349	0.014609	H	-8.484004	-7.306304	-2.487678
Si	-9.462756	-7.833256	0.396153	H	-7.459993	-6.385789	0.236225
C	-9.022968	-8.943098	-1.155433	H	-7.005065	-5.774395	2.591875
C	-10.259762	-9.394561	-1.939057	H	-7.946202	-5.298642	-4.397246
U	-10.290622	-4.224525	-0.376467	H	-12.297141	-7.081247	-1.196572
N	-10.991439	-2.977584	1.561304	H	-8.703925	-5.476357	2.200427
C	-12.242795	-3.463603	2.160885	H	-12.732391	-6.093609	1.651333
C	-13.322034	-3.731359	1.116602	H	-9.534040	-5.667576	-3.705322
N	-8.310014	-3.976611	-0.808527	H	-14.023119	-6.453238	0.486473
C	-9.244674	-3.551925	-4.200601	H	-9.367642	-5.356045	-5.434863
C	-9.016060	-5.047103	-4.443467	H	-12.081811	-4.384182	2.749550
Si	-10.273775	-1.635501	2.476889	H	-7.472159	-3.002437	-5.343355
C	-9.782591	-2.067472	4.323888	H	-9.452439	-4.211114	4.124519
C	-8.894710	-3.311157	4.404550	H	-11.599483	-3.054160	5.042600
C	-8.621596	-1.128561	1.575859	H	-8.789413	-3.311380	-3.227418
C	-7.582973	-0.454422	2.478604	H	-11.581052	-6.033551	-5.211908
C	-11.526115	-0.138358	2.635543	H	-13.486836	-5.443733	-1.748193
C	-10.923111	1.081566	3.340301	H	-8.963925	-2.880064	-6.272542
C	-8.857229	-0.289640	0.317446	H	-8.512731	-3.467736	5.423639
C	-7.783118	-7.080307	1.024857	H	-14.258999	-4.072736	1.593029
C	-6.699528	-8.146440	1.222138	H	-10.608541	-2.357039	6.321889
C	-10.065139	-9.015327	1.850553	H	-12.794374	-5.677925	-3.987088
C	-10.684749	-10.355269	1.439388	H	-12.694767	-2.750605	2.872390
C	-7.949424	-6.261903	2.306765	H	-8.028716	-3.250546	3.735696
C	-10.952180	-8.313185	2.885398	H	-8.572023	-1.647899	-5.071623
C	-8.049893	-8.236405	-2.103800	H	-13.273046	-5.860320	-5.683168
C	-8.534521	-2.723082	-5.276010	H	-11.598859	-1.307017	5.307815
C	-11.868484	-3.674650	-6.689004	H	-14.669433	-4.361126	-0.990876
C	-12.204684	0.262068	1.323482	H	-13.539004	-2.789060	0.604773
C	-10.962416	-2.198558	5.292911	H	-10.909998	-4.147681	-6.933686
Cs	-5.613991	-2.623252	-0.343993	H	-9.494482	-0.664614	-3.131505
H	-7.877768	-4.597721	-1.507587	H	-9.185292	-1.204616	4.656831
H	-10.859296	-10.991076	2.319567	H	-13.618000	-3.320082	-2.905798
H	-10.042238	-10.921546	0.756718	H	-8.226099	-2.103022	1.249231
H	-11.655657	-10.225598	0.946558	H	-13.182986	-2.428186	-1.464777
H	-8.522609	-9.838166	-0.754097	H	-13.267211	-3.482261	-5.094938
H	-10.989896	-9.921926	-1.317591	H	-12.317324	-0.529050	3.293537
H	-9.115334	-9.254297	2.354379	H	-11.001321	-0.466626	-2.238881
H	-9.983016	-10.069912	-2.761336	H	-12.607278	-4.068000	-7.401977
H	-6.962033	-8.844731	2.026839	H	-11.766436	-2.602248	-6.890818
H	-11.030866	-8.908982	3.806049	H	-10.806198	-1.032121	-5.244188
H	-6.515641	-8.744421	0.323725	H	-12.591183	-0.609232	0.785784
H	-7.815607	-8.865792	-2.974437	H	-7.279762	-1.082947	3.322060
H	-5.741889	-7.687529	1.509389	H	-9.579035	-0.754821	-0.359097
H	-10.770188	-8.534683	-2.387495	H	-10.552200	0.712622	-3.477754
H	-11.972054	-8.176676	2.509503	H	-10.478075	0.828334	4.309171
H	-7.100203	-7.979674	-1.622890	H	-13.296926	-0.802470	-3.468216
H	-10.575941	-7.323711	3.161892	H	-6.669840	-0.269000	1.915432
H	-8.245239	-6.893257	3.153644	H	-13.046323	0.945574	1.504411
H	-12.164964	-8.017241	0.269609	H	-7.926166	-0.146783	-0.253843

H	-13.295097	-1.212905	-5.186925
H	-11.514571	0.777130	0.646898
H	-7.952998	0.489681	2.894123
H	-9.224961	0.712884	0.563503
H	-11.686017	1.850780	3.527236
H	-10.141543	1.554383	2.733026
H	-12.761343	0.383260	-4.657448

Supplementary Table 41. Final coordinates and energy for 2Cs

Energy: -4169.110539 a.u

C	-1.143788	0.568651	6.147908
C	-1.577089	-0.678033	5.369565
C	-0.347757	-1.473805	4.917036
Si	-2.930119	-0.414440	3.964385
C	-4.012602	1.141529	4.444568
C	-5.388452	1.111161	3.771213
N	-2.266259	-0.310570	2.316454
C	-1.095916	0.578644	2.221160
C	0.039044	-0.076039	1.442054
N	-0.376721	-0.387643	0.063106
C	-0.253455	0.782084	-0.823949
C	-1.289238	0.747190	-1.944987
N	-2.668373	0.724397	-1.426646
Si	-3.695168	2.001566	-2.126749
C	-2.899122	3.776546	-1.916509
C	-3.767687	4.887770	-2.518915
U	-2.999227	-1.008118	0.169722
N	-5.011994	-1.398601	0.344781
N	-9.135876	-4.085931	-0.499038
U	-11.154871	-4.334154	-0.185044
N	-12.375294	-3.499464	-2.051446
Si	-12.001515	-3.138634	-3.754621
C	-13.261337	-3.990197	-4.987729
C	-12.943611	-3.684817	-6.456793
N	-1.736254	-2.918297	-0.505392
C	-0.337425	-2.858924	-0.048884
C	0.337544	-1.558809	-0.471727
Si	-2.132382	-4.422159	-1.375964
C	-2.158829	-6.011999	-0.233232
C	-0.785039	-6.417766	0.310316
C	-0.799469	-4.841911	-2.748245
C	-0.464456	-3.698223	-3.710485
C	-1.113860	-6.124169	-3.528585
C	-3.924470	-4.240104	-2.119627
C	-3.956233	-3.352449	-3.367110
C	-4.101365	-1.961676	4.112000
C	-3.357075	-3.274435	3.847850
C	-5.450321	1.874855	-1.289083
C	-6.623200	2.289490	-2.184982
C	-3.957831	1.811417	-4.061063
C	-2.762819	2.228065	-4.925729
C	-5.536817	2.611569	0.051137
C	-4.625271	-5.572468	-2.409312
C	-3.154029	-5.868260	0.918341
C	-4.399702	0.396213	-4.435370
C	-2.468874	4.135717	-0.491513
C	-4.845374	-2.031863	5.438657
C	-3.319062	2.469439	4.122367
N	-11.949989	-3.344386	1.838645
C	-13.112252	-4.074666	2.371477
C	-14.233829	-4.159484	1.342432
N	-13.784432	-4.888266	0.143659
C	-14.470170	-4.426173	-1.075846
C	-13.772150	-3.211655	-1.683599
Si	-11.523943	-1.904745	2.791796
C	-10.772296	-2.320919	4.550142
C	-11.700660	-3.169465	5.424934
C	-13.111410	-0.811333	3.159055
C	-13.687037	-0.118518	1.919448
C	-12.949710	0.194296	4.305818
C	-10.129548	-0.920640	1.859081
C	-10.494052	-0.503620	0.433823
N	-11.452920	-6.661243	0.017675
C	-12.787051	-7.072202	-0.447985
C	-13.888734	-6.348148	0.320083
Si	-10.350876	-8.008344	0.380370
C	-9.551011	-8.770661	-1.233465
C	-8.482672	-7.845087	-1.823616
C	-11.148428	-9.469680	1.426020
C	-12.254057	-9.024696	2.390870
C	-8.878462	-7.346889	1.466795
C	-9.343406	-6.805243	2.820899
C	-11.602180	-10.706369	0.642639
C	-10.162886	-3.693694	-4.087210
C	-9.430306	-2.870546	-5.153512
C	-12.121045	-1.229883	-4.188703
C	-13.547776	-0.681079	-4.303041
C	-10.027255	-5.186196	-4.405765
C	-9.623103	0.289621	2.651433
C	-9.391668	-2.972893	4.456111
C	-11.315952	-0.370064	-3.213043
C	-13.468957	-5.493377	-4.783342
C	-7.776145	-8.392159	1.667661
C	-10.592035	-9.102372	-2.307927
Cs	-7.351620	-1.670502	-1.944546
Cs	-6.737580	-3.932153	1.771085
H	-3.018132	2.183911	-5.994139
H	-1.992387	-6.001768	-4.173045
H	-0.276624	-6.396547	-4.186335

H	-3.454228	-3.834415	-4.212980
H	-4.100250	-6.150052	-3.178159
H	-1.275366	-3.509577	-4.421998
H	-2.429059	3.249985	-4.718549
H	-4.988540	-3.159249	-3.696574
H	0.430641	-3.933363	-4.303212
H	-5.646425	-5.409774	-2.784909
H	-1.903220	1.564458	-4.778021
H	-1.301827	-6.982946	-2.875137
H	-4.659151	0.319785	-5.501148
H	-3.471982	-2.384865	-3.203672
H	-4.706185	-6.213816	-1.525990
H	-0.274477	-2.758087	-3.184535
H	-4.784584	2.497427	-4.298164
H	-4.039990	4.691973	-3.561886
H	-3.243555	5.853584	-2.498372
H	-3.600798	-0.326036	-4.237827
H	0.114586	-5.043313	-2.169315
H	-1.980363	3.725182	-2.520425
H	-1.085663	-0.125965	-2.588719
H	-4.484287	-3.718189	-1.326568
H	-1.076328	1.622087	-2.580788
H	-2.510385	-6.826047	-0.884135
H	-5.275519	0.071450	-3.861263
H	-4.699301	5.021996	-1.956540
H	0.310066	-1.499351	-1.563136
H	0.772174	0.851118	-1.227661
H	-0.045591	-6.570360	-0.483096
H	-1.841200	5.037855	-0.485096
H	-6.674423	1.721385	-3.120627
H	-4.169223	-5.664757	0.557014
H	0.290451	-3.674008	-0.444351
H	-1.894883	3.331017	-0.021213
H	-0.849176	-7.358483	0.875499
H	1.398627	-1.535815	-0.165707
H	-3.200480	-6.782315	1.528173
H	-6.567729	3.348631	-2.462201
H	-0.430564	1.681099	-0.227000
H	-3.327167	4.340607	0.156516
H	-5.534987	0.800270	-1.062714
H	-0.383180	-5.662165	0.994962
H	-2.866121	-5.044024	1.578723
H	-7.583117	2.158241	-1.663004
H	-0.265552	-2.954090	1.048536
H	-5.524546	3.699177	-0.083719
H	0.944808	0.556018	1.436014
H	-4.711208	2.353490	0.720967
H	0.293111	-1.015978	1.940095
H	-2.820448	-3.266648	2.894163
H	-1.346716	1.539547	1.739641
H	-6.473000	2.370171	0.574236
H	-4.041313	-4.137295	3.845012
H	-4.834714	-1.820263	3.292283
H	-5.290511	1.075462	2.680186
H	-0.685723	0.860228	3.204111
H	-2.621513	-3.473148	4.636173
H	-0.608078	-2.360391	4.331341
H	-5.991367	0.249099	4.074408
H	0.319107	-0.863015	4.299304
H	-3.163671	2.576336	3.042992
H	-5.550092	-2.876584	5.462561
H	-5.968347	2.012811	4.014144
H	-5.419945	-1.127288	5.661134
H	0.241204	-1.809728	5.782203
H	-4.152396	-2.187573	6.274592
H	-3.931355	3.322770	4.446294
H	-2.123474	-1.313010	6.084670
H	-2.342239	2.568726	4.604943
H	-4.161253	1.086799	5.533640
H	-0.552289	1.255829	5.531122
H	-1.994992	1.131167	6.545042
H	-0.512302	0.292604	7.004565
H	-5.734356	-0.838702	0.816289
H	-13.538591	0.367708	-4.632734
H	-12.200748	0.961781	4.079017
H	-13.895453	0.720792	4.496812
H	-11.286330	0.250951	0.425239
H	-10.383957	1.076264	2.706136
H	-13.077203	0.741882	1.620057
H	-14.159620	-1.237015	-5.021184
H	-9.630029	-0.053457	-0.077422
H	-14.697906	0.265487	2.115045
H	-8.740783	0.738429	2.173475
H	-14.068034	-0.704652	-3.338706
H	-12.653580	-0.283259	5.245446
H	-11.286976	0.682393	-3.529958
H	-10.840286	-1.343619	-0.177640
H	-9.340819	0.038910	3.679256
H	-13.752270	-0.791601	1.057911
H	-11.656010	-1.140135	-5.181943
H	-12.845007	-2.611836	-6.654157
H	-13.734573	-4.063658	-7.119204
H	-11.755395	-0.400285	-2.210826
H	-13.861837	-1.545334	3.490014
H	-14.222695	-3.505663	-4.758610
H	-13.834224	-2.375775	-0.964833
H	-9.304228	-1.645951	1.783542
H	-14.392308	-2.895047	-2.537884
H	-10.649033	-1.346565	5.044846
H	-10.278367	-0.711658	-3.118691

H	-12.010103	-4.164626	-6.775038
H	-14.499158	-3.140395	1.044330
H	-15.531979	-4.212356	-0.861007
H	-12.698262	-2.727947	5.529853
H	-14.339209	-5.850181	-5.351854
H	-9.423066	-1.795563	-4.940969
H	-8.662695	-2.307755	3.978334
H	-13.546355	-3.620798	3.276515
H	-13.636292	-5.746062	-3.731906
H	-11.291453	-3.287103	6.438402
H	-15.140631	-4.630488	1.762731
H	-8.996847	-3.222002	5.451684
H	-9.889051	-2.991846	-6.141803
H	-14.442254	-5.237770	-1.808064
H	-12.606635	-6.075789	-5.122880
H	-9.676377	-3.534381	-3.111624
H	-11.829591	-4.176183	5.011382
H	-9.440554	-3.906188	3.881515
H	-8.384781	-3.198806	-5.257353
H	-12.836845	-5.097384	2.678730
H	-10.435724	-5.424671	-5.394257
H	-14.891528	-6.700195	0.021243
H	-10.542441	-5.816228	-3.674509
H	-13.762079	-6.570916	1.383228
H	-10.113848	-6.033570	2.718882
H	-12.924299	-6.878054	-1.526253
H	-8.971861	-5.493179	-4.416960
H	-8.506389	-6.373580	3.392250
H	-8.471482	-6.507370	0.882994
H	-8.909109	-6.866864	-2.073975
H	-12.969645	-8.152713	-0.333439
H	-9.758353	-7.602768	3.448567
H	-11.994997	-8.119372	2.947836
H	-7.647024	-7.673579	-1.136991
H	-13.190385	-8.822096	1.859649
H	-11.097998	-8.194626	-2.655392
H	-6.919846	-7.974251	2.218071
H	-8.061619	-8.263382	-2.748966
H	-7.390488	-8.793514	0.725198
H	-12.472048	-9.813214	3.124924
H	-8.133576	-9.244052	2.258696
H	-10.120661	-9.565417	-3.186330
H	-10.296222	-9.792451	2.045081
H	-11.363949	-9.791214	-1.952554
H	-9.062596	-9.708043	-0.925727
H	-12.471902	-10.495745	0.008851
H	-10.812638	-11.107614	-0.001020
H	-11.899037	-11.512433	1.328867
H	-8.485734	-4.667277	-1.042437

Supplementary Table 42. Final coordinates and energy for 3Cs

Energy: -2778.184802 a.u

U	-10.243029	-4.196022	-0.326641
Cs	-5.350705	-2.555219	-0.593583
Si	-9.426752	-7.804874	0.470857
Si	-10.328906	-1.602322	2.540459
Si	-11.011589	-3.074699	-3.892041
N	-10.577840	-6.510652	0.061700
N	-12.846747	-4.692574	0.096435
N	-11.002375	-2.948925	1.597880
N	-11.495691	-3.564178	-2.248746
N	-8.264631	-3.951429	-0.736768
N	-3.213303	-2.519729	2.063383
N	-3.370408	-4.462064	-2.453022
N	-1.836446	-2.178699	-0.789372
N	-3.705197	0.182576	-1.748122
C	-7.710241	-0.288323	2.638929
C	-8.954281	-3.246397	4.496200
C	-11.063120	1.100298	3.405076
C	-11.095246	-2.230577	5.328063
C	-9.891067	-2.040424	4.399866
C	-12.267958	0.265828	1.350512
C	-11.616120	-0.129608	2.677632
C	-10.480269	-0.327524	-3.163098
C	-12.339265	-5.437470	-5.026614
C	-8.877851	-4.990609	-4.372574
C	-11.694367	-3.634722	-6.673547
C	-12.143593	-3.934596	-5.239471
C	-8.408001	-2.655684	-5.184167
C	-9.128965	-3.499645	-4.128126
C	-6.682135	-8.109588	1.359639
C	-10.168308	-9.380124	-1.876370
C	-10.657025	-10.334376	1.490304
C	-7.961216	-8.211004	-1.995839
C	-8.951346	-8.919688	-1.067450
C	-10.970975	-8.293839	2.927732
C	-10.055992	-8.989712	1.913220
C	-11.952458	-6.988706	-0.123441
C	-12.956768	-6.079444	0.577049
C	-13.315800	-3.724437	1.100231
C	-12.260487	-3.452333	2.167494
C	-7.967221	-6.233060	2.416600
C	-7.764389	-7.047163	1.137223
C	-12.930643	-3.371512	-2.003526
C	-13.531747	-4.514270	-1.193517
C	-8.864760	-0.276982	0.406007
C	-8.657338	-1.053718	1.708607
C	-12.645376	-0.668803	-4.391288
C	-11.201730	-1.154678	-4.229268

C -3.877042 -5.813064 -2.237703
C -3.726891 -1.693055 3.148943
C -3.308910 -3.925351 2.437917
C -1.835890 -2.135677 1.751840
C -1.266296 -2.707005 0.454233
C -3.272842 0.429341 -3.116048
C -3.562356 -4.087975 -3.848946
C -1.962285 -4.388769 -2.063292
C -1.381447 -2.979536 -1.936530
C -1.478528 -0.760805 -0.975289
C -2.605550 0.256285 -0.787432
C -4.738046 1.144770 -1.385888
H -12.673661 0.402748 -4.637191
H -10.286078 1.602443 2.815745
H -11.852396 1.844347 3.585290
H -9.292653 0.715289 0.590629
H -8.140623 0.663474 2.971275
H -11.568072 0.792382 0.692689
H -13.179154 -1.194747 -5.190292
H -7.911882 -0.114440 -0.119853
H -13.123221 0.937411 1.512648
H -6.768981 -0.038986 2.125369
H -13.223431 -0.797603 -3.469192
H -10.627542 0.852121 4.379516
H -10.496314 0.745420 -3.404552
H -9.526786 -0.802744 -0.287264
H -7.448457 -0.856682 3.537001
H -12.628311 -0.608921 0.800647
H -10.691798 -0.988803 -5.190504
H -11.598574 -2.560232 -6.867376
H -12.412248 -4.031751 -7.405712
H -10.959490 -0.449050 -2.185338
H -12.416289 -0.542822 3.310803
H -13.130572 -3.465150 -5.110403
H -13.133259 -2.420082 -1.480173
H -8.180958 -2.010452 1.444701
H -13.529785 -3.317679 -2.928826
H -9.340953 -1.158571 4.762492
H -9.431772 -0.623828 -3.044751
H -10.725388 -4.096488 -6.897625
H -13.525824 -2.782393 0.585201
H -14.615160 -4.362415 -1.035486
H -11.771407 -1.368409 5.326301
H -13.103965 -5.839387 -5.706843
H -8.457317 -1.582882 -4.969901
H -8.066716 -3.135765 3.862885
H -12.734880 -2.748971 2.874050
H -12.651523 -5.663332 -4.002346
H -10.769250 -2.380205 6.367671
H -14.261453 -4.070778 1.556021
H -8.606180 -3.404968 5.527247
H -8.819195 -2.806095 -6.189425
H -13.409636 -5.437625 -1.766898
H -11.417525 -5.999099 -5.211266
H -8.698596 -3.261046 -3.143130
H -11.685579 -3.111936 5.052685
H -9.461230 -4.164055 4.179489
H -7.342233 -2.925395 -5.236712
H -12.102603 -4.375395 2.753560
H -9.206110 -5.301767 -5.371450
H -13.991178 -6.448460 0.450348
H -9.498000 -5.619550 -3.644696
H -12.732419 -6.083545 1.647595
H -8.715039 -5.444443 2.288904
H -12.222293 -7.063907 -1.190420
H -7.805609 -5.228572 -4.306654
H -7.031715 -5.747973 2.731077
H -7.422980 -6.348695 0.359733
H -8.388648 -7.280927 -2.387465
H -12.119729 -8.002927 0.276555
H -8.290623 -6.866488 3.251788
H -10.607579 -7.302366 3.213946
H -7.023930 -7.953523 -1.492192
H -11.982931 -8.163517 2.528830
H -10.669347 -8.524869 -2.343595
H -5.734130 -7.646866 1.671479
H -7.706281 -8.839406 -2.861604
H -6.473468 -8.705623 0.465104
H -11.066865 -8.891244 3.845830
H -6.961513 -8.810689 2.156345
H -9.871240 -10.061771 -2.686406
H -9.116172 -9.222089 2.438469
H -10.911997 -9.903520 -1.267822
H -8.455150 -9.811143 -0.652905
H -11.618977 -10.212101 0.978336
H -9.996940 -10.895866 0.820615
H -10.844281 -10.971522 2.367026
H -7.745962 -4.560394 -1.383342
H -3.714665 -0.636817 2.860497
H -4.761256 -1.971287 3.372759
H -3.143651 -1.795363 4.083351
H -2.996616 -4.576134 1.616354
H -2.690075 -4.175043 3.320536
H -4.347293 -4.174214 2.678071
H -4.934422 -5.864495 -2.515265
H -3.339263 -6.576240 -2.830741
H -3.790488 -6.087873 -1.181126
H -4.621571 -4.169057 -4.112636
H -3.258584 -3.052473 -4.024425
H -2.991921 -4.732809 -4.544055

H -2.790122 1.417125 -3.243282
H -2.567337 -0.335495 -3.451061
H -4.138611 0.391313 -3.784740
H -5.596787 1.046497 -2.058233
H -5.083074 0.967093 -0.361818
H -4.388155 2.193155 -1.437792
H -3.037511 0.133138 0.212343
H -2.135807 1.261762 -0.804987
H -1.035663 -0.623969 -1.968098
H -0.680097 -0.475399 -0.272525
H -1.792064 -1.041544 1.719065
H -1.152260 -2.441747 2.568453
H -1.392861 -3.792766 0.462862
H -0.172180 -2.532306 0.481001
H -1.850367 -4.926641 -1.115941
H -1.328067 -4.932908 -2.791195
H -0.278797 -3.086843 -1.927225
H -1.620918 -2.422060 -2.846522

Supplementary Table 43. Final coordinates and energy for Cs-TS-dis

Energy: -4169.067079 a.u
Imaginary Frequency : -1380.7640
U -11.390544 -4.410913 -0.264636
U -4.913327 -2.275166 -0.177193
Cs -7.741344 -2.252142 -2.868781
Cs -7.673268 -4.496707 2.416417
Si -11.003378 -8.176915 0.557852
Si -12.012613 -1.937711 2.614650
Si -5.538008 1.260522 -2.198370
Si -3.830700 -5.672948 -1.749644
Si -12.308778 -3.543779 -3.961461
Si -3.784073 -1.808092 3.639652
N -11.820787 -6.696944 -0.036325
N -14.092100 -4.851952 -0.065927
N -12.261218 -3.460252 1.692434
N -9.217776 -4.132301 -0.415933
N -6.739880 -2.982090 -0.103119
N -3.753131 -1.559931 1.890019
N -2.293529 -1.440896 -0.700985
N -4.899227 -0.366925 -1.862339
N -12.509156 -3.613795 -2.179298
N -3.516915 -4.122700 -0.960349
C -13.308947 -8.852419 2.323540
C -12.122444 -9.449615 1.559818
C -10.138559 -9.149950 -0.893056
C -8.904458 -8.402970 -1.402719
C -13.113545 -7.020372 -0.681353
C -14.267874 -6.311379 0.012331
C -14.553696 -4.184669 1.161734
C -13.440888 -4.192893 2.196120
C -11.540877 -2.248800 4.489484
C -12.590435 -2.986266 5.328392
C -9.614532 -7.671277 1.818185
C -10.194348 -7.012281 3.074490
C -12.575556 -10.702108 0.801563
C -10.517755 -0.993027 1.829974
C -10.684290 -0.636158 0.349004
C -13.657452 -0.872924 2.679751
C -14.011287 -0.180682 1.359200
C -2.861254 -0.462437 1.486688
C -1.742781 -0.948932 0.570140
C -2.395911 -0.391529 -1.726197
C -3.639628 -0.606415 -2.590256
C -4.369735 2.791088 -1.751161
C -4.973464 4.138063 -2.174026
C -13.847977 -3.129114 -1.782031
C -14.711864 -4.270932 -1.268131
C -2.120211 -3.896966 -0.570663
C -1.570233 -2.619488 -1.196450
C -5.873012 1.620748 -4.119050
C -4.649603 1.956591 -4.983149
C -7.260746 1.414736 -1.293554
C -8.271316 2.349030 -1.969712
C -4.225825 -7.129627 -0.500159
C -2.999884 -7.632310 0.268482
C -5.413882 -5.449117 -2.858160
C -5.136142 -4.610357 -4.109439
C -2.306379 -6.363295 -2.776973
C -1.647943 -5.390790 -3.759603
C -13.803139 -4.384505 -4.916812
C -13.754156 -4.123194 -6.428790
C -12.302184 -1.696941 -4.623338
C -13.657889 -0.979415 -4.576824
C -10.603212 -4.365947 -4.408567
C -9.961497 -3.815458 -5.688314
C -2.096214 -1.567927 4.632978
C -1.892690 -0.225441 5.343793
C -4.279806 -3.653479 4.021421
C -3.217100 -4.654970 3.561641
C -5.117325 -0.659537 4.499963
C -6.545390 -1.107359 4.177056
C -11.246559 -0.827378 -3.939311
C -10.650556 -5.896681 -4.475236
C -8.716175 -8.850169 2.211482
C -11.067106 -9.460460 -2.071765
C -10.118750 0.250190 2.634161
C -13.695612 0.148753 3.823811
C -10.185120 -2.935766 4.645243
C -6.790000 0.546382 -4.824894

C -7.123609 1.785516 0.186374
C -4.652356 -3.908509 5.485487
C -6.129373 -6.746496 -3.240089
C -2.624539 -7.685744 -3.487419
C -5.329753 -6.722201 0.475171
C -14.032508 -5.878893 -4.665223
C -3.862813 2.907594 -0.312092
C -4.952406 0.809713 4.096024
C -0.820479 -1.945763 3.871422
H -8.889203 -4.976660 -0.912460
H -13.029874 -11.426233 1.492521
H -11.748966 -11.213938 0.299062
H -13.334966 -10.471238 0.045076
H -9.807183 -10.104716 -0.457270
H -11.955363 -10.028224 -1.781119
H -11.400603 -9.794173 2.316567
H -10.541194 -10.048189 -2.836932
H -9.277573 -9.622275 2.751172
H -13.669458 -9.553038 3.089343
H -8.245911 -9.333523 1.349764
H -8.423613 -8.951385 -2.223677
H -7.909440 -8.527811 2.885041
H -11.408297 -8.539073 -2.556655
H -14.154377 -8.661629 1.654145
H -8.148136 -8.251581 -0.626141
H -13.068663 -7.911782 2.828812
H -10.862472 -7.699536 3.605355
H -13.333623 -8.099521 -0.681502
H -9.178576 -7.415994 -1.794708
H -8.996058 -6.942167 1.274982
H -9.408061 -6.748101 3.798449
H -9.637589 -6.317223 -4.525034
H -13.113801 -6.735453 -1.745737
H -10.773364 -6.108944 2.851104
H -14.259239 -6.598159 1.065695
H -11.149255 -6.342102 -3.608844
H -15.243974 -6.609078 -0.406430
H -11.177154 -6.240435 -5.371455
H -13.185797 -5.236155 2.439678
H -8.991104 -4.294509 -5.881272
H -10.219569 -3.946024 4.219138
H -12.653524 -4.044677 5.051260
H -9.961491 -4.111316 -3.551927
H -13.305514 -6.496431 -5.201249
H -14.760670 -5.039884 -2.042887
H -10.586206 -4.016796 -6.565820
H -9.908791 -3.040450 5.704041
H -15.478427 -4.655690 1.538178
H -12.328605 -2.953059 6.395289
H -13.964461 -6.150514 -3.607697
H -13.853293 -3.777161 3.127005
H -9.384178 -2.370372 4.157067
H -9.797283 -2.733114 -5.659592
H -15.027718 -6.183052 -5.017733
H -13.594520 -2.559712 5.230619
H -15.745592 -3.949425 -1.055851
H -14.788485 -3.143582 0.920626
H -12.897218 -4.622344 -6.897177
H -10.235573 -1.205598 -4.118129
H -11.443693 -1.235267 4.905360
H -14.395172 -2.632926 -2.596364
H -9.682179 -1.707116 1.888693
H -13.769477 -2.363264 -0.993565
H -14.686709 -3.855954 -4.527730
H -14.449080 -1.610359 2.881169
H -11.402538 -0.781177 -2.855494
H -14.654920 -4.516380 -6.920062
H -13.691767 -3.059322 -6.677400
H -12.020856 -1.787442 -5.683312
H -13.939470 -0.850855 0.496460
H -9.987603 0.040729 3.700624
H -11.064569 -1.469353 -0.254430
H -11.269852 0.202943 -4.320294
H -13.581544 -0.311202 4.809812
H -13.943799 -0.725391 -3.550202
H -9.172861 0.666058 2.268714
H -15.037272 0.211941 1.384684
H -9.722266 -0.336158 -0.084278
H -14.472243 -1.567463 -5.012622
H -13.351581 0.672544 1.166438
H -10.873310 1.040907 2.547443
H -11.382178 0.195829 0.209082
H -14.654636 0.685476 3.829808
H -12.909878 0.906349 3.723757
H -13.611327 -0.032407 -5.132197
H -8.103429 -3.539142 -0.246174
H -1.002580 -0.261157 5.988783
H -2.742 0.045333 5.981905
H -1.733663 0.596288 4.635407
H -4.967288 -0.741681 5.587220
H -3.967212 1.211762 4.347545
H -2.219368 -2.318372 5.430557
H -5.700653 1.444272 4.592374
H -3.785853 -3.775213 6.144464
H 0.023722 -2.069459 4.564877
H -5.437710 -3.240491 5.855131
H -7.283917 -0.429380 4.627240
H -4.997529 -4.942340 5.638023
H -5.089594 0.932874 3.015750
H -0.531239 -1.162685 3.162829

H -6.760598 -2.112790 4.556547
H -0.918188 -2.878227 3.307934
H -2.294738 -4.550418 4.144602
H -2.392153 0.064343 2.335099
H -6.719104 -1.104088 3.093135
H -5.168379 -3.822572 3.401491
H -3.557437 -5.692360 3.694173
H -8.095841 1.727552 0.692523
H -3.415133 0.322766 0.948024
H -2.962959 -4.518774 2.506156
H -1.235341 -1.778849 1.068138
H -6.435688 1.121303 0.719555
H -0.990154 -0.160708 0.387411
H -6.764770 2.812525 0.312818
H -2.009719 -3.810243 0.524586
H -9.221504 2.359808 -1.417112
H -4.973486 -5.910038 1.120421
H -2.542160 -6.834699 0.866235
H -7.677052 0.396573 -1.317016
H -4.658772 3.186872 0.385165
H -2.490749 0.576574 -1.227641
H -7.912099 3.383971 -1.998886
H -5.626926 -7.562571 1.120672
H -0.484310 -2.509842 -1.020392
H -3.272405 -8.438078 0.966354
H -3.426247 1.977363 0.057857
H -1.441077 -4.716732 -0.858070
H -6.220429 -6.353837 -0.046021
H -8.500332 2.058175 -3.321000
H -3.087556 3.683776 -0.237127
H -2.224336 -8.032591 -0.393117
H -1.473645 -0.357596 -2.334857
H -1.723578 -2.680610 -2.277988
H -5.849646 4.391788 -1.565050
H -7.667429 0.384751 -4.335480
H -4.601219 -7.963417 -1.110969
H -3.525683 0.018448 -3.490984
H -6.078812 -4.876492 -2.190509
H -3.597747 -1.644042 -2.971283
H -3.482947 2.620528 -2.381591
H -1.553390 -6.592402 -2.008090
H -6.157858 -0.407193 -4.864408
H -4.247222 4.951549 -2.033009
H -5.284600 4.166059 -3.222648
H -6.493336 2.528372 -4.087259
H -1.357037 -4.451246 -3.283435
H -6.403844 -7.341862 -2.365666
H -4.640953 -3.661208 -3.874757
H -6.920618 0.829283 -5.864610
H -3.094462 -8.423782 -2.828711
H -4.040979 1.069114 -5.190849
H -7.054962 -6.539650 -3.796977
H -0.738630 -5.830442 -4.194640
H -6.061388 -4.387812 -4.665036
H -3.991934 2.702979 -4.527675
H -2.309333 -5.139378 -4.595716
H -5.513006 -7.381703 -3.886196
H -4.489431 -5.147694 -4.811995
H -1.709422 -8.145740 -3.886980
H -3.296252 -7.532859 -4.340731
H -4.962110 2.356888 -5.958772

Supplementary Table 44. Final coordinates and energy for Cs-product-dis

Energy: -4169.113815 a.u
U -12.510540 -4.721583 -0.222440
U -3.701785 -1.930131 -0.256495
Cs -7.785090 -3.999780 1.499390
Cs -7.567668 -2.506351 -2.645455
Si -11.451026 -8.317796 0.431281
Si -13.593069 -3.586094 -3.743449
Si -12.798740 -2.170872 2.639792
Si -2.894888 -5.303211 -1.782378
Si -4.221689 1.250401 -2.402906
Si -3.579409 -1.626246 3.579531
N -13.904969 -4.039725 -2.054344
N -13.267273 -3.644109 1.769812
N -15.079399 -5.430817 0.258928
N -12.668345 -7.078798 0.087522
N -10.171711 -4.075398 -0.705995
N -2.447413 -3.799093 -0.933120
N -5.438201 -2.438605 -0.292704
N -3.322990 -0.174964 -1.816011
N -1.097849 -1.298234 -0.275586
N -2.996789 -1.301022 1.929228
C -12.460083 -11.064405 1.028939
C -11.855979 -9.643938 -2.131829
C -10.660942 -3.183750 4.342446
C -8.739246 -8.463685 1.462425
C -10.747692 -0.097473 2.357542
C -14.148229 0.086051 3.986541
C -14.697051 -6.126669 -4.756234
C -13.287393 -0.751513 -3.196161
C -11.308194 -5.290599 -4.365523
C -9.806850 -8.224659 -1.955187
C -10.747137 -9.161151 -1.190357
C -10.289886 -6.917161 2.688785
C -9.920439 -7.496791 1.321050
C -13.071575 -9.281568 2.690980

C -12.030021 -9.737125 1.662543
C -15.475762 -1.364832 -4.258728
C -13.985873 -1.711660 -4.161979
C -11.144651 -2.940647 -5.238093
C -11.690320 -3.828724 -4.113602
C -14.463301 -4.274311 -6.452795
C -14.714217 -4.611625 -4.978584
C -11.791403 -0.869928 0.209537
C -11.390387 -1.282189 1.627238
C -14.996789 -0.485101 1.683050
C -14.362548 -1.036006 2.964677
C -15.849591 -5.137951 -0.957546
C -15.322200 -3.903605 -1.684033
C -12.948864 -3.221942 5.375749
C -12.019140 -2.481978 4.409028
C -14.382678 -4.377019 2.389849
C -15.522690 -4.619386 1.403760
C -15.081907 -6.869565 0.570578
C -14.004692 -7.604956 -0.221839
C -3.009068 3.314111 -0.708105
C -5.659376 0.097584 -4.666347
C -5.868579 1.365980 0.025384
C -4.681611 -6.333220 0.243049
C -5.186606 -6.157215 -3.396833
C -1.729115 -7.319361 -3.553525
C -5.136222 -3.683762 4.929176
C -4.563768 1.083869 3.809336
C -4.120157 -3.976880 -4.068714
C -4.437040 -4.916131 -2.902197
C -0.675177 -5.038828 -3.706163
C -1.384186 -6.037095 -2.785913
C -2.306992 -7.160523 0.374180
C -3.439482 -6.725912 -0.560870
C -0.366724 -2.486113 -0.762042
C -1.087254 -3.771762 -0.377293
C -3.405433 1.045130 -5.218596
C -4.636612 1.180123 -4.316719
C -6.835512 2.473581 -2.011452
C -5.940982 1.336727 -1.502257
C -6.300218 -0.666846 3.365720
C -4.981511 -0.364482 4.085737
C -3.599710 4.065194 -3.033837
C -3.155244 2.878212 -2.170427
C -1.946091 -0.246310 -2.333793
C -0.932828 -0.158058 -1.199357
C -0.710517 -0.942045 1.105079
C -1.895495 -0.331689 1.844851
C -3.285856 -4.512001 3.454037
C -4.339955 -3.417164 3.645955
C -2.058054 -0.323790 5.795641
C -2.215029 -1.620344 4.992744
C -0.834704 -2.142483 4.573782
H -9.909328 -4.660529 -1.506874
H -12.620252 -11.830519 1.801336
H -11.711832 -11.460205 0.334347
H -13.404774 -10.969526 0.480225
H -10.167730 -10.037513 -0.862148
H -12.539973 -10.353038 -1.655768
H -11.102230 -9.944507 2.218740
H -11.436104 -10.142246 -3.017377
H -8.996770 -9.324670 2.091516
H -13.171587 -10.018439 3.500580
H -8.391139 -8.858900 0.502935
H -9.431375 -8.700252 -2.872652
H -7.878116 -7.977834 1.945455
H -12.456187 -8.800311 -2.490554
H -14.062119 -9.174446 2.236190
H -8.934310 -7.922896 -1.366003
H -12.821424 -8.319941 3.148486
H -10.612691 -7.703214 3.381511
H -14.116351 -8.678497 0.006199
H -10.331778 -7.311072 -2.259828
H -9.614643 -6.665637 0.666958
H -9.428667 -6.426414 3.169138
H -10.215680 -5.424169 -4.384749
H -14.240603 -7.520807 -1.296842
H -11.101265 -6.184537 2.619489
H -14.858802 -6.987033 1.634996
H -11.709529 -5.961484 -3.599642
H -16.082425 -7.303994 0.390025
H -11.680426 -5.640727 -5.334900
H -14.052781 -5.350095 2.792230
H -10.073638 -3.134117 -5.410216
H -10.751844 -4.174543 3.881605
H -13.123724 -4.254401 5.052942
H -11.204379 -3.523920 -3.173460
H -13.758622 -6.579591 -5.094045
H -15.750687 -5.993464 -1.632267
H -11.644526 -3.134877 -6.193458
H -10.235356 -3.328090 5.346430
H -16.389125 -5.092754 1.901468
H -12.513926 -3.270474 6.384383
H -14.818075 -6.385632 -3.699751
H -14.829469 -3.855882 3.252560
H -9.931458 -2.604315 3.763890
H -11.255881 -1.872174 -5.026566
H -15.506901 -6.616750 -5.315057
H -13.927568 -2.738143 5.471013
H -16.924013 -5.028637 -0.720583
H -15.851980 -3.647009 1.024537

H -13.476599 -4.620281 -6.784058
H -12.210911 -0.945497 -3.118110
H -11.853786 -1.474131 4.817573
H -15.991894 -3.761682 -2.549694
H -10.626696 -2.070732 1.530303
H -15.493540 -3.016019 -1.049136
H -15.733906 -4.271756 -4.740865
H -15.093776 -1.731377 3.405344
H -13.703744 -0.839517 -2.187185
H -15.202761 -4.763768 -7.102231
H -14.517991 -3.198934 -6.655546
H -13.553838 -1.555446 -5.162073
H -15.094967 -1.252106 0.907267
H -10.411302 -0.349205 3.368597
H -12.299906 -1.676478 -0.331681
H -13.407829 0.295348 -3.510260
H -13.795603 -0.287487 4.953758
H -15.980041 -1.457426 -3.290122
H -9.873108 0.278034 1.806743
H -15.999638 -0.080433 1.879475
H -10.912178 -0.567295 -0.379853
H -16.011568 -2.001811 -4.970621
H -14.403996 0.334123 1.260643
H -11.444539 0.743219 2.448923
H -12.471710 -0.012535 0.218951
H -15.085426 0.629292 4.173276
H -13.418695 0.826012 3.636985
H -15.615562 -0.325761 -4.589884
H -10.284539 -3.137925 -1.107304
H -1.377503 -0.478266 6.645081
H -3.007210 0.034422 6.207016
H -1.630623 0.486015 5.192285
H -5.144518 -0.478362 5.168037
H -3.652605 1.371359 4.342313
H -2.636347 -2.363528 5.689008
H -5.352664 1.787406 4.110326
H -4.487985 -3.649386 5.813460
H -0.222456 -2.368658 5.457983
H -5.941140 -2.961075 5.099179
H -7.052102 0.107742 3.572700
H -5.587067 -4.687263 4.918699
H -4.381077 1.239038 2.740427
H -0.282291 -1.395737 3.993417
H -6.730597 -1.622725 3.684031
H -0.887208 -3.053566 3.971016
H -2.590607 -4.545850 4.300872
H -1.536834 0.010887 2.827504
H -6.158640 -0.706763 2.279107
H -5.023596 -3.461894 2.785520
H -3.748673 -5.506233 3.387494
H -6.869078 1.263564 0.467680
H -2.198685 0.592022 1.321355
H -2.696569 -4.359063 2.544626
H -0.424644 -1.859738 1.625855
H -5.256812 0.553726 0.429725
H 0.166427 -0.272703 1.091584
H -5.451616 2.307884 0.394268
H -1.087542 -3.860224 0.723659
H -7.843630 2.404552 -1.578618
H -4.498952 -5.417583 0.816748
H -1.989466 -6.340185 1.027821
H -6.419488 0.384530 -1.775517
H -3.929047 3.777859 -0.334065
H -1.122647 0.765359 -0.644656
H -6.438509 3.453680 -1.723951
H -4.964858 -7.126299 0.950291
H 0.671109 -2.470378 -0.389267
H -2.624370 -7.986451 1.026366
H -2.769936 2.477033 -0.043281
H -0.457876 -4.605534 -0.724536
H -5.544027 -6.146524 -0.407280
H -6.948186 2.476327 -3.101110
H -2.211491 4.061815 -0.597656
H -1.423441 -7.506412 -0.173501
H 0.105799 -0.119084 -1.570077
H -0.325966 -2.431614 -1.853442
H -4.609285 4.405667 -2.776048
H -6.609793 0.253205 -4.142181
H -3.703022 -7.588349 -1.190363
H -1.699277 0.552495 -3.049631
H -5.083173 -4.371429 -2.195748
H -1.774533 -1.182178 -2.892061
H -2.152534 2.588716 -2.519589
H -0.660077 -6.324281 -2.008508
H -5.273937 -0.897611 -4.411954
H -2.928687 4.923367 -2.887951
H -3.595723 3.834062 -4.103730
H -5.100726 2.153128 -4.535056
H -0.410684 -4.112297 -3.188541
H -5.481803 -6.827446 -2.583447
H -3.606025 -3.068226 -3.739408
H -5.883665 0.092316 -5.742862
H -2.224530 -8.068994 -2.927382
H -2.927867 0.065846 -5.099954
H -6.104088 -5.882043 -3.939657
H 0.252875 -5.467313 -4.109841
H -5.034618 -3.670468 -4.600944
H -2.646425 1.807937 -5.012372
H -1.297332 -4.761971 -4.564279
H -4.581667 -6.745582 -4.094900

H -3.485257 -4.464066 -4.816734
H -0.820754 -7.787100 -3.958191
H -2.385362 -7.116985 -4.408272
H -3.680405 1.141451 -6.278526

Supplementary Table 45. Final coordinates and energy for 4Cs

Energy: -4167.916939 a.u
U -0.681702 -7.539118 3.342466
U -7.193690 -7.651427 -2.308952
Cs -3.335084 -5.721372 -0.244623
Cs -4.553839 -9.449663 1.226077
Si 1.948912 -5.930265 1.034741
Si -3.158708 -5.451321 5.398431
Si -0.512747 -11.337604 3.858214
Si -9.269605 -10.421461 -0.604806
Si -4.447045 -8.600487 -4.796373
Si -8.293151 -4.033487 -1.776209
N -1.533812 -6.022283 4.932895
N -0.362187 -9.643865 4.396280
N 1.065478 -7.297165 5.364233
N 1.419625 -6.749440 2.523122
N -1.924038 -7.628777 2.030813
N -8.640488 -9.529007 -2.013993
N -5.964421 -7.531580 -0.987329
N -6.015585 -7.890269 -4.334822
N -8.934475 -7.812102 -4.358223
N -8.490038 -5.678180 -2.430088
C 3.293166 -3.853759 2.643174
C 3.545646 -8.218392 0.206800
C 2.410524 -11.719465 3.593937
C -3.659991 -3.551383 3.253306
C -4.520581 -4.569 7.860203
C -5.929030 -6.066458 4.743842
C 0.764506 -4.358817 -1.100977
C 0.156045 -11.096232 1.032431
C -3.231733 -11.629554 4.842051
C 1.385572 -7.899965 -1.014235
C 2.557132 -7.181147 -0.337606
C -0.129044 -3.904181 1.200922
C 0.442978 -4.972552 0.266158
C 4.785806 -5.004583 0.977999
C 3.348904 -4.572214 1.290357
C -3.249452 -6.734769 8.043608
C -3.315216 -5.368183 7.351959
C -4.416106 -8.059093 4.985430
C -4.498039 -6.588944 4.571092
C -2.560089 -2.592215 5.298932
C -3.549212 -3.639182 4.777007
C -1.870144 -12.492699 1.519422
C -1.028017 -11.299129 1.982626
C -1.501991 -12.773965 6.246513
C -1.883473 -12.351551 4.823417
C 0.442391 -6.400406 6.358566
C -0.436610 -5.369606 5.663356
C 1.015864 -13.813587 3.712885
C 1.135539 -12.351443 4.160442
C 0.064814 -9.515531 5.796961
C 1.306147 -8.642384 5.920065
C 2.306966 -6.711534 4.819974
C 2.533931 -7.169208 3.385551
C -11.944843 -9.156344 -0.720790
C -2.673287 -6.410319 -4.093098
C -3.349874 -10.047680 -7.120352
C -2.299294 -10.103178 -3.539488
C -10.375115 -2.070627 -2.621542
C -6.144981 -2.321117 -0.838230
C -9.387756 -8.340026 1.439707
C -7.195919 -12.464298 -0.676095
C -8.346598 -10.492237 2.185207
C -8.568498 -9.554761 0.992288
C -9.417591 -13.224690 -1.536035
C -8.710776 -12.297721 -0.540245
C -11.791340 -11.320659 0.554199
C -11.225437 -10.505002 -0.614957
C -9.846103 -8.941957 -4.091884
C -9.138640 -10.035840 -3.301251
C -5.436104 -11.030222 -6.115716
C -4.656057 -9.737241 -6.378861
C -4.609284 -10.646394 -2.709843
C -3.703936 -9.550355 -3.274302
C -5.598690 -3.742480 -2.835209
C -6.400852 -3.662308 -1.534256
C -3.546755 -6.361698 -6.445874
C -3.110439 -7.262901 -5.286346
C -6.785153 -7.337987 -5.458813
C -8.135646 -8.030971 -5.580831
C -9.662488 -6.529038 -4.420024
C -9.809434 -5.925162 -3.029958
C -8.833992 -2.871485 -4.435674
C -8.969918 -2.595866 -2.934038
C -8.287202 -4.508626 1.080785
C -9.124679 -3.841909 -0.015939
C -10.553167 -4.395155 0.017853
H -2.268063 -13.434314 6.677358
H -5.471028 -5.036021 7.578796
H -4.510520 -4.505166 8.957539
H -4.659208 -8.197993 6.043512
H -6.264456 -6.139694 5.784647
H -4.193758 -7.281789 7.943107

H -0.552069 -13.317109 6.287070
H -5.140326 -8.668285 4.423386
H -3.062729 -6.623774 9.120763
H -6.636917 -6.655912 4.144152
H -1.417933 -11.910319 6.916098
H -4.535437 -3.542343 7.480078
H -4.025357 -12.266937 5.257708
H -3.421373 -8.483394 4.818246
H -6.042979 -5.021282 4.438462
H -2.457733 -7.372196 7.635113
H -2.003029 -13.272152 4.232426
H 0.151330 -14.322620 4.152536
H 1.907853 -14.388576 3.998669
H -3.184218 -10.723655 5.454818
H -2.418613 -4.811971 7.665112
H 1.244531 -12.356729 5.255940
H -0.736434 -9.091403 6.426578
H -4.239199 -6.538572 3.502068
H 0.314746 -10.478867 6.269751
H -4.537752 -3.401074 5.195975
H -3.551728 -11.321904 3.839638
H 0.924786 -13.894603 2.622920
H -0.193125 -7.001919 7.014697
H 1.650599 -8.564279 6.965576
H -2.472593 -2.604213 6.391314
H 3.306243 -12.216440 3.992157
H -2.788776 -12.623612 2.101689
H -4.439525 -4.215256 2.861806
H -0.777166 -4.661494 6.433629
H 2.488004 -10.655418 3.837812
H -2.868544 -1.577178 5.011068
H 1.221427 -5.929267 6.982183
H -3.908397 -2.529848 2.930272
H -1.313308 -13.434067 1.591856
H 2.109058 -9.107928 5.342353
H 2.457778 -11.803573 2.503716
H -1.637777 -10.385013 1.919279
H -1.556371 -2.750334 4.887794
H -2.710258 -3.822739 2.778574
H -2.158178 -12.385016 0.463002
H 0.195196 -4.763611 4.991153
H 0.789490 -11.988921 0.979250
H 3.160102 -6.959598 5.474504
H 0.790927 -10.257067 1.333064
H 2.195366 -5.624081 4.814544
H -0.417690 -4.312628 2.175096
H 2.670687 -8.264505 3.377881
H -0.191424 -10.892670 0.010397
H -1.015432 -3.417778 0.764303
H -0.316257 -5.755995 0.129639
H 0.771452 -8.433797 -0.280095
H 3.507802 -6.763371 3.067723
H 0.596417 -3.102492 1.382711
H 2.277344 -3.560101 2.923872
H 0.725368 -7.215329 -1.556991
H 3.683812 -4.484773 3.448654
H 3.066947 -8.855539 0.958653
H -0.124006 -3.889749 -1.550126
H 1.744741 -8.643735 -1.739638
H 1.143137 -5.090594 -1.821563
H 3.907071 -2.942119 2.629441
H 1.519163 -3.567028 -1.019819
H 3.904703 -8.879221 -0.594595
H 3.076612 -3.829030 0.524505
H 4.425731 -7.765763 0.673895
H 3.077112 -6.576794 -1.096689
H 5.166303 -5.727877 1.709052
H 4.880875 -5.457310 -0.014402
H 5.466852 -4.141849 1.004375
H -9.138381 -14.273244 -1.359594
H -2.665075 -10.649816 -6.512233
H -3.550075 -10.623162 -8.035207
H -4.714929 -11.486273 -3.403702
H -2.319776 -10.915321 -4.275111
H -4.825822 -11.763696 -5.576590
H -10.508920 -13.168341 -1.468822
H -4.191505 -11.062710 -1.780719
H -5.741316 -11.505737 -7.058221
H -1.860024 -10.519748 -2.622192
H -9.138397 -12.993486 -2.570473
H -2.810911 -9.143783 -7.421299
H -6.886666 -13.501223 -0.480778
H -5.616874 -10.281438 -2.485542
H -1.604651 -9.343118 -3.912714
H -6.342739 -10.860073 -5.524864
H -8.996004 -12.627324 0.470305
H -11.350470 -12.320705 0.626949
H -12.877803 -11.453786 0.454520
H -6.862703 -12.208669 -1.687147
H -5.269960 -9.131175 -7.061729
H -11.455013 -11.058410 -1.538688
H -8.317632 -10.443003 -3.916681
H -3.625584 -8.769669 -2.502605
H -9.854649 -10.867547 -3.202855
H -2.235406 -7.838592 -5.621697
H -6.639883 -11.822886 0.017710
H -11.623135 -10.817344 1.514304
H -7.959880 -9.105219 -5.689093
H -10.263759 -9.323981 -5.039232
H -3.857630 -6.931377 -7.328948

H -13.001114 -9.294135 -0.990463
H -7.677421 -11.329811 1.960440
H -2.238066 -7.020335 -3.292744
H -6.280717 -7.435709 -6.432099
H -11.494306 -8.503315 -1.474757
H -2.728935 -5.698241 -6.760784
H -8.699426 -7.692063 -6.466882
H -1.914983 -5.670652 -4.388445
H -9.288920 -10.927750 2.537509
H -10.679844 -8.570454 -3.490300
H -11.929063 -8.611228 0.227956
H -7.594510 -9.168643 0.654605
H -4.386864 -5.719589 -6.156625
H -3.525916 -5.858919 -3.678790
H -7.920647 -9.946056 3.040166
H -6.952027 -6.254100 -5.338953
H -10.358761 -8.639995 1.849039
H -10.638140 -6.670946 -4.915272
H -9.574608 -7.636994 0.622476
H -9.077300 -5.835522 -5.029447
H -5.720044 -4.705540 -3.341773
H -10.424923 -6.602756 -2.412768
H -8.867266 -7.785408 2.232337
H -4.522636 -3.590353 -2.656368
H -6.067725 -4.473032 -0.871111
H -8.115480 -5.568811 0.861354
H -10.417602 -5.012671 -3.134166
H -5.901368 -2.958473 -3.539252
H -7.852777 -3.270989 -4.709097
H -7.305456 -4.039144 1.202836
H -9.586082 -3.589262 -4.780857
H -10.560639 -5.471211 -0.187883
H -5.075823 -2.179235 -0.620132
H -8.795503 -4.453394 2.053720
H -6.685072 -2.220243 0.108398
H -8.987405 -1.951010 -5.016456
H -6.441667 -1.476979 -1.472567
H -11.008238 -4.253283 1.008272
H -8.273675 -1.776640 -2.694946
H -11.212544 -3.917572 -0.713196
H -9.169013 -2.761923 0.193255
H -11.152402 -2.807192 -2.858386
H -10.493065 -1.791159 -1.569836
H -10.596622 -1.175446 -3.220042

Supplementary Table 46. Final coordinates and energy for 5Cs

Energy: -2777.590457 a.u
U -3.591518 5.297808 -7.956864
Cs -1.911381 1.352503 -5.458364
Si -7.230455 4.579549 -7.095169
Si -2.141754 3.920906 -11.187813
Si -1.494549 7.407175 -5.582935
N -3.433933 -1.715844 -5.519831
N 1.190598 0.038381 -5.613703
N -5.971377 5.446494 -8.014023
N -2.789414 5.253253 -10.200514
N -4.345328 7.507579 -9.247001
N -2.020094 0.918906 -2.110951
N -0.829527 -1.562920 -3.707151
N -3.065060 3.811370 -7.082186
N -2.734395 7.234266 -6.851244
C -0.968631 1.324158 -1.189461
C -3.069933 -2.016271 -6.898557
C -4.884581 -1.627379 -5.405434
C -2.918028 -2.723731 -4.594051
C -1.403670 -2.725703 -4.393289
C 2.051096 0.924771 -4.840232
C 1.567051 0.103578 -7.021151
C 1.271296 -1.344195 -5.142242
C 0.638889 -1.620995 -3.777429
C -4.018487 1.950413 -10.205598
C 0.541924 3.568592 -10.150868
C -5.811875 4.178751 -4.595199
C -9.365552 4.188728 -9.099556
C -4.403427 4.334811 -13.015188
C 0.312231 5.384337 -11.852029
C -0.199009 4.006570 -11.418081
C -2.162862 4.334150 -14.153688
C -2.970970 3.792846 -12.970295
C -2.652102 9.454105 -3.804649
C -7.769946 6.975113 -5.473806
C -7.542968 2.461490 -9.042408
C -8.371010 3.439209 -8.206254
C -9.536165 5.174355 -5.394231
C -8.460333 5.851195 -6.250219
C -7.216890 2.230560 -5.343277
C -6.363123 3.416902 -5.803911
C -6.536189 6.370918 -9.001922
C -5.789083 7.697698 -9.012697
C -1.839087 1.039096 -11.052577
C -2.517481 2.214113 -10.339003
C -2.758195 6.547752 -10.887687
C -4.059806 7.310768 -10.681299
C -3.569174 8.638605 -8.703754
C -3.366515 8.509407 -7.200148
C -0.030433 5.486094 -3.912132
C -3.203002 1.741841 -1.890878
C -2.377693 -0.492141 -1.978178
C -1.275373 -1.502354 -2.303570

C 0.316849 5.296979 -6.398948
C -0.671313 5.671560 -5.291177
C 0.955915 8.921350 -5.072893
C -3.347311 7.074451 -3.391235
C -2.210632 7.987808 -3.856680
C -0.189249 8.779618 -6.081569
C 0.348280 8.647896 -7.508813
H -2.972958 9.733120 -2.790601
H -9.105120 4.698102 -4.505444
H -10.274451 5.905519 -5.034344
H -6.616008 4.529554 -3.937174
H -8.130564 2.551448 -4.829229
H -7.323214 6.610273 -4.543111
H -1.850833 10.144926 -4.088913
H -5.161525 3.535339 -3.985389
H -8.484036 7.765103 -5.200598
H -6.659946 1.603974 -4.630372
H -3.502538 9.644720 -4.469205
H -10.088659 4.403178 -5.943256
H -3.681920 7.330441 -2.375537
H -5.217658 5.049133 -4.888437
H -7.520051 1.581686 -6.171611
H -6.966155 7.437443 -6.055371
H -1.373244 7.874274 -3.151273
H 0.595756 9.068519 -4.048260
H 1.595307 9.781764 -5.317291
H -4.215387 7.163891 -4.053607
H -8.977753 6.323134 -7.099202
H -0.760587 9.719671 -6.045898
H -4.339123 8.639716 -6.693044
H -5.495767 3.026936 -6.357245
H -2.775703 9.392500 -6.902013
H -8.961182 2.850898 -7.486899
H -3.057836 6.017448 -3.386174
H 1.604595 8.036880 -5.069750
H -5.909091 8.164404 -8.031007
H -4.061707 9.591843 -8.965321
H -10.025956 4.853372 -8.531457
H 0.921958 9.539053 -7.800982
H -0.743500 5.630856 -3.094043
H -6.842976 1.880513 -8.431899
H -7.592932 6.627103 -8.813530
H -0.459397 8.517671 -8.235895
H -10.010821 3.484360 -9.644291
H -6.203639 8.390863 -9.765926
H -8.184316 1.747684 -9.579174
H 0.803807 6.177040 -3.743494
H -2.586983 8.631958 -9.184482
H 1.016216 7.786769 -7.616217
H -1.522849 4.980147 -5.376661
H -8.854817 4.797767 -9.853965
H -6.948366 2.994502 -9.792072
H 0.381113 4.470558 -3.805444
H -6.528521 5.941482 -10.019346
H -4.045875 8.286824 -11.197974
H -0.109364 5.444767 -7.395860
H -4.875095 6.719670 -11.106373
H -4.529882 2.753018 -9.666541
H -1.916889 7.176477 -10.549493
H 0.615363 4.240504 -6.328716
H -4.211878 1.015894 -9.658739
H -2.106208 2.299095 -9.323582
H 0.298371 4.225437 -9.308724
H -2.621107 6.463608 -11.979383
H -4.498441 1.848735 -11.186741
H -5.007084 4.406000 -12.166380
H 0.291494 2.546672 -9.848731
H -4.414982 5.430296 -13.007832
H 0.137148 6.129202 -11.067787
H -2.017203 0.096246 -10.514380
H 1.631658 3.611025 -10.292967
H -0.754562 1.159454 -11.145312
H -4.915710 4.017608 -13.934890
H -2.235409 0.897085 -12.065961
H 1.395652 5.362040 -12.038897
H -3.037386 2.702401 -13.110931
H -0.166511 5.749428 -12.765514
H 0.036519 3.286915 -12.217659
H -2.080805 5.427386 -14.128528
H -1.146723 3.927482 -14.190666
H -2.647306 4.077123 -15.106964
H -5.167104 -1.343778 -4.386312
H -5.268220 -0.862035 -6.087313
H -5.396196 -2.578198 -5.646159
H -1.984090 -2.008168 -7.029886
H -3.442412 -3.002588 -7.235095
H -3.489708 -1.257972 -7.566985
H 1.495794 1.134802 -7.380573
H 2.600388 -0.244552 -7.207312
H 0.892943 -0.513808 -7.624409
H 1.973051 1.945846 -5.226125
H 1.751688 0.948382 -3.789124
H 3.116599 0.628985 -4.883465
H -1.232090 1.145730 -0.129355
H -0.034872 0.795560 -1.397922
H -0.771098 2.393862 -1.309998
H -2.959798 2.798719 -2.038773
H -3.991384 1.469134 -2.600263
H -3.620783 1.632835 -0.872003
H -3.237152 -0.668358 -2.633240

H -2.718449 -0.713542 -0.945279
H -0.411499 -1.299458 -1.662091
H -1.643478 -2.489156 -1.975958
H -3.415664 -2.582064 -3.628967
H -3.197442 -3.742308 -4.930145
H -0.921590 -2.826719 -5.369317
H -1.159814 -3.655143 -3.840113
H 0.810709 -1.981353 -5.904816
H 2.327915 -1.673194 -5.077674
H 1.009716 -2.612406 -3.449715
H 1.037305 -0.900758 -3.057215
H 1.237715 5.889653 -6.339301

Supplementary Table 47. Final coordinates and energy for 6Cs

Energy: -2064.988573
U 0.143460 0.008287 0.009544
Si 0.882260 -2.946330 -2.325939
Si 0.388018 -0.425586 3.786404
Si -0.667181 3.426977 -1.452477
N 1.393067 -1.672577 -1.213021
N 1.019299 0.163817 2.249153
N 2.811578 0.653387 -0.032761
N 0.473774 2.124589 -1.089930
N -2.156044 -0.364501 0.057055
C 1.861378 -4.482794 -4.621393
C 2.304936 -3.441191 -3.587526
C 1.748666 -0.931877 5.121608
C 2.063531 0.087491 6.221173
C 2.990205 -2.260960 -4.281668
C 3.053702 -1.464892 4.517390
C 2.776934 -1.733534 -0.736809
C 3.472112 -0.375069 -0.849287
C 3.271399 0.641749 1.363120
C 2.160934 1.077146 2.320081
C 2.902440 1.989029 -0.639587
C 1.807567 2.213399 -1.685107
C -2.024907 3.454749 -0.062529
C -1.497965 3.999490 1.268005
C -3.334585 4.154824 -0.442288
C -0.723643 -2.341754 -3.246326
C -1.601286 -3.454976 -3.830238
C -0.428467 -1.290895 -4.321033
C 0.413601 -4.633985 -1.450766
C -0.723517 -4.441005 -0.445636
C 1.610509 -5.308660 -0.773703
C -1.615637 3.178267 -3.147084
C -2.415361 1.873806 -3.139307
C -0.678406 3.223346 -4.357224
C 0.242501 5.155914 -1.655835
C 1.170848 5.540132 -0.499514
C -0.714721 6.309567 -1.975614
C -0.615022 -2.072170 3.498158
C -1.462257 -2.477248 4.710237
C 0.296292 -3.237646 3.100604
C -0.845562 0.841862 4.630943
C -0.244046 2.245303 4.757799
C -2.162916 0.926014 3.851811
H -1.229140 3.079053 -5.298714
H 1.138392 -4.063702 -5.331822
H 2.716303 -4.839481 -5.215259
H 0.088287 -1.733132 -5.181726
H -1.094027 -3.998252 -4.636770
H 2.313450 -1.744265 -4.970043
H -0.144514 4.177349 -4.439754
H -1.355375 -0.843009 -4.705210
H 3.855424 -2.597651 -4.872525
H -2.522212 -3.035970 -4.261266
H 0.076245 2.429838 -4.303258
H 1.397651 -5.364285 -4.164372
H -2.947785 1.718255 -4.089447
H 0.197844 -0.479220 -3.937229
H -1.905293 -4.193542 -3.081947
H 3.349708 -1.514804 -3.567472
H -2.324637 4.015589 -3.235269
H -1.373426 6.087536 -2.822300
H -0.161435 7.226438 -2.228185
H -1.751352 1.015094 -2.988334
H 3.067194 -3.924739 -2.958025
H 0.878339 5.018898 -2.543892
H 1.950520 1.475375 -2.495931
H -1.306452 -1.850476 -2.450760
H 2.020914 3.188944 -2.155677
H 0.057420 -5.302360 -2.249609
H -3.161333 1.847653 -2.337549
H -1.353614 6.553617 -1.118441
H 3.415468 -0.058099 -1.895436
H 3.908642 2.153691 -1.071309
H 2.417564 -5.541561 -1.477879
H 1.779226 6.420950 -0.754625
H -3.790026 3.739149 -1.347006
H -1.622297 -4.011006 -0.900472
H 3.407732 -2.449180 -1.294430
H 1.858486 4.732630 -0.231322
H 1.318787 -6.254929 -0.294064
H 4.544012 -0.452798 -0.583785
H -1.013688 -5.391876 0.026196
H -3.195519 5.229512 -0.613117
H 2.760605 2.729158 0.154655
H 0.607394 5.792793 0.405112

H -2.248626 2.386118 0.088191
H 2.034452 -4.671084 0.011019
H -0.422003 -3.754837 0.352563
H -4.075437 4.056666 0.364604
H 2.841819 -2.067213 0.316025
H -1.351393 5.086390 1.230135
H 4.176166 1.269189 1.481583
H -0.541495 3.541627 1.539401
H 3.545217 -0.386065 1.621052
H 0.925364 -2.991383 2.239162
H 1.877171 2.116634 2.073380
H -2.204140 3.802228 2.086046
H -0.289544 -4.130236 2.841361
H -1.293920 -1.876462 2.654725
H -1.984562 1.226696 2.812484
H 2.614767 1.142471 3.324893
H 0.958562 -3.522775 3.927871
H 2.886210 -2.175186 3.702971
H -2.702946 -0.026082 3.823786
H 3.668005 -0.652102 4.114172
H -0.005926 2.651767 3.769139
H -2.039080 -3.389475 4.500744
H -2.841743 1.669738 4.295282
H -2.176370 -1.704913 5.012658
H 3.661678 -1.972321 5.281336
H -0.833939 -2.696810 5.583872
H -0.952783 2.941513 5.230543
H 1.258151 -1.782532 5.622011
H 0.675116 2.265971 5.350990
H -1.059820 0.459209 5.641528
H 2.579231 0.971247 5.826651
H 1.164422 0.434529 6.741334
H 2.726774 -0.350945 6.982429
H -2.739681 -0.613353 0.853580
H -2.793894 -0.253119 -0.729876
